# Catalytic and stoichiometric stepwise conversion of side-on bound dinitrogen to ammonia mediated by a uranium complex

**DOI:** 10.1038/s41557-025-01867-z

**Published:** 2025-07-16

**Authors:** Mikhail S. Batov, Heather T. Partlow, Lucile Chatelain, John A. Seed, Rosario Scopelliti, Ivica Zivkovic, Ralph W. Adams, Stephen T. Liddle, Marinella Mazzanti

**Affiliations:** 1 Group of Coordination Chemistry, Institut des Sciences et Ingénierie Chimiques, École Polytechnique Fédérale de Lausanne (EPFL), Lausanne, Switzerland; 2 Department of Chemistry, The University of Manchester, Manchester, UK; 3 Chimie, Electrochimie Moléculaires et Chimie Analytique, Université de Bretagne Occidentale, Brest, France; 4 Laboratory for Quantum Magnetism, Institute of Physics, Ecole Polytechnique Fédérale de Lausanne (EPFL), Lausanne, Switzerland

**Keywords:** Ligands, Homogeneous catalysis

## Abstract

The well-defined catalytic conversion of dinitrogen (N_2_) to ammonia (NH_3_) by molecular complexes is of fundamental interest and important for providing an atomic-level understanding of reactivity that can be related to industrial and biological nitrogen-fixation processes. Molecular catalytic N_2_ to NH_3_ conversion currently involves the reduction and protonation of terminal or bridging end-on bound metal–N_2_ complexes. However, catalytic N_2_ to NH_3_ conversion by side-on bound metal–N_2_ molecular complexes is relevant to both the industrial and biological nitrogen-fixation processes. Here, using a uranium triamidoamine complex, we report catalytic N_2_ to NH_3_ conversion involving side-on bound N_2_ binding. Stoichiometric reactions reveal stepwise reduction of N_2_ from free N_2_ to bridging side-on bound forms and subsequently to bridging nitrides, uniquely accessing four different states of side-on bound N_2_ for the same molecular system. This reveals the roles of N_2_, N_2_^2−^, N_2_^3−^, N_2_^4−^ and N^3−^ in the catalytic conversion of N_2_ to NH_3_ when involving side-on bridging N_2_.

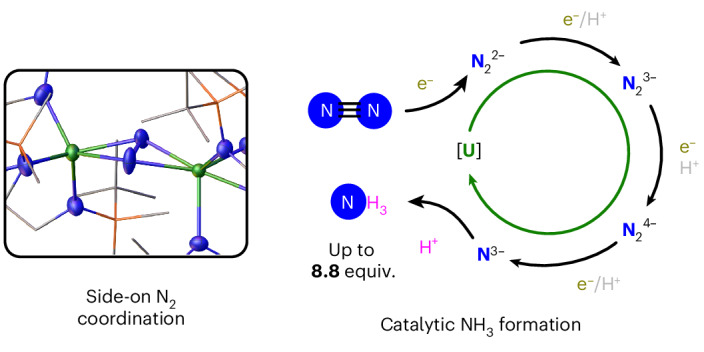

## Abstract (web summary)

The catalytic conversion of nitrogen to ammonia by molecular complexes typically involves the reduction and protonation of end-on metal–N_2_ compounds. Now, a uranium complex has been shown to catalyse the conversion of N_2_ to ammonia through the formation of a side-on bound dinitrogen complex. Four different states of side-on bound dinitrogen and the cleavage to nitride have been observed.

## Main

The well-defined catalytic conversion of dinitrogen (N_2_) to ammonia (NH_3_)—promoted by a molecular Mo–triamidoamine complex—was reported by Schrock in 2003 (ref. ^1^). The proposed mechanism involved the reduction of terminal end-on bound N_2_ by the stepwise, alternating addition of six protons and six electrons. Since that seminal report, a broad range of mononuclear and dinuclear Fe and Mo complexes^2–4^ capable of binding and catalytically converting N_2_ into NH_3_ have been identified^5,6^, with some reaching very high conversion efficiencies^7^. Since the discovery of the Fe–Mo cofactor, numerous mechanistic studies, mostly focused on Mo and Fe due to their biological relevance, have been carried out on metal–N_2_ complexes as models for the biological reduction of N_2_ (refs. ^6,8–10^). Although synthetic model chemistry—even based on Fe and Mo—does not provide direct mechanistic information on enzymatic species, such studies can provide relevant models and permit unforeseen reactivity patterns to be recognized. The catalytic conversion of N_2_ to NH_3_ has subsequently expanded beyond Mo and Fe, but involves a still exceedingly small number of metals (Ti, Co, V, Ru and Os)^11–14^.

The plethora of reported mechanistic studies on molecular catalytic N_2_ to NH_3_ conversion involve the reduction and protonation of end-on terminal or end-on/end-on bridging metal–N_2_ complexes^5,6,8^. However, the recent identification of intermediates of nitrogenase reactive sites where substrates such as CO or imide bridge—with only one atom—two neighbouring Fe centres highlights the need for more studies on dinuclear N_2_-bridged complexes^15–17^. Furthermore, the reduction and hydrogenation of side-on bound systems is also potentially relevant to gaining a better understanding of the mechanism of the Haber–Bosch process for converting N_2_ and hydrogen gas (H_2_) into NH_3_ (refs. ^18–20^). Although the catalytic N_2_ to NH_3_ conversion for a side-on bound N_2_ complex remains elusive, stoichiometric N_2_ to NH_3_ conversion has been reported for a few side-on bridged N_2_ complexes of group 4 metals^21,22^ and uranium^23–28^. Side-on coordinated N_2_ can undergo stepwise reduction to a variety of formal charge states including N_2_^2−^, N_2_^3−^ and N_2_^4−^, but in most cases only one of these activated N_2_ species is isolated for a given metal complex^29^. In f-element chemistry where side-on N_2_ complexes dominate, interconversion between N_2_^2−^ and the N_2_^3−^ radical is documented^26,30–33^, but further reduction could not be observed for lanthanide ions. Nonetheless, cleavage of side-on bound N_2_^4−^ to 2N^3−^ following the addition of external reducing agents has also been reported by some of us for U-complexes^23,26,34^, which suggested to us that under suitable conditions, side-on coordinated bridging U–N_2_ complexes^35–38^ might prove to be competent for catalytic N_2_ to NH_3_ conversion. It is also germane to recall that before the use of Fe-based catalysts in the industrial Haber–Bosch process, Haber reported that uranium and uranium nitride materials (UN_*x*_) were effective heterogeneous catalysts for the production of ammonia from dinitrogen^39^. Accordingly, studies on molecular analogues may afford insights into heterogeneous scenarios.

**Fig. 1:**
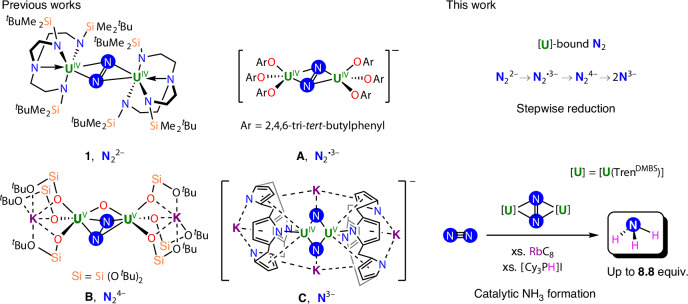
Selected literature examples (1, A–C) of N_2_ binding and reduction by uranium complexes, as well as a summary of this work. Previously reported N_2_^2−^-bridged complexes: [{U^IV^(Tren^DMBS^)}_2_(*μ-η*^2^:*η*^2^*-*N_2_)] (**1**, Tren^DMBS^ = {N(CH_2_CH_2_NSiMe_2_^*t*^Bu)_3_}^3−^)^35^; N_2_^3−^ radical-bridged aryloxide complex [K(2.2.2-crypt)][{(ArO)_3_U^IV^}_2_(*μ-η*^2^:*η*^2^-N_2_)] (**A**, OAr = 2,4,6-tri-*tert*-butylphenoxide)^26^; N_2_^4–^-bridged siloxide complex [K_2_{[U^V^(OSi(O^*t*^Bu)_3_)_3_]_2_(*μ-*O)(*μ-η*^2^:*η*^2^-N_2_)}] (**B**)^25^; bis-nitride bridged calix[4]tetrapyrrole complex [{K(DME)(Et_8_-calix[4]tetrapyrrole)U}_2_(*μ-*NK)_2_][K(DME)_4_] (**C**, DME = 1,2-dimethoxyethane)^78^; this work: stepwise reduction of **1** and catalytic conversion of N_2_ to ammonia by **1**. xs., excess.

Given our earlier collective work in early metal and f-block N_2_-activation^23,25–27,33,40–43^ and U-nitride chemistries^26,34,44–65^, we developed our investigations using the diuranium–N_2_ complex [{U^IV^(Tren^DMBS^)}_2_(*μ-η*^2^*:η*^2^*-*N_2_)] (**1**, Tren^DMBS^ = {N(CH_2_CH_2_NSiMe_2_^*t*^Bu)_3_}^3−^), reported by Scott^35^ in 1998 (Fig. 1), as the formation of **1** from its trivalent precursor [U^III^(Tren^DMBS^)] is reversible, suggesting that it could be amenable to catalysis rather than being an irreversible thermodynamic sink.

In this Article we report the stepwise reduction of N_2_—from free-N_2_ to bridging side-on bound forms to bridging nitrides—overall uniquely accessing four different states of side-on bound N_2_ for the same dinuclear U-complex. Experimental characterization and quantum-chemical studies suggest that the addition of reducing agents leads to the formal sequential reduction of the bound N_2_ rather than the U-centres. We establish molecular U-mediated catalytic N_2_ to NH_3_ conversion, moreover involving side-on bound coordination of N_2_ in metal-mediated N_2_ to NH_3_ catalysis. The stoichiometric reactions suggest the relevance of N_2_, N_2_^2−^, N_2_^3−^, N_2_^4−^ and N^3−^ in the catalytic conversion of N_2_ to NH_3_ when involving side-on bridging N_2_.

## Results and discussion

### Synthesis and characterization

**Fig. 2:**
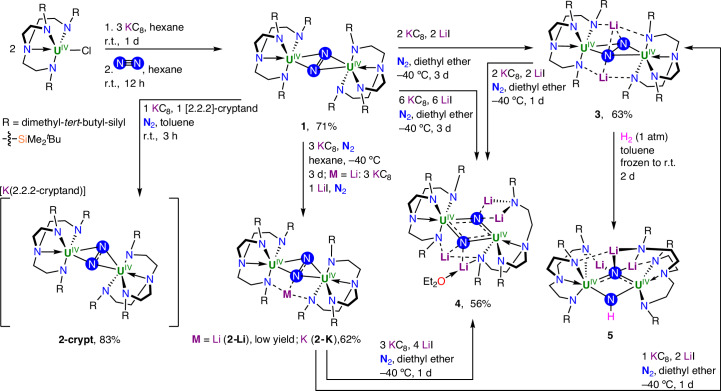
Synthesis of uranium–dinitrogen complexes 1–5. Synthesis of the previously reported pernitride-bridged complex [{U^IV^(Tren^DMBS^)}_2_(*μ-η*^2^:*η*^2^*-*N_2_)], **1**, from [U^IV^Tren^DMBS^)Cl] via a modified procedure, followed by reduction of **1** to yield the N_2_^3−^ radical-bridged complexes [K(2.2.2-cryptand)][{U^IV^(Tren^DMBS^)}_2_(*μ*-*η*^2^:*η*^2^-N_2_)] (**2-crypt**), [Li{U^IV^(Tren^DMBS^)}_2_(*μ*-*η*^2^:*η*^2^-N_2_)] (**2-Li**) and [K{U^IV^(Tren^DMBS^)}_2_(*μ*-*η*^2^:*η*^2^-N_2_)] (**2-K**). Further reduction yields the hydrazido(4–)-bridged complex [Li_2_{U^IV^(Tren^DMBS^)}_2_(*μ*-*η*^2^:*η*^2^-N_2_)] (**3**) and ultimately leads to splitting of the N_2_ molecule, affording the bis-nitride complex [Li_4_(OEt_2_){U^IV^(Tren^DMBS^)}_2_(*μ*-N)_2_] (**4**). Cleavage of the hydrazido moiety is observed upon reaction of **3** with dihydrogen, and allows the isolation of the nitrido/imido-bridged complex [Li_3_{U^IV^(Tren^DMBS^)}_2_(*μ*-N)(*μ*-NH)] (**5**).

Complex **1** (Fig. 2), was prepared from hexane in 71% yield through a modified procedure, circumventing the sublimation step of the previously reported synthesis^35^.

**Fig. 3:**
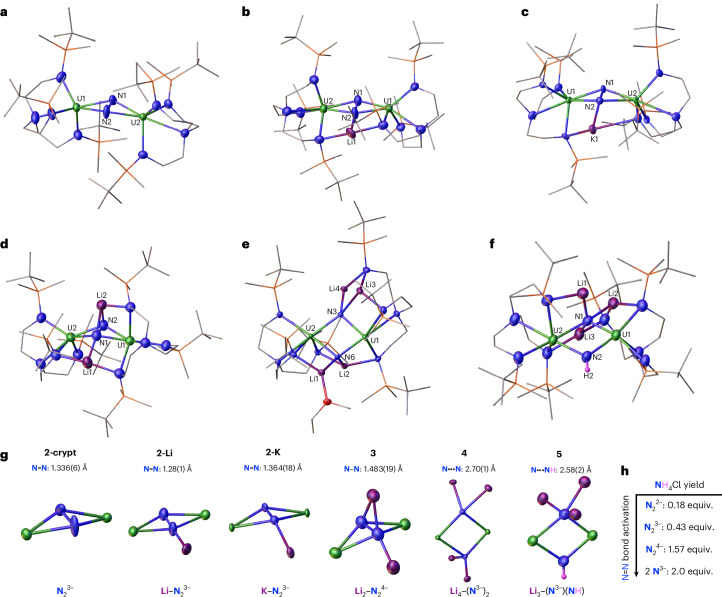
Single-crystal X-ray diffraction solid-state structures of the dinitrogen-bridged Tren^DMBS^ diuranium complexes with displacement ellipsoids at 50% probability level and selective labelling. **a**–**f**, Molecular structures of the anionic complex [{U^IV^(Tren^DMBS^)}_2_(*μ-η*^2^:*η*^2^*-*N_2_)]^−^ in **2-crypt** (**a**), as well as in **2-Li** (**b**) and **2-K** (**c**), the hydrazido-bridged complex **3** (**d**) with disorder in the dinitrogen core and with one lithium position removed for clarity, the bis-nitride complex **4** (**e**) and the nitrido/imido complex **5** (**f**). For all complexes, the triamidoamine ligand framework is depicted as a wireframe, and hydrogen atoms (apart from the imido-bound H in **5**) and disordered *tert*-butyl groups have been removed for clarity. **g**, Molecular U_2_N_2_ core structures and XRD-derived dinitrogen distance summaries for complexes **2**–**5**. **h**, Correlation between the degree of N–N bond activation and the NH_4_Cl yields obtained after direct quenching with 2 M HCl in diethyl ether.

The redox reactivity of **1** was then explored (Fig. 2). After sequential addition of 1 equiv. of 2.2.2-cryptand and then KC_8_ to a brown-red solution of **1** in toluene, the mixture turned dark-brown, and full consumption of the starting material occurred after 3 h of stirring at room temperature (r.t.). Brown-black crystals of the mono-reduced complex [K(2.2.2-cryptand)][{U^IV^(Tren^DMBS^)}_2_(*μ-η*^2^:*η*^2^*-*N_2_)] (**2-crypt**) were isolated in 83% yield from the filtered reaction mixture when stored at −40 °C for 2 days (Fig. 3a and Supplementary Fig. 2). The N1–N2 distance of 1.336(6) Å in **2-crypt** is significantly longer compared to that found in **1** (1.109(7) Å), the latter having an N=N distance close to that of the free N_2_ (1.0975 Å) (Supplementary Table 1). The longer N–N distance in **2-crypt** indicates reduction at the central N_2_ rather than at the uranium ions, and the U1–N_2_–U2 unit is bent (fold angle of 157.43°, Fig. 3g) compared to the essentially planar angle of 177.83° in **1**. Elongation of the N1–N2 distance in **2-crypt** is associated with population of its antibonding π* orbital and formation of the N_2_^3−^ radical. Similar values of the N–N distance were found for the only other N_2_^3−^ radical-bridged diuranium system [K(L)][{(ArO)_3_U^IV^}_2_(*μ-η*^2^:*η*^2^-N_2_)] (for L = 2.2.2-cryptand, 1.425(5) Å; for L = (THF)_6_, 1.39(1) Å, THF = tetrahydrofuran; ArO = 2,4,6-^*t*^Bu-C_6_H_2_O, Fig. 1, **A**)^26^, as well as for the N_2_^3−^ radical-bridged lanthanide complexes, which show N=N distances in the range of 1.396(7)–1.405(3) Å (refs. ^66,67^). The U–N_amide_ distances for **2-crypt** (average 2.238 Å) are close to those of [U^IV^(Tren^DMBS^)Cl] (average 2.22 Å)^68^, and the U–N_amine_ distance (average 2.743 Å) is longer than in [U^IV^(Tren^DMBS^)Cl] (2.656(19) Å). In contrast to **1**, ^1^H NMR studies showed that **2-crypt** is stable towards N_2_ loss under dynamic vacuum in the solid state and after three freeze–pump–thaw cycles in toluene-*d*_8_ solution, and no N_2_ displacement was evidenced by exposure to coordinating solvents such as tetrahydrofuran and diethyl ether.

Reduction of **1** was also performed without chelating agents. Dark-brown microcrystals of [K{U^IV^(Tren^DMBS^)}_2_(*μ-η*^2^:*η*^2^*-*N_2_)] (**2-K**) were obtained in 62% yield from the filtered reaction mixture left standing at −40 °C for 2 days. When the reduction was performed under similar conditions but with adventitious Li cations present, the complex [Li{U^IV^(Tren^DMBS^)}_2_(*μ-η*^2^:*η*^2^*-*N_2_)] (**2-Li**) was isolated after 3 days of storage at −40 °C. The N1–N2 distance (1.28(1) Å) in complex **2-Li** is shorter than the N–N distances in **2-K** (1.364(18) Å) and in **2-crypt** (1.336(6) Å), suggesting stronger N–N activation in the K- and K-crypt complexes.

Magnetic susceptibility data were collected for **1** and **2-crypt** under an applied field of 0.1 T and in a temperature range of 2–300 K. The susceptibility data for **1** indicate the presence of two noninteracting 5*f*^2^ U(IV) centres, with a magnetic moment of 0.8 *μ*_B_ at 2.5 K, 4.2 *μ*_B_ at 300 K (0.6 *μ*_B_ at 2.5 K and 3.0 *μ*_B_ at 300 K per U ion), in agreement with the assignment of **1** as a pernitride-bridged U(IV)/U(IV) complex^69^ (Supplementary Figs. 137–140). By contrast, the magnetic data for **2-crypt** show that the magnetic moment of the complex increases from 4.10 *μ*_B_ (2 K) to 4.90 *μ*_B_ (300 K) (Supplementary Fig. 143). Similar data were obtained for **2-K** (per complex, 3.94 *μ*_B_ at 2 K and 6.84 *μ*_B_ at 300 K under 0.1 T applied field; 3.24 *μ*_B_ at 2 K and 5.12 *μ*_B_ at 300 K under 1 T applied field; Supplementary Fig. 149). These values are increased compared to **1** across the entire temperature range, reflecting the presence of an *S* = ½ radical in addition to two 5*f*^2^ U(IV) ions (Supplementary Fig. 146).

The *χ* versus *T* data for **2-crypt** and **2-K** do not exhibit any maxima feature that could be associated with U···U magnetic exchange coupling (Supplementary Fig. 142). However, the variable temperature behaviour is also not as expected for isolated spins. Interestingly, the magnetic data for **2-crypt** and **2-K** are similar to those found for the N_2_^3−^ radical complex [K(2.2.2-crypt)][{(ArO)_3_U^IV^}_2_(*μ-η*^2^:*η*^2^-N_2_)] (1.8 K, 3.6 *μ*_B_; 300 K, 5.0 *μ*_B_)^26^. Powdered **1** was found to be electron paramagnetic resonance (EPR)-silent (X-band) at 6 and 298 K, which agrees with its U(IV)/U(IV)-N_2_^2−^ formulation^70^. In contrast, the X-band EPR spectrum of **2-crypt**, although silent at 298 K, shows a single axial feature at *g* = 8.6 (*H*_0_ = 780 Gs; *g* = Landé *g-*factor, *H*_0_ = magnetic field) both in solution and in frozen 2-methyltetrahydrofuran glass at 6 K, indicating that the entire molecular species possesses a Kramers ground state (Supplementary Figs. 134 and 135)^71^. A better-resolved EPR signal was observed for the inner-sphere N_2_^3−^ radical complex **2-K** in the solid state at 6 K, with *g*_1_ = 7.9 (*H*_0_ = 850 Gs), *g*_2_ = 0.73 (*H*_0_ = 9,205 Gs) and *g*_3_ ~ 0.5 (*H*_0_ = 1,330 Gs) (Supplementary Fig. 136). Although EPR signals were not previously observed for the radical-bound U-complexes [{(SiMe_2_NPh)_3_-tacn}U^IV^(*η*^2^-N_2_Ph_2_)]^72^ and [{(^*t*Bu^ArO)_3_tacn}U^IV^(*η*^2^-NNCPh_2_)] (tacn = 1,4,7-triazacyclononane)^73^, nor the N_2_^3−^ radical-bridged diuranium complex [K(2.2.2-crypt)][{(ArO)_3_U}_2_(*μ-η*^2^:*η*^2^-N_2_)]^26^, the magnetic and anisotropic EPR data of **2-crypt** and **2-K** are similar to those of the diphosphorus radical trianion diuranium(IV) complex [K(2.2.2-crypt)][{U(Tren^TIPS^)}_2_(*μ-η*^2^:*η*^2^-P_2_)] (Tren^TIPS^ = {N(CH_2_CH_2_NSi^*i*^Pr_3_)_3_}^3−^). The latter was found to have a strong U–P radical, but very weak U···U magnetic coupling^74^.

Overall, the magnetic and EPR data are in agreement with the presence of two magnetically independent U(IV) ions bridged by an N_2_^2−^ in **1**, and the presence of two N_2_^3−^-bridged U(IV) ions in **2-crypt** and **2-K** with a strong uranium radical but weak U···U magnetic exchange coupling.

Having established that the mono-reduced N_2_ analogues of **1** are synthetically accessible, we probed the further reduction of the N_2_ unit (Fig. 2). To accommodate the increased steric and electronic loadings anticipated from further reductions, and thus facilitate the isolation of the reduced species, we carried out reductions in the presence of added Li cations. Addition at −40 °C of a solution of **1** and 2 equiv. of LiI in diethyl ether to a suspension of 2 equiv. of KC_8_ resulted in a colour change from brown-red to maroon over 3 days at −40 °C. After workup, dark-red crystals of [Li_2_{U^IV^(Tren^DMBS^)}_2_(*μ-η*^2^:*η*^2^*-*N_2_)] (**3**) were obtained after 4 days in 63% yield. The solid-state structure of **3** shows the presence of a central hydrazido(4−) moiety bridging two U ions disordered over two orientations (Fig. 3d and Supplementary Figs. 5 and 6). The N–N bond (Fig. 3g) is substantially elongated compared to **1** and **2-crypt** and is within the same range for both orientations (N1–N2 = 1.483(19); N3–N4 = 1.47(2) Å), and essentially the same as free hydrazine (N–N = 1.47 Å). This value falls within the range of the N–N bond distance values found in U(IV) and U(V) hydrazido complexes, namely 1.491(5) Å in K_4_[U^IV^_2_(*μ-*N_2_H_2_)(mTP^R−^)_2_] (mTP = [{2-(OC_6_H_2_-^*t*^Bu-2,Me-4)_2_CH}-C_6_H_4_-1,3]^4−^; R = Me^*t*^,Bu), 1.40(1) Å (ref. ^24^) in [K_2_{[U^V^(OSi(O^*t*^Bu)_3_)_3_]_2_(*μ-*O)(*μ-η*^2^:*η*^2^-N_2_)}]^25^ (Fig. 1, **B**) and 1.521(18) Å in [K_3_{[U^V^(OSi(O^*t*^Bu)_3_)_3_]_2_(*μ-*N)(*μ-η*^2^:*η*^2^-N_2_)}^23^. Similar N–N distances were also found in Zr(IV) hydrazido complexes (1.457(3)–1.548(7) Å)^21,75^. The metrical parameters of bonding between the triamidoamine framework and the U-centres in **3** (U–N_amide_ = 2.402 Å and U–N_amine_ = 2.628 Å) are similar to those found in **2-Li**, in agreement with the presence of U(IV) ions (Supplementary Table 1).

With the products of mono- and di-reduction of **1** in hand, we pursued further reduction to split the N_2_ molecule and access nitrides. When an excess of 6 equiv. of KC_8_ was added to the mixture of **1** and LiI (6 equiv.) and left stirring for 3 days at −40 °C, the colour of the reaction mixture gradually turned dark-brown. Subsequent workup and crystallization at −40 °C afforded the bis-nitride complex [Li_4_(OEt_2_){U^IV^(Tren^DMBS^)}_2_(*μ-*N)_2_] (**4**) as brown-black crystals in 56% yield (Fig. 3e). The solid-state structure of **4** shows two U-centres bridged by two nitrides (N6 and N3) at a non-bonding N···N distance of 2.70(1) Å in a diamond core U_2_N_2_ motif (Fig. 3g) with U–N bond distances of U2–N3 = 2.248(9) Å and U2–N6 = 2.184(9) Å (Supplementary Fig. 7 and Supplementary Table 1). Each nitride binds two Li cations, with two different binding modes: the Li cations interacting with N6 are coordinatively supported by two U-bound amide arms (Li1–N6 = 1.96(2); Li2–N6 = 1.98(2) Å), and the two cations capping the second nitride are held by one Tren^DMBS^ arm that is dissociated from U (Li3–N3 = 2.16(3); Li3–N3 = 1.97(2) Å).

Complex **4** adds to the limited examples of U-nitrides obtained from the reductive cleavage of N_2_ (refs. ^27,34,76,77^) following the first report (Fig. 1, **C**)^78^ with only one example of a U(IV) bis-nitride obtained from cleavage of N_2_ having been reported^77^. Alternatively, brown-black crystals of **4** could be isolated through reduction of **2-K** with 3 equiv. of KC_8_ and 4 equiv. of LiI for 1 day at −40 °C in diethyl ether, or from **3** and 2 equiv. of LiI with the addition of 2 equiv. of KC_8_ after 1 day under similar experimental conditions (Supplementary Figs. 24 and 25). Furthermore, it was found that **3** could in turn be accessed from **2-K** by reaction with 1 equiv. of KC_8_ and 2 equiv. of LiI (diethyl ether at −40 °C, 1 day; Supplementary Figs. 20 and 21). These results highlight the possibility of controlled, stepwise addition of electrons into the side-on bound N_2_ by controlling the amount of external reducing agent. Although the stepwise reduction of end-on bound N_2_ in molecular complexes has been studied extensively^6^, here four different products of N_2_ reduction (N_2_^2−^, N_2_^3−^, N_2_^4−^, N^3−^) have all been isolated for the same ligand system and for the same metal, and all have been shown to be accessible in a stepwise controlled manner. Recently, some of us reported the stepwise reduction of the U_2_–N_2_ complex [{(ArO)_3_U^IV^}_2_(*μ-η*^2^:*η*^2^*-*N_2_)]^26^, but in that case reduction of the N_2_^3−^ analogue resulted in the immediate splitting of N_2_, and identification of a potential hydrazido(4−) intermediate was not possible under conditions similar to those used here.

Finally, we explored the reactivity of complexes **3** and **4** with H_2_ (1.01 bar) at room temperature. X-ray-quality crystals of the nitrido/imido complex complex [Li_3_{(Tren^DMBS^)U^IV^}_2_(*μ*-N)(*μ*-NH)] (**5**) could be isolated in good yield (Supplementary Figs. 27 and 28) from the reaction mixture, indicating that H_2_ can cleave the hydrazido(4−) moiety at room temperature. The solid-state structure of **5** (Fig. 3f) reveals an asymmetric diamond-shape U_2_–N_2_ core (Fig. 3g) with disparate U–N_nitride_ distances (U2–N5 = 2.35(1); U1–N5 = 2.02(1) Å) (Supplementary Fig. 8). Loss of one Li-cation is observed following the protonation of the N6 nitrido to give an imido-group (U2–N6 = 2.21(1); U1–N6 = 2.14(1) Å).

Cleavage of metal-bound N_2_ by H_2_, a reaction directly relevant to the Haber process, has only been reported for group 4 ions at substantially higher temperatures^21^. To unambiguously confirm that the imido- proton originates from the H_2_ gas, complex **3** was reacted with D_2_. An immediate reaction was observed, producing **5** as a major species, with the ^1^H NMR spectrum of the crude reaction mixture dissolved in toluene-*d*_8_ showing the full set of resonances assigned to **5** (Supplementary Figs. 31 and 32), apart from the broad N*H* singlet at −170.25 ppm. The structure of **5** indicates that a second species must be formed. Other species were observed in the ^1^H and ^7^Li NMR spectra of the reaction mixture but could not be isolated. The origin of the imido- proton was further confirmed by observing the shift in the N-*H*/*D* IR stretch from 3,313 cm^−1^ (for **5**) to 2,452 cm^−1^ (for **5-D**_**2**_) upon isotopic substitution (Supplementary Fig. 127). The formation of **5** could be the result of H^+^/Li^+^ scrambling from a putative bis-imido complex formed upon cleavage of the N–N bond followed by rearrangement and formation of **3** and an imido–amido complex ([Li{(Tren^DMBS^)U^IV^}_2_(*μ*-NH_2_)(*μ*-NH)]).

Exposure of a frozen solution of **4** in toluene-*d*_8_ to 1.01-bar H_2_ and subsequent warming to room temperature resulted in an immediate colour change from dark-brown to brown. The ^1^H NMR and ^7^Li NMR spectra of the reaction mixture indicate that multiple species are formed, but only complex **5** could be isolated and crystallographically characterized. Similarly to the D_2_ reactivity of **3**, exposure of **4** to deuterium gas gives a full set of resonances assigned to **5**, with only the N*H* resonance absent, as the sole identifiable species (Supplementary Figs. 34 and 35). The formation of **5** is probably the result of heterolytic splitting of H_2_ by the bis-nitride complex, accompanied by elimination of LiH, but the latter could not be unambiguously identified. Germane to this point, heterolytic splitting of H_2_ by a nitride bridged U(IV) complexes to give a rare imide–hydride complex, with the hydride bridging two U(IV) and a Cs cation, has been reported^53^. Here, the high affinity of the hydride for the Li-cation would account for elimination of the putative LiH.

### Computational analysis

To provide further insight into the U–N_2_ complexes reported here, we conducted density functional theory (DFT) calculations on **2-crypt**, **2-Li**, **2-K** and **3**. Additionally, although **1** has already been extensively examined by DFT^79^, we computed **1** at the same level of theory to provide a meaningful benchmark. The closely related analogue of **4**, [{U^IV^(*μ-*NLi_2_)(Tren^TIPS^)}_2_], has been computationally analysed in detail previously^80^, so we did not compute **4** here. Given the Li-cation disorder in **3**, we computed two isomers with respect to the positions of the Li cations: the side-on/end-on isomer **3A** and the end-on/end-on isomer **3B**. We find that **3A**, in the gas phase, is more stable than **3B** by 3.37 kcal mol^−1^, consistent with the experimental observation of crystallographic disorder.

For spin-quintet **1**, there are four α-spin electrons, where HOMO (highest occupied molecular orbital) to HOMO−2 are of essentially pure 5*f* character, and HOMO−3 is mixed 5*f*/N_2_-π* (46/45%, the B(π_g⊥_) δ-symmetry orbital). HOMO−4 (and its β-spin counterpart) constitute the A(π_g=_) in-plane bonding combination (Supplementary Fig. 151). The addition of an extra electron to give **2-crypt**, **2-Li** and **2-K** in each case introduces an extra α-spin 5*f* electron, producing spin-sextet formulations. Hence for **2-crypt**, **2-Li** and **2-K**, in each case HOMO to HOMO−3 are 5*f*-character, and HOMO−4 is the 5*f*/N_2_-π* B(π_g⊥_) (average 33/57%) (Supplementary Figs. 152–154). HOMO−5 and its β-spin counterpart are the A(π_g=_) combination. On moving to spin-quintet **3A**/**3B**, again, four α-spin electrons of 5*f*-character are found in HOMO to HOMO−3, and HOMO−4 and HOMO−5 and their β-spin counterparts are the B(π_g⊥_) and A(π_g=_) orbitals, confirming full occupation of the π*-manifold of the N_2_ unit (Supplementary Figs. 155 and 156).

The computed uranium MDC_q_ charges (1.90–2.42; MDC_q_ is the multipole derived charge, where q denotes quadrupolar) and MDC_m_ (MDC_m_ is the multipole derived charge, where m denotes multipole) net spin densities (2.09–2.53) for **1**, **2-crypt**, **2-Li**, **2-K** and **3** reflect the formal presence of U(IV) ions in all complexes, consistent with the characterization data (Supplementary Table 4). The analogous MDC_q_ data for the N_2_ units progressively increase from −1.88 to −2.73, reflecting the increasing formal N_2_ charges across the series. This is also reflected in the MDC_m_ data, where the net spin density of 0.25 electrons on the N_2_^2−^ unit in **1** increases to 0.36 (average) for the N_2_^3−^ radical group in **2-crypt**, **2-Li** and **2-K**, but becomes a net spin density deficiency in **3** (−0.3), reflecting the strongly donating capacity of N_2_^4−^. Notably, across the series, the N–N bond order progressively weakens, from 1.66 in **1** to 1.27 in **3A**, and this is reflected by quantum theory of atoms in molecules (QTAIM) topological bond analysis and in analytical frequency calculations, where clear N–N stretches are computed at 1,207 cm^−1^ (**1**), 1,180 cm^−1^ (**2-K**), 1,139 cm^−1^ (**2-crypt**) and 990/915 cm^−1^ (**3B**/**3A**).

### Stoichiometric and catalytic conversion of N_2_ to NH_3_

To provide a baseline for catalytic studies, we first studied the stoichiometric reactions of **1**, **2-crypt**, **3** and **4** with H^+^, because the yield of the resulting NH_3_, isolated as the conjugate acid NH_4_Cl, could indicate the degree of N_2_ activation, with higher yield associated with greater activation (Table 1, entries 1–4). A previous control experiment where [Ti(Tren^TMS^)Cl] (Tren^TMS^ = {N(CH_2_CH_2_NSiMe_3_)_3_}^3−^) was treated with 1 M HCl only produced 0.04 NH_3_ equivalents, suggesting only minor degradation of Tren^R^ ligands under the action of strong acids. This was confirmed by treating [U^IV^(Tren^DMBS^)Cl] with 2 M ethereal HCl, which produced 0.03 NH_3_ equivalents. Treatment of crystalline **1** with excess 2 M HCl affords 0.18 NH_3_ equivalents, indicating weak activation of the pernitride unit (Supplementary Fig. 36). Analogously, crystalline **2-crypt** produced 0.43 NH_3_ equivalents, suggesting stronger activation than **1** (Supplementary Fig. 37). This is more NH_3_ equivalents than found for [K(2.2.2-crypt)][{(ArO)_3_U^IV^}_2_(*μ-η*^2^:*η*^2^-N_2_)] (0.34 equiv.), noting that no NH_3_ was detected from HCl treatment of [{(ArO)_3_U^IV^}_2_(*μ-η*^2^:*η*^2^-N_2_)]^26^. For HCl treatment of hydrazido(4−)-bridged **3**, 1.57 NH_3_ equivalents were detected, indicating much stronger activation of the N–N bond compared to **1** and **2-crypt** (Supplementary Fig. 38). For comparison, 0.54–1.08 NH_3_ equivalents were detected upon direct protonation of the dihydrohydrazido(2−) complex K_4_[U^IV^_2_(*μ-*N_2_H_2_)(mTP^R−^)_2_] (mTP^R−^ = [{2-(OC_6_H_2_-^*t*^Bu-2,R-4)_2_CH}-C_6_H_4_-1,3]^4−^; R = Me, ^*t*^Bu)^24,81^ with [HPy]Cl, and [K_3_{[U^V^(OSi(O^*t*^Bu)_3_)_3_]_2_(*μ-*N)(*μ-η*^2^:*η*^2^-N_2_)}]^23^ gave 0.5–0.84 NH_3_ equivalents after addition of excess ethereal HCl, and 1.54 NH_3_ equivalents when the same compound was first exposed to 1.01-bar H_2_ gas before HCl. Finally, crystalline bis-nitride **4** quantitatively produced 2.0 equiv. of NH_3_ when treated with HCl, as previously found for other U(IV/V/VI)–nitride complexes (Supplementary Fig. 39)^34,54,55,61,82^. These reactions confirm the anticipated range of N–N activation, consistent with the experimental and computational characterization data of **1**, **2-crypt**, **3** and **4**.

**Table 1:** Stoichiometric and catalytic acidification experiments for the reaction of crystalline **1**, **2-crypt**, **3** and **4** with 2 M ethereal HCl and solutions of **1** with acids and reductants under N_2_ to produce NH_3_ and N_2_H_4_

Entry^a^	Compound	Solvent	Acid	Reductant	Acid (equiv.)	Reductant (equiv.)	NH_3_ (equiv.)	N_2_H_4_ (equiv.)	Fixed-N (equiv.)
1	**1**	Et_2_O	2M HCl	−	>1,500	−	0.18	−	0.18
2	**2-crypt**	Et_2_O	2M HCl	−	>1,500	−	0.43	−	0.43
3	**3**	Et_2_O	2M HCl	−	>1,500	−	1.57	−	1.57
4	**4**	Et_2_O	2M HCl	−	>1,500	−	2.00	−	2.00
5	−	Et_2_O	[Cy_3_PH][I]	KC_8_	600	600	0.00	0.03	0.06
6	−	Et_2_O	[Cy_3_PH][I]	KC_8_	600	600	0.00^b^	0.03^b^	0.06^b^
7	**1**	Hexane	[Cy_3_PH][I]	KC_8_	10	4	0.70	0.00	0.70
8	**1**	Toluene	[Cy_3_PH][I]	KC_8_	10	4	0.07	0.00	0.07
9	**1**	Et_2_O	[Cy_3_PH][I]	KC_8_	10	4	1.18	0.00	1.18
10	**1**	Et_2_O	[Et_3_NH][BPh_4_]	KC_8_	600	600	0.00	0.00	0.00
11	**1**	Et_2_O	[Et_3_NH][Cl]	KC_8_	600	600	0.00	0.08	0.15
12	**1**	Et_2_O	[Et_3_NH][I]	KC_8_	600	600	0.00	0.01	0.02
13	**1**	Et_2_O	[Cy_3_PH][BAr^F20^]	KC_8_	129	129	0.40	−	0.40
14	**1**	Et_2_O	[Cy_3_PH][I]	Na	600	600	0.00	0.13	0.26
15	**1**	Et_2_O	[Cy_3_PH][I]	K	600	600	0.00	0.12	0.24
16	**1**	Et_2_O	[Cy_3_PH][I]	KC_8_	173	173	1.74	0.46	2.66
17	**1**	Et_2_O	[Cy_3_PH][I]	KC_8_	173	173	0.34	0.12	0.58
18	**1**	Et_2_O	[Cy_3_PH][I]	KC_8_	300	300	0.88	0.13	1.14
19	**1**	Et_2_O	[Cy_3_PH][I]	KC_8_	600	600	0.62	0.05	0.72
20	**1**	Et_2_O	[Cy_3_PH][I]	Rb	600	600	1.11	0.07	1.25
21	**1**/**1-**^**15**^**N**_**2**_	Et_2_O	[Cy_3_PH][I]	RbC_8_	600	600	8.84/8.17^c^	0.00^c^	8.84/8.17^c^
22	**1**/**1-**^**15**^**N**_**2**_	Et_2_O	[Cy_3_PH][I]	RbC_8_	600	600	1.49/1.12^d^	0.05/0.11^d^	1.59/1.34^d^
23	**1**	Et_2_O	[Cy_3_PH][I]	Cs	600	600	4.94	0.06	5.06
24	**1**	Et_2_O	[Cy_3_PH][I]	CsC_8_	600	600	2.61	0.03	2.67
25	**4**	Et_2_O	[Cy_3_PH][I]	RbC_8_	600	600	4.43	0.00	4.43

All reactions were conducted twice to ensure reproducibility. Solutions of **1** were as follows: 1.3 mM for entries 5–7, 11, 14–16; 0.65 mM for entries 8–10, 12, 13, 17–25. Entries 1, 2, 3 and 4 were conducted with no reductant present. Entries 5 and 6 were conducted with no U-based catalyst present.

^a^Experiments were performed under 1.31-bar N_2_ (except for entry 17 (2.21 bar) and entry 22 (~1.0 bar)) at −78 °C, followed by warming to 25 °C and then stirring for 17 h (MC_8_, M = K, Rb, Cs) or 72 h (M^0^, M = Na, K, Rb, Cs).

^b^Conducted under ^15^N_2_.

^c^Conducted with **1-**^**15**^**N**_**2**_ and under ^15^N_2_ (1.31 bar).

^d^Conducted with **1-**^**15**^**N**_**2**_ and under ^15^N_2_ (~1.01 bar).

We next undertook catalytic studies, and, based on the recently reported titanium-catalysed conversion of N_2_ to NH_3_ (ref. ^14^), we examined the reactivity of **1** with the weak acid source [Cy_3_PH][I] (Cy = cyclohexyl) and elemental alkali metals (Na, K, Rb, Cs) or MC_8_ alkali-graphite (M = K, Rb, Cs) reagents (Table 1). These combinations benefit from the low solubility of both the H^+^ and e^−^ sources, minimizing their sacrifical reaction with each other rather than sequential reactivity with U_2_–N_2_ species (Supplementary Figs. 40–73).

To provide a firm basis on which to undertake catalytic studies, we established several experimental baselines. The absence of **1** is decisive, with only 0.03 equiv. of N_2_H_4_ formed when [Cy_3_PH][I] and KC_8_ are mixed together then worked up (Table 1, entries 5 and 6). To examine the potential effect of solvent (Table 1, entries 7–9), we tested the reactivity of ~1.3 mM solutions **1** with 4 equiv. of KC_8_ and 10 equiv. of [Cy_3_PH][I] and found up to 1.18 NH_3_ equivalents, with yields in the order diethyl ether > hexane > toluene. We thus focused on using diethyl ether for further reactions. The weakly acidic nature of [Cy_3_PH][I] minimizes protonation of the tren-ligand. This is underscored by the fact that attempts to obtain catalytic conversion of N_2_ to NH_3_ using **1**, [Et_3_NH][X] (X = BPh_4_^−^, Cl^−^ or I^−^) and KC_8_ did not result in any NH_3_ being detected (Table 1, entries 10–12), suggesting that the [Et_3_NH][BPh_4_] is too aggressive a H^+^ source, leading to protonation of the Tren^DMBS^ ligand (Supplementary Fig. 74). The importance of a halide source to the catalysis, to produce [U(Tren^DMBS^)I] (after fixed-N is eliminated) that can be reduced to regenerate **1**, is demonstrated by the failure to generate NH_3_ with [Et_3_NH][BPh_4_], and the use of [Cy_3_PH][BAr^F20^] also did not sustain any catalysis (Table 1, entries 10 and 13), with only 0.4 NH_3_ equivalents detected (Supplementary Figs. 45–48). Finally, although the catalytic conditions in this study are quite different to those of electrochemical scenarios, to benchmark potential NO_*x*_-to-NH_3_ conversion false positives, we conducted control experiments^83,84^ with 1 equiv. of nitrite or nitrate present (Supplementary Table 3), and found that only up to 0.53 equiv. of ammonia are produced. Given that the catalysis described here is in a sealed rather than continuous-flow scenario, with scrupulously cleaned glassware, and that NO_*x*_ impurities in N_2_ gases are at low levels, NO_*x*_-to-NH_3_ conversion false positives can thus be ruled out. Having ascertained the optimal solvent and H^+^ source, we examined varying the reductant.

**Fig. 4:**
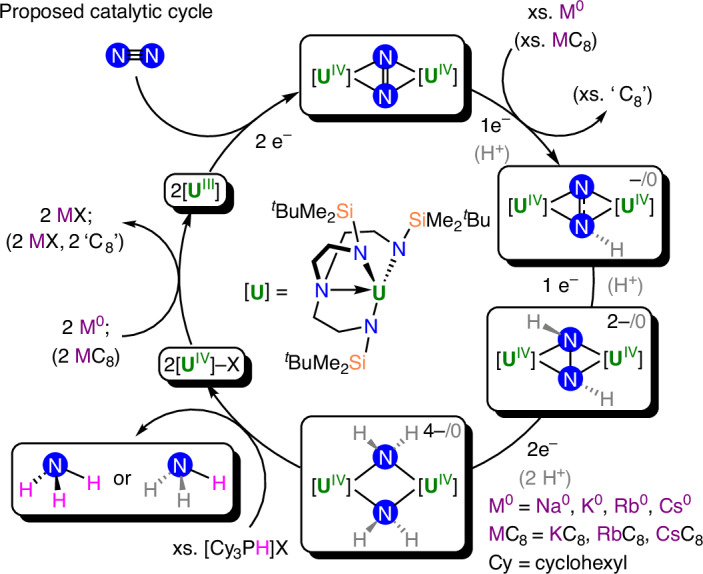
Proposed catalytic cycles for the ^DMBS^Tren uranium–N_2_ system. Two possible mechanisms are shown for the conversion of N_2_ to NH_3_ by **1**: (1) sequential reduction with protonation only occurring at the nitride step; (2) proton-coupled sequential reduction (grey H atoms).

Reaction of 0.65 mM solutions of **1** with 600 equiv. of Na and [Cy_3_PH][I] under 1.31-bar N_2_ produced no NH_3_ equivalents and only a small quantity of N_2_H_4_ (0.13 equiv.), with a maximum of 0.26 equiv. of fixed-N (Table 1, entry 14). Equivalent reactions with K and Rb under 1.31-bar N_2_ were similarly unproductive (Table 1, entries 15 and 20), and we attribute the poor performance of Na, K and Rb to kinetic factors. In contrast, however, the equivalent reaction with Cs (Table 1, entry 23) produced 4.94 equiv. of NH_3_ along with a trace amount of N_2_H_4_. We rationalize this on the basis that during the catalytic runs with Cs, a small amount of Cs would be liquid due to the low melting point of Cs (28.5 °C), and this could thus facilitate the reaction kinetics. Noting that **1** exhibited 2.5 turnovers with Cs, we turned our attention to MC_8_ reagents using 600 equiv. of MC_8_/[Cy_3_PH][I] (Table 1, entries 16–19, 21, 22, 24 and 25). Starting with KC_8_, we find that reactions conducted with 173, 300 and 600 equiv. of KC_8_/[Cy_3_PH][I] produced 1.74, 0.88 and 0.62 equiv. of NH_3_, respectively, along with modest amounts of N_2_H_4_ (0.12–0.46 equiv.). On first inspection these observations are counterintuitive, but they probably reflect the complexity of a number of finely balanced factors critical to successful catalytic turnover. In contrast to Cs, CsC_8_ produced 2.61 equiv. of NH_3_ with trace N_2_H_4_. However, importantly, RbC_8_ reproducibly produced 8.84 equiv. of NH_3_ and no N_2_H_4_, representing 4.4 catalytic turnovers (Supplementary Fig. 56). To confirm the source of the fixed-N over and above the baseline acidifications, we repeated the RbC_8_ reaction with **1-**^**15**^**N**_**2**_ and ^15^N_2_, and found that 8.17 equiv. of NH_3_ are formed, which is consistent with the ^14^N_2_ data (Supplementary Fig. 57). When the same catalytic reaction (600 equiv. RbC_8_/600[Cy_3_PH][I]) was conducted with nitride complex **4**, 4.4 equiv. of NH_3_ were formed, suggesting that a nitride species could be involved in the catalytic pathway, but the presence of strongly bound Li cations in **4** may hinder the catalytic efficiency (Supplementary Fig. 73; Table 1, entry 25). This work thus establishes catalytic N_2_ fixation to NH_3_, albeit modest, with a side-on bound complex of N_2_, and, when taken together with the stoichiometric sequential reductions, we propose two possible catalytic cycles (Fig. 4): (1) sequential reduction, with protonation only occurring at the nitride step, or (2) proton-coupled sequential reduction. Notably, the addition of 600 equiv. of weak acid ([Cy_3_PH][I]) to the N_2_^3−^, N_2_^4−^ and N^3−^ species in the absence of reducing agent results in ammonia formation only for the nitride complex. However, when the addition of acid is carried out in the presence of 600 equiv. RbC_8_ under Ar we observed the formation of ammonia for the three complexes, albeit to a different extent (1.3 equiv. for **2-crypt**, 0.9 equiv. for hydrazido **3** and 2.0 equiv. for nitride **4**). This suggests that protonation at earlier stages could also play a role in the catalytic cycle (Supplementary Figs. 70–72 and Supplementary Table 3, entries 33–35). Finally, given prior work on Mo–triamidoamine complexes, it may well be that the triamidoamine amide centres shuttle incoming protons to the reduced dinitrogen moiety^85,86^; such mechanistic detail will require further mechanistic study to elaborate.

## Conclusion

To conclude, we have reported the stepwise reduction of N_2_ from free-N_2_ to bridging side-on bound forms and subsequently to bridging nitrides, in the process accessing four different states of side-on bound N_2_ for the same dinuclear U-complex. Our combined experimental and quantum-chemical calculation characterization data provide a consistent description of the U-complexes in this study and show that although the coordinated N_2_ undergoes sequential reduction steps, the U ions largely remain as U(IV) throughout. Although the catalytic turnover is currently modest, we have established molecular U-mediated catalytic N_2_ to NH_3_ conversion and moreover demonstrated N_2_ to NH_3_ catalysis with side-on bound coordination of N_2_, adding this mode of reactivity to the paradigm of catalytic conversion of N_2_ to NH_3_ by terminal or bridging end-on metal–N_2_ complexes. Our stoichiometric reactions suggest the relevance of N_2_^2−^, N_2_^3−^, N_2_^4−^ and N^3−^ in the catalytic conversion of N_2_ to NH_3_ when involving side-on bridging N_2_. The recognition that side-on bound N_2_ can be catalytically activated and converted into NH_3_ through polymetallic cooperativity provides conceptual homogeneous–heterogeneous links to, for example, Haber–Bosch processes, and offers new vistas for the elaboration of molecular catalytic transformations involving N_2_ and probably other small molecules.

## Methods

### General considerations

All manipulations were carried out under an inert dinitrogen atmosphere using a standard Schlenk line or glovebox techniques. Catalytic trials were carried out using a J. Young sealable two-bulb apparatus (Supplementary Fig. 1). Water and oxygen levels were kept below 0.1 ppm at all times. Glass-coated stir bars were used instead of Teflon-coated ones to prevent unwanted polytetrafluoroethylene (PTFE) reactivity during reduction reactions. All solvents and reagents were rigorously dried and deoxygenated before use. Prepared compounds were characterized by elemental analyses, NMR, infrared (IR) and EPR spectroscopy, superconducting quantum interference device (SQUID) magnetometry, single-crystal X-ray diffraction studies and DFT, natural bond orbital (NBO) and bond topology theoretical calculations. Further details on the synthetic preparations as well as additional characterization for all compounds are provided in Supplementary Information.

### Synthesis of [{U^IV^(Tren^DMBS^)}_2_(*μ*-*η*^2^:*η*^2^-N_2_)] (1), modified procedure

Under an atmosphere of Ar (ref. ^35^), a pale-green solution of [U^IV^(Tren^DMBS^)Cl] (374.2 mg, 0.493 mmol, 1 equiv.) in *n*-hexane (10 ml) was added to a Schlenk tube containing a glass-coated stir bar. Solid potassium graphite (199.8 mg, 1.478 mmol, 3 equiv.) was then added in portions, which resulted in the formation of a dark-purple reaction mixture that was allowed to stir for 24 h at room temperature. Full conversion to the [U^III^(Tren^DMBS^)] species was confirmed by ^1^H NMR spectroscopy. The resulting reaction mixture was filtered on a porosity ‘4’ glass frit, and the solid residue was rinsed with *n*-hexane (3 × 0.5 ml), yielding a dark-purple solution. The volatiles were removed in vacuo, and the resulting purple solid was brought into the dinitrogen glovebox (1.016-bar absolute N_2_ pressure) and dissolved in *n*-pentane (2 ml) to immediately form a dark-red solution. The solution was then stored at −40 °C for 2 days to yield dark-red crystals of **1** in 48% yield (178.1 mg, 0.118 mmol) that were dried under N_2_ flow. The mother liquor was concentrated to 1 ml, and then stored at −40 °C, yielding additional **1** (48.2 mg, 0.032 mg) after 2 days. The procedure was repeated one more time (concentration to 0.5 ml and storage at −40 °C), yielding a third and final crop of **1** after 2 days (0.024 mmol, 37.1 mg). The total yield was 71% (263.4 mg). It was found that the use of *n*-hexane instead of pentane for the crystallization step resulted in lower overall yield. Elemental analysis: Calcd. for C_48_H_114_N_10_Si_6_U_2_ (**1**): C, 39.06; H, 7.78; N, 9.49. Found: C, 39.22; H, 7.79; N, 9.30. ^1^H NMR (400 MHz, toluene-*d*_8_, 298 K): δ 10.95 (s, 12H, C*H*_2_), 7.21 (s, 12H, C*H*_2_), 3.84 (s, 54H, C(C*H*_3_)_3_), −20.24 ppm (s, 36H, Si(C*H*_3_)_2_) (Supplementary Fig. 9). The preparation of the ^15^N_2_-labelled isotopologue, **1-**^**15**^**N**_**2**_, is described in Supplementary Information.

When the synthesis of **1** was performed under 2.21-bar N_2_, analytically clean **1** could be isolated in a better yield (compared to when performed under 1 atm (1.01 bar)) from a single crystallization. Once isolated, complex **1** is stable in the solid state under N_2_, but quickly releases N_2_ under dynamic vacuum. Complex **1** (~8.9 mM concentration) in toluene-*d*_8_ and cyclohexane-*d*_12_ at 1.01-bar N_2_ exist in equilibrium with [U^III^(Tren^DMBS^)] in an approximate 2:1 ratio (^1^H NMR, Supplementary Fig. 9). No increase in N_2_ uptake was observed by ^1^H NMR spectroscopy when lowering the solution temperatures to −80 °C. However, increasing the dinitrogen pressure to 2.51 bar leads to a further shift in **1**:[U^III^(Tren^DMBS^)] equilibrium, from 2:1 to 5:1 (Supplementary Fig. 12) at room temperature. In contrast, the ^1^H NMR spectrum of the isolated crystalline **1** in THF-*d*_8_ showed predominantly [U^III^(Tren^DMBS^)] resonances, indicating that N_2_ loss occurs in the presence of coordinating solvents (Supplementary Fig. 10). This was further corroborated by the ^15^N NMR spectroscopy of **1-**^**15**^**N**_**2**_, where only the resonance for free ^15^N_2_ could be observed (Supplementary Fig. 14), with the absence of a resonance for **1-**^**15**^**N**_**2**_ being attributed to its paramagnetism.

### Synthesis of [K(2.2.2-cryptand)][{U^IV^(Tren^DMBS^)}_2_(*μ*-*η*^2^:*η*^2^-N_2_)] (2-crypt)

Under an atmosphere of dinitrogen, a solution of **1** (130.1 mg, 0.086 mmol, 1 equiv.) and 2.2.2-cryptand (32.5 mg, 0.086 mmol, 1 equiv.) in toluene (4 ml) was added to a Schlenk tube containing a glass-coated stir bar. Solid potassium graphite (11.7 mg, 0.086 mmol, 1 equiv.) was then added at room temperature, and the reaction was left stirring for 3 h to yield a dark-brown mixture. The volatiles were then removed in vacuo, and soluble residues were extracted into THF (4 ml) to facilitate graphite removal by filtration on a porosity ‘4’ glass frit. Volatiles were then removed in vacuo from the filtrate, the residue dried for 1 h, and toluene (~2 ml) added. Storage of the brown solution at −40 °C for 2 days yielded **2-crypt**·2C_7_H_8_O as dark-brown crystals in 83% yield (135.6 mg, 0.072 mmol). **2-crypt** is readily soluble in THF and only modestly in toluene; the complex was found to be stable towards nitrogen loss in THF and toluene (no change in the ^1^H NMR spectra after dissolution or a freeze–pump–thaw cycle). Elemental analysis: Calcd. for C_69.5_H_154_KN_12_O_6_Si_6_U_2_ (**2-crypt** · 0.5C_7_H_8_O): C, 43.08; H, 8.01; N, 8.67. Found: C, 43.04; H, 8.05; N, 7.93. ^1^H NMR (400 MHz, 298 K, THF-*d*_8_): δ 86.21 (s, 12H, C*H*_2_), 25.89 (s, 12H, C*H*_2_), 3.06–3.03 (m, 24H, 2.2.2-crypt), 2.04 (m, 12H, crypt), −13.02 (s, 54H, C(C*H*_3_)_3_), −33.64 (s, 36H, Si(C*H*_3_)_2_) (Supplementary Fig. 15a). ^29^Si{^1^H} NMR (79.5 MHz, 298 K, THF-*d*_8_): δ −181.81 (Supplementary Fig. 15b).

### Synthesis of [K{U^IV^(Tren^DMBS^)}_2_(*μ*-*η*^2^:*η*^2^-N_2_)] (2-K)

Under an atmosphere of dinitrogen, solid potassium graphite (0.217 mmol, 29.3 mg, 3 equiv.), pre-chilled to −40 °C, was added to a cold solution of **1** (108.7 mg, 0.072 mmol, 1 equiv.) in *n-*hexane. The mixture was left to stir for 3 days at −40 °C, then filtered on a porosity ‘4’ glass frit, also pre-chilled to −40 °C, and concentrated to 0.5 ml while cold. Dark-brown/green crystals of **2-K** were obtained from *n-*hexane after 2 days at −40 °C, in 62% yield (69.1 mg, 0.045 mmol). Elemental analysis: Calcd. for C_51_H_121_KN_10_Si_6_U_2_ (**2-K**·0.5C_6_H_14_): C, 39.31; H, 7.83; N, 8.99. Found: C, 39.17; H, 7.78; N, 8.63. ^1^H NMR (400 MHz, 233 K, toluene-*d*_8_): δ 5.88 (s, br.), −21.20 (s, very br.), 83.58 (s, very br.) (Supplementary Fig. 16a). (193 K): −12.46 (s, very br.), −27.79 (s, very br.), −36.57 (s, very br.), −132.49 (s, very br.), −150.65 (s, very br.) (Supplementary Fig. 16b).

### Synthesis of [Li_2_{U^IV^(Tren^DMBS^)}_2_(*μ*-*η*^2^:*η*^2^-N_2_)] (3)

Under an atmosphere of dinitrogen, crystalline **1** (130.4 mg, 0.088 mmol, 1 equiv.) was combined with solid lithium iodide (23.7 mg, 0.177 mmol, 2 equiv.). At room temperature, diethyl ether (2 ml) was added, and the mixture was stirred until fully dissolved, before being stored at −40 °C for 1 h. The resulting cold mixture was added to cold (−40 °C) solid potassium graphite (23.9 mg, 0.177 mmol, 2 equiv.) in cold diethyl ether (1 ml) and stirred for 3 days at −40 °C before filtration on a porosity ‘4’ glass frit pre-chilled to −40 °C to obtain a dark-brown/red solution. Concentration in vacuo to ~1 ml and storage at −40 °C for 4 days yielded dark-red/brown crystals of **3** in 63% yield, obtained in several crops (82.9 mg, 0.056 mmol). Elemental analysis: Calcd. for C_52_H_124_Li_2_N_10_OSi_6_U_2_ (**3** ·1C_4_H_10_O): C, 39.93; H, 7.99; N, 8.96. Found: C, 39.56; H, 7.79; N, 8.65. ^1^H NMR (400 MHz, 298 K, toluene-*d*_8_): the spectrum shows paramagnetically shifted resonances spanning a wide range from −130 to 200 ppm, indicating the presence of a highly asymmetric structure in solution (Supplementary Figs. 18 and 19).

### Synthesis of [Li_4_(OEt_2_){U^IV^(Tren^DMBS^)}_2_(*μ*-N)_2_] (4)

Under an atmosphere of dinitrogen, crystalline **1** (107.9 mg, 0.073 mmol, 1 equiv.) was combined with solid lithium iodide (58.8 mg, 0.439 mmol, 6 equiv.). At room temperature, diethyl ether (3 ml) was added and the mixture stirred until fully dissolved, before being stored at −40 °C for 1 h. The resulting mixture was added to cold solid potassium graphite (59.3 mg, 0.439 mmol, 6 equiv.) in diethyl ether (1 ml) and allowed to stir for 3 days at −40 °C before filtration on a cold porosity ‘4’ glass frit to obtain a dark-brown solution. Concentration in vacuo to ~1 ml and storage at −40 °C for 3 days yielded dark-brown crystals of **4**·1C_4_H_10_O in 56% yield (61.6 mg, 0.056 mmol). Elemental analysis: Calcd. for C_52_H_124_Li_4_N_10_OSi_6_U_2_ (**4**): C, 39.58; H, 7.92; N, 8.88. Found: C, 39.48; H, 7.89; N, 8.89. ^1^H NMR (400 MHz, 298 K, toluene-*d*_8_): the spectrum shows paramagnetically shifted resonances spanning a wide range from −135 to 150 ppm, indicating the inequivalence of many of the 114 protons present in the complex (Supplementary Figs. 22 and 23).

### Synthesis of [Li_3_{U^IV^(Tren^DMBS^)}_2_(*μ*-N)(*μ*-NH)] (5)

A dark-red/brown solution of **3** (37.1 mg, 0.025 mmol, 1 equiv.) in toluene (0.5 ml) was transferred to a J. Young valve-capped tube and connected to a Schlenk line. The resulting solution was degassed by three cycles of freeze–pump–thawing, and hydrogen gas (1.01 bar) was added to the reaction mixture and the tube warmed to room temperature. A gradual colour change to brown was observed over the course of 2 days as full consumption of the starting material occurred. Brown crystals of **5** were collected from the reaction mixture after leaving it to stand at room temperature (7.9 mg) for 3 days. An additional crop (4.4 mg) of crystalline **5** was isolated after 1 day upon placing the reaction mixture at −40 °C. Elemental analysis: Calcd. for C_48_H_115_Li_3_N_10_Si_6_U_2_ (**5**): C, 38.49; H, 7.74; N, 9.35. Found: C, 38.97; H, 7.75; N, 9.32. ^1^H NMR (400 MHz, 298 K, toluene-*d*_8_): the spectrum shows paramagnetically shifted resonances spanning a wide range from −170 to 170 ppm, including the NH resonance identified at −170.25 ppm (s, 1H, N*H*) (Supplementary Figs. 29 and 30). IR (KBr pellet, *ν*/cm^−1^): 3,313 (w, N–H), 2,953 (s), 2,929 (s), 2,883 (s), 2,852 (s), 2,735 (w), 2,705 (w), 2,672 (w), 1,605 (m), 1,468 (m), 1,405 (w), 1,388 (w), 1,357 (w), 1,246 (m), 1,133(w), 1,113 (w), 1,092 (m), 1,072 (m), 1,057 (m), 1,023 (m), 1,011 (w), 967 (m), 925 (s), 903 (m), 888 (s), 825 (s), 777 (s), 717 (m), 655 (s), 586 (m), 560 (m), 527 (m), 505 (m) (Supplementary Fig. 127).

A second species observed in the ^1^H NMR spectrum (Supplementary Figs. 26 and 27) of the mother liquor measured in toluene-*d*_8_ could not be isolated despite repeated attempts.

### Synthesis of [Li_3_{U^IV^(Tren^DMBS^)}_2_(*μ*-N)(*μ*-ND)] (5-D_2_) through the reaction of 3 with D_2_ gas

A dark-red/brown solution of crystalline **3** (37.1 mg, 0.025 mmol, 1 equiv.) in toluene (0.3 ml) was transferred to a J. Young valve-capped NMR tube and connected to a Schlenk line. The resulting solution was degassed by three cycles of freeze–pump–thawing and deuterium gas (0.5 bar absolute pressure of D_2_) was added to the reaction mixture and the tube was warmed to room temperature. An immediate colour change from dark-red/brown to brown was observed. After 1 h, all volatiles were removed in vacuo and toluene-*d*_8_ (0.3 ml) was added. Dark-brown crystals of **5-D**_**2**_ (6.6 mg) were isolated from the reaction mixture after 2 days at −40 °C. ^1^H NMR (400 MHz, 298 K, toluene-*d*_8_): the spectrum of the reaction mixture as well as the one of isolated crystalline **5-D**_**2**_ showed the full set of resonances assigned to **5** (Supplementary Figs. 31–33), except for the broadened N*H* singlet at −170.25 ppm (Supplementary Fig. 32), which is not present in the deuterated mixture. IR (KBr pellet, *ν*/cm^−1^): 2,953 (s), 2,928 (s), 2,883 (s), 2,851 (s), 2,704 (w), 2,452 (w, N–D), 1,468 (m), 1,444 (w), 1,387 (w), 1,357 (w), 1,288 (m), 1,133 (w), 1,111 (w), 1,091 (m), 1,056 (m), 1,024 (m), 968 (m), 924 (s), 902 (m), 889 (s), 825 (s), 779 (s), 717 (m), 654 (s), 584 (m), 526 (w), 505 (w), 484 (w) (Supplementary Fig. 127).

### Reaction of 1, 2-crypt, 3 and 4 with excess HCl

To J. Young valve-capped NMR tubes containing solid complexes **1** to **4**, a solution of HCl (2 M, 2.0 ml in total) in diethyl ether was added at −80 °C in a glovebox-fitted cold well. On warming each tube to −40 °C and then subsequently to room temperature, discolouration of the solids occurred, and formation of light-yellow solutions was observed. After 1 h, all volatiles were removed in vacuo and the resulting solids were dissolved in dimethylsulfoxide (DMSO)-*d*_6_ (0.5 ml). 2,5-Dimethylfuran was added to the tubes directly or in the form of an NMR insert (sealed quartz capillary) as an internal standard for the quantitative detection of NH_4_Cl. Formation of 0.18 equiv. of NH_4_Cl for **1**, 0.43 equiv. for **2-crypt**, 1.57 equiv. for **3** and 2.0 equiv. for **4** were detected by quantitative ^1^H NMR experiments (Supplementary Figs. 36–39). Under similar experimental conditions, the parent monomeric complex [U^IV^(Tren^DMBS^)Cl] produced 0.03 equiv. of NH_4_Cl.

### Acidification of [{U^IV^(Tren^DMBS^)}_2_(*μ*-*η*^2^:*η*^2^-N_2_)] under a ^14^N_2_ atmosphere

Under an atmosphere of Ar, bulb A was charged with **1** (0.0052 mmol), the acid and reductant, along with a glass-coated stirrer bar. Bulb A was then sealed and transferred to a Schlenk line. Et_2_O (stored under Ar, 4–8 ml, vide infra) was added to bulb B via syringe, and then bulb B was submerged in a LN_2_ dewar (−196 °C) and frozen. The entire apparatus was placed under a dynamic vacuum (1 × 10^−3^ mbar) and sealed, leaving the apparatus under static vacuum. The LN_2_ dewar (−196 °C) was removed from bulb B and immediately transferred to bulb A such that the frozen solvent in bulb B could thaw and distil onto the frozen solids in bulb A. After the distillation had concluded, the apparatus was placed under a dinitrogen atmosphere, sealed, and allowed to warm to room temperature. For reactions involving MC_8_ (M = K, Rb, Cs) as the reductant, the reaction mixture was stirred for 17 h. In the case of the alkali metals, the reaction mixtures were subsequently sonicated until the metal was finely dispersed (1–40 min) and the reactions were stirred for 72 h. After this time, HCl (2 M in Et_2_O, 2 ml) was added to bulb B via syringe and both bulbs were subsequently submerged into separate LN_2_ (−196 °C) dewars and frozen. The entire distillation apparatus was then placed under a dynamic vacuum and sealed, leaving the vacuum under static vacuum, and the LN_2_ dewar removed from bulb A. During this process, the reaction mixture in bulb A thaws, and the volatiles were distilled onto the frozen HCl in bulb B. Once the acid distillation was complete (~30 min), bulb A was resubmerged in a LN_2_ (−196 °C) dewar and the contents frozen. Bulb B was sealed, and aqueous KOH (30%, 4 ml) was added via syringe to bulb A under a flow of dinitrogen. Once the contents had frozen, the entire apparatus was once again placed under a dynamic vacuum and sealed, leaving the apparatus under static vacuum, and the LN_2_ (−196 °C) dewar removed from bulb A. The base distillation was performed with vigorous stirring for 1 h, then both bulbs A and B were sealed, and bulb B was allowed to warm to room temperature and stirred for 10 min. All volatiles in bulb B were subsequently removed in vacuo and the remaining residue analysed for NH_3_/NH_4_Cl and N_2_H_4_/N_2_H_4_·2HCl via ^1^H NMR spectroscopy (Supplementary Figs. 40, 45–56, 58, 61–73) and pdmab methods (vide infra). As previously reported^5^, N_2_H_4_ is only partially transferred under these conditions, and heating the distillation mixture should be avoided because N_2_H_4_ undergoes thermal decomposition to NH_3_, N_2_ and H_2_. Thus, after the distillation, the solids remaining in bulb A were also analysed for N_2_H_4_/N_2_H_4_·2HCl via the pdmab method (Supplementary Figs. 78, 79, 82–105, 108–119). The acidification of **1-**^**15**^**N**_**2**_ under a ^15^N_2_ atmosphere is described in Supplementary Information.

## Online content

Any methods, additional references, Nature Portfolio reporting summaries, source data, extended data, supplementary information, acknowledgements, peer review information; details of author contributions and competing interests; and statements of data and code availability are available at 10.1038/s41557-025-01867-z.

## Supplementary information

Supplementary Information Supplementary materials and methods (general experimental details, preparations and computational details), Figs. 1–156 and Tables 1–4.

Supplementary Data 1 Geometry optimized coordinates and single point energy of **1**.

Supplementary Data 2 Geometry optimized coordinates and single point energy of **2′**.

Supplementary Data 3 Geometry optimized coordinates and single point energy of **2-Li**.

Supplementary Data 4 Geometry optimized coordinates and single point energy of **2-K**.

Supplementary Data 5 Geometry optimized coordinates and single point energy of **3A**.

Supplementary Data 6 Geometry optimized coordinates and single point energy of **3B**.

## Data Availability

The X-ray crystallographic coordinates for structures reported in this Article have been deposited at the Cambridge Crystallographic Data Centre (CCDC) under deposition nos. CCDC 2377328 (**2-crypt**), 2355682 (**2-K**), 2377329 (**2-Li**), 2377330 (**3**), 2377331 (**4**) and 2377332 (**5**). These data can be obtained free of charge from The Cambridge Crystallographic Data Centre (www.ccdc.cam.ac.uk/data_request/cif). All other data are presented in the main text and Supplementary Information, and are also available from the corresponding authors on reasonable request. The data that support the findings of this study are openly available in the Zenodo repository at 10.5281/zenodo.15309919 (ref. ^87^).
