# Supplementary material for: Catalytic and stoichiometric stepwise conversion of side-on bound dinitrogen to ammonia mediated by a uranium complex
Source: Nat Chem. 2025 Jul 16;17(9):1425–33. doi: 10.1038/s41557-025-01867-z (PMC12411223; doi:10.1038/s41557-025-01867-z)
Supplement: Supplementary file 1 — Supplementary materials and methods (general experimental details, preparations and computational details), Figs. 1–156 and Tables 1–4. [file 41557_2025_1867_MOESM1_ESM.pdf]

nature chemistry

Supplementary information

https://doi.org/10.1038/s41557-025-01867-z

Catalytic and stoichiometric stepwise conversion of side-on bound dinitrogen to ammonia mediated by a uranium complex

In the format provided by the authors and unedited

INDEX

SUPPLEMENTARY METHODS **2**

EXPERIMENTAL CONSIDERATIONS 2
GENERAL 2
PHYSICAL MEASUREMENTS 2
SYNTHESES AND SYNTHETIC TRIALS 4
CATALYTIC STUDIES 8
COMPUTATIONAL DETAILS 11
CRYSTALLOGRAPHIC DETAILS 11

SUPPLEMENTARY FIGURES **14**

SOLID-STATE STRUCTURES 15
NMR SPECTROSCOPY 18
Syntheses and reactivity studies *18*
Acidifications and catalytic studies *32*
Miscellaneous *51*
UV-VIS-NIR SPECTRA 53
IR SPECTRA 74
RAMAN SPECTRA 78
EPR SPECTRA 81
SQUID MAGNETOMETRY 83
DENSITY FUNCTIONAL THEORY MOLECULAR ORBITAL (MO) REPRESENTATIONS 90

SUPPLEMENTARY TABLES **93**

SUPPLEMENTARY REFERENCES **97**

SUPPLEMENTARY METHODS

Experimental Considerations

General

For the synthesis and characterisation of complexes 1-5 and catalytic trials. Unless otherwise noted, all manipulations were carried out at ambient temperatures under an inert dinitrogen atmosphere using standard Schlenk line or glovebox (MBraun UNIlab pro) techniques. Low-temperature syntheses were performed in a glovebox-fitted –40 ºC freezer. Catalytic trials were carried out using a J. Young-sealable two-bulb apparatus (**Supplementary Figure 1**). Water and oxygen levels were kept below 0.1 ppm at all times. Glass-coated stir bars were used instead of the Teflon-coated ones to prevent unwanted PTFE reactivity during reduction reactions. Anhydrous solvents were purchased from Sigma-Aldrich and further dried over potassium/benzophenone (for THF, toluene, and diethyl ether), sodium sand/benzophenone (for *n*-hexane and *n*-pentane), and distilled. 2,5-dimethylfuran was distilled and dried over 3 Å molecular sieves for several days prior to use. Deuterated solvents for NMR spectroscopy (THF-d_8, toluene-d_8) were purchased from Cortecnet, freeze-degassed and distilled over K/benzophenone. DMSO-d_6 and cyclohexane-d_{12} were freeze-degassed and dried over 3 Å molecular sieves for several days. Depleted uranium turnings were purchased from IBI Labs, Florida, USA (**EPFL**) and depleted uranium turnings and uranium trioxide were obtained from the National Nuclear Laboratory (**UoM**). Unless otherwise noted, all reagents were purchased from commercial suppliers and used without further purification. [U^{IV}($Tren^{DMBS}$)Cl] (see below)[1a] and KC_8[2] were prepared according to the respective published procedures.

For the acidification procedure and $^{15}N_2$ studies. Unless stated otherwise, all manipulations were carried out using Schlenk techniques, or an MBraun UniLab glovebox, under an atmosphere of dry dinitrogen or argon. Solvents were dried by passage through activated alumina towers, degassed and stored over potassium mirrors (n-pentane, n-hexane, Et_2O). For the catalytic acidifications, Et_2O was degassed prior to each use. Deuterated solvents were distilled from potassium, degassed by three freeze-pump-thaw cycles, and stored under argon. Acidification glassware was soaked in aqua regia for 12 hours before use, and all glassware was silylated with TMSCl (TMS = $SiMe_3$) and stored overnight in a 150 °C oven, followed by flame drying under dynamic vacuum (1×10^{-3} mbar) before use. PCy_3, NaI, DMSO-d_6, HCl (2 M in Et_2O), $N_2H_4 \cdot 2HCl$, Na, K, Rb, and Cs metal and KOH were purchased from commercial suppliers and used as received. TMSI was stored over copper wire under an atmosphere of Ar. ^{t}BuOH was dried over activated 4 Å molecular sieves before use. [Et_3NH][Cl] was dried for 48 hours under dynamic vacuum (1×10^{-3} mbar) at 70 °C before use. $NaNO_3$ and $NaNO_2$ were dried for 48 hours under dynamic vacuum (1×10^{-3} mbar) at 100 °C before use. Graphite was dried for four hours under dynamic vacuum ($1x10^{-3}$ mbar) at 150 °C prior to use. RbC_8, CsC_8 and [Et_3NH][I] were synthesised according to published procedures.[2, 3] The acidic indicator solution used in the quantification of $N_2H_4 \cdot 2HCl$ was prepared by the addition of 3 g of para-dimethylaminobenzaldehyde (pdmab) to 150 mL of EtOH, 15 mL HCl_{conc} and 257.5 mL HCl (1 M), as reported previously.[4]

Note about pressure. Atmospheric pressure is the pressure exerted by the Earth's atmosphere and is equal to 1.01 bar (1 atm) at sea level. Gauge pressure is the pressure measured relative to atmospheric pressure and can intuitively be thought of as the pressure reading on the gas cylinder regulator. When measuring the gauge pressure, the atmospheric pressure is the baseline. Absolute pressure is the sum of gauge and atmospheric pressure, where $P_{absolute} = P_{gauge} + P_{atmospheric}$. For example, if a gas cylinder regulator reads 1 bar, then the absolute pressure is equal to 2.01 bar.

Caution: *Depleted uranium (primary isotope ^{238}U) is a weak α-emitter (4.197 MeV) with a half-life of 4.47×10^9 years. Manipulations and reactions should be carried out in monitored fume hoods or in an inert glovebox in a radiation laboratory equipped with α- and β-counting equipment.*

Physical measurements

NMR measurements were carried out using NMR tubes adapted with J-Young valves. **EPFL**: NMR spectra were recorded on a Bruker 400 MHz or 600 MHz spectrometers, and the chemical shifts are reported in ppm with residual H-solvent as internal reference. **UoM**: ^1H, $^{13}C\{^1H\}$, $^{31}P\{^1H\}$, and $^{29}Si\{^1H\}$ spectra were recorded on a Bruker AVIII HD 400 spectrometer operating at 400.13, 100.61, 161.98, and 79.50 MHz, respectively or on a JEOL JNM-ECZ 400 MHz spectrometer operating at 399.78, 100.52, 161.83, and 79.42 MHz, respectively;

chemical shifts are quoted in ppm and are relative to tetramethylsilane (^{1}H, ^{13}C, ^{31}P, ^{29}Si). ^{15}N NMR spectra were acquired using a Bruker AV NEO 700 spectrometer operating at 70.97 MHz; spectra were indirectly referenced to NH_3(l) at 0 ppm using the field frequency lock.

FTIR spectra were recorded on a Bruker Alpha spectrometer with a Platinum-ATR module in a glovebox (**UoM**) or with a Perkin Elmer 1600 Series FTIR spectrophotometer flushed with nitrogen, where for the measurement 0.5-0.7 mg of each compound were mixed with 35 mg of KBr in an agate mortar and pressed into a pellet (**EPFL**).

Raman spectra were recorded on a Horiba XploRA Plus Raman microscope using a 638 nm laser with a power of 1.5 mW. The number of scans and acquisition time were varied for each complex to inhibit sample decomposition. The power was adjusted using a power filter for each complex to inhibit sample decomposition.

UV/Vis/NIR spectra were recorded on a Perkin Elmer LAMBDA 1050 spectrometer where data was collected in 1 cm path length cuvettes and were run versus HCl (1 M) (**UoM**).

Elemental analyses were performed under inert atmosphere of nitrogen using a Thermo Scientific Flash 2000 Organic Elemental Analyzer at the Institute of Chemistry and Chemical Engineering at **EPFL**.

EPR analysis was performed at **EPFL** on a Bruker Elexsys E500 spectrometer working at 9.4 GHz frequency with an Oxford ESR900 cryostat for 6 K and 298 K measurements. Liquid sample was prepared by the dissolution of a solid compound in glassing solvent (2-methyltetrahydrofuran). The concentration of the sample was kept at 10 mM [(mmol spins)/L]. J. Young valve-capped quartz X-band EPR tubes (ID = 2.8 mm, OD = 3.8 mm, L = 300 mm) were used, and the sample was cantered in the cavity so the locker ring is at the 165 mm position.

Magnetic measurements were performed at **EPFL** using a Quantum Design MPMS3 superconducting quantum interference device (SQUID) magnetometer in a temperature range 2-300 K. The crushed crystalline sample was enclosed in an evacuated and flame-sealed quartz capsule and placed inside a plastic straw. Samples were restrained in an eicosane matrix to prevent sample torquing during measurements. The measurements were performed with applied magnetic field of 0.1 T in the zero-field cooled (ZFC) regime. Diamagnetic corrections (for ligand and eicosane) were applied using Pascal's constants.[5] The magnetic moment per uranium centre for compounds **1** and **2-crypt** were calculated using the formula:

$$\mu = \sqrt{\frac{8\chi T}{2}};$$

Syntheses and synthetic trials

Synthesis of [{U^{IV}(Tren^{DMBS})}$_2$(μ-η^2:η^2-N_2)] (1), modified procedure. Under an atmosphere of argon, a pale-green solution of [U^{IV}(Tren^{DMBS})Cl] (374.2 mg, 0.493 mmol, 1 eq.) in n-hexane (10 mL) was added to a Schlenk tube containing a glass-coated stir bar. Solid potassium graphite (199.8 mg, 1.478 mmol, 3 eq.) was then added in portions, which resulted in the formation of a dark-purple reaction mixture that was allowed to stir for 24 hours at room temperature. Full conversion to the [U^{III}(Tren^{DMBS})] species was confirmed by ^{1}H NMR spectroscopy. The resulting reaction mixture was filtered on a porosity "4" glass frit, and the solid residue was rinsed with n-hexane (3 x 0.5 mL) yielding a dark-purple solution. The volatiles were removed *in vacuo*, and the resulting purple solid was brought into the dinitrogen glovebox (1.016 bar absolute N_2 pressure) and dissolved in n-pentane (2 mL) to immediately form a dark-red solution. The solution was then stored at –40 ºC for 2 days to yield dark-red crystals of **1** in 48% yield (178.1 mg, 0.118 mmol) which were dried under N_2 flow. The mother liquor was concentrated to 1 mL, and stored at –40 ºC, yielding additional **1** (48.2 mg, 0.032 mg) after two days. The procedure was repeated one more time (concentration to 0.5 mL and storage at –40 ºC), yielding a third and final crop of **1** after two days (0.024 mmol, 37.1 mg). Total yield 71% (263.4 mg). It was found that the use of *n*-hexane instead of pentane for the crystallisation step resulted in lower overall yield.

Elemental analysis: *Calcd.* for $C_{48}H_{114}N_{10}Si_6U_2$ (**1**): C, 39.06; H, 7.78; N, 9.49. *Found:* C, 39.22; H, 7.79; N, 9.30.

^{1}H NMR (400 MHz, toluene-d$_8$, 298 K): δ 10.95 (s, 12H, CH_2), 7.21 (s, 12H, CH_2), 3.84 (s, 54H, C(CH_3)$_3$), –20.24 ppm (s, 36H, Si(CH_3)$_2$) (**Supplementary Figure 9**). **ATR-IR** (ν/cm^{-1}): 2947 (m), 2922 (m), 2877 (m), 2848 (s), 1463 (m), 1387 (w), 1346 (br), 1243 (s), 1136 (w), 1062 (s), 1020 (br), 922 (s), 766 (vs), 766 (vs), 711 (m), 655 (m) **(Supplementary Figure 120)**. **Raman** (ν/cm^{-1}): 69 (m), 126 (w), 180 (m), 213 (w), 263 (w), 309 (s), 333 (m), 355 (s), 462 (br, w), 535 (m), 573 (w), 636 (w), 746 (s), 804 (s), 908 (br, w), 988 (w), 1113 (w), 1183 (w), 1206 (w), 1239 (m), 1303 (s), 1345 (w) (**Supplementary Figure 128**).

Note: When the complex **1** was redissolved in toluene-d$_8$ (**Supplementary Figure 9**) and cyclohexane-d$_{12}$ under a N_2 pressure of 1.01 bar, it was found to be in equilibrium with [U^{III}(Tren^{DMBS})] in an approximate ratio of 2:1. On the contrary, the ^{1}H NMR spectrum of complex **1** in THF-d$_8$ showed mostly [U^{III}(Tren^{DMBS})] resonances (**Supplementary Figure 10**). As evidenced by ^{1}H NMR spectroscopy (**Supplementary Figure 11**), we note that dissociation of **1** to free N_2 and [U^{III}(Tren^{DMBS})] can be suppressed during recrystallisation by increasing the gauge pressure of N_2 to 1.5 bar (absolute pressure 2.51 bar), leading to the shift in **1**:[U^{III}(Tren^{DMBS})] equilibrium from 2:1 to 5:1 (**Supplementary Figure 12**).

Synthesis of [{U^{IV}(Tren^{DMBS})}$_2$(μ-η^2:η^2-$^{15}N_2$)] (1-$^{15}N_2$). Under an atmosphere of argon, a Schlenk flask was charged with a glass-coated stirrer bar and a solid mixture of [U^{IV}(Tren^{DMBS})Cl] (750 mg, 1 mmol, 1 eq.) and potassium graphite (400 mg, 3 mmol, 3 eq.). At –40°C, hexane (20 mL) was added, stirred cold for five minutes, and the reaction mixture allowed to warm slowly to room temperature resulting in the formation of a dark brown/purple suspension, which was stirred for 36 hours. The suspension was then filtered, volatiles removed *in vacuo*, and the dark purple solid dried for two hours. Full conversion to the [U^{III}(Tren^{DMBS})] species was confirmed by ^{1}H NMR spectroscopy. Under an atmosphere of argon, pentane (2 mL) was added to form a very dark purple solution. The Schlenk flask was then submerged in a LN_2 dewar (–196 °C) and frozen. The entire apparatus was placed under a dynamic vacuum (1x10^{-3} mbar) for 10 minutes before being sealed. The Schlenk flask was then connected to an ampoule containing $^{15}N_2$ gas (dried over activated 4 Å molecular sieves for one week) via a three-way connector and the Schlenk flask was placed under a $^{15}N_2$ atmosphere. The mixture was allowed to thaw, resulting in a colour change to deep red. The solution was then stored at –35 ºC for 3 days to yield dark-red crystals of **1-$^{15}N_2$** in a 63% yield (476 mg, 0.315 mmol).

^{1}H NMR (400 MHz, benzene-d$_6$, 298 K): δ 10.64 (s, 12H, CH_2), 7.22 (s, 12H, CH_2), 3.69 (s, 54H, C(CH_3)$_3$), –20.12 ppm (s, 36H, Si(CH_3)$_2$) (**Supplementary Figure 13**). **ATR-IR** (ν/cm^{-1}): 2951 (m), 2925 (m), 2879 (m), 2847 (s), 1462 (s), 1387 (w), 1346 (w), 1245 (s), 1139 (w), 1071 (s), 1028 (m), 943 (s), 925 (w), 822 (w), 799 (s), 767 (s), 708 (m), 655 (s), 590 (m), 562 (m), 545 (w), 524 (m), 445 (s) (**Supplementary Figure 121**). **Raman** (ν/cm^{-1}): 35 (s), 58 (w), 82 (s), 123 (m), 177 (s), 197 (w), 201 (w), 219 (w), 239 (w), 263 (w), 293 (w), 339 (w), 355 (m), 373 (m), 419 (m), 455 (s), 567 (s), 655 (m), 671 (w), 703 (m), 743 (w), 769 (w), 811 (m), 890 (w), 918 (w), 931 (m), 1058 (w), 1135 (w), 1176 (m), 1201 (m), 1235 (w), 1263 (w), 1327 (w), 1353 (w), 1403 (w), 1438 (m), 1457 (m) **(Supplementary Figure 129)**.

Synthesis of [K(2.2.2-cryptand)][{U^{IV}(Tren^{DMBS})}$_2$(μ-η^2:η^2-N_2)] (2-crypt). Under an atmosphere of dinitrogen, a solution of **1** (130.1 mg, 0.086 mmol, 1 eq.) and 2.2.2-cryptand (32.5 mg, 0.086 mmol, 1 eq.) in toluene (4 mL)

was added to a Schlenk tube containing a glass-coated stir bar. Solid potassium graphite (11.7 mg, 0.086 mmol, 1 eq.) was then added at room temperature, and the reaction was left stirring for 3 hours to yield a dark-brown mixture. The volatiles were then removed *in vacuo*, and soluble residues were extracted into THF (4 mL) in order to facilitate graphite removal by filtration on a porosity "4" glass frit. Volatiles were then removed *in vacuo* from the filtrate, the residue dried for one hour, and toluene (~2 mL) added. Storage of the brown solution at –40 ºC for 2 days yielded **2-crypt**·$2C_7H_8O$ as dark-brown crystals in an 83% yield (135.6 mg, 0.072 mmol). **2-crypt** is readily soluble in THF and only modestly in toluene; the complex was found to be stable towards nitrogen loss in THF and toluene (no change in the ^{1}H NMR spectra after dissolution or a freeze-pump-thaw cycle).

Elemental analysis: *Calcd.* for $C_{69.5}H_{154}KN_{12}O_6Si_6U_2$ (**2-crypt** · $0.5C_7H_8O$): C, 43.08; H, 8.01; N, 8.67. *Found:* C, 43.04; H, 8.05; N, 7.93.

^{1}H NMR (400 MHz, 298 K, THF-d_8): δ 86.21 (s, 12H, CH_2), 25.89 (s, 12H, CH_2), 3.06-3.03 (m, 24H, 2.2.2-crypt), 2.04 (m, 12H, crypt), –13.02 (s, 54H, C(CH_3)$_3$), –33.64 (s, 36H, Si(CH_3)$_2$) (**Supplementary Figure 15, a**). **^{29}Si{^{1}H} NMR** (79.5 MHz, 298 K, THF-d_8): δ –181.81 (**Supplementary Figure 15, b**). **ATR-IR** (ν/cm^{-1}): 2951 (m), 2918 (m), 2877 (m), 2838 (s), 1467 (w), 1459 (w), 1444 (w), 1354 (m), 1294 (w), 1235 (m), 1132 (w), 1103 (s), 1072 (s), 1058 (m), 1031 (m), 926 (s), 889 (m), 803 (vs), 778 (s), 764 (s), 721 (s), 669 (m), 647 (s), 552 (br) (**Supplementary Figure 123**). **Raman** (ν/cm^{-1}): 42 (w), 65 (m), 141 (w), 183 (m), 213 (w), 260 (w), 309 (s), 336 (m), 358 (w), 461 (br, w), 539 (m), 575 (w), 604 (w), 640 (w), 747 (s), 806 (s), 896 (w), 989 (w), 1129 (w), 1189 (w), 1238 (m), 1306 (s), 1340 (w), 1360 (w) (**Supplementary Figure 131**).

Note: All attempts to further reduce **2-crypt** by addition of excess KC_8/[2.2.2] equivalents to either **1** or to isolated **2-crypt** lead only to oily intractable mixtures both in toluene-d_8 and THF-d_8. No solids could be obtained from any of these mixtures despite repeated attempts.

Synthesis of [K{U^{IV}(Tren^{DMBS})}$_2$(μ-η^2:η^2-N$_2$)] (2-K). Under an atmosphere of dinitrogen, solid potassium graphite (0.217 mmol, 29.3 mg, 3 eq.) pre-chilled to –40 ºC was added to a cold solution of **1** (108.7 mg, 0.072 mmol, 1 eq.) in *n*-hexane. The mixture was left to stir for 3 days at –40 ºC, then filtered on a porosity "4" glass frit, also pre-chilled to –40 ºC and concentrated to 0.5 mL while cold. Dark brown-green crystals of **2-K** were obtained from *n*-hexane after 2 days at –40 ºC, in a 62% yield (69.1 mg, 0.045 mmol).

Elemental analysis: *Calcd.* for $C_{51}H_{121}KN_{10}Si_6U_2$ (**2-K** · $0.5C_6H_{14}$): C, 39.31; H, 7.83; N, 8.99. *Found:* C, 39.17; H, 7.78; N, 8.63.

^{1}H NMR (400 MHz, 233 K, toluene-d_8): δ 5.88 (s, br.), –21.20 (s, very br.), 83.58 (s, very br.) (**Supplementary Figure 16, a**). (193 K): –12.46 (s, very br.), –27.79 (s, very br.), –36.57 (s, very br.), –132.49 (s, very br.), –150.65 (s, very br.) (**Supplementary Figure 16, b**). **ATR-IR** (ν/cm^{-1}): 2947 (m), 2922 (m), 2877 (m), 2846 (s), 1459 (m), 1385 (w), 1348 (w), 1239 (s), 1138 (w), 1060 (s), 1027 (m), 1004 (w), 922 (s), 887 (m), 820 (vs), 803 (vs), 774 (vs), 762 (vs), 715 (s), 698 (s), 672 (m), 647 (s), 593 (m), 563 (m), 534 (s) (**Supplementary Figure 124**). **Raman** (ν/cm^{-1}): 39 (w), 60 (w), 130 (w), 181 (m), 209 (w), 260 (w), 309 (s), 334 (s), 355 (w), 433 (w), 463 (w), 535 (m), 574 (w), 597 (w), 634 (w), 745 (s), 804 (s), 895 (w), 985 (w), 1107 (w), 1125 (w), 1158 (w), 1185 (br, w), 1237 (m), 1305 (s), 1334 (w), 1360 (w) (**Supplementary Figure 132**).

Note: **2-K** is stable over two days in toluene-d_8 at –40 °C but decomposes completely to unidentified species in toluene-d_8 at room temperature after 6 hours, accompanied by a solution colour change from dark-brown to light-yellow (**Supplementary Figure 17**).

Note: Further reduction of the complex **2-K** in hexane or toluene-d_8 with excess KC_8 resulted in light-yellow reaction mixtures and reduction-induced decomposition. No species could be isolated from these mixtures by crystallisation and ^{1}H NMR showed no known resonances.

Note: When the same reaction in hexane was carried in the presence of lithium iodide as a source of lithium cations, crystals of [Li{U(Tren^{DMBS})}$_2$(μ-η^2:η^2-N$_2$)] (**2-Li**) were obtained in low yield after storing the filtered reaction mixture at –40 ºC for 2 days. Attempts to scale this reaction up in order to obtain analytically meaningful quantities of **2-Li** were unsuccessful, most probably due to insolubility of lithium iodide in hexane.

Synthesis of [Li$_2${U^{IV}(Tren^{DMBS})}$_2$(μ-η^2:η^2-N$_2$)] (3). Under an atmosphere of dinitrogen, crystalline **1** (130.4 mg, 0.088 mmol, 1 eq.) was combined with solid lithium iodide (23.7 mg, 0.177 mmol, 2 eq.). At room temperature, diethyl ether (2 mL) was added, and the mixture stirred until fully dissolved, before being stored at –40 ºC for one hour. The resulting cold mixture was added to cold (–40 ºC) solid potassium graphite (23.9 mg, 0.177 mmol, 2

eq.) in cold diethyl ether (1 mL) and stirred for 3 days at –40 ºC before filtration on a porosity "4" glass frit pre-chilled to –40 ºC to obtain a dark brown/red solution. Concentration *in vacuo* to ~1 mL and storage at –40 ºC for four days yielded dark-red/brown crystals of **3** in 63% yield obtained in several crops (82.9 mg, 0.056 mmol). Once isolated, crystalline **3** is stable at room temperature and resistant towards N_2 loss under vacuum.

Elemental analysis: *Calcd.* for $C_{52}H_{124}Li_2N_{10}OSi_6U_2$ (**3** ·1$C_4H_{10}O$): C, 39.93; H, 7.99; N, 8.96. *Found:* C, 39.56; H, 7.79; N, 8.65.

^{1}H NMR (400 MHz, 298 K, toluene-d_8): 191.23 (s, 1H), 183.70 (s, 1H), 131.38 (s, 1H), 117.37 (s, 1H), 113.92 (s, 1H), 93.70 (s, 1H), 80.98 (s, 1H), 73.60 (s, 1H), 68.72 (s, 1H), 58.72 (s, 1H), 55.89 (s, 1H), 39.69 (s, 1H), 36.89 (s, 1H), 31.81 (s, 11H), 17.44 (s, 2H), 12.47 (s, 2H), 10.98 (s, 2H), 9.34 (s, 2H), 2.77 (s, 11H), –3.79 (s, 4H), –5.11 (s, 4H), –8.44 (s, 8H), –9.80 (s, 6H), –15.53 (s, 7H), –20.48 (s, 7H), –21.58 (s, 4H), –22.51 (s, 1H), –28.36 (s, 9H), –29.39 (s, 1H), –30.26 (s, 2H), –32.36 (s, 2H), –40.40 (s, 2H), –48.58 (s, 2H), –52.24 (s, 3H), –58.49(s, 1H), –79.78 (s, 1H), –86.11 (s, 1H), –129.45 (s, 3H) (**Supplementary Figures 18-19**). **ATR-IR** (ν/cm^{-1}): 2949 (m), 2922 (m), 2879 (m), 2846 (s), 1461 (m), 1385 (w), 1354 (w), 1241 (s), 1056 (s), 1021 (w), 1002 (w), 976 (w) 926 (s), 896 (s), 817 (vs), 801 (vs), 766 (vs), 709 (s), 649 (s), 622 (s), 550 (s), 528 (s) (**Supplementary Figure 125**). **Raman** (ν/cm^{-1}): 44 (w), 64 (m), 136 (w), 182 (m), 212 (w), 263 (w), 308 (s), 335 (m), 355 (w), 460 (w), 539 (m), 574 (w), 601 (w), 637 (w), 747 (s), 805 (s), 895 (w), 985 (m), 996 (w), 1126 (w), 1188 (w), 1241 (m), 1307 (s), 1334 (w), 1360 (w) (**Supplementary Figure 133**).

*Complex **3** could also be obtained from the reduction of isolated **2-K**:* Diethyl ether (2 mL) pre-chilled to –40 ºC in a glovebox freezer was added to **2-K** (9.4 mg, 0.0062 mmol, 1 eq.) at –40 ºC to give a dark brown solution. The resulting solution was added to solid cold potassium graphite (0.9 mg, 0.0062 mmol, 1 eq.) and lithium iodide (1.7 mg, 0.0124 mmol, 2 eq.), and stirred at –40 ºC for 24 hours, which was accompanied by a gradual colour change to dark-red. Subsequently, the reaction mixture was filtered on a cold porosity '4' glass filter frit and all volatiles were removed *in vacuo*, resulting in a brown-red microcrystalline solid. The ^{1}H NMR spectrum of the solid showed the formation of the hydrazido- complex **3** as the only identifiable product (**Supplementary Figures 20-21**).

Note: When the reductions were carried out with KC_8 in the absence of Li cations, the ^{1}H NMR spectrum showed the formation of multiple species that could not be isolated in clean form, irrespective of the stoichiometry or the solvent utilised.

Synthesis of [$Li_4(OEt_2)\{U^{IV}(Tren^{DMBS})\}_2(\mu\text{-}N)_2$] (4). Under an atmosphere of dinitrogen, crystalline **1** (107.9 mg, 0.073 mmol, 1 eq.) was combined with solid lithium iodide (58.8 mg, 0.439 mmol, 6 eq.). At room temperature, diethyl ether (3 mL) was added and the mixture stirred until fully dissolved, before being stored at –40 ºC for one hour. The resulting mixture was added to cold solid potassium graphite (59.3 mg, 0.439 mmol, 6 eq.) in diethyl ether (1 mL) and allowed to stir for 3 days at –40 ºC before filtration on a cold porosity "4" glass frit to obtain a dark brown solution. Concentration *in vacuo* to ~1 mL and storage at –40 ºC for three days yielded dark-brown crystals of **4** · 1$C_4H_{10}O$ in a 56% yield (61.6 mg, 0.056 mmol).

Elemental analysis: *Calcd.* for $C_{52}H_{124}Li_4N_{10}OSi_6U_2$ (**4**): C, 39.58; H, 7.92; N, 8.88. *Found:* C, 39.48; H, 7.89; N, 8.89.

^{1}H NMR (400 MHz, 298 K, toluene-d_8): δ 147.00 (s, 1H), 140.31 (s, 1H), 134.84 (s, 1H), 113.57 (s, 1H), 101.86 (s, 1H), 83.95 (s, 1H), 56.14 (s, 1H), 55.76 (s, 1H), 53.64 (s, 1H), 50.56 (s, 1H), 41.05 (s, 1H), 40.89 (s, 1H), 39.58 (s, 1H), 38.34 (m, 2H), 32.09 (m, 2H), 24.33 (s, 1H), 23.61 (s, 8H), 14.25 (s, 2H), 10.26 (s, 2H), 9.48 (s, 6H), 6.58 (s, 10H), –2.45 (s, 8H), –5.06 (s, 10H), –10.72 (s, 11H), –12.94 (s, 4H), –17.44 (s, 4H), –19.80 (s, 13H), –22.03 (s, 3H), –28.78 (s, 1H), –35.26 (s, 4H), –39.37 (s, 2H), –47.66 (s, 3H), –53.07 (s, 1H), –67.44 (s, 2H), –81.85 (s, 1H), –95.57 (s, 3H), –113.73 (s, 1H), –133.97 (s, 3H) (**Supplementary Figure 22-23**). **IR** (KBr pellet, ν/cm^{-1}): 2953 (s), 2928 (s), 2883 (m), 2852 (s), 1630 (m), 1470 (m), 1388 (m), 1358 (m), 1249 (m), 1184 (w), 1093 (m), 1060 (m), 1025 (m), 1006 (m), 930 (m), 901 (m), 825 (s), 775 (m), 708 (m), 654 (m), 607 (m), 528 (w), 467 (w) (**Supplementary Figure 126**).

*Complex **4** could also be obtained by reduction of **2-K**:* At –40º C, in a glovebox-fitted freezer diethyl ether (2 mL) was added to **2-K** (13.5 mg, 0.0089 mmol, 1 eq.) to give a dark brown solution. The resulting solution was added to solid potassium graphite (3.6 mg, 0.027 mmol, 3 eq.) and lithium iodide (4.8 mg, 0.036 mmol, 4 eq.), both pre-chilled at –40º C, and stirred at –40 ºC for 24 hours, Subsequently, the reaction mixture was filtered at –40º C on a pre-chilled porosity '4' glass filter frit and concentrated *in vacuo* to approximately 0.5 mL. Storage of

the solution at –40 ºC for 1 day yielded dark-brown microcrystals that were identified as the bis-nitride complex **4** by SC-XRD and ^{1}H NMR (**Supplementary Figure 24-25**, performed in toluene-d_8).

Complex ***4*** *could also be obtained by reduction of* ***3****:* At –40º C, in a glovebox-fitted freezer: cold diethyl ether (2 mL) was added to **3** (16.2 mg, 0.011 mmol, 1 eq.) to give a dark red solution. The resulting solution was added to solid cold potassium graphite (2.9 mg, 0.022 mmol, 2 eq.) and lithium iodide (2.9 mg, 0.022 mmol, 2 eq.) and stirred at –40 ºC for 24 hours, which was accompanied by a gradual colour change to dark brown. Subsequently, the reaction mixture was filtered on a cold porosity '4' glass filter frit and concentrated *in vacuo* to approximately 0.5 mL. Storage of the solution at –40 ºC for 1 day yielded dark-brown microcrystals that were identified as the bis-nitride complex **4** by SC-XRD and ^{1}H NMR.

Synthesis of [Li_3{U^{IV}($Tren^{DMBS}$)}$_2$(μ-N)(μ-NH)] (5). A dark-red/brown solution of **3** (37.1 mg, 0.025 mmol, 1 eq.) in toluene (0.5 mL) was transferred to a J. Young valve-capped tube and connected to a Schlenk line. The resulting solution was degassed by three cycles of freeze-pump-thawing and hydrogen gas (1.01 bar) was added to the reaction mixture and the tube was warmed up to room temperature. A gradual colour change to brown was observed over the course of two days as full consumption of the starting material occurred. Brown crystals of **5** were collected from the reaction mixture after leaving it to stand at room temperature (7.9 mg) for 3 days. An additional crop (4.4 mg) of crystalline **5** was isolated after 1 day upon placing the reaction mixture at –40 ºC.

Elemental analysis: *Calcd.* for $C_{48}H_{115}Li_3N_{10}Si_6U_2$ (**5**): C, 38.49; H, 7.74; N, 9.35. *Found:* C, 38.97; H, 7.75; N, 9.32.

^{1}H NMR (400 MHz, 298 K, toluene-d_8): δ 169.78 (s, 2H), 134.53 (s, 1H), 125.88 (s, 1H), 68.40 (s, 1H), 46.12 (s, 2H), 37.31 (s, 2H), 32.58 (s, 1H), 28.90 (s, 2H), 24.94 (s, 1H), 22.95 (m, 6H), 21.65 (s, 1H), 21.48 (s, 1H), 18.45 (d, 2H), 16.21 (s, 2H), 15.48 (s, 1H), 13.82 (s, 4H), 12.86 (s, 2H), 11.73 (s, 1H), 11.07 (s, 1H), 10.09 (s, 2H), 9.25 (s, 1H), 8.19 (s, 4H), 7.86 (s, 1H), 6.83 (s, 2H), 4.09 (s, 1H), 3.20 (s, 1H), 1.59 (s, 2H), 1.26 (s, 3H), –0.70 (s, 1H), –1.26 (s, 1H), –3.43 (s, 5H), –4.33 (s, 1H), –7.18 (s, 2H), –7.83 (s, 2H), –12.89 (m, 2H), –13.67 (d, 9H), –15.20 (s, 2H), –19.99 (s, 2H), –29.18 (s, 1H), –32.11 (s, 1H), –36.08 (s, 2H), –39.75 (s, 2H), –43.11 (s, 1H), –46.51 (s, 1H), –49.14 (s, 1H), –50.62 (s, 1H), –58.78 (s, 2H), –66.67 (s, 2H), –74.31 (s, 2H), –170.25 (s, 1H, N*H*) (**Supplementary Figure 29-30**). **^{7}Li NMR** (156 MHz, 298 K, toluene-d_8): δ 126.33, 107.45, 90.86 (**Supplementary Figure 28**). **IR** (KBr pellet, ν/cm^{-1}): 3313 (w, N–H), 2953 (s), 2929 (s), 2883 (s), 2852 (s), 2735 (w), 2705 (w), 2672 (w), 1605 (m), 1468 (m), 1405 (w), 1388 (w), 1357 (w), 1246 (m), 1133(w), 1113 (w), 1092 (m), 1072 (m), 1057 (m), 1023 (m), 1011 (w), 967 (m), 925 (s), 903 (m), 888 (s), 825 (s), 777 (s), 717 (m), 655 (s), 586 (m), 560 (m), 527 (m), 505 (m) (**Supplementary Figure 127**).

A second species observed in the ^{1}H NMR spectrum (**Supplementary Figure 26-27**) of the mother liquor measured in toluene-d_8 could not be isolated despite repeated attempts.

Note: A few crystals of complex **5** could also be isolated from a reaction mixture obtained after the exposure of bis-nitride complex **4** to a 1.01 bar pressure of hydrogen gas, in which case the reaction (accompanied by a colour change from dark-brown to brown) is immediate. However, the ^{1}H NMR spectrum of the reaction mixture shows the presence of multiple species.

Synthesis of [Li_3{U^{IV}($Tren^{DMBS}$)}$_2$(μ-N)(μ-ND)] (5-D_2) through the reaction of 3 with D_2 gas. A dark-red/brown solution of crystalline **3** (37.1 mg, 0.025 mmol, 1 eq.) in toluene (0.3 mL) was transferred to a J. Young valve-capped NMR tube and connected to a Schlenk line. The resulting solution was degassed by three cycles of freeze-pump-thawing and deuterium gas (0.5 bar absolute pressure of D_2) was added to the reaction mixture and the tube was warmed up to room temperature. An immediate colour change from dark-red/brown to brown was observed. After 1 hour, all volatiles were removed *in vacuo* and toluene-d_8 (0.3 mL) was added. The ^{1}H NMR spectrum of the reaction mixture showed the full set of resonances assigned to **5** (**Supplementary Figure 31-33**) except for the broadened N*H* singlet at –170.25 ppm (**Supplementary Figure 32**) which is not present in the deuterated mixture. Dark-brown crystals of **5-D_2** (6.6 mg) were isolated from the reaction mixture after 2 days at –40 ºC.

IR (KBr pellet, ν/cm^{-1}): 2953 (s), 2928 (s), 2883 (s), 2851 (s), 2704 (w), 2452 (w, N–D), 1468 (m), 1444 (w), 1387 (w), 1357 (w), 1288 (m), 1133 (w), 1111 (w), 1091 (m), 1056 (m), 1024 (m), 968 (m), 924 (s), 902 (m), 889 (s), 825 (s), 779 (s), 717 (m), 654 (s), 584 (m), 526 (w), 505 (w), 484 (w) (**Supplementary Figure 127**).

Note: analogously to the hydrazido- complex **3**, when the bis-nitride complex **4** is exposed to deuterium gas (0.5 bar absolute pressure of D_2) in toluene, gradual colour change to brown is observed and full set of resonances belonging to **5** apart from the N*H* singlet (–170.21 ppm) was identified as the only known species (by ^{1}H and ^{7}Li NMR, **Supplementary Figure 34**), after the dried reaction mixture was subsequently brought to toluene-d_8 for analysis. Other lithium-ligated species present in the mixture (**Supplementary Figure 35**) could not be isolated despite repeated attempts.

Reaction of 1, 2-crypt, 3 and 4 with excess HCl. To J. Young valve-capped NMR tubes containing solid complexes **1** to **4,** a solution of HCl (2 M, 2.0 mL in total) in diethyl ether was added at –80 ºC in a glovebox-fitted cold well. Upon warming up each tube to –40 ºC and then subsequently to room temperature, discoloration of solids occurred, and formation of light-yellow solutions was observed. After 1 hour, all volatiles were removed *in vacuo* and the resulting solids were dissolved in DMSO-d_6 (0.5 mL). 2,5-dimethylfuran was added to the tubes as an internal standard for the quantitative detection of NH_4Cl. Formation of 0.18 eq. of NH_4Cl for **1,** 0.43 eq. for **2-crypt,** 1.57 eq. for **3**, and 2.0 eq. for **4** were detected by quantitative ^{1}H NMR experiments (**Supplementary Figure 36-39**). Under similar experimental conditions, the parent monomeric complex [U^{IV}Tren^{DMBS})Cl] produced 0.03 eq. of NH_4Cl.

Synthesis of [U^{IV}(Tren^{DMBS})Cl]. Compound was prepared according to the previously reported procedure.[1a] At –80 ºC in a glovebox-fitted cold well, pre-chilled colourless suspension of the [(Tren^{DMBS})$Li_3(THF)_{2.2}$] (1332 mg, 2 mmol, 1 eq.) in 20 mL THF was added in portions to cold green solution of uranium tetrachloride (760 mg, 2 mmol, 1 eq.) in 20 mL of THF while stirring. After 1 hour, the mixture was warmed up to room temperature and further stirred for 16 hours. Afterwards, all the volatiles were removed *in vacuo*, the product was extracted into pentane (30 mL) and filtered on a porosity ‘4’ glass filter frit to remove the insoluble lithium chloride. The volatiles were again removed *in vacuo* from the filtrate and the resulting solid was recrystallised from minimal pentane (~ 4 mL) at –40 ºC to give light-green crystals in 62% yield (942 mg). Characterisation data matched the one reported previously.

^{1}H NMR (400 MHz, toluene-d_8, 298 K): δ 7.73 (s, 6H, CH_2), 6.47 (s, 27H, C(CH_3)$_3$), 5.89 (18H, Si(CH_3)$_2$), –22.73 ppm (s, 6H, CH_2).

Catalytic studies

The glass distillation apparatus (**Supplementary Figure 1**)[4] required for these procedures contains two separate glass bulbs (A and B), which can be sealed, but are connected to one another via a glass tube for which a right-angled tap is attached allowing the entire apparatus to be placed under inert gas or vacuum.

Catalytic trials under $^{14}N_2$ atmosphere. Inside a N_2 glovebox, the J. Young valve-capped distillation apparatus (**Supplementary Figure 1**) was placed in a glovebox-fitted cold well, which was in turn pre-chilled to –140 ºC using an external LN_2 dewar . Once the temperature was found to be stable, 2 M HCl in diethyl ether (2 mL, stored at –40 ºC) was pipetted into bulb *A* and frozen. The apparatus was connected to the glovebox vacuum line and placed under dynamic vacuum. After 5 minutes, the J. Young valve leading to the bulb *A* was sealed. The apparatus was disconnected from the vacuum line, after which the diuranium complex **1** or **2-crypt**, KC_8, [Cy_3PH][I], and a 1 cm glass-coated stir bar were added to bulb *B*. After allowing the contents of bulb *B* to cool, solvent (diethyl ether, toluene or hexane) pre-chilled near the melting point (3 mL) was carefully added drop-by-drop to the solid mixture in bulb *B*. After the addition was complete, bulb *B* was sealed, and the apparatus was taken out of the box and both bulbs placed in a –78 ºC dry ice/acetone bath.

The mixture was allowed to warm up to room temperature, and bulb B stirred for 15 hours. After that, both bulbs were frozen in liquid nitrogen (–196 °C), and the apparatus was placed under static vacuum. The contents of bulb *B* were then distilled onto the frozen HCl in diethyl ether in bulb *A*. The J. Young valve separating the two bulbs (Young valve of bulb *A*) was closed, bulb *B* was frozen in liquid nitrogen (–196 °C) and a solution of NaO^{t}Bu (1.2-fold molar excess with regards to the acid used) in diethyl ether (3 mL) was added under a strong flow of nitrogen *via* syringe, after which bulb *B* was sealed, frozen in liquid nitrogen (–196 °C), and placed under dynamic vacuum again. A second transfer of the volatiles was performed under static vacuum.

Once the distillation was complete, the apparatus was warmed to room temperature and brought back into the glovebox, where all the volatiles were removed *in vacuo* from bulb *A*, and the solids were analysed for the presence

of NH_4Cl *via* their dissolution in DMSO-d_6 (0.4 mL), containing 2,5-dimethylfuran (2,5-DMF/DMFu) or $DMSO_2$ as an integration standard (**Supplementary Figure 42-44**).

Modified synthesis of [Cy_3PH][I]. Under an atmosphere of argon, a solution of Me_3SiI (11.3 mL, 56.5 mmol) in Et_2O (20 mL) was added dropwise to a pre-cooled (–78 °C) solution of ^{t}BuOH (5.4 mL, 56.5 mmol) in Et_2O (20 mL) in the absence of light before being allowed to slowly warm to room temperature to form a pale yellow HI solution. To a pre-cooled (–78 °C) solution of PCy_3 (5 g, 17.8 mmol) in Et_2O (30 mL), was then added the freshly prepared HI solution dropwise resulting in the immediate precipitation of [Cy_3PH][I] as a white solid. The suspension was stirred for 1 hour, before the phosphonium salt was isolated by filtration, washed with Et_2O (3 x 20 mL) and dried *in vacuo*. Yield: 22.9% (1.67 g, 4.08 mmol).

^{1}H NMR (400 MHz, C_6D_6, 298 K): δ 3.03 - 2.68 (br, 3H, C_6H_{11}), 2.36 (d, 1H, PCy_3HI), 2.10 – 1.05 (br, 30H, C_6H_{11}) (**Supplementary Figure 75**). **^{31}P{^{1}H} NMR** (162 MHz, C_6D_6, 298 K): 33.5 (**Supplementary Figure 76**).

Acidification of [{U^{IV}(Tren^{DMBS})}$_2$(μ-η^2:η^2-N_2)] under $^{14}N_2$ atmosphere. Under an atmosphere of argon, bulb *A* was charged with **1** (0.0052 mmol), the acid and reductant, along with a glass-coated stirrer bar. Bulb *A* was then sealed and transferred to a Schlenk line. Et_2O (stored under argon, 4-8 mL, *vide infra*) was added to bulb *B* via syringe and then bulb *B* was submerged in a LN_2 dewar (–196 °C) and frozen. The entire apparatus was placed under a dynamic vacuum (1x10^{-3} mbar) and sealed, leaving the apparatus under static vacuum. The LN_2 dewar (–196 °C) was removed from bulb *B* and immediately transferred to bulb *A* such that the frozen solvent in bulb B could thaw and distil onto the frozen solids in bulb *A*. After the distillation had concluded, the apparatus was placed under a dinitrogen atmosphere, sealed, and allowed to warm to room temperature. For reactions involving MC_8 (M = K, Rb, Cs) as the reductant, the reaction mixture was stirred for 17 hours. In the case of the alkali metals, the reaction mixtures were subsequently sonicated until the metal was finely dispersed (1-40 minutes) and the reactions were stirred for 72 hours. After this time, HCl (2 M in Et_2O, 2 mL) was added to bulb *B* via syringe and both bulbs were subsequently submerged into separate LN_2 (–196 °C) dewars and frozen. The entire distillation apparatus was then placed under a dynamic vacuum and sealed, leaving the vacuum under static vacuum, and the LN_2 dewar removed from bulb *A*. During this process, the reaction mixture in bulb A thaws, and the volatiles were distilled onto the frozen HCl in bulb B. Once the acid distillation is complete (approximately 30 minutes), bulb *A* was resubmerged in a LN_2 (–196 °C) dewar and the contents frozen. Bulb *B* was sealed, and aqueous KOH (30%, 4 mL) via syringe was added to bulb *A* under a flow of dinitrogen. Once the contents had frozen, the entire apparatus was once again placed under a dynamic vacuum and sealed, leaving the apparatus under static vacuum, and the LN_2 (–196 °C) dewar removed from bulb *A*. The base distillation was performed with vigorous stirring for 1 hour, after which time both bulbs *A* and *B* were sealed, and bulb *B* was allowed to warm to room temperature and stirred for 10 minutes. All volatiles in bulb *B* were subsequently removed *in vacuo* and the remaining residue analysed for NH_3/NH_4Cl and N_2H_4/$N_2H_4{\cdot}2HCl$ via ^{1}H NMR spectroscopy (**Supplementary Figures 40, 45-46, 58, 61-73**) and pdmab methods (*vide infra*). As previously reported,[5] N_2H_4 is only partially transferred under these conditions, and heating the distillation mixture should be avoided since N_2H_4 undergoes thermal decomposition to NH_3, N_2, and H_2. Thus, after the distillation, the solids remaining in bulb *A* were also analysed for N_2H_4/$N_2H_4{\cdot}2HCl$ via the pdmab method (**Supplementary Figures 78-79, 82-105, 108-119**).

NH_3 and N_2H_4 quantification procedures. Quantifications were performed as previously described.[4]

Catalytic Trials. Unless stated otherwise, all catalytic reduction and acidifications of N_2 were performed with **1** (0.0052 mmol) as the catalyst. 4 mL Et_2O was used as the solvent in entries 5,6, 7, 11, 14, 15, and 16 (for the 1.3 mM starting concentration of **1**), this was increased to 8 mL (0.65 mM) for entries 8, 9, 10, 12, 13, 17, 18, 19, 20, 21, 22, 23, and 24 due to increased scale of reagents (**Table 1**). Entries 26-34 were performed with 8 mL solvent (0.65 mM, **Supplementary Table 3**).

Acidification of [{U^{IV}(Tren^{DMBS})}$_2$(μ-η^2:η^2-$^{15}N_2$)] under $^{15}N_2$ atmosphere. Under an atmosphere of argon, bulb *A* was charged with ^{15}N-isotopically labelled catalyst, **1-$^{15}N_2$** (0.0052 mol), the acid and reductant, along with a glass-coated stirrer bar. Bulb *A* was then sealed and transferred to a Schlenk line. Et_2O (freeze-pump-thawed three times and stored under 1.01 bar $^{15}N_2$, 4 or 8 mL, *vide infra*) was added to bulb *B* via syringe and then bulb *B* was submerged in a LN_2 dewar (–196 °C) and frozen. The entire apparatus was placed under a dynamic vacuum (1x10^{-3} mbar) and sealed, leaving the apparatus under static vacuum. The LN_2 dewar (–196 °C) was removed from bulb *B* and immediately transferred to bulb *A* such that the frozen solvent in bulb *B* could thaw and distil onto the frozen solids in bulb *A*. After the distillation had concluded, the apparatus was connected to an ampoule containing $^{15}N_2$ gas (dried over activated 4 Å molecular sieves for one week) via a three-way connector and bulb *A* was placed under a $^{15}N_2$ atmosphere. The apparatus was sealed and allowed to warm to room temperature. For reactions

involving MC_8 (M = K, Rb, Cs) as the reductant, the reaction mixture was stirred for 17 hours. In the case of the alkali metals, the reaction mixtures were subsequently sonicated until the metal was finely dispersed (1-40 minutes), and the reactions were stirred for 72 hours. Subsequent distillation and analysis were performed analogously to that of the $^{14}N_2$ procedure (**Supplementary Figures 41, 57, 59, 60, 80-81, 106-107**).

Acidification of [{U^{IV}(Tren^{DMBS})}$_2$(μ-η^2:η^2-$^{14}N_2$)] under $^{14}N_2$ atmosphere using $NaNO_3$ or $NaNO_2$. Under an atmosphere of argon, bulb *A* was charged with ^{14}N-isotopically labelled catalyst, **1** (0.0052 mol), the acid and reductant (600 equivalents), and either $NaNO_3$ or $NaNO_2$ (0.004 g/0.003 g, 0.0052 mmol, 1 equivalent) along with a glass-coated stirrer bar. Bulb *A* was then sealed and transferred to a Schlenk line. Et_2O (stored under argon, 4 or 8 mL, *vide infra*) was added to bulb *B* via syringe and then bulb *B* was submerged in a LN_2 dewar (–196 °C) and frozen. The entire apparatus was placed under a dynamic vacuum ($1x10^{-3}$ mbar) and sealed, leaving the apparatus under static vacuum. The LN_2 dewar (–196 °C) was removed from bulb *B* and immediately transferred to bulb *A* such that the frozen solvent in bulb *B* could thaw and distil onto the frozen solids in bulb *A*. After the distillation had concluded, the apparatus was placed under a dinitrogen atmosphere, sealed, and allowed to warm to room temperature. The apparatus was sealed and allowed to warm to room temperature. For reactions involving MC_8 (M = K, Rb, Cs) as the reductant, the reaction mixture was stirred for 17 hours. Subsequent distillation and analysis were performed analogously to that of the original $^{14}N_2$ procedure (**Supplementary Figures 63-66, 108-115).**

Computational Details

General

Unrestricted geometry optimisations for **1**, the anion component of **2-crypt** (**2'**), **2-Li**, **2-K**, and **3** were performed using coordinates derived from their crystal structures as the starting points. No constraints were imposed on the structures during the geometry optimisations. Due to the disorder of the lithium cations in **3** we computed two potential isomers, an end-on/side on isomer (**3A**, as shown in Figure X) and also an end-on/end-on isomer. (**3B**) We note that the N_2 unit in **3** is disordered over two sites meaning there are potentially four isomers in the crystal structure, but they are equivalent end-on/side-on and end-on/end-on pairs so only one example of each was computed. The calculations were performed using the Amsterdam Density Functional (ADF) suite version 2017 with standard convergence criteria.[6,7] The DFT geometry optimisations employed Slater type orbital (STO) triple-ζ-plus polarisation all-electron basis sets (from the Dirac and ZORA/TZP database of the ADF suite). Scalar relativistic approaches (spin-orbit neglected) were used within the ZORA Hamiltonian[8-10] for the inclusion of relativistic effects and the local density approximation (LDA) with the correlation potential due to Vosko *et al* was used in all of the calculations.[11] Generalised gradient approximation (GGA) corrections were performed using the functionals of Becke and Perdew.[12,13] Analytical frequency calculations were carried out within the ADF program. The Quantum Theory of Atoms in Molecules analysis[14,15] was carried out within the ADF program. We quote Nalewajski-Mrozek bond orders since they reproduce expected bond multiplicities reliably in polar heavy atom structures whereas Mayer bond orders for polar bonds often do not conform with chemical intuition.[16] The ADF-GUI (ADFview) was used to prepare the three-dimensional plots of the electron density. In all cases, Aufbau 'high-spin' formulations were found.

Crystallographic Details

General

X-ray crystallography data for the analysed crystal structures were collected using Cu*K*α radiation on a Rigaku SuperNova dual system in combination with Atlas (**2-crypt, 2-Li, 3** and **5**) and Atlas S2 type CCD detector (**4**) or an XtaLAB Synergy R, DW system, HyPix- Arc 150 diffractometer (for **2-K**), operating at T = 140.01(10) K (for **2-K** and **4**), T = 199.99(10) K (for **2-crypt**) and T = 200.00(10) K (for **2-Li, 3** and **5**).

Clear intense brown prism-shaped crystals of **2-crypt** (dimensions 0.20 × 0.07 × 0.06 mm^3), clear light green prism-shaped crystals of **2-K** (dimensions 0.10 × 0.02 × 0.01mm^3), clear intense brown plate-shaped crystals of **2-Li** (dimensions 0.07 × 0.06 × 0.05 mm^3), clear dark red prism-shaped crystals of **3** (dimensions 0.65×0.10×0.06 mm^3), clear dark orange plate-shaped crystals of **4** (dimensions 0.28 × 0.23 × 0.11 mm^3) and clear light brown plate-shaped crystals of **5** (dimensions 0.13 × 0.06 × 0.05 mm^3) were used as supplied.

The following data reduction and correction were carried out by *CrysAlis*^{Pro}.[17] The solutions and refinements were performed by *SHELXT* and *SHELXL* [18, 19] respectively. The crystal structures were refined using full-matrix least-squares based on $\boldsymbol{F^2}$ with all non-H atoms defined in anisotropic manner. Hydrogen atoms were placed in calculated positions by means of the "riding" model.

Response to B alerts in CIF files (no A alerts)

Complex 2-crypt (lc-b-55)
Alert level B
PLAT972_ALERT_2_B Check Calcd Resid. Dens. 0.82Ang From U2: -2.62 eA^{-3}
Author Response: The residual density is due to absorption artefacts
Complex 2-K (MB470)
PLAT097_ALERT_2_B Large Reported Max. Positive) Residual Density: 14.75 eA^{-3}
Author Response: A high residual density might indeed be due to unaccounted for twinning. All our attempts failed to improve the model. Crystal quality and diffraction pattern were very messy. Compounds of this type are very difficult to grow crystals of good quality. We were able to obtain a highly reliable model and good value for Rint despite this.
PLAT342_ALERT_3_B Low Bond Precision on C---C Bond : 0.02708 Ang.

Author Response: Crystal quality and diffraction pattern were very messy. Compounds of this type are very difficult to grow crystals of good quality. We were able to obtain a highly reliable model and good value for Rint despite this.
Complex 2-Li (lc-b-39)
PLAT972_ALERT_2_B Check Calcd Resid. Dens. 0.78Ang From U1: -2.59 eA^{-3}
Author Response: this is very likely due to absorption artifacts
Complex 3 (MB541)
PLAT342_ALERT_3_B Low Bond Precision on C---C Bond: 0.02583 Ang.
Author Response: This is, very likely, due to the extensive disorder affecting the structure
Complex 4 (mb538-b)
PLAT342_ALERT_3_B Low Bond Precision on C---C Bond: 0.02371 Ang.
Author Response: This is, very likely, due to twinning issues
Complex 5 (mb591)
PLAT342_ALERT_3_B Low Bond Precision on C---C Bond: 0.023625 Ang.
Author Response: The diffraction pattern of the crystal was rather weak and could explain this issue
PLAT410_ALERT_2_B Short Intra H...H Contact H3A… H6A 1.88 Ang. x,y,z = 1_555 Check
Author Response: The H's are at calculated position and there is no clear sign of disorder in the structure. There is then no easy explanation for this.
PLAT971_ALERT_2_B Check Calcd Resid. Dens. 0.87 Ang From U2 : 2.80 eA^{-3}
Author Response: This is due to absorption effects
PLAT971_ALERT_2_B Check Calcd Resid. Dens. 1.03Ang From U1 2.66 eA^{-3}
Author Response: This is due to absorption effects

Structure-specific

Description of 2-crypt: The N1-N2 distance of 1.336(6) Å in **2-crypt** is significantly longer compared to that found in **1** (1.109(7) Å)[1b], the latter of which has an N=N distance close to that of the free N_2 (1.0975 Å) (Supplementary Table 1). The longer N-N distance in **2-crypt** indicates reduction at the central N_2 rather than at the uranium ions, and the U1-N_2-U2 unit is bent (fold angle of 157.43°, Figure 3, g) compared to the essentially planar angle of 177.83° in **1**. The U-N_{amide} distances for **2-crypt** (av. 2.238 Å) are close to those of [U^{IV}($Tren^{DMBS}$)Cl] (av. 2.22 Å),[1a] while the U-N_{amine} distance (av. 2.743 Å) is longer than in [U^{IV}($Tren^{DMBS}$)Cl] (2.656(19) Å).

Description of 2-K: The solid-state structure of **2-K** features a dinuclear U_2-N_2 $Tren^{DMBS}$ assembly (one per asymmetric unit) with a single inner-sphere K-ion bound to the central N_2 (Figure 3, c; Supplementary Figure 3). Similarly to **2-crypt**, the U1-N_2-U2 unit is folded (∠ = 158.1(12)°, Figure 3, g), but the poor precision of N1-N2 distance (1.364(18) Å) precludes a detailed analysis of the impact of the coordinated K-ion and its surrounding bonding (K1-N2 = 2.58(7) Å; K1-N_{amide} = 2.938(12) and 2.968(9) Å). The average U-N_{amido} distance of 2.332 Å is similar to the one found in **2-crypt** (2.238 Å), while the U-N_{amine} distance (av. 2.670 Å) is closer to that found in the mononuclear [U^{IV}($Tren^{DMBS}$)Cl] (2.656(19) Å).

Description of 2-Li: The molecular structure of **2-Li** consists of a single U(IV)/U(IV) complex per asymmetric unit, with a Li-cation closely bound to the bridging N_2 and two amide arms (Li1-N2 = 1.92(2) Å, Li1-N_{amide} = 2.30(2) Å and 2.34(2) Å), and contrary to **2-crypt** and **2-K** the U1-N_2-U2 unit is planar (Figure 3, b; g; Supplementary Figure 4). The average U-N_{amide} and U-N_{amine} distances (2.319 Å and 2.653 Å, respectively), are similar to those found in other complexes in the series and match the values expected for U(IV)-bound species (Supplementary Table 1).

Description of 3: The solid-state structure of **3** shows the presence of a central hydrazido(4–) moiety bridging two U-ions disordered over two orientations (Figure 3, d; Supplementary Figures 5-6). The N-N bond (Figure 3, g) is significantly elongated compared to **1** and **2-crypt** and is within the same range for both orientations (N1-N2 = 1.483(19); N3-N4 = 1.47(2) Å), and essentially the same as free hydrazine (N-N = 1.47 Å).

Description of 4: The solid-state structure of **4** shows two U-centres bridged by two nitrides (N6 and N3) at a non-bonding N···N distance of 2.70(1) Å in a diamond core U_2N_2 motif (Figure 3, g) with U-N bond distances of U2-N3 = 2.248(9) Å and U2-N6 = 2.184(9) Å (Supplementary Figure 7, Supplementary Table 1). Each nitride binds two Li-cations, with two different binding modes: the Li-cations interacting with N6 are coordinatively supported by two U-bound amide arms (Li1-N6 = 1.96(2); Li2-N6 = 1.98(2) Å), while the two cations capping the second nitride are held by one $Tren^{DMBS}$ arm that is dissociated from U (Li3-N3 = 2.16(3); Li3-N3 = 1.97(2) Å). The U-nitride distances in **4** are slightly longer than those reported for the U^V/U^V nitrides

$[K_2\{U^V(OSi(O^tBu)_3)_3(\mu\text{-}N)\}_2]$ (U^V-$N_{nitride}$ = 2.022(5) Å, 2.101(6) Å), $[\{U^{VI}(OSi(O^tBu)_3)_3\}_2(\mu\text{-}N)_2(\mu\text{-}THF)]$ (U^{VI}-$N_{nitride}$ = 1.850(12) Å and 2.252(16) Å) and mixed-valent U(V)/U(VI) $[K\{U(OSi(O^tBu)_3)_3(\mu\text{-}N)\}_2]$ ($U^{V/VI}$-$N_{nitride}$ = 2.067(4) Å and 2.020(4) Å). [20-22]

The closely related diuranium(IV) bis-nitride complex $[\{U^{IV}(\mu\text{-}NLi_2)(Tren^{TIPS})\}_2]$ was generated by reduction of the azide precursor $[U^{IV}(N_3)(Tren^{TIPS})]$ with excess Li-metal.[23] It bears slightly more sterically demanding Si^iPr_3 amide substituents, and due to the steric congestion one arm of each $Tren^{TIPS}$ ligand does not bind the U-centre to accommodate formation of the **4**.

Description of 5: The solid-state structure of 5 (Figure 3, f) reveals an asymmetric diamond-shape U_2-N_2 core (Figure 3, g) with disparate U-$N_{nitride}$ distances (U2-N5 = 2.35(1); U1-N5 = 2.02(1) Å) (Supplementary Figure 8). Loss of one Li-cation is observed following the protonation of the N6 nitrido to give an imido-group (U2-N6 = 2.21(1); U1-N6 = 2.14(1) Å). One Li-cation has migrated to the upper side of the $Tren^{DMBS}$ assembly, and now all three remaining Li-cations reside in close proximity to the N5 nitride (average N5-Li = 2.038 Å) with Li1 and Li3 supported by the de-coordinated $Tren^{DMBS}$ arm, similarly to the binding motif in **4**. Additionally, both triamidoamine ligands adopt a *cis*- orientation with regards to the remaining nitride linkage (N7-U2-N1 ∠ = 108.2º, N3-U1-N5 ∠ = 96.3º). U-N_{imide} distances are on average longer than U-$N_{nitride}$ in **4** and compare well with other U(IV)-supported bridging imido complexes, for example $[K_2\{U^{IV}(OSi(O^tBu)_3)_3(\mu\text{-}NH)\}_2]$ (U-N_{imide} = 2.192(3)–2.273(3) Å)[22] and $[\{U^{IV}(Tren^{TIPS})\}_2(\mu\text{-}NH)(\mu\text{-}NLi_2)Li]$ (U-N_{imide} = 2.122(4) Å).[23] Nonetheless, the U-$N_{nitride}$ distance (U2-N5 = 2.35(1) Å) is elongated compared to the longest U2-N3 = 2.25(1) Å in **4**, which is most probably associated with steric and electrostatic effects exerted on the N3 vertex by three closely-coordinated Li-cations (Supplementary Table 1).

SUPPLEMENTARY FIGURES

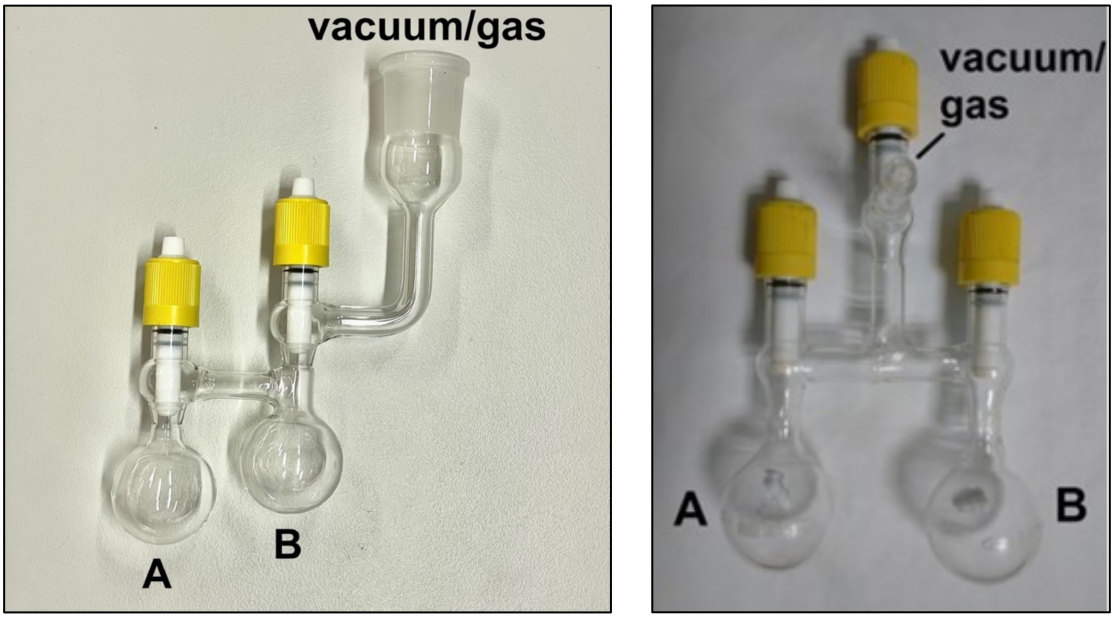

Supplementary Figure 1. Distillation apparatus used to perform catalytic trials.

Solid-state structures

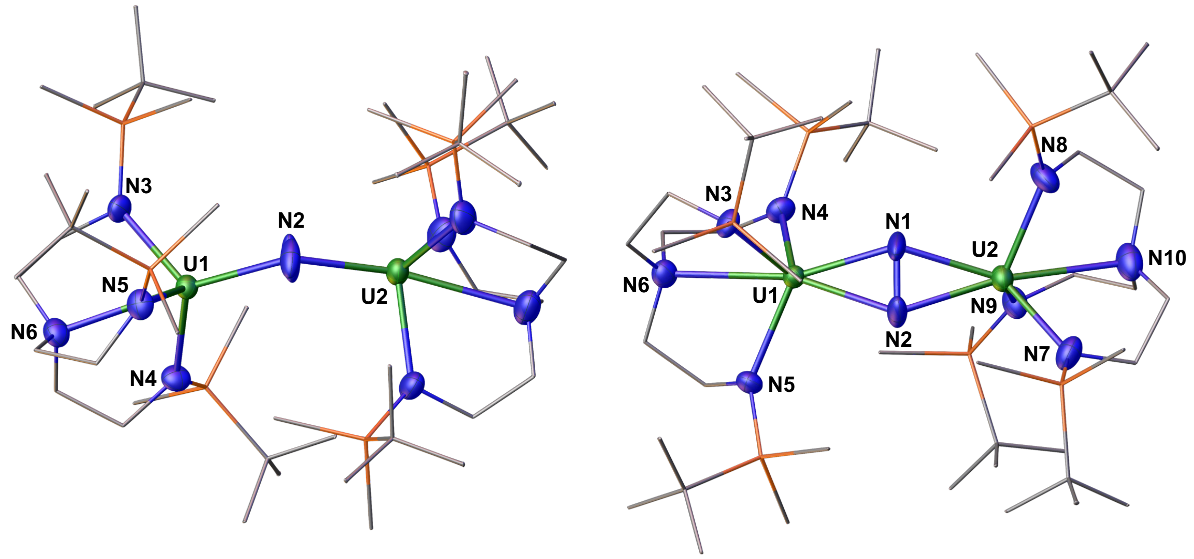

Supplementary Figure 2. Molecular structure of the anionic unit in **2-crypt**, with thermal ellipsoids drawn at the 50% probability level. Triamidoamine ligand framework was depicted as wireframe, and hydrogen atoms as well as disordered tert-butyl groups were removed for clarity. Two representations (along the N-N unit axis and perpendicular to it) are presented.

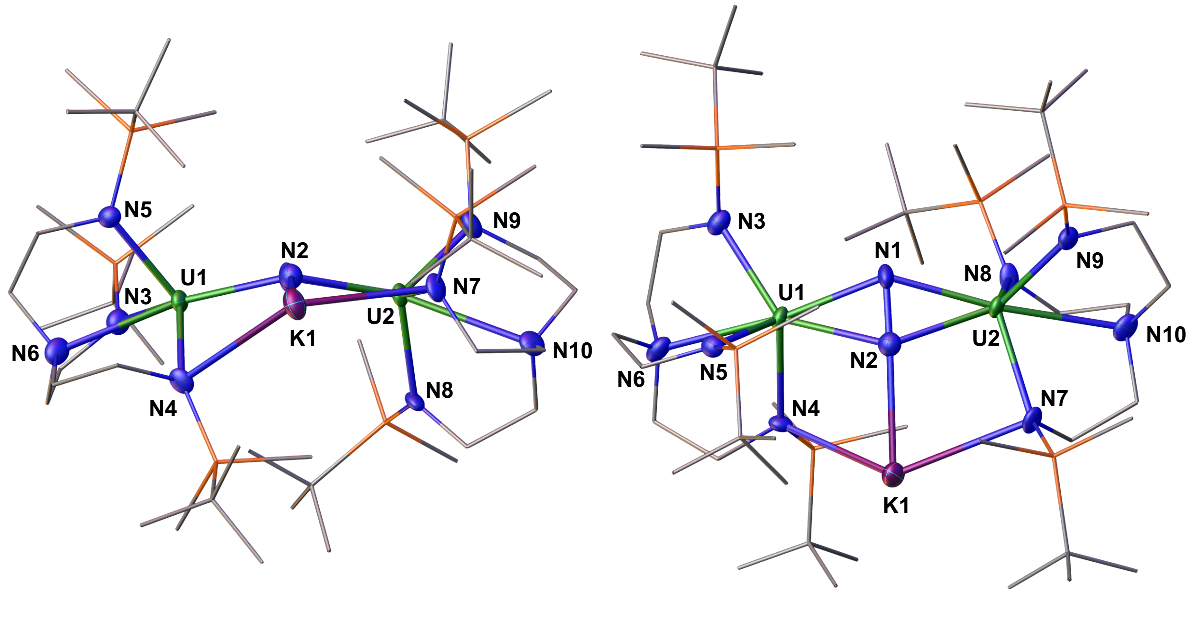

Supplementary Figure 3. Molecular structure of **2-K**, with thermal ellipsoids drawn at the 50% probability level. Triamidoamine ligand framework was depicted as wireframe, and hydrogen atoms as well as disordered tert-butyl groups were removed for clarity. Two representations (along the N-N unit axis and perpendicular to it) are presented.

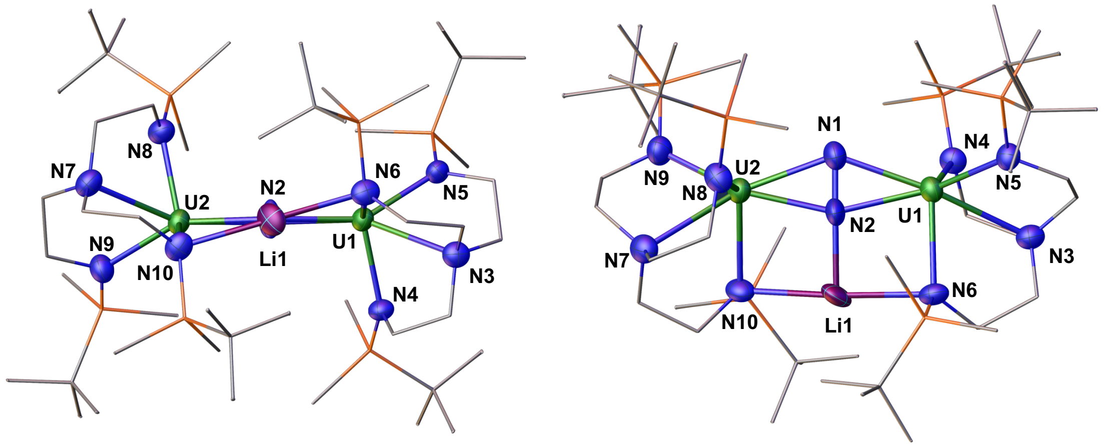

Supplementary Figure 4. Molecular structure of **2-Li**, with thermal ellipsoids drawn at the 50% probability level. Triamidoamine ligand framework was depicted as wireframe, and hydrogen atoms as well as disordered tert-butyl groups were removed for clarity. Two representations (along the N-N unit axis and perpendicular to it) are presented.

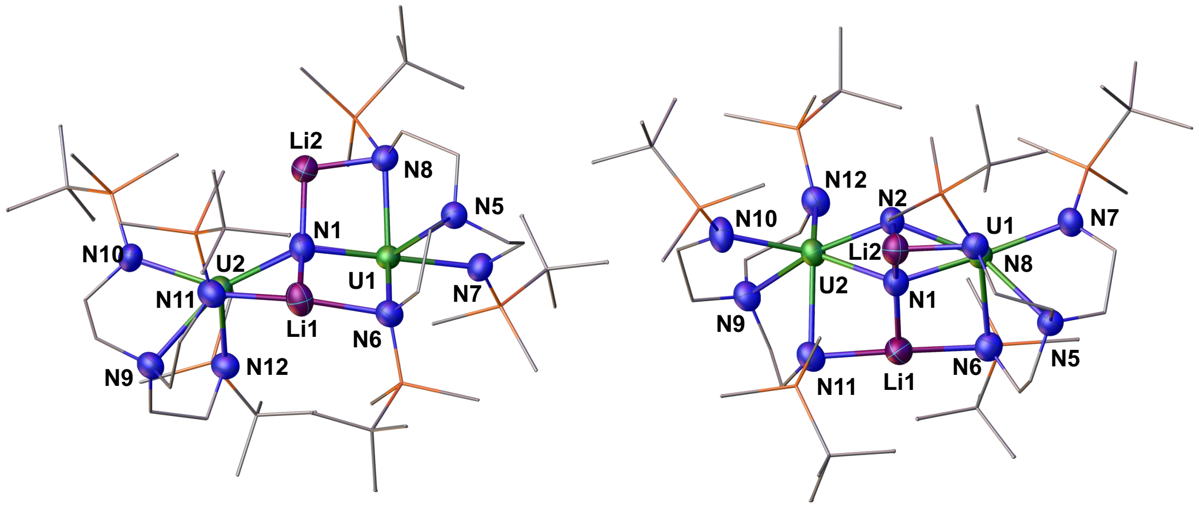

Supplementary Figure 5. Molecular structure of **3**, with thermal ellipsoids drawn at the 50% probability level. Triamidoamine ligand framework was depicted as wireframe, and hydrogen atoms as well as disordered tert-butyl groups were removed for clarity. The second component of the dinitrogen disorder and the disordered lithium were removed for clarity. Two representations (along the N-N unit axis and perpendicular to it) are presented.

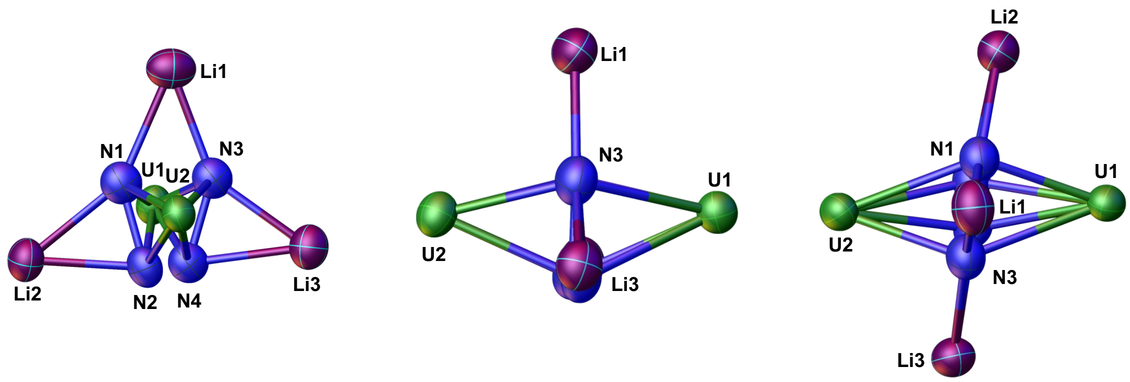

Supplementary Figure 6. Molecular structure of the core of **3**, with thermal ellipsoids drawn at the 50% probability level and depicted disorder (Li3, N3-N4). Triamidoamine ligand framework, hydrogen atoms as well as disordered tert-butyl groups were all removed for clarity. Three representations are presented.

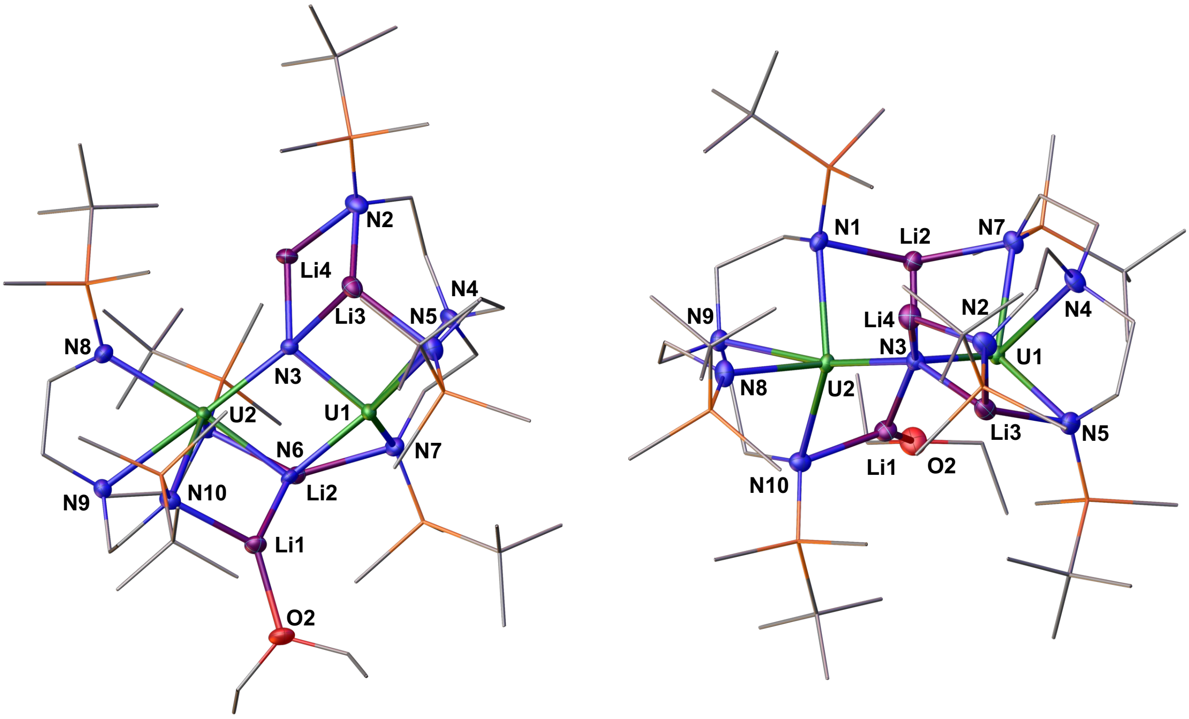

Supplementary Figure 7. Molecular structure of **4**, with thermal ellipsoids drawn at the 50% probability level. Triamidoamine ligand framework was depicted as wireframe, and hydrogen atoms as well as disordered tert-butyl groups were removed for clarity. Two representations (along the axis of the nitride unit and perpendicular to it) are presented.

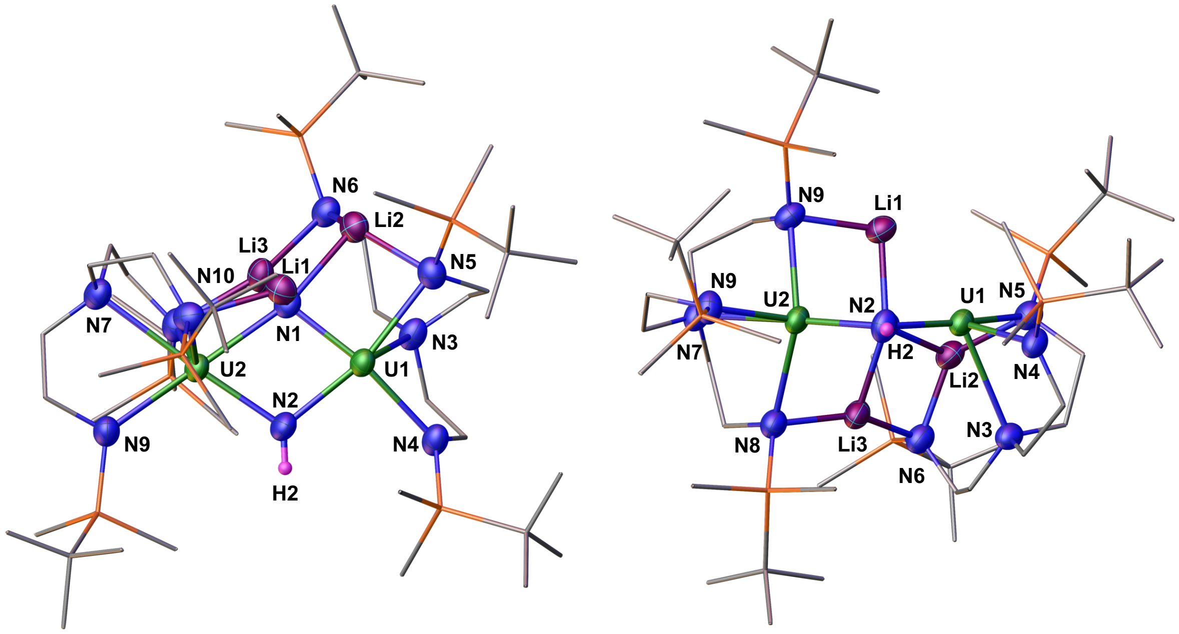

Supplementary Figure 8. Molecular structure of **5**, with thermal ellipsoids drawn at the 50% probability level. Triamidoamine ligand framework was depicted as wireframe, and hydrogen atoms as well as disordered tert-butyl groups were removed for clarity. Two representations (along the axis of the nitrido/imido unit and perpendicular to it) are presented.

NMR spectroscopy

Syntheses and reactivity studies

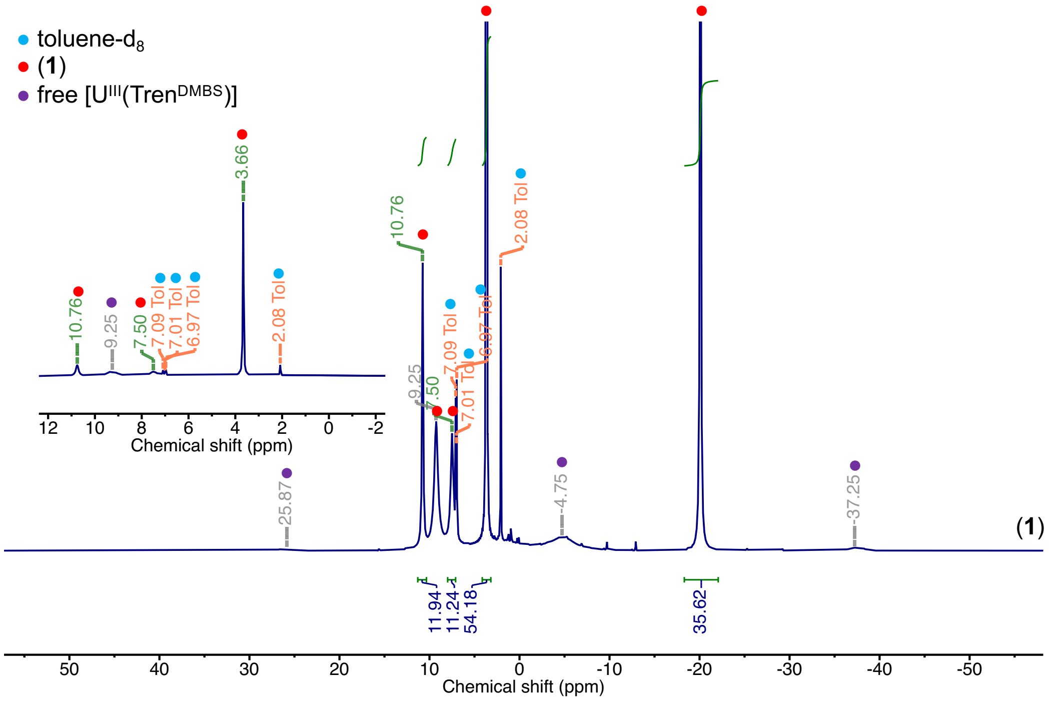

Supplementary Figure 9. ^{1}H NMR (400 MHz, toluene-d$_8$, 298 K) spectrum of isolated **1** under 1.01 bar absolute pressure of N_2.

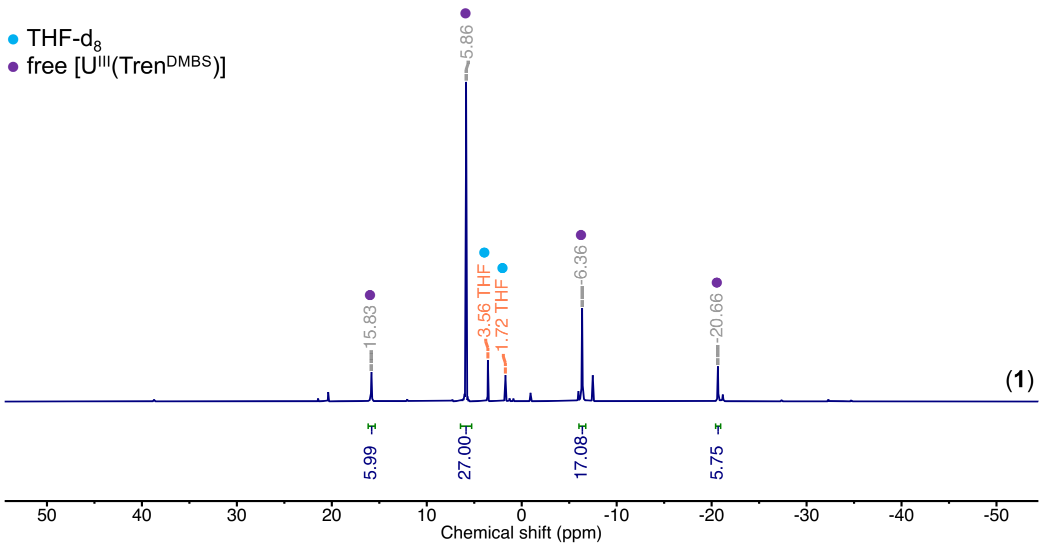

Supplementary Figure 10. ^{1}H NMR (400 MHz, THF-d$_8$, 298 K) spectrum of **1**.

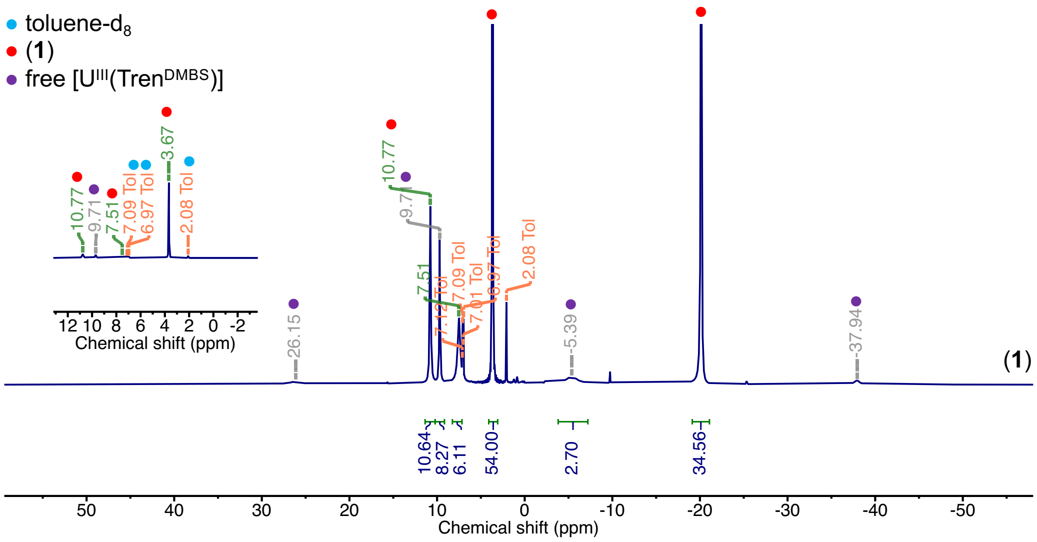

Supplementary Figure 11. ^{1}H NMR (400 MHz, tol-d$_8$, 298 K) spectrum obtained of **1** under 2.51 bar absolute pressure of N_2.

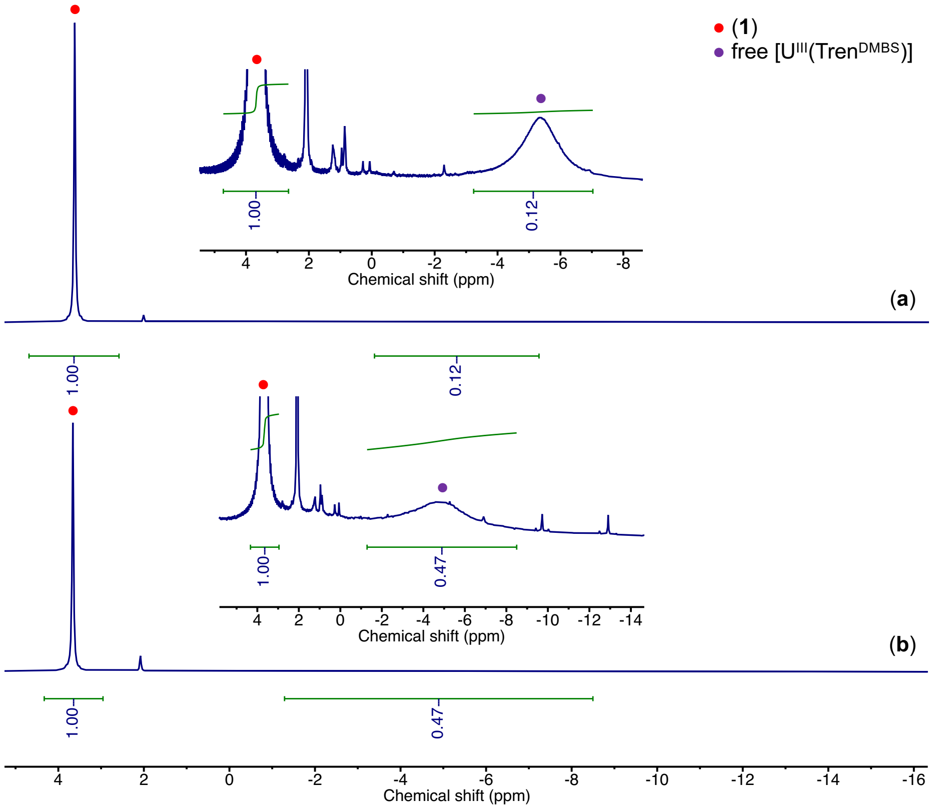

Supplementary Figure 12. Comparison of ^{1}H NMR (400 MHz, tol-d$_8$, 298 K) spectra of **1** under 2.51 bar (**a**) and 1.01 bar (**b**) absolute N_2 pressure.

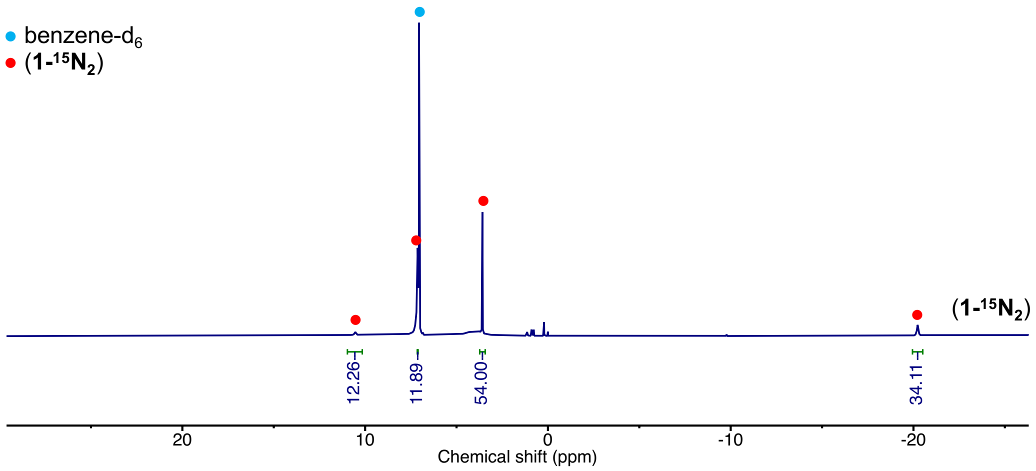

Supplementary Figure 13. ^{1}H NMR (400 MHz, benzene-d$_6$, 298 K) spectrum of **1-$^{15}N_2$**.

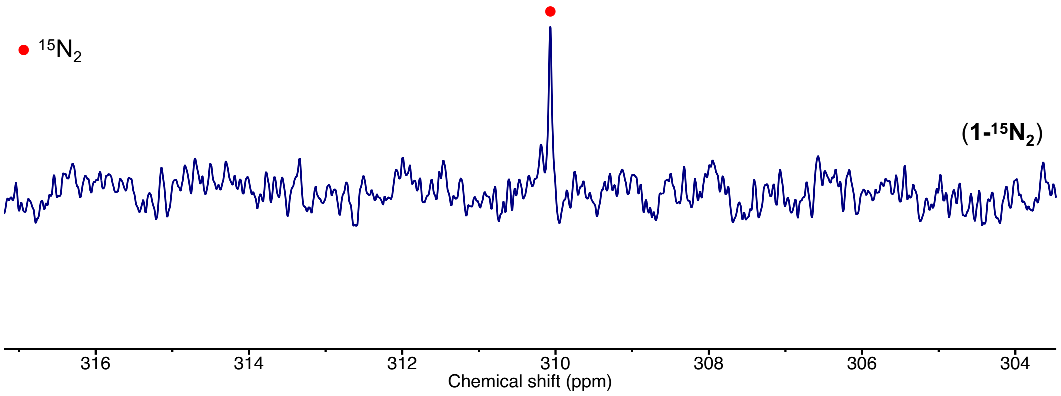

Supplementary Figure 14. ^{15}N NMR (70.97 MHz, benzene-d$_6$, 298 K) spectrum of **1-$^{15}N_2$** showing only the presence of dissociated $^{15}N_2$ at 310 ppm referenced to NH_3 at 0 ppm. No signal for **1-$^{15}N_2$** was identified over the examined (in 500 ppm steps) range of 6000 to –500 ppm, which we attribute to broadening caused by the paramagnetism of the uranium(IV) (5f^2) ions.

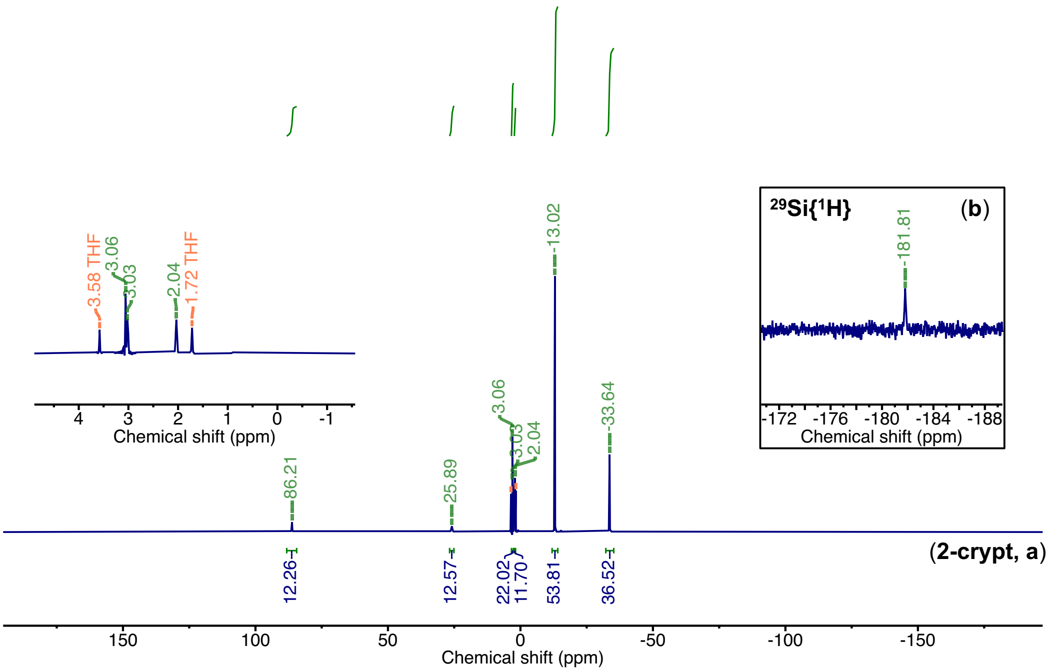

Supplementary Figure 15. ^{1}H NMR (400 MHz, THF-d$_8$, 298 K) spectrum of **2-crypt** (**a**), with $^{29}Si\{^{1}H\}$ NMR (79.5 MHz, THF-d$_8$, 298 K) spectrum as an inset (**b**).

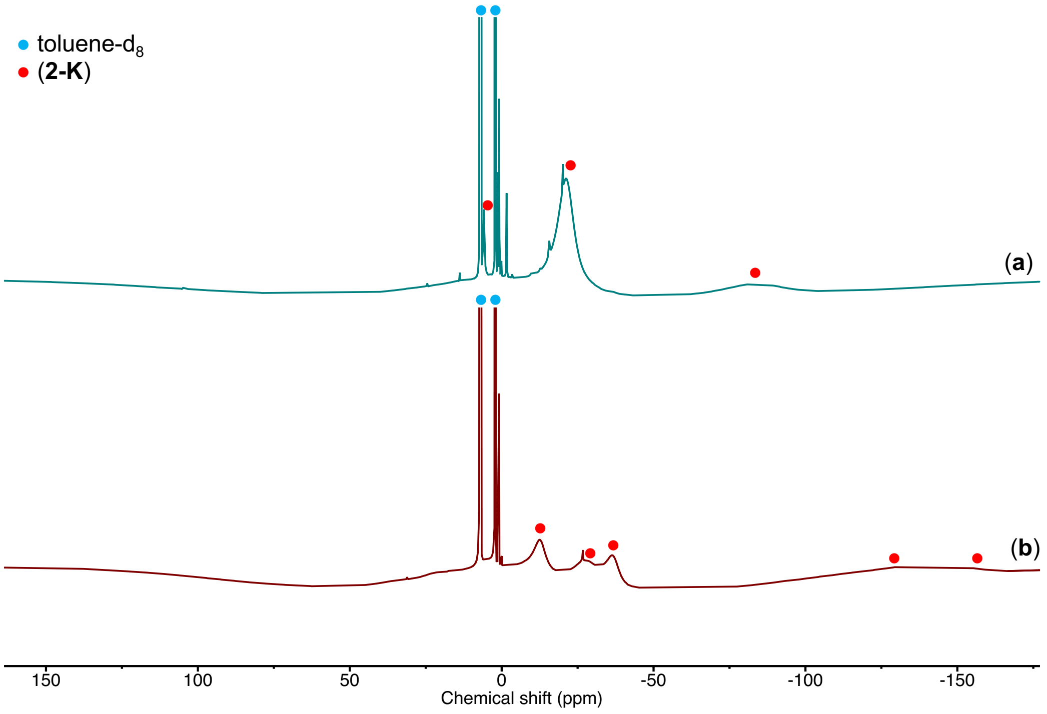

Supplementary Figure 16. Variable-temperature ^{1}H NMR (400 MHz, toluene-d$_8$) spectrum of **2-K** at 233 K (**a**) and 193 K (**b**).

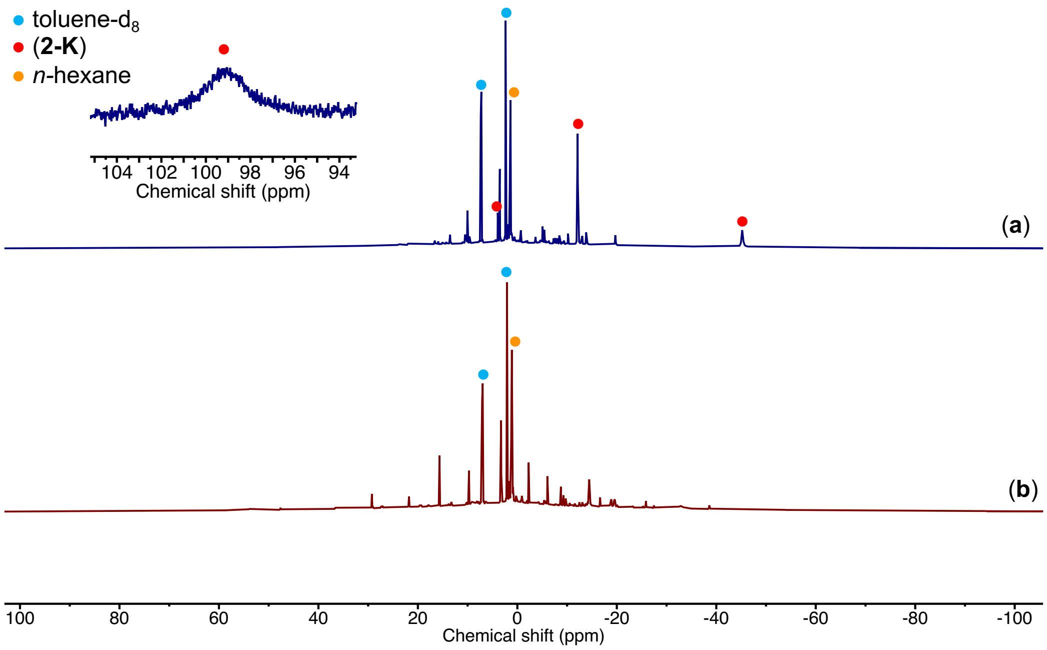

Supplementary Figure 17. ^{1}H NMR (400 MHz, toluene-d8, 298 K) spectrum obtained after dissolution of crystalline **2-K** in toluene-d8 at room temperature, immediately (**a**), and after 6 hours (**b**).

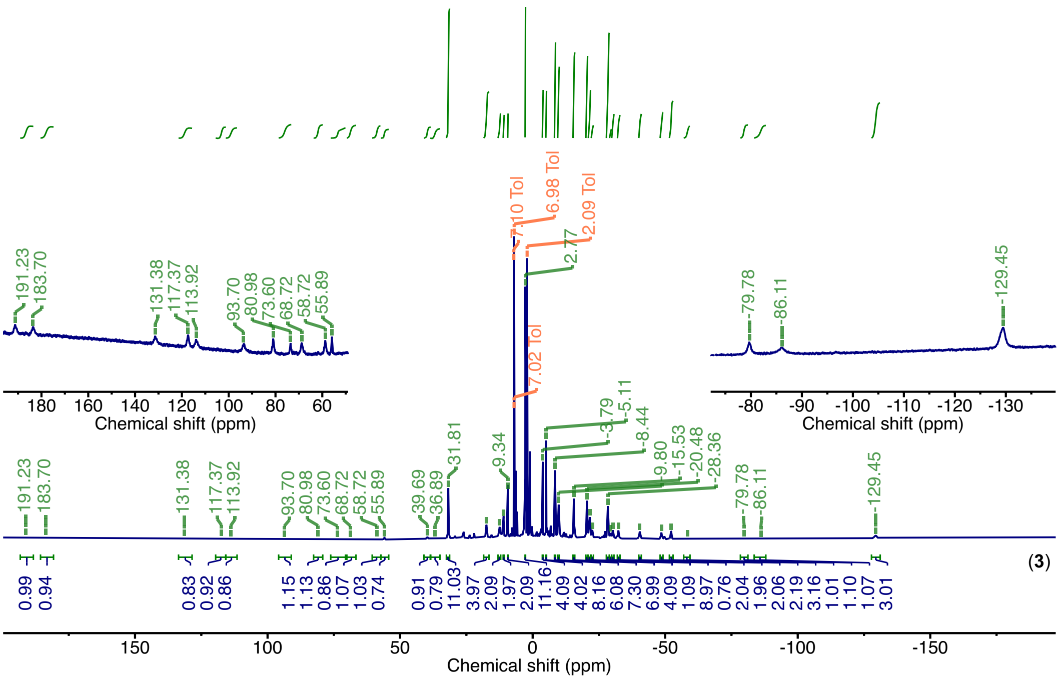

Supplementary Figure 18. ^{1}H NMR (400 MHz, toluene-d8, 298 K) spectrum of **3**.

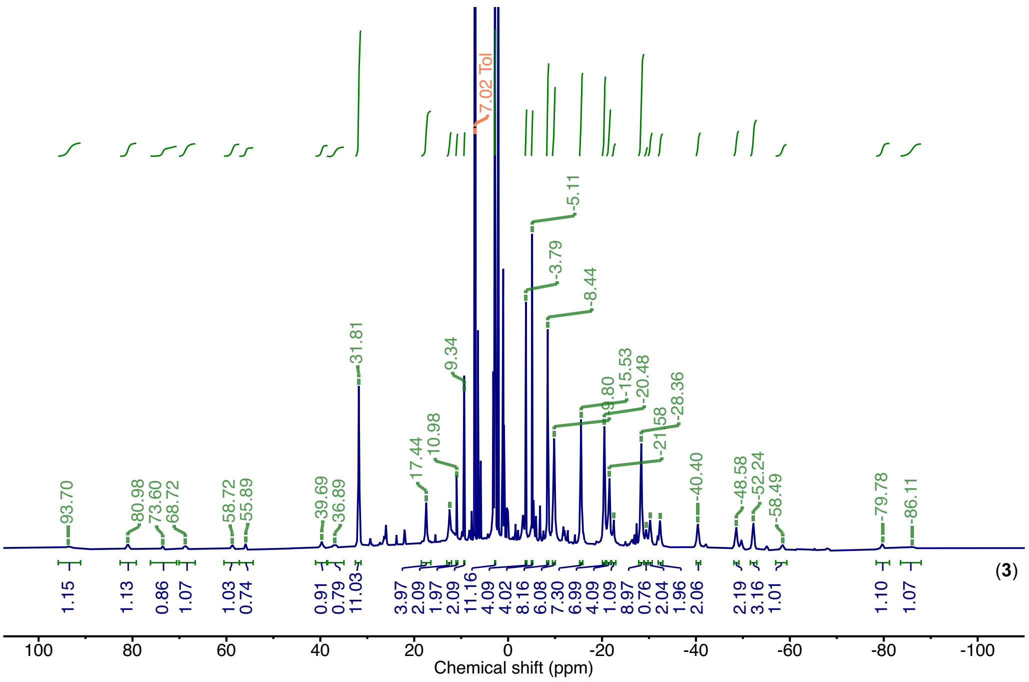

Supplementary Figure 19. Zoom in of ^{1}H NMR (400 MHz, toluene-d$_8$, 298 K) spectrum of **3** between 100 and –100 ppm.

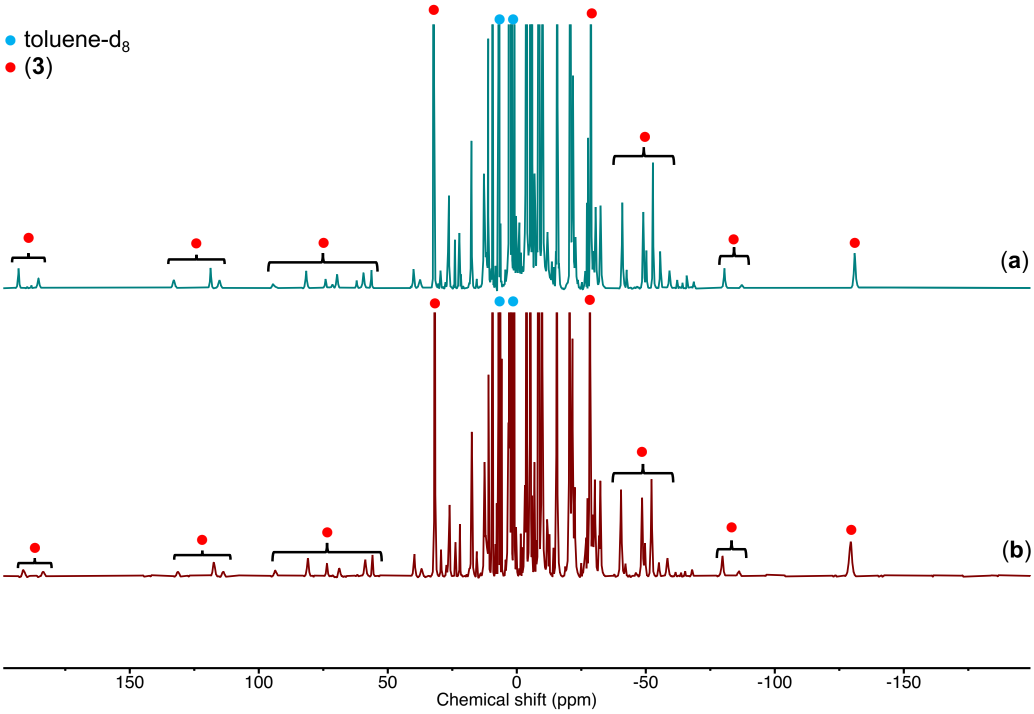

Supplementary Figure 20. ^{1}H NMR (400 MHz, toluene-d$_8$, 298 K) spectrum of the reaction mixture (**a**) obtained after reduction of **2-K** in diethyl ether with 1 eq. KC_8 + 2 eq. LiI giving **3** as the only identifiable product; spectrum of isolated **3** is given for comparison, **b**. Only the most prominent resonances are labelled for clarity.

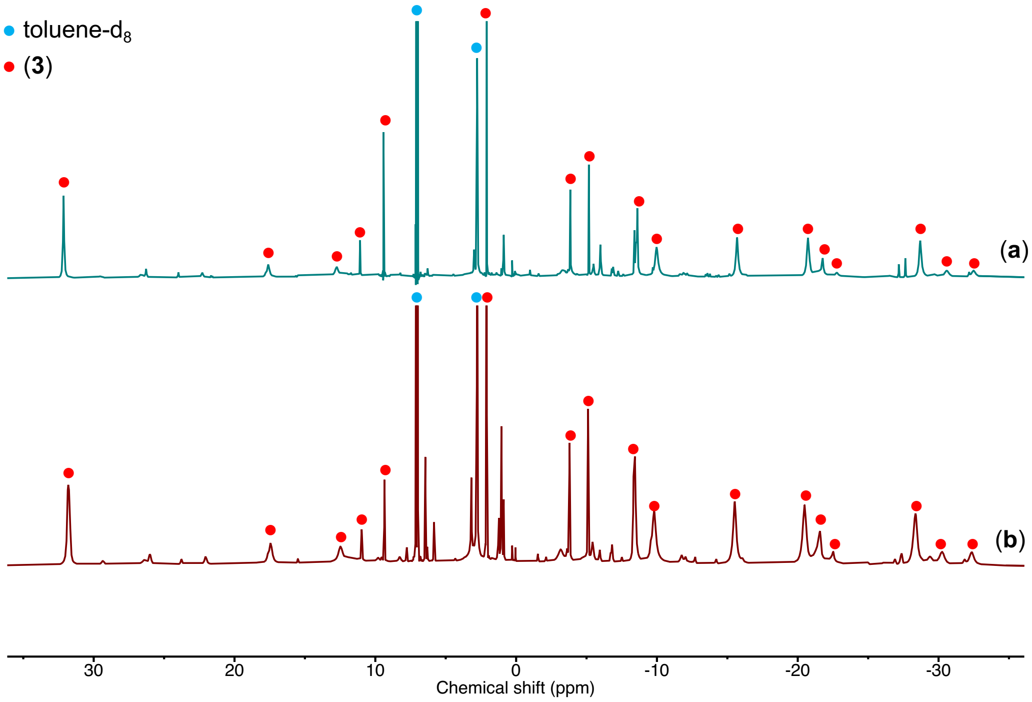

Supplementary Figure 21. ^{1}H NMR (400 MHz, toluene-d$_8$, 298 K) spectrum of the reaction mixture (**a**) obtained after reduction of **2-K** in diethyl ether with 1 eq. KC_8 + 2 eq. LiI giving **3** as the only identifiable product; spectrum of isolated **3** is given for comparison, **b**. Only the most prominent resonances are labelled for clarity. Zoom-in in the 30 ppm to –30 ppm range.

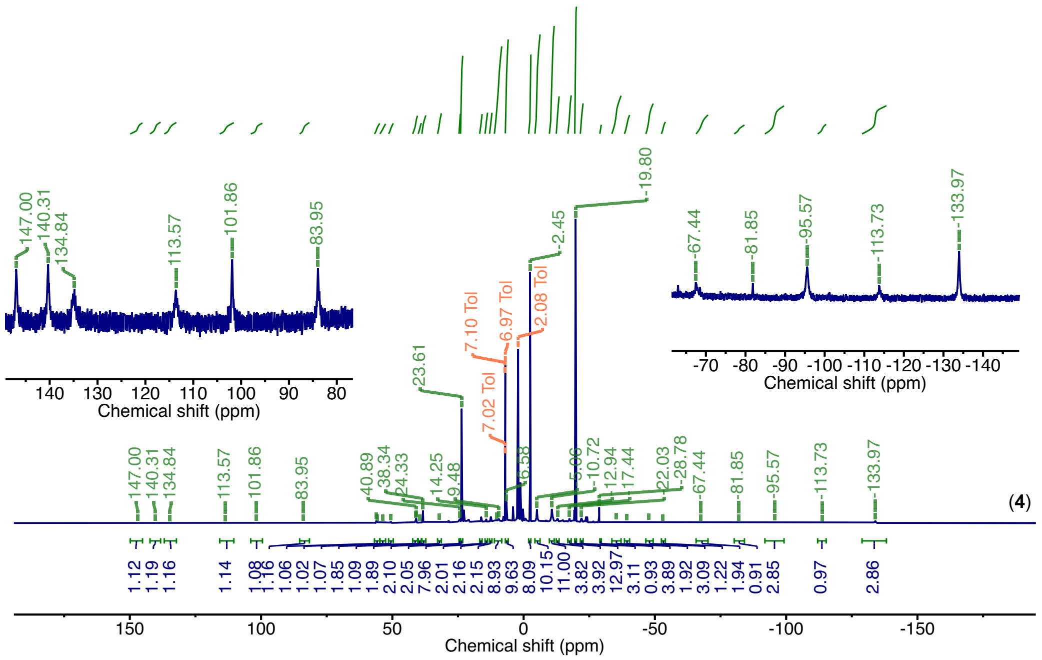

Supplementary Figure 22. ^{1}H NMR (400 MHz, toluene-d$_8$, 298 K) spectrum of **4**.

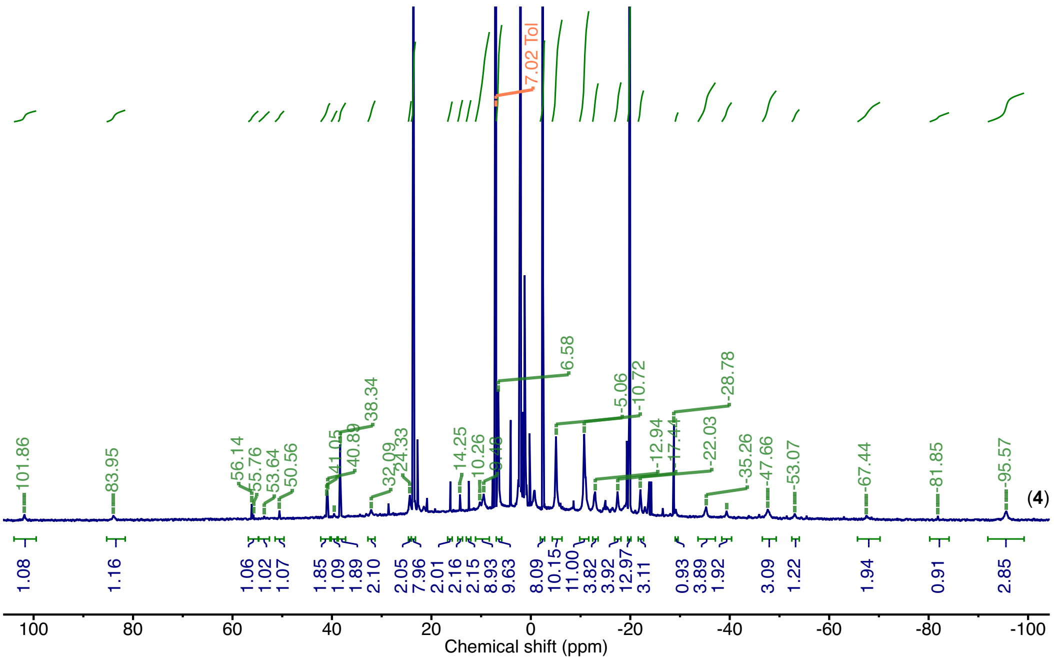

Supplementary Figure 23. Zoom in of ^{1}H NMR (400 MHz, toluene-d$_8$, 298 K) spectrum of **4** between 100 and –100 ppm.

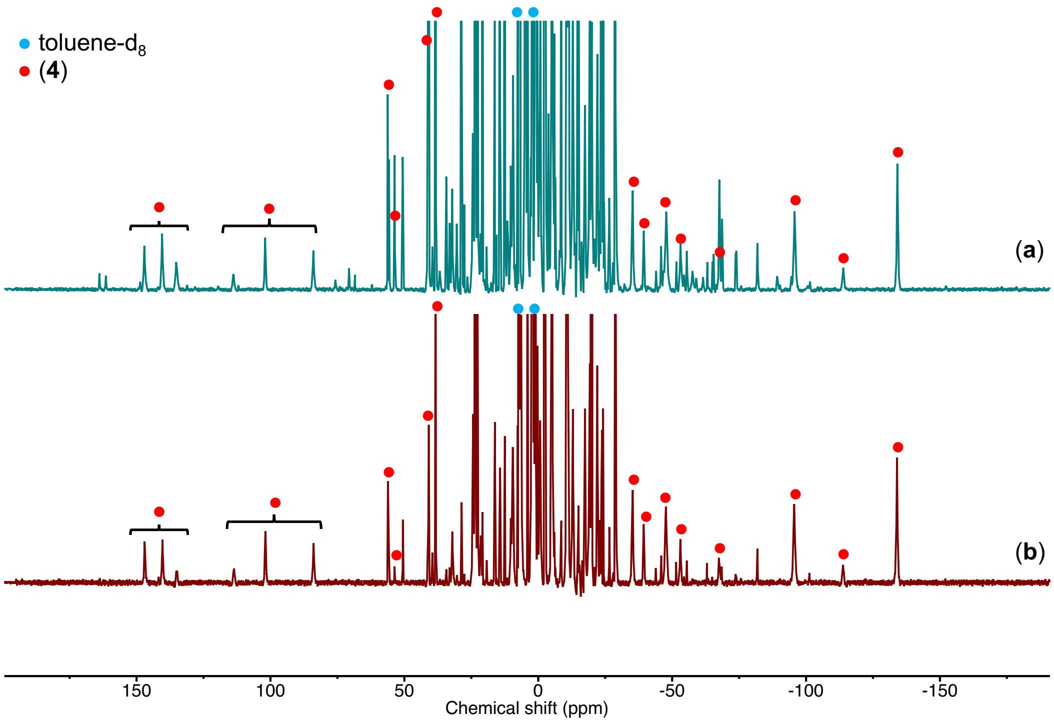

Supplementary Figure 24. ^{1}H NMR (400 MHz, toluene-d$_8$, 298 K) spectrum of the solid obtained from the reduction of **2-K** in diethyl ether with 3 eq. KC_8 + 4 eq. LiI giving **4** as the only identifiable species, **a**; spectrum of isolated **4** is given for comparison, **b**. Only the most prominent resonances are labelled for clarity.

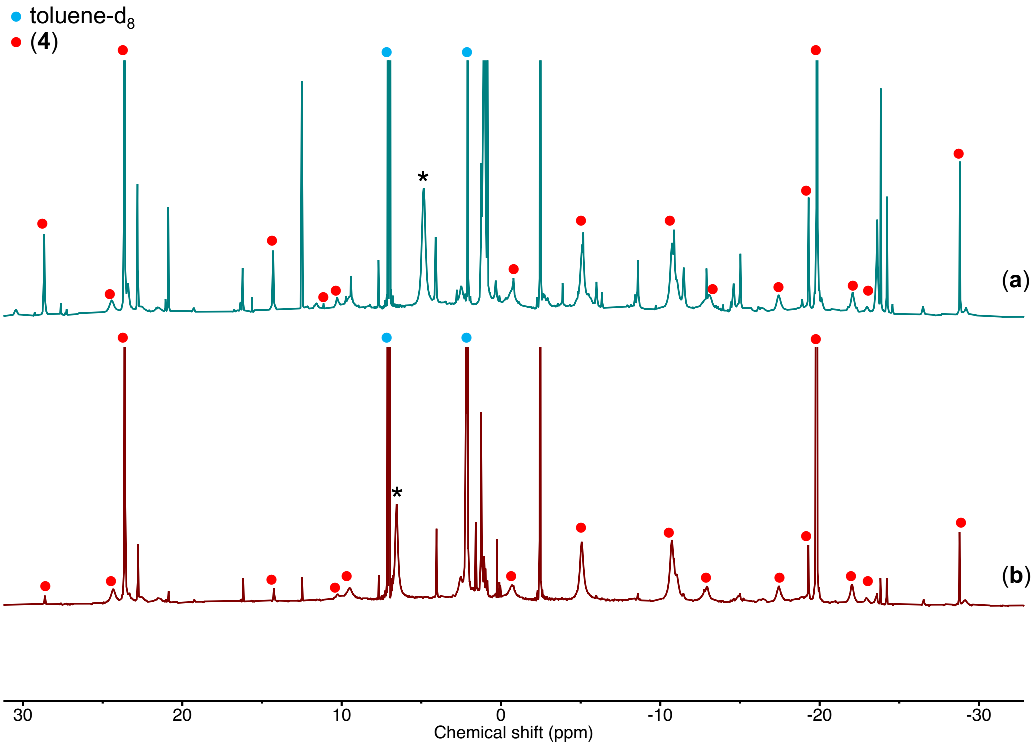

Supplementary Figure 25. ^{1}H NMR (400 MHz, toluene-d$_8$, 298 K) spectrum of the solid obtained from the reduction of **2-K** in diethyl ether with 3 eq. KC_8 + 4 eq. LiI giving **4** as the only identifiable species, **a**; spectrum of isolated **4** is given for comparison, **b**. Paramagnetically-shifted diethyl ether peak is labelled with an asterisk.

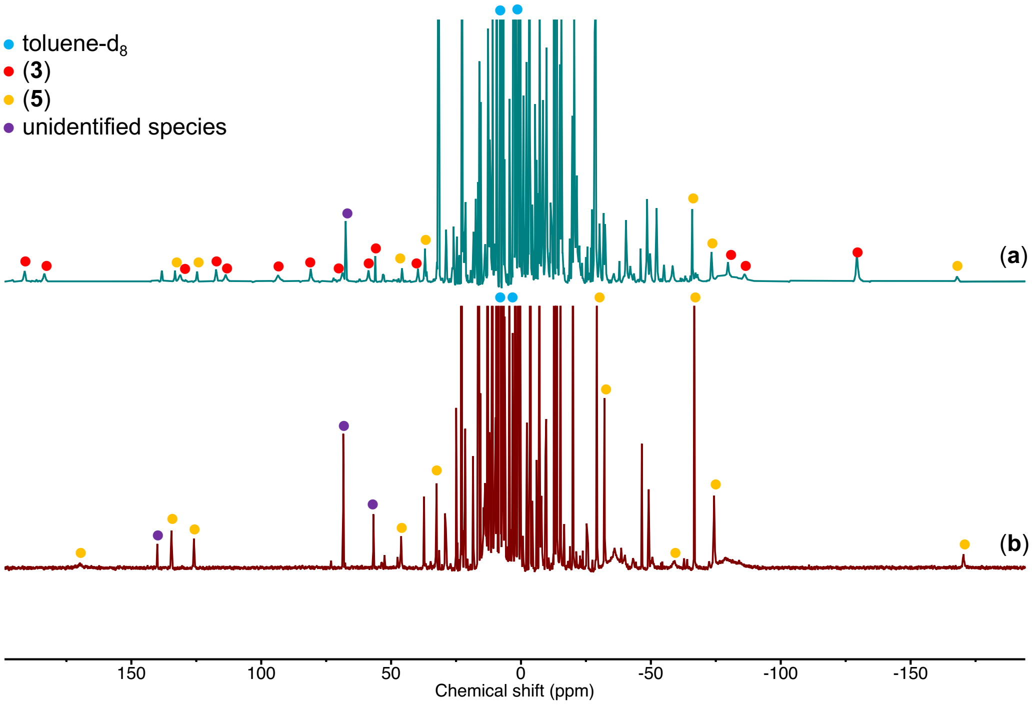

Supplementary Figure 26. ^{1}H NMR (400 MHz, toluene-d$_8$, 298 K) spectrum of the reaction mixture obtained after exposure of **3** to H_2 gas, immediately (**a**) and after 48 hours (**b**). Only the most prominent resonances are labelled for clarity.

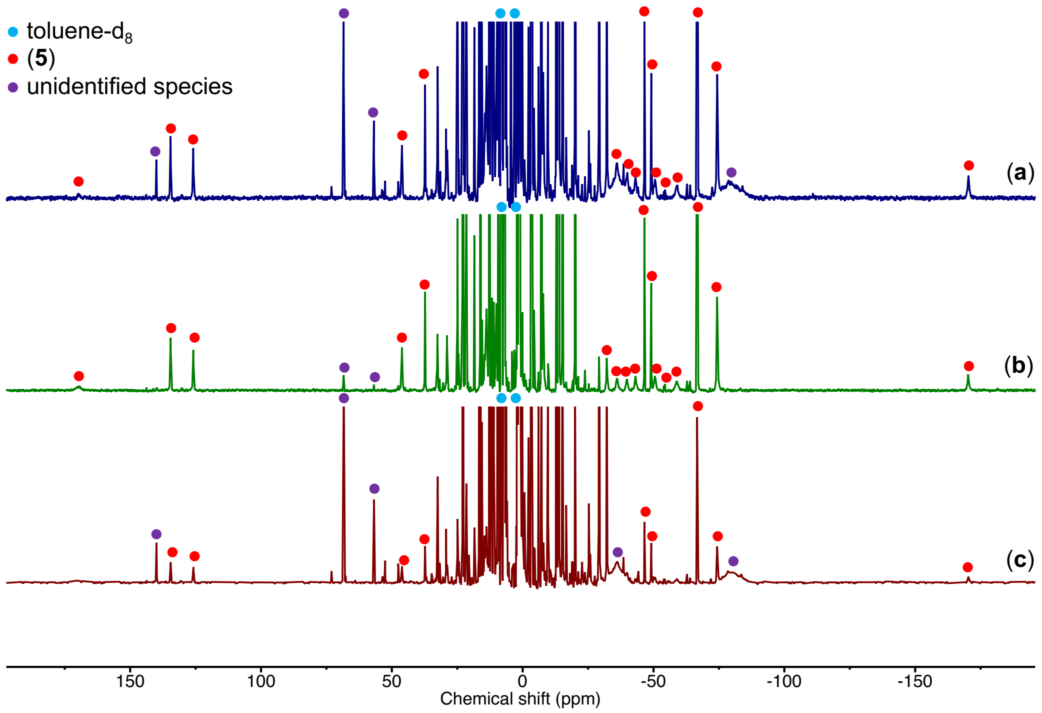

Supplementary Figure 27. Comparison of the ^{1}H NMR (400 MHz, toluene-d8, 298 K) spectra of the reaction mixture obtained after exposure of **3** to H_2 gas (**a**), crystals of **5** isolated from the reaction mixture (**b**) and the remaining mother liquor, indicating the presence of unidentified species in the solution (**c**). Only the most prominent resonances are labelled for clarity.

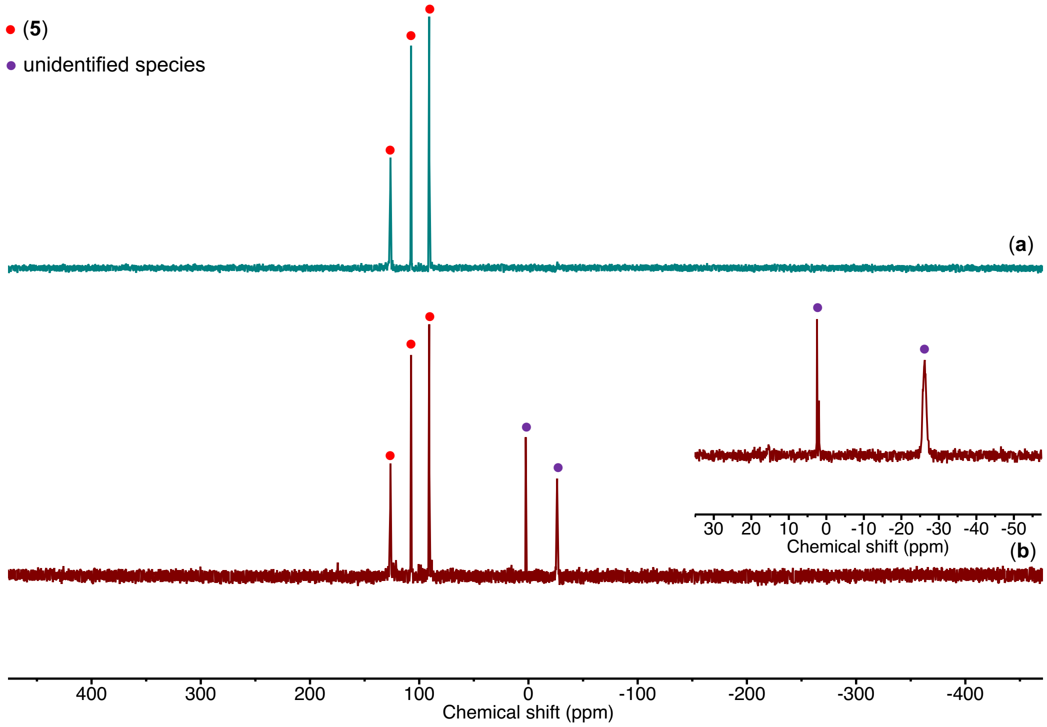

Supplementary Figure 28. Comparison of ^{7}Li NMR (156 MHz, toluene-d8, 298 K) spectra of the isolated crystals of **5** (**a**) and of the mother liquor containing unidentified lithium-ligated species (**b**).

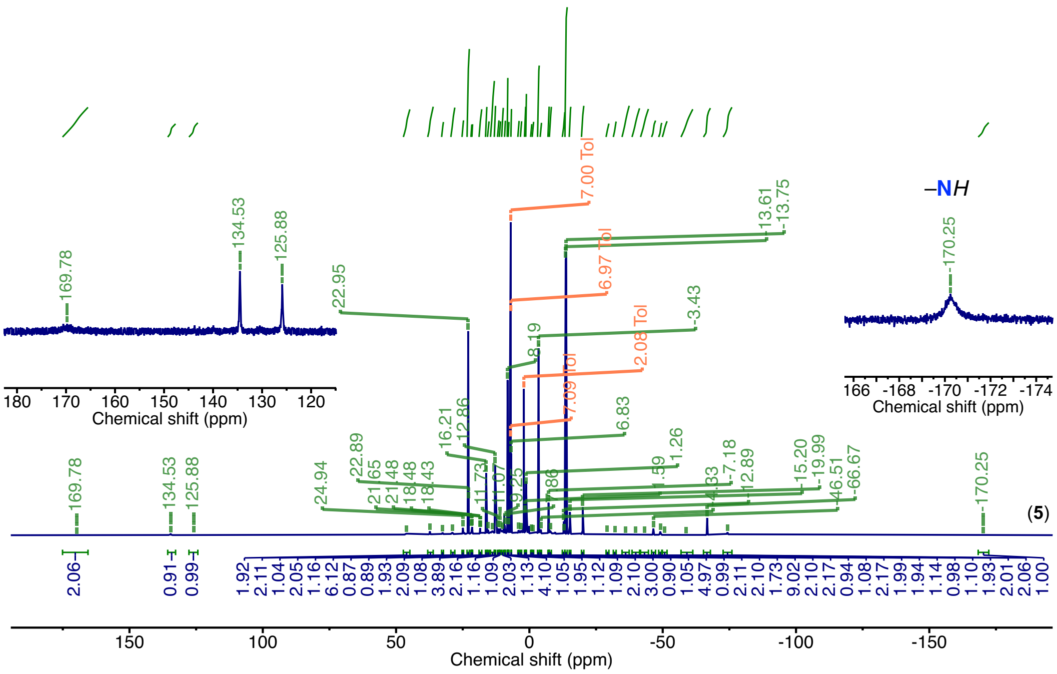

Supplementary Figure 29. ^{1}H NMR (400 MHz, toluene-d$_8$, 298 K) spectrum of **5**. Resonance of the imido-group is specifically labelled for clarity.

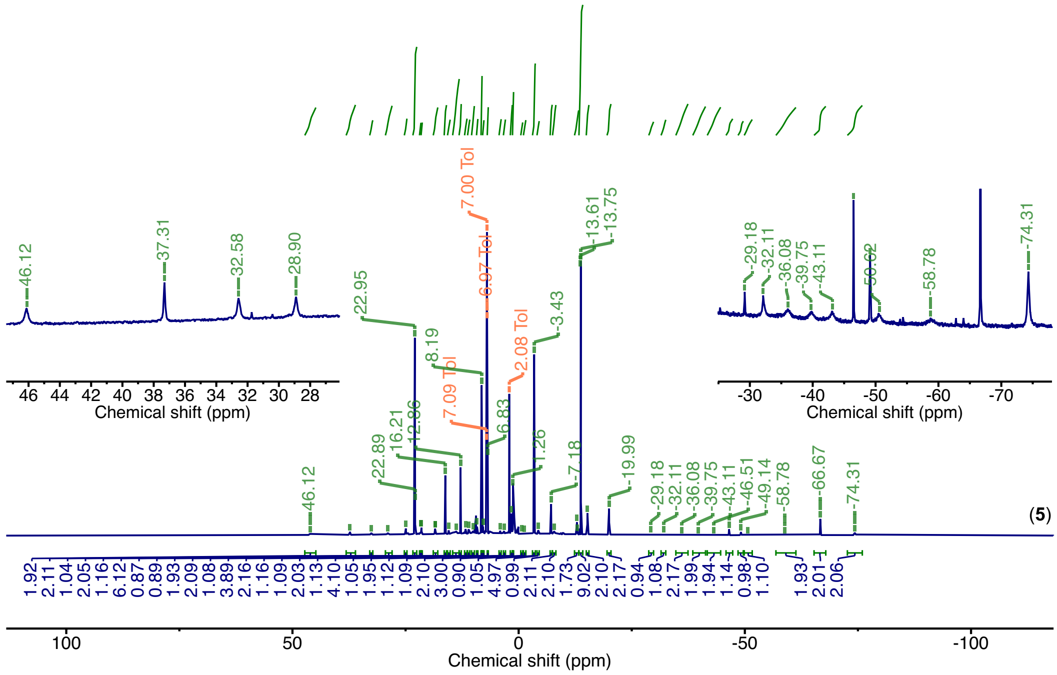

Supplementary Figure 30. Zoom in of ^{1}H NMR (400 MHz, toluene-d$_8$, 298 K) spectrum of **5** between 100 and –100 ppm.

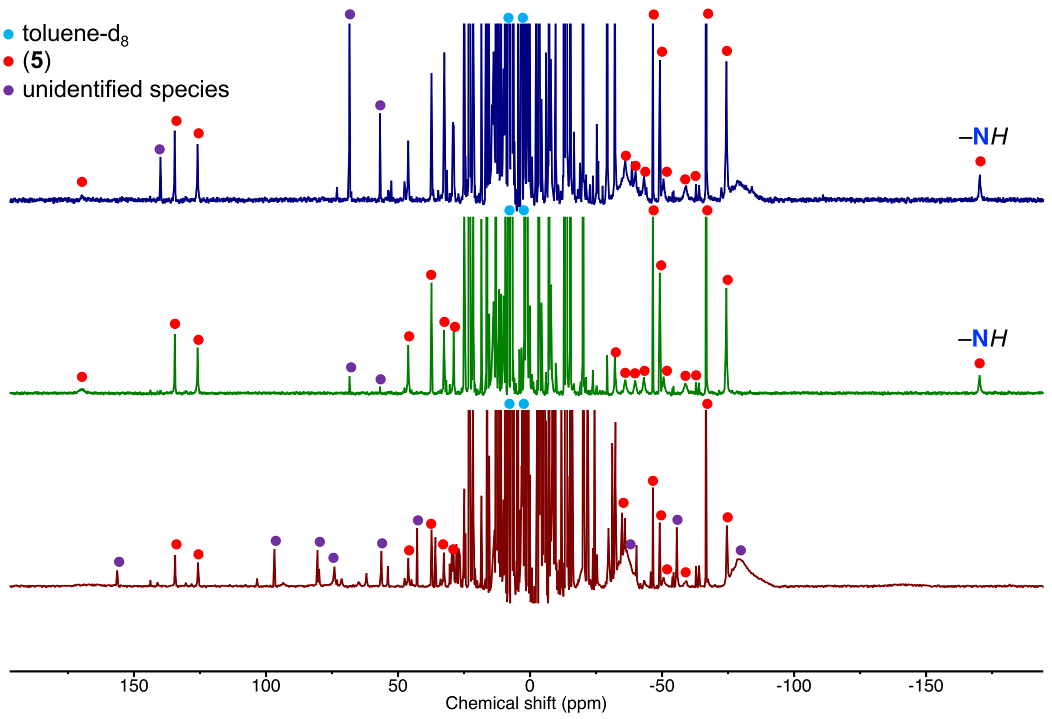

Supplementary Figure 31. Comparison of ^{1}H NMR (400 MHz, toluene-d$_8$, 298 K) spectra of the reaction mixture obtained after exposure of **3** to H_2 gas (**a**), crystals of **5** isolated from the reaction mixture (**b**) and the reaction mixture obtained after exposure of **3** to D_2 gas (**c**). Only the most prominent resonances are labelled for clarity, and the imido proton at –170.25 ppm is explicitly labelled.

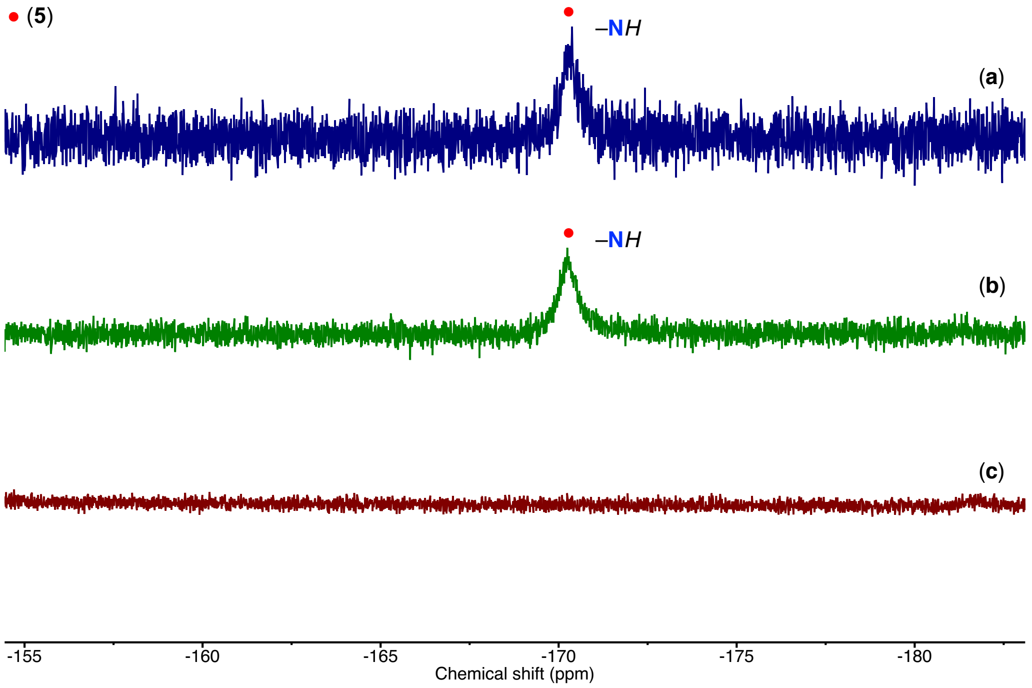

Supplementary Figure 32. Zoomed-in comparison of ^{1}H NMR (400 MHz, toluene-d$_8$, 298 K) spectra of the reaction mixture obtained after exposure of **3** to H_2 gas (**a**), crystals of **5** isolated from the reaction mixture (**b**) and the reaction mixture obtained after exposure of **3** to D_2 gas (**c**). The imido proton at –170.25 ppm is explicitly labelled.

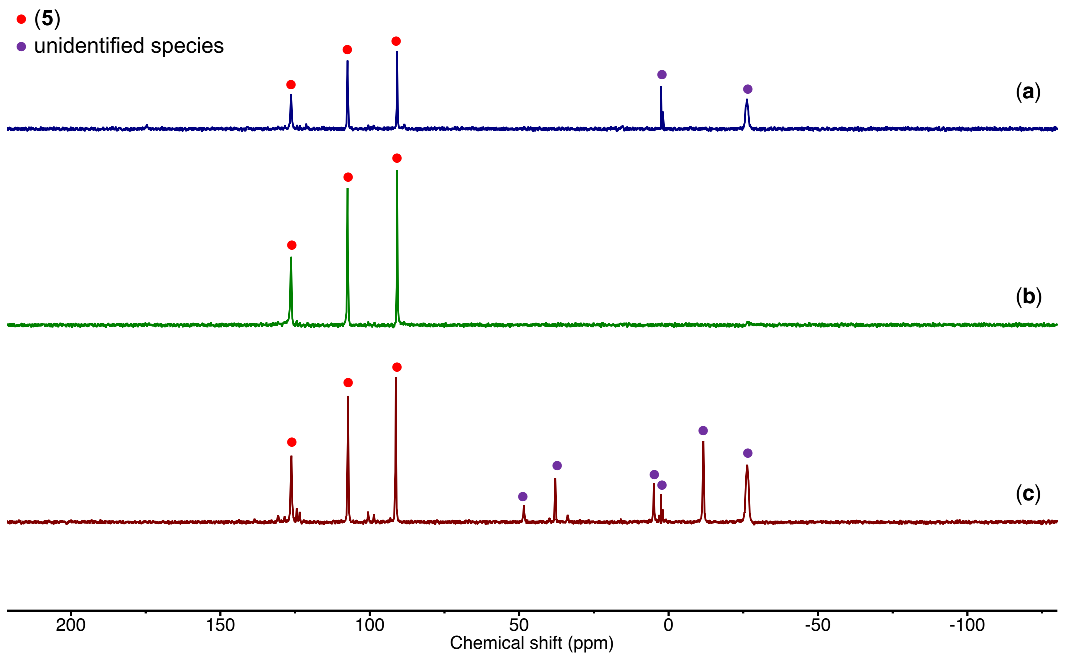

Supplementary Figure 33. Comparison of ^{7}Li NMR (156 MHz, toluene-d_8, 298 K) spectra of the post-crystallisation mother liquor obtained from a reaction of **3** with H_2 (a), isolated crystals of **5** (**b**) and the post-crystallisation mother liquor obtained from a reaction of **3** with D_2 (**c**). For both H_2 and D_2, mother liquor was found to contain unidentified lithium-ligated species.

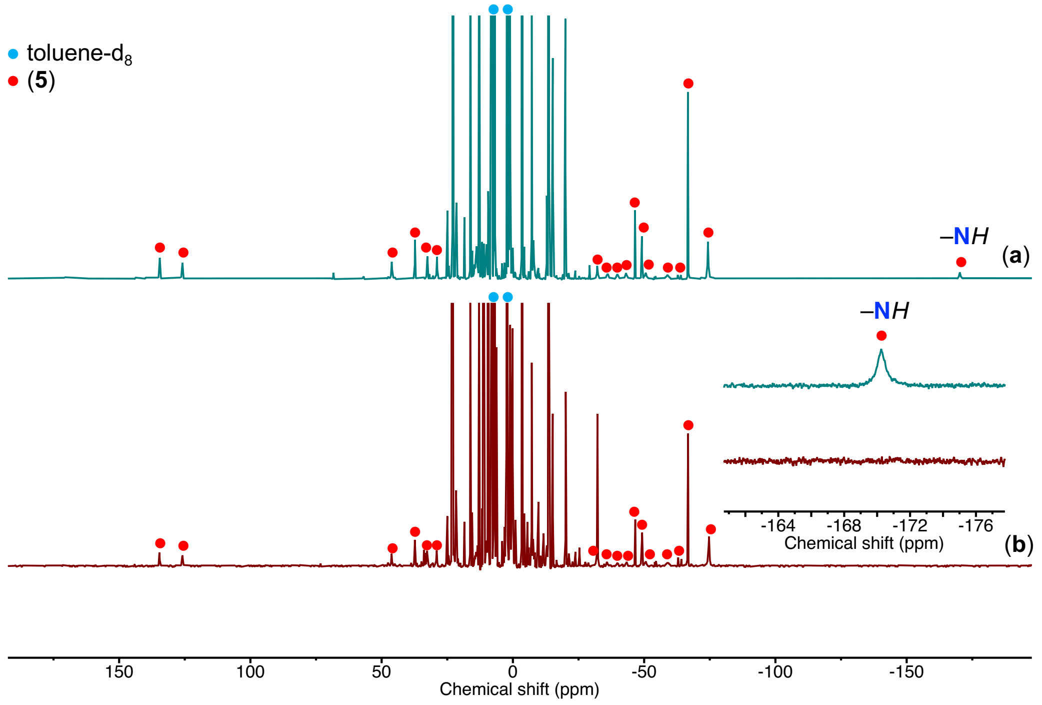

Supplementary Figure 34. Comparison of ^{1}H NMR (400 MHz, toluene-d_8, 298 K) spectra of crystals of **5**, isolated from the reaction mixture obtained after exposure of **3** to H_2 gas (**a**) and the reaction mixture obtained after exposure of **4** to D_2 gas in toluene, giving **5-D₂** (**b**). Only the most prominent resonances are labelled for clarity, and the imido proton at –170.25 ppm is explicitly labelled.

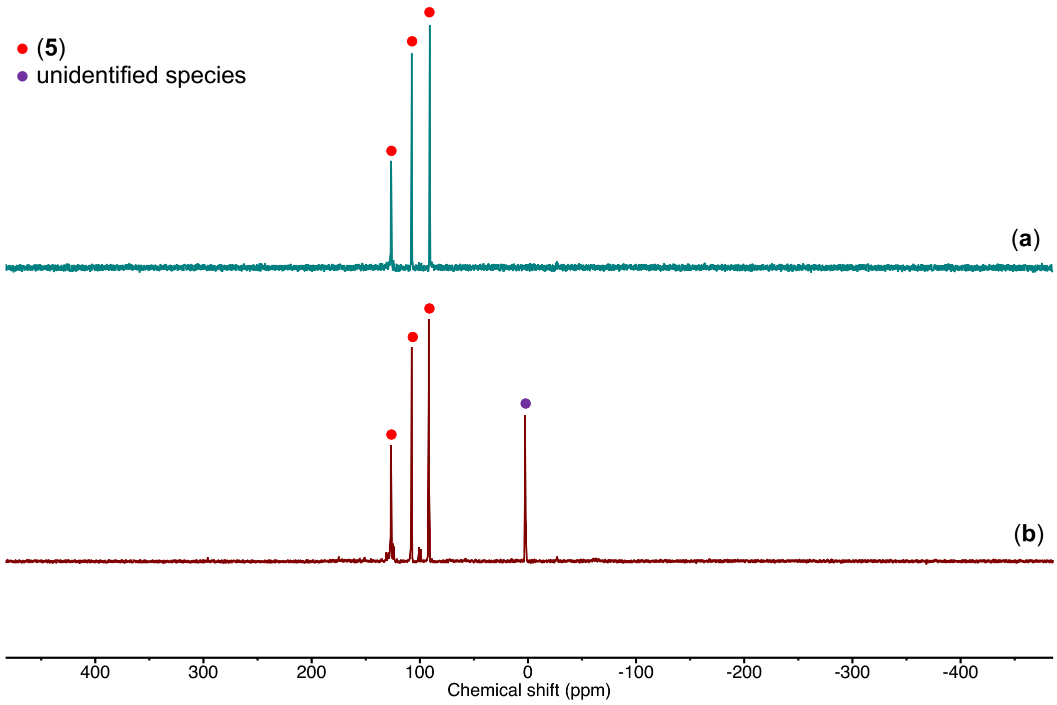

Supplementary Figure 35. Comparison of ^{7}Li NMR (156 MHz, toluene-d$_8$, 298 K) spectra of crystals of **5**, isolated from the reaction mixture obtained after exposure of **3** to H_2 gas (**a**) and the reaction mixture obtained after exposure of **4** to D_2 gas in toluene, giving **5-D$_2$** (**b**).

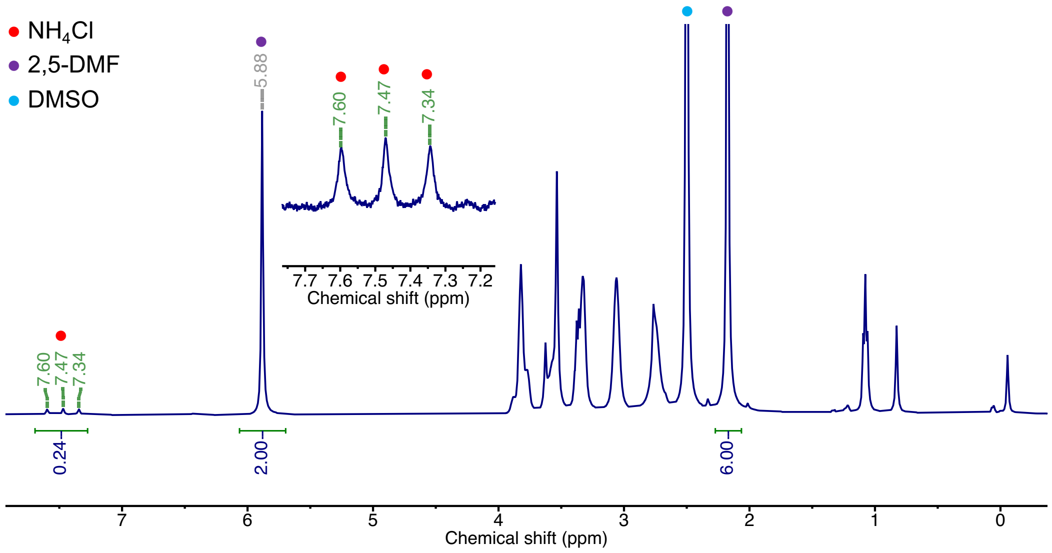

Supplementary Figure 36. Quantitative ^{1}H NMR (400 MHz, DMSO-d$_6$, 298 K) spectrum of a mixture of NH_4Cl and other quenching products formed after the addition of **1** and 2 M HCl in Et_2O (Table 1, Entry 1). 2,5-DMF was added as an internal standard and the spectra was acquired with a 120-s relaxation delay.

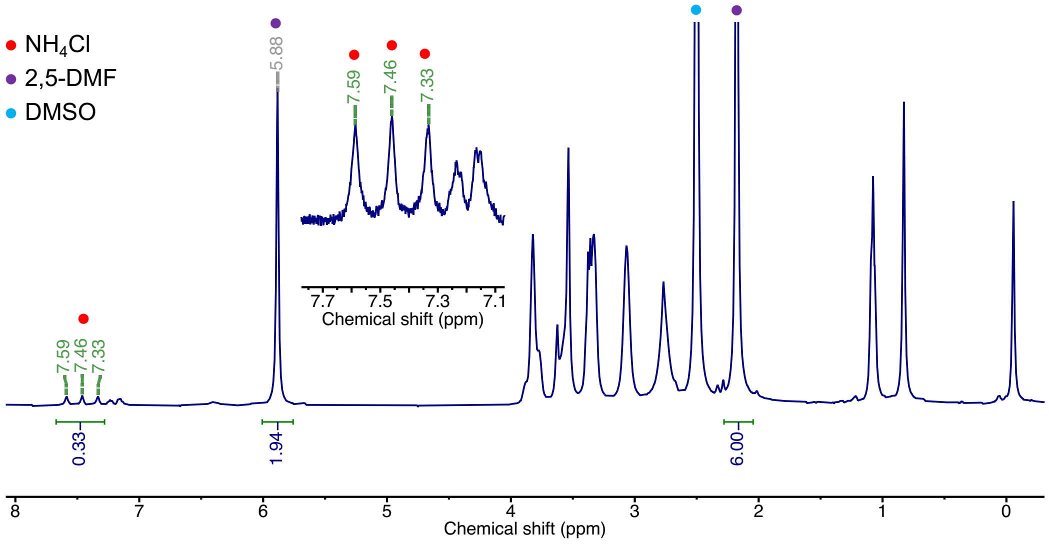

Supplementary Figure 37. Quantitative ^{1}H NMR (400 MHz, DMSO-d$_6$, 298 K) spectrum of a mixture of NH_4Cl and other quenching products formed after the addition of **2-crypt** and 2 M HCl in Et_2O (Table 1, Entry 2). 2,5-DMF was added as an internal standard and the spectra was acquired with a 120-s relaxation delay.

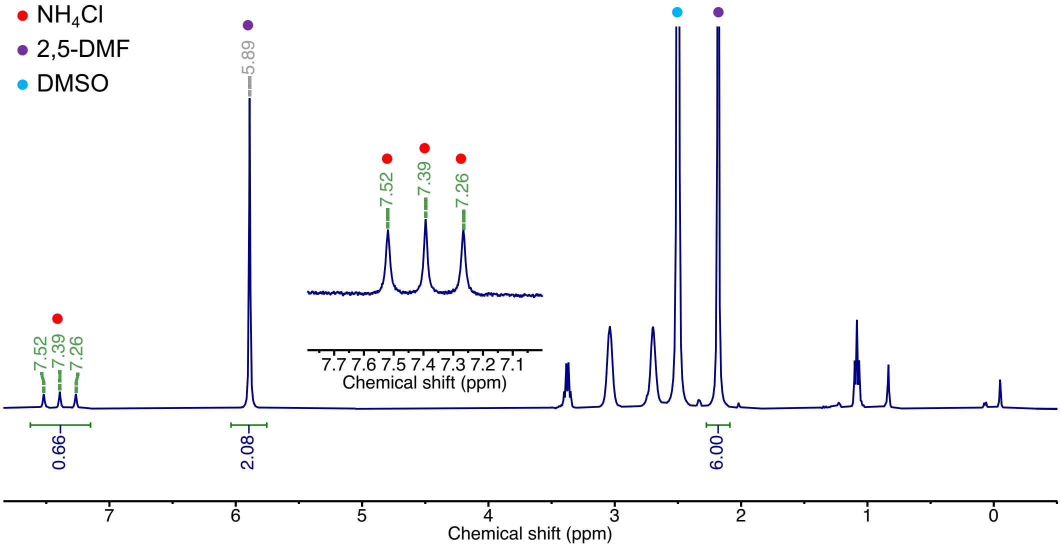

Supplementary Figure 38. Quantitative ^{1}H NMR (400 MHz, DMSO-d6, 298 K) spectrum of a mixture of NH_4Cl and other quenching products formed after the addition of **3** and 2 M HCl in Et_2O (Table 1, Entry 3). 2,5-DMF was added as an internal standard and the spectra was acquired with a 120-s relaxation delay.

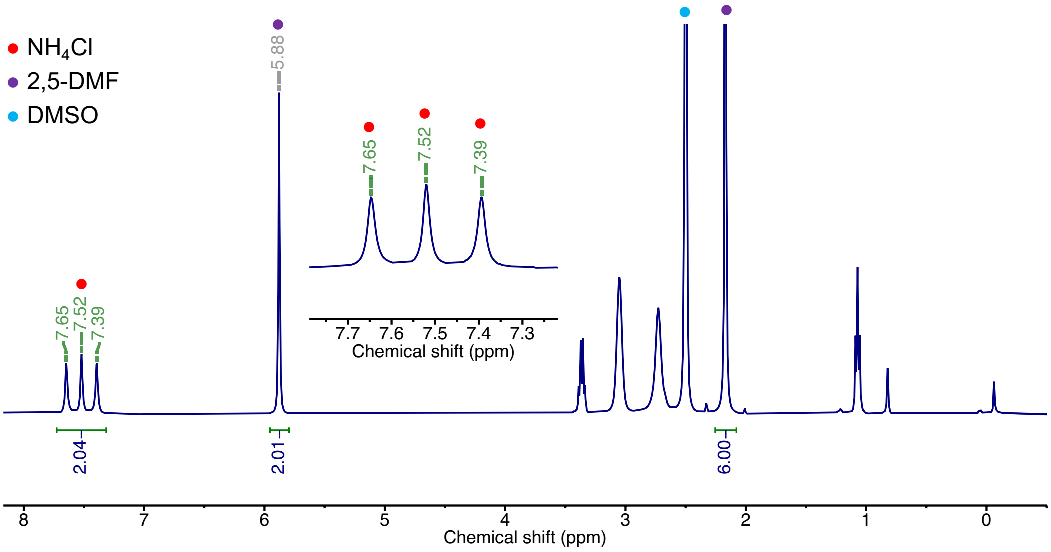

Supplementary Figure 39. Quantitative ^{1}H NMR (400 MHz, DMSO-d6, 298 K) spectrum of a mixture of NH_4Cl and other quenching products formed after the addition of **4** and 2 M HCl in Et_2O (Table 1, Entry 4). 2,5-DMF was added as an internal standard and the spectra was acquired with a 120-s relaxation delay.

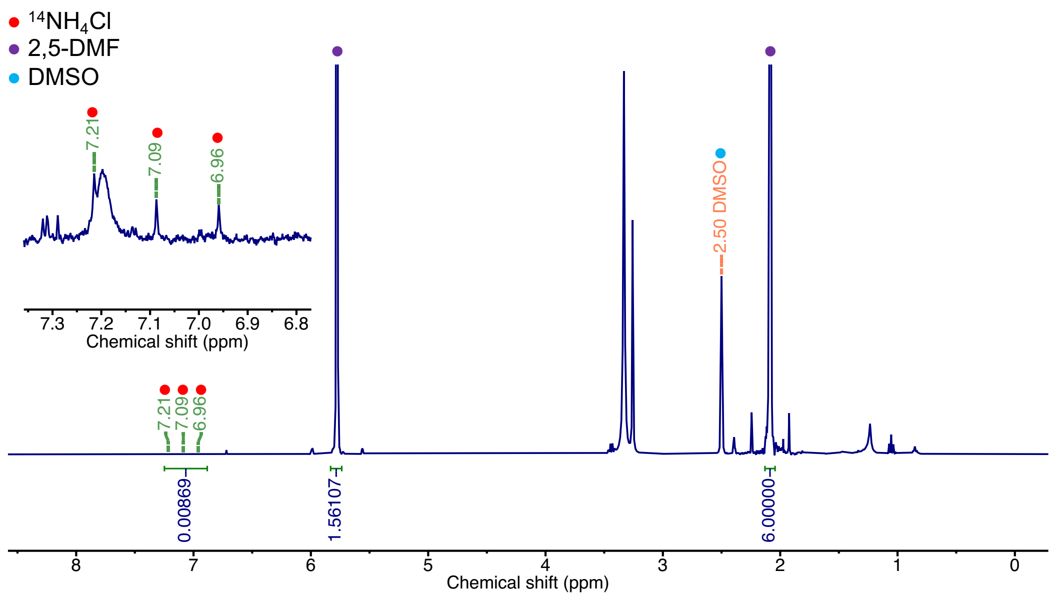

Supplementary Figure 40. Quantitative ^{1}H NMR (400 MHz, DMSO-d_6, 298 K) spectrum of an aliquot taken from the HCl-trapped volatile fraction in a catalyst-free acidification reaction using 600 equivalents of KC_8/[Cy_3PH][I] in diethyl ether (Table 1, Entry 5). <0.01 eq. of $^{14}NH_4^+$ was detected. Spectrum was acquired with a 25-s relaxation delay.

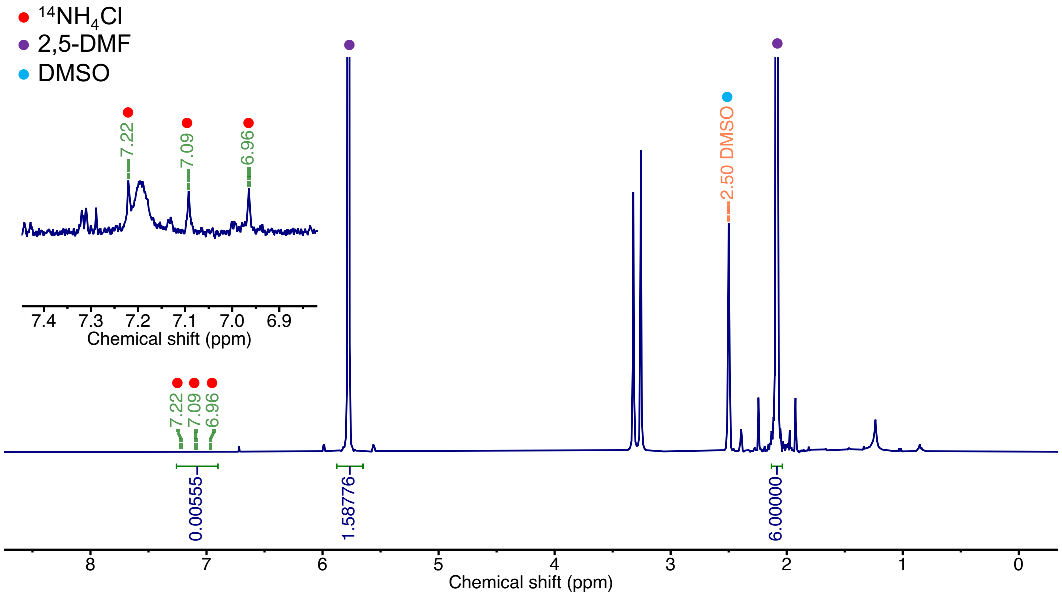

Supplementary Figure 41. Quantitative ^{1}H NMR (400 MHz, DMSO-d_6, 298 K) spectrum of an aliquot taken from the HCl-trapped volatile fraction in a catalyst-free acidification reaction using 600 equivalents of KC_8/[Cy_3PH][I] in diethyl ether (Table 1, Entry 6). No $^{15}NH_4^+$ and <0.01 eq. of $^{14}NH_4^+$ was detected. Spectrum was acquired with a 25-s relaxation delay.

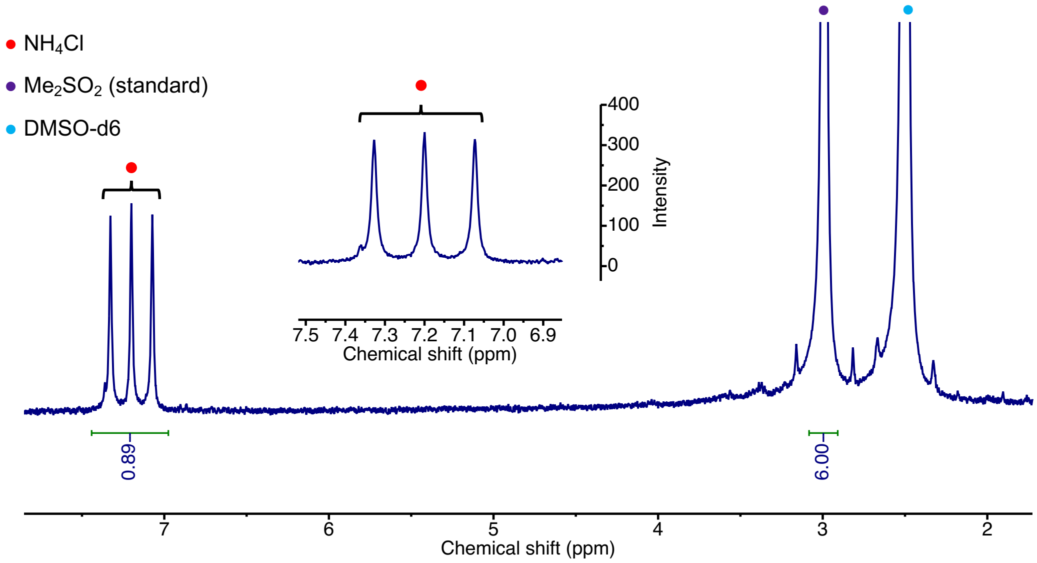

Supplementary Figure 42. Quantitative ^{1}H NMR (400 MHz, DMSO-d_6, 298 K) spectrum of a mixture of NH_4Cl and other quenching products formed after trapping the volatiles from the addition of **1** and 4 eq./10 eq. of KC_8/[CyPH][I] in *n*-hexane (Table 1, Entry 7). Me_2SO_2 was added as an internal standard and the spectra was acquired with a 120-s relaxation delay.

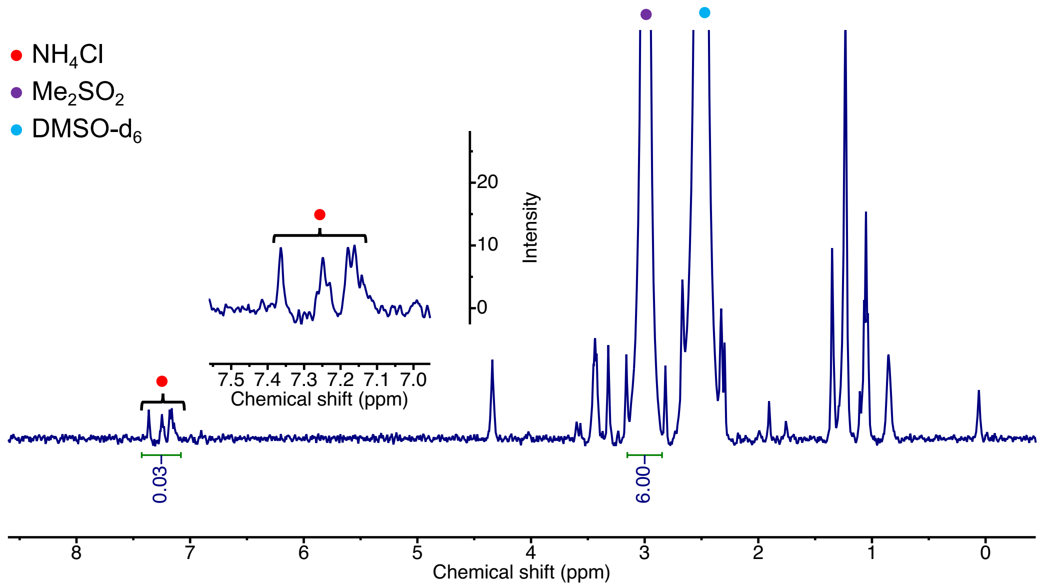

Supplementary Figure 43. Quantitative ^{1}H NMR (400 MHz, DMSO-d_6, 298 K) spectrum of a mixture of NH_4Cl and other quenching products formed after trapping the volatiles from the addition of **1** and 4 eq./10 eq. of KC_8/[Cy_3PH][I] in toluene (Table 1, Entry 8). Me_2SO_2 was added as an internal standard and the spectra was acquired with a 120-s relaxation delay.

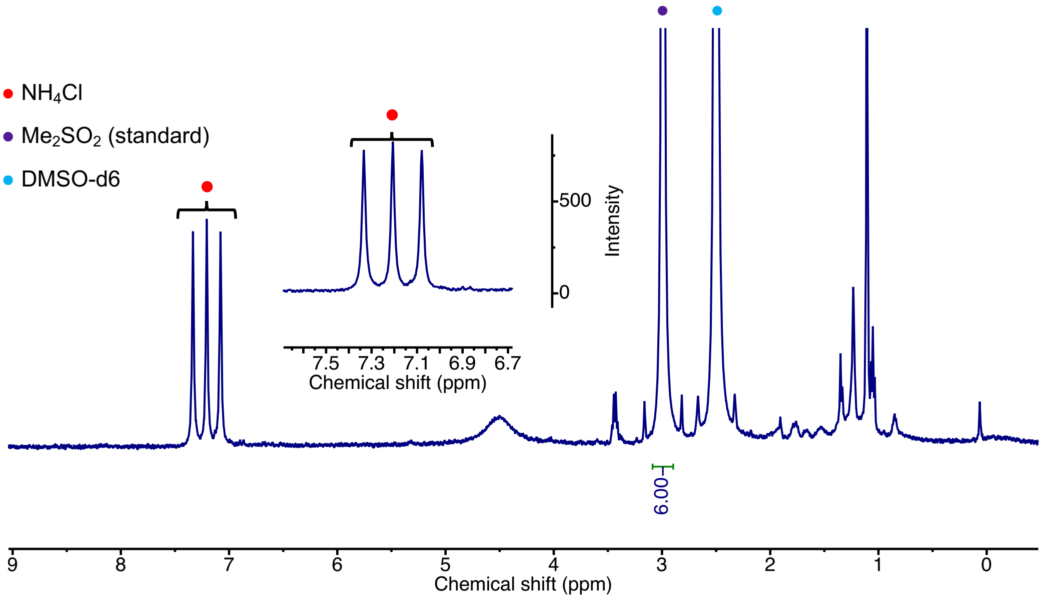

Supplementary Figure 44. Quantitative ^{1}H NMR (400 MHz, DMSO-d$_6$, 298 K) spectrum of a mixture of NH_4Cl and other quenching products formed after trapping the volatiles from the addition of **1** and 4 eq./10 eq. of KC_8/[Cy_3PH][I] in diethyl ether (Table 1, Entry 9). Me_2SO_2 was added as an internal standard and the spectra was acquired with a 120-s relaxation delay.

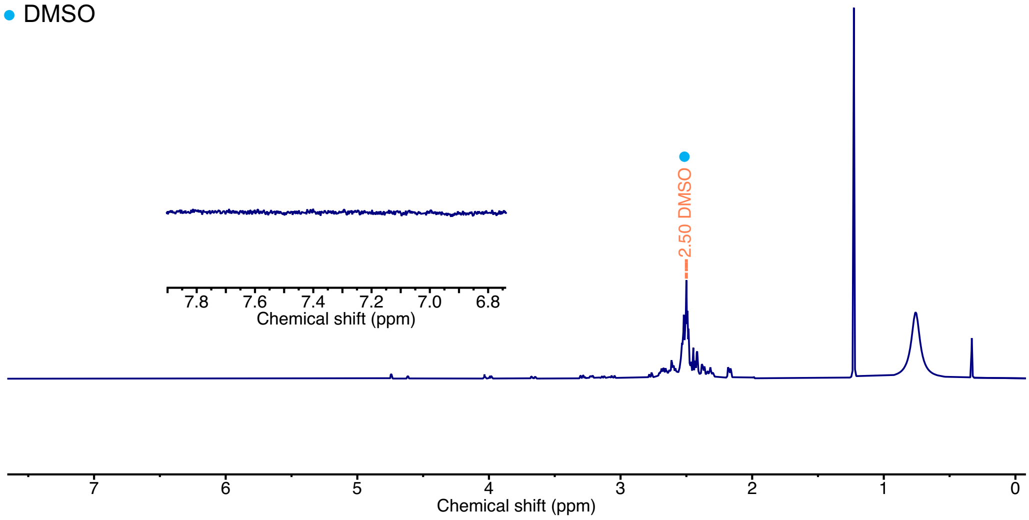

Supplementary Figure 45. Quantitative ^{1}H NMR (400 MHz, DMSO-d$_6$, 298 K) spectrum of an aliquot taken from the HCl-trapped volatile fraction in a catalytic acidification reaction using 600 equivalents of KC_8/[Et_3NH][BPh_4] in diethyl ether (Table 1, Entry 10). No $^{14}NH_4^+$ peak was detected. Spectrum was acquired with a 25-s relaxation delay.

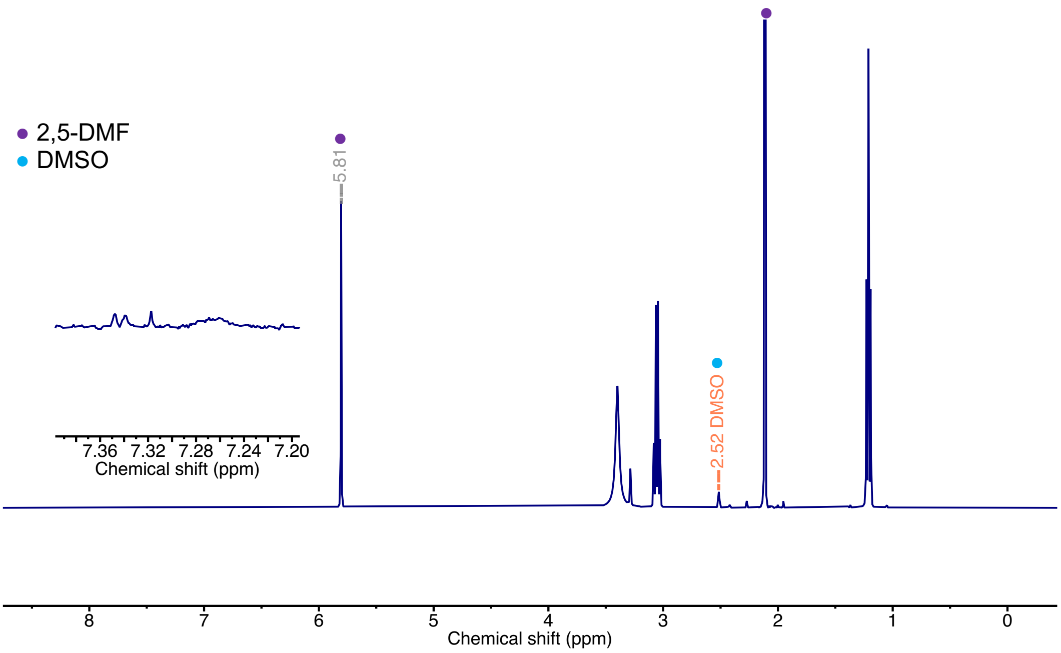

Supplementary Figure 46. Quantitative ^{1}H NMR (400 MHz, DMSO-d_6, 298 K) spectrum of an aliquot taken from the HCl-trapped volatile fraction in a catalytic acidification reaction using 600 equivalents of KC_8/[Et_3NH][Cl] in diethyl ether (Table 1, Entry 11). No $^{14}NH_4^+$ peak was detected. Spectrum was acquired with a 25-s relaxation delay.

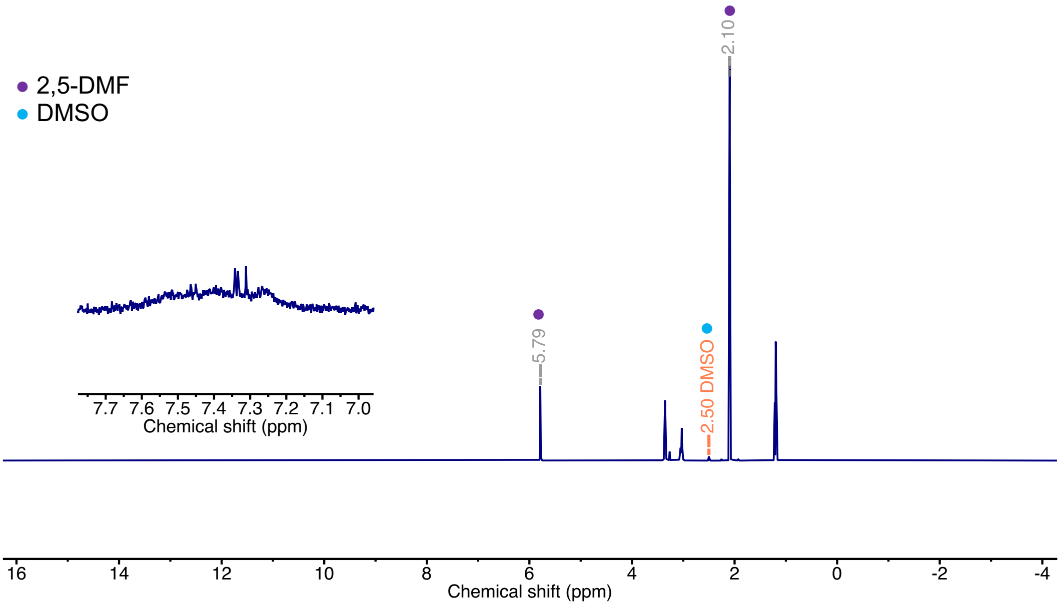

Supplementary Figure 47. Quantitative ^{1}H NMR (400 MHz, DMSO-d_6, 298 K) spectrum of an aliquot taken from the HCl-trapped volatile fraction in a catalytic acidification reaction using 600 equivalents of KC_8/[Et_3NH][I] in diethyl ether (Table 1, Entry 12). No $^{14}NH_4^+$ peak was detected. Spectrum was acquired with a 25-s relaxation delay.

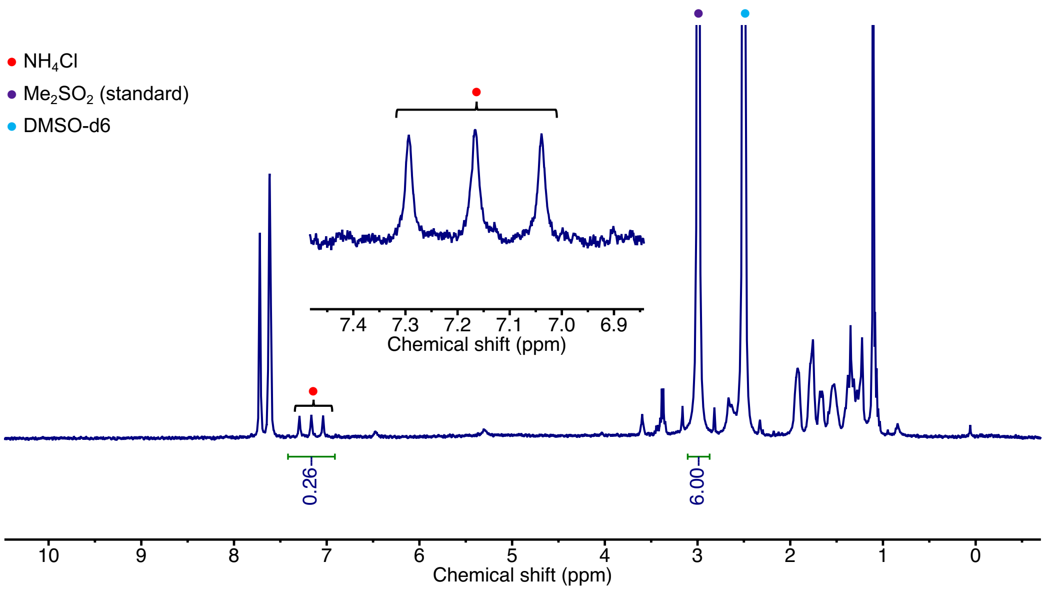

Supplementary Figure 48. Quantitative ^{1}H NMR (400 MHz, DMSO-d$_6$, 298 K) spectrum of a mixture of NH_4Cl and other quenching products formed after trapping the volatiles from the addition of **1** and 129 eq./129 eq. of KC_8/[Cy_3PH][BAr^{F20}] in Et_2O (Table 1, Entry 13). Me_2SO_2 was added as an internal standard and the spectra was acquired with a 120-s relaxation delay.

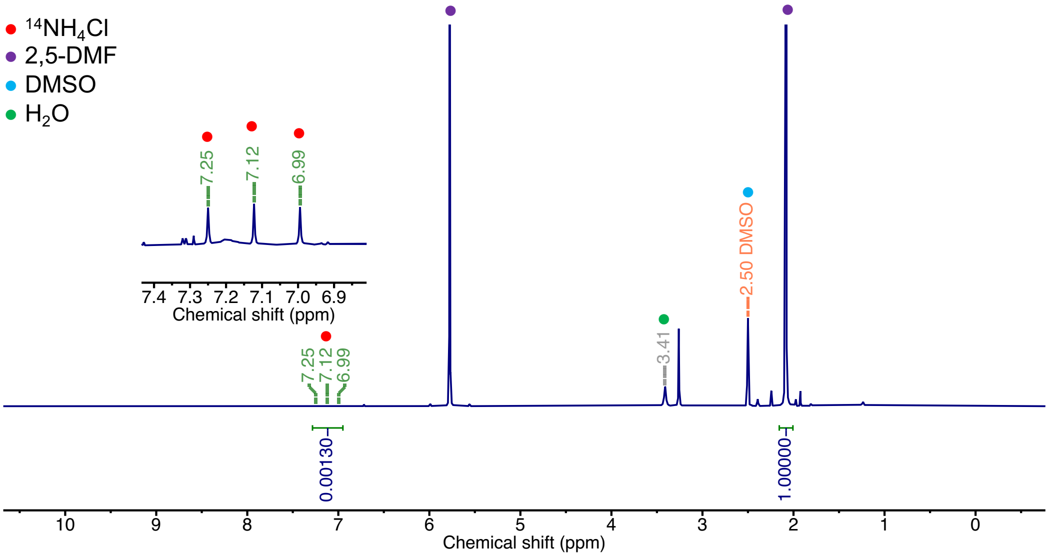

Supplementary Figure 49. Quantitative ^{1}H NMR (400 MHz, DMSO-d$_6$, 298 K) spectrum of an aliquot taken from the HCl-trapped volatile fraction in a catalytic acidification reaction using 600 equivalents of Na/[Cy_3PH][I] in diethyl ether (Table 1, Entry 14). The $^{14}NH_4^{+}$ 1:1:1 triplet was integrated relative to the methyl protons of 2.5-dimethylfuran (2.15 ppm, s). Spectrum was acquired with a 25-s relaxation delay.

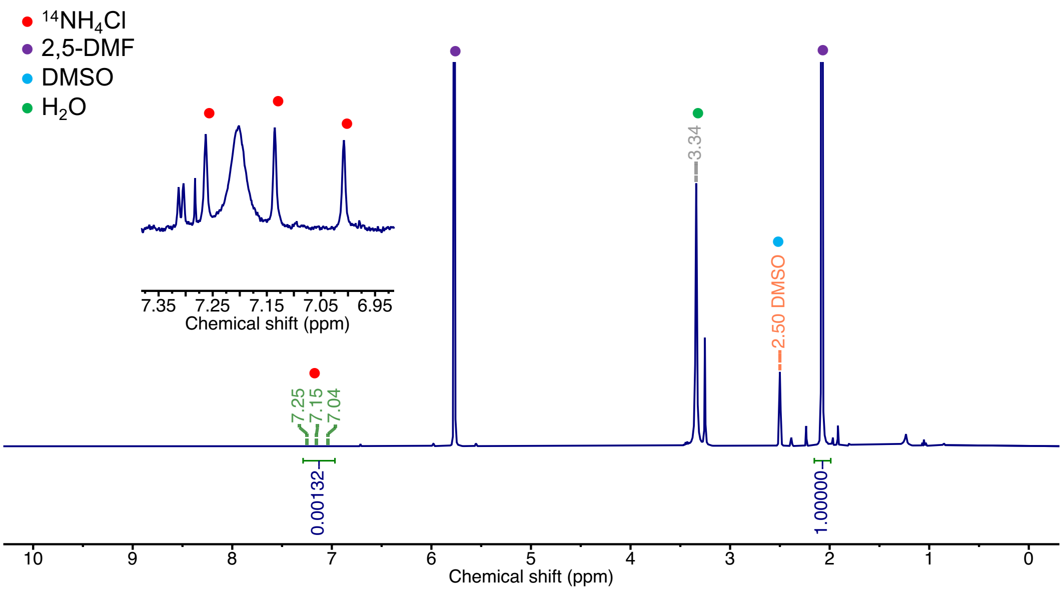

Supplementary Figure 50. Quantitative ^{1}H NMR (400 MHz, DMSO-d_6, 298 K) spectrum of an aliquot taken from the HCl-trapped volatile fraction in a catalytic acidification reaction using 600 equivalents of K/[Cy_3PH][I] in diethyl ether (Table 1, Entry 15). The $^{14}NH_4^+$ 1:1:1 triplet was integrated relative to the methyl protons of 2.5-dimethylfuran (2.15 ppm, s). Spectrum was acquired with a 25-s relaxation delay.

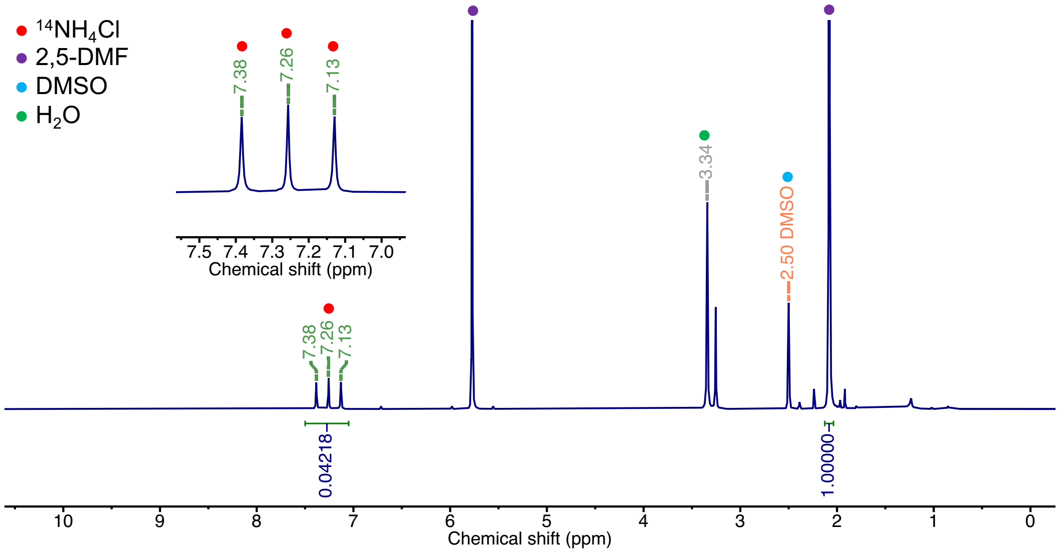

Supplementary Figure 51. Quantitative ^{1}H NMR (400 MHz, DMSO-d_6, 298 K) spectrum of an aliquot taken from the HCl-trapped volatile fraction in a catalytic acidification reaction using 173 equivalents of KC_8/[Cy_3PH][I] in diethyl ether (Table 1, Entry 16). The $^{14}NH_4^+$ 1:1:1 triplet was integrated relative to the methyl protons of 2.5-dimethylfuran (2.15 ppm, s). Spectrum was acquired with a 25-s relaxation delay.

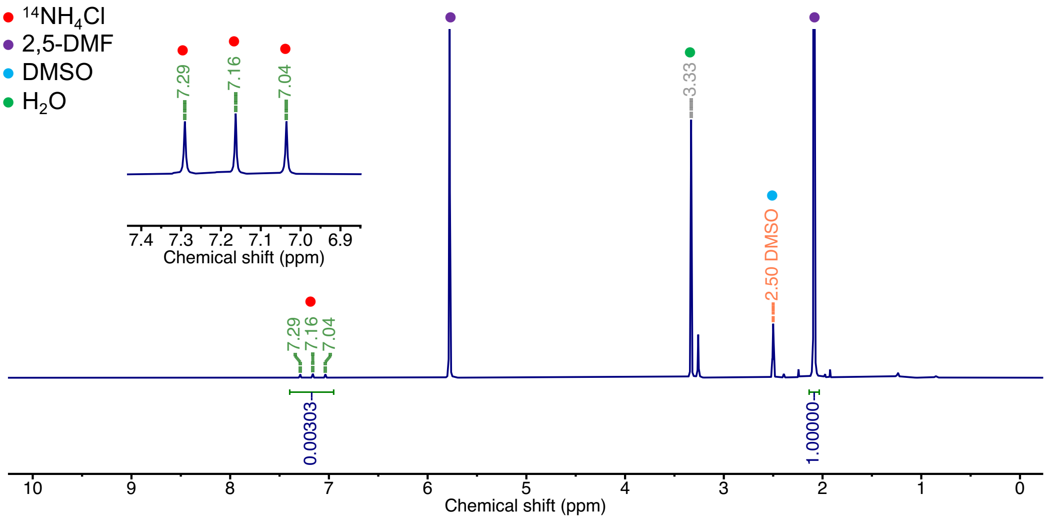

Supplementary Figure 52. Quantitative ^1H NMR (400 MHz, DMSO-d$_6$, 298 K) spectrum of an aliquot taken from the HCl-trapped volatile fraction in a catalytic acidification reaction using 173 equivalents of KC_8/[Cy_3PH][I] in diethyl ether (Table 1, Entry 17) at an increased absolute pressure of 2.21 bar. The $^{14}NH_4^+$ 1:1:1 triplet was integrated relative to the methyl protons of 2.5-dimethylfuran (2.15 ppm, s). Spectrum was acquired with a 25-s relaxation delay.

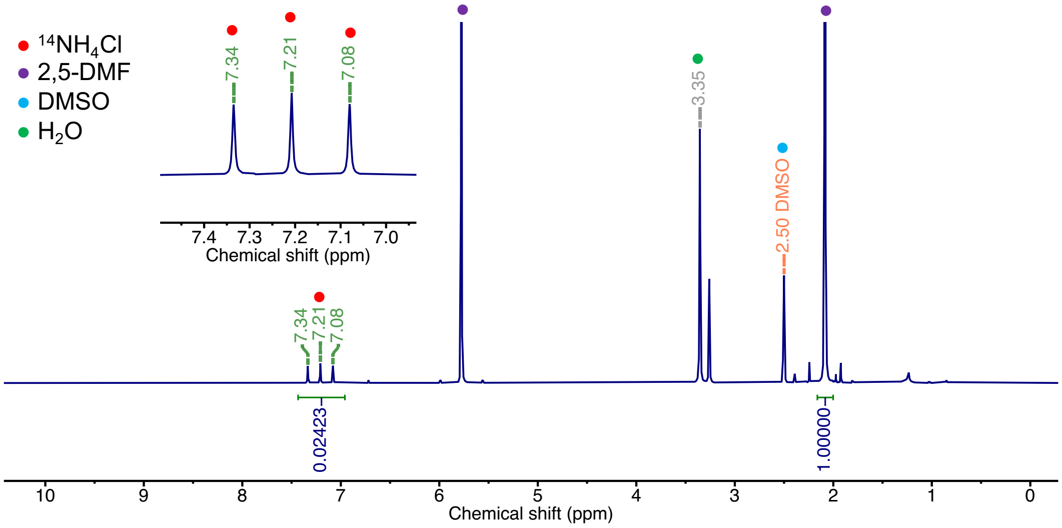

Supplementary Figure 53. Quantitative ^1H NMR (400 MHz, DMSO-d$_6$, 298 K) spectrum of an aliquot taken from the HCl-trapped volatile fraction in a catalytic acidification reaction using 300 equivalents of KC_8/[Cy_3PH][I] in diethyl ether (Table 1, Entry 18). The $^{14}NH_4^+$ 1:1:1 triplet was integrated relative to the methyl protons of 2.5-dimethylfuran (2.15 ppm, s). Spectrum was acquired with a 25-s relaxation delay.

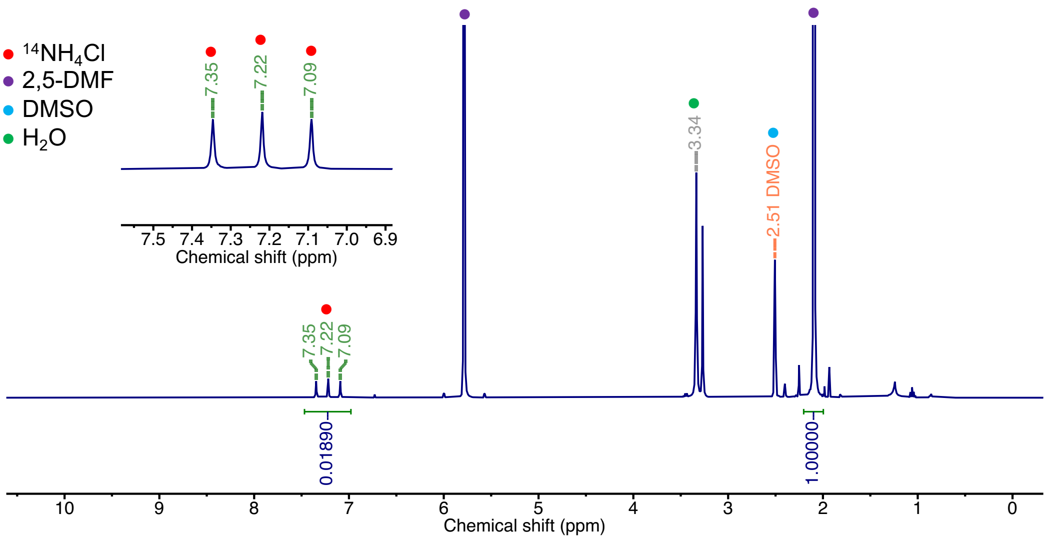

Supplementary Figure 54. Quantitative ^{1}H NMR (400 MHz, DMSO-d$_6$, 298 K) spectrum of an aliquot taken from the HCl-trapped volatile fraction in a catalytic acidification reaction using 600 equivalents of KC_8/[Cy_3PH][I] in diethyl ether (Table 1, Entry 19). The $^{14}NH_4^+$ 1:1:1 triplet was integrated relative to the methyl protons of 2.5-dimethylfuran (2.15 ppm, s). Spectrum was acquired with a 25-s relaxation delay.

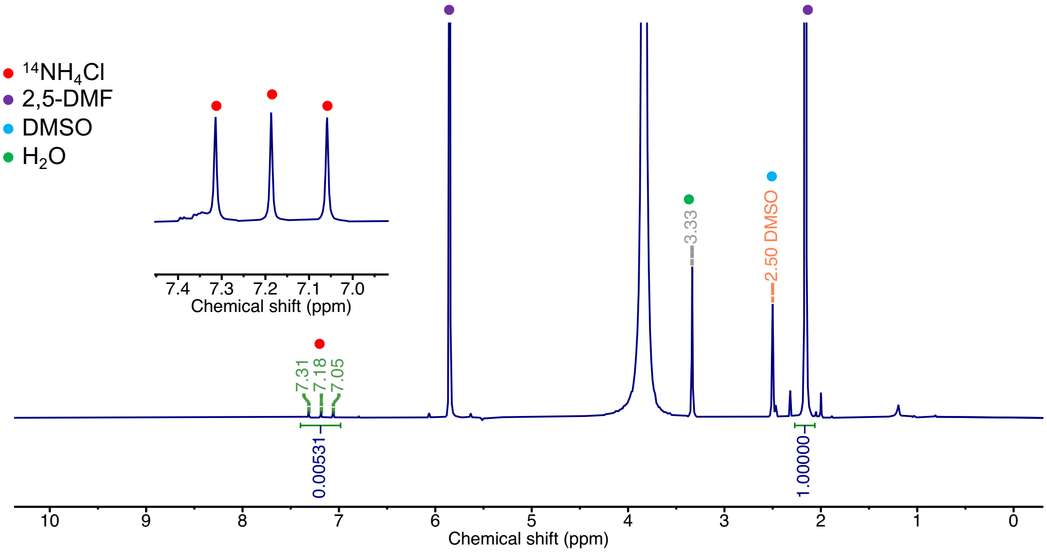

Supplementary Figure 55. Quantitative ^{1}H NMR (400 MHz, DMSO-d$_6$, 298 K) spectrum of an aliquot taken from the HCl-trapped volatile fraction in a catalytic acidification reaction using 600 equivalents of Rb/[Cy_3PH][I] in diethyl ether (Table 1, Entry 20). The $^{14}NH_4^+$ 1:1:1 triplet was integrated relative to the methyl protons of 2.5-dimethylfuran (2.15 ppm, s). Spectrum was acquired with a 25-s relaxation delay.

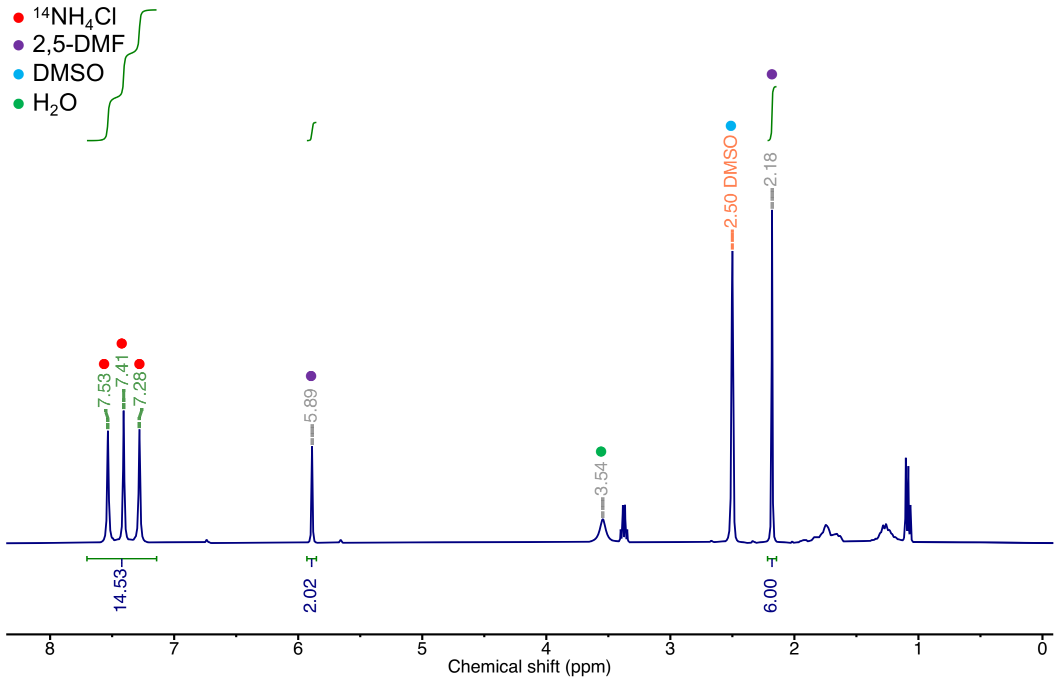

Supplementary Figure 56. Quantitative ^{1}H NMR (400 MHz, DMSO-d6, 298 K) spectrum of an aliquot taken from the HCl-trapped volatile fraction in a catalytic acidification reaction using 600 equivalents of RbC_8/[Cy_3PH][I] in diethyl ether (Table 1, Entry 21, $^{14}N_2$). The $^{14}NH_4^+$ 1:1:1 triplet was integrated relative to the methyl protons of 2.5-dimethylfuran (2.15 ppm, s). Spectrum was acquired with a 25-s relaxation delay.

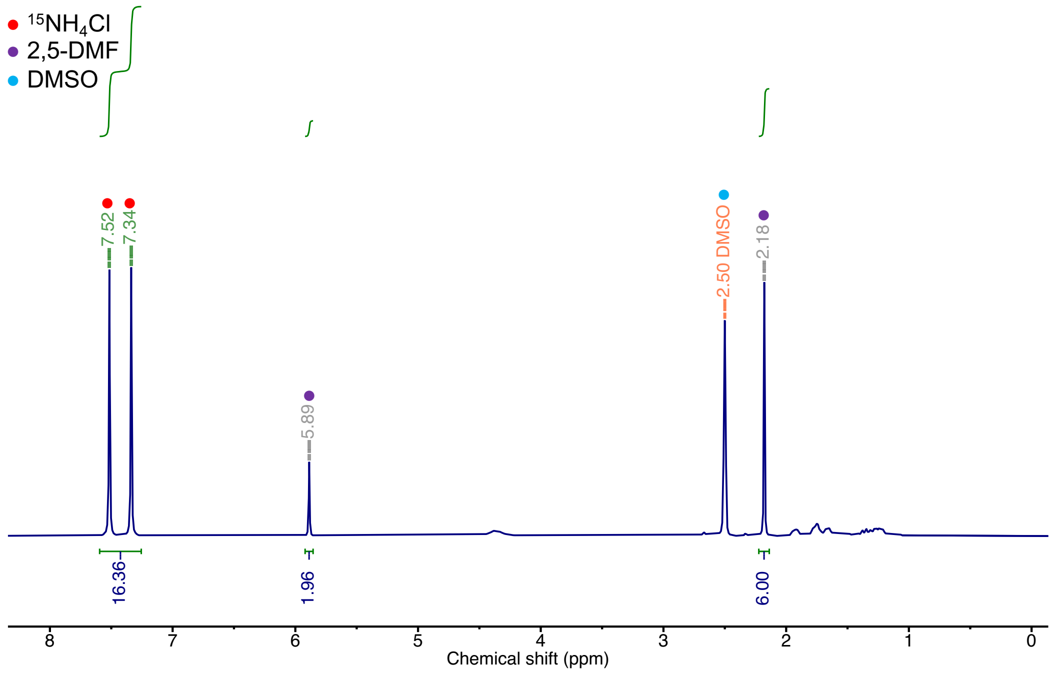

Supplementary Figure 57. Quantitative ^{1}H NMR (400 MHz, DMSO-d6, 298 K) spectrum of an aliquot taken from the HCl-trapped volatile fraction in a catalytic acidification reaction using 600 equivalents of RbC_8/[Cy_3PH][I] in diethyl ether (Table 1, Entry 21, $^{15}N_2$). The $^{15}NH_4^+$ 1:1 doublet was integrated relative to the methyl protons of 2.5-dimethylfuran (2.15 ppm, s). Spectrum was acquired with a 25-s relaxation delay.

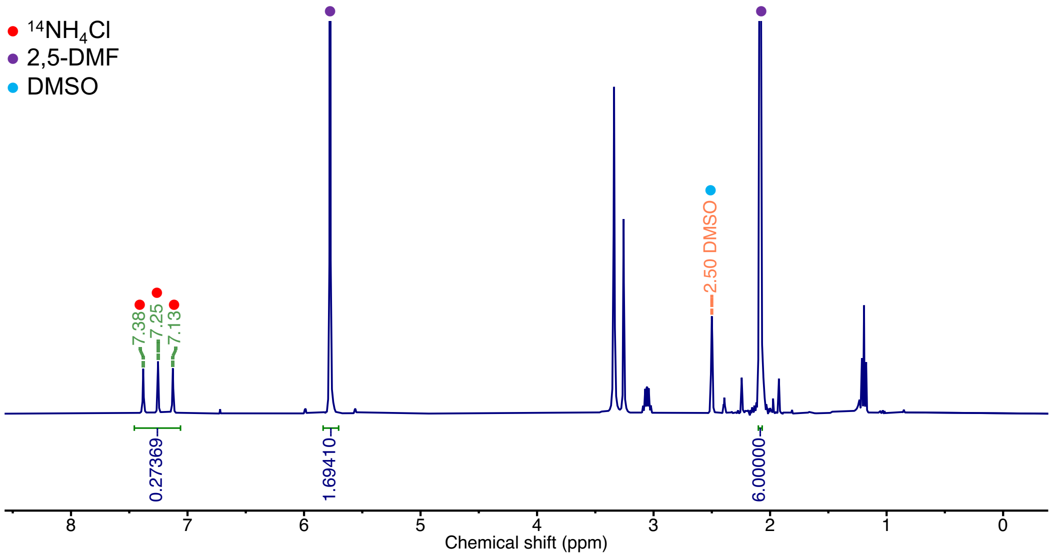

Supplementary Figure 58. Quantitative ^1H NMR (400 MHz, DMSO-d_6, 298 K) spectrum of an aliquot taken from the HCl-trapped volatile fraction in a catalytic acidification reaction using 600 equivalents of RbC_8/[Cy_3PH][I] in diethyl ether (Table 1, Entry 22, $^{14}N_2$). The $^{14}NH_4^+$ 1:1:1 triplet was integrated relative to the methyl protons of 2.5-dimethylfuran (2.15 ppm, s). Spectrum was acquired with a 25-s relaxation delay.

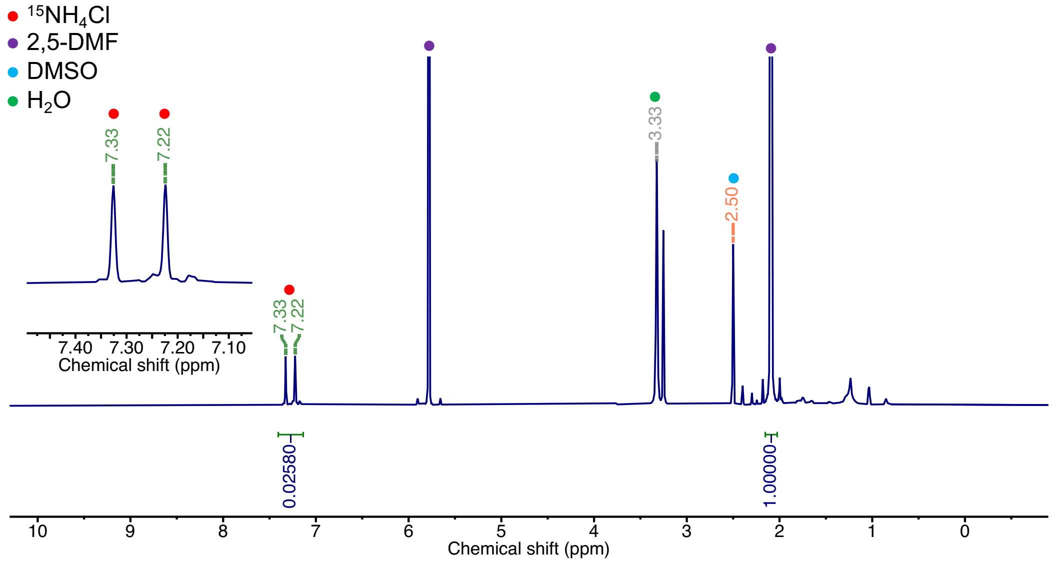

Supplementary Figure 59. Quantitative ^1H NMR (400 MHz, DMSO-d_6, 298 K) spectrum of an aliquot taken from the HCl-trapped volatile fraction in a catalytic acidification reaction using 600 equivalents of RbC_8/[Cy_3PH][I] in diethyl ether (Table 1, Entry 22, $^{15}N_2$). The $^{15}NH_4^+$ 1:1 doublet was integrated relative to the methyl protons of 2.5-dimethylfuran (2.15 ppm, s). Spectrum was acquired with a 25-s relaxation delay.

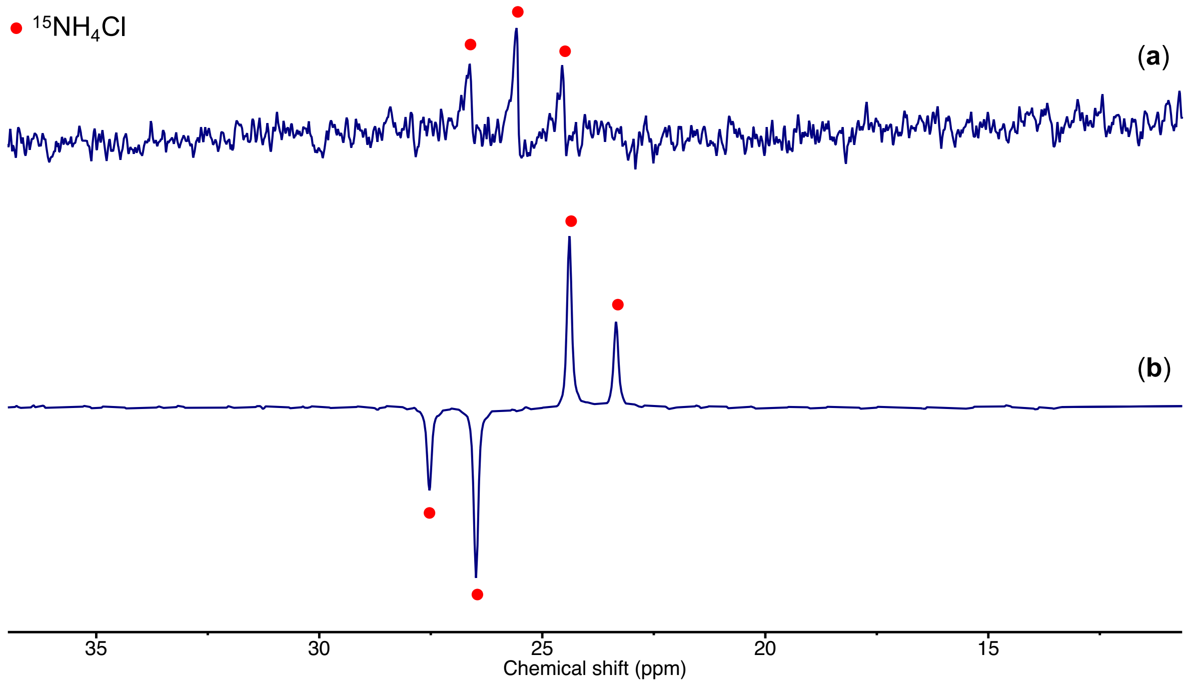

Supplementary Figure 60. ^{15}N NMR (70.97 MHz, benzene-d$_6$, 298 K) (a) conventional and (b) INEPT spectra of an aliquot taken from the HCl-trapped volatile fraction in a catalytic acidification reaction using 600 equivalents of RbC_8/[Cy_3PH][I] in diethyl ether (Table 1, Entry 22, $^{15}N_2$) showing the presence of $^{15}NH_4^+$ at 24.8 ppm ($^1J_{15N-H}$ = 70.4 Hz) referenced to NH_3 at 0 ppm.

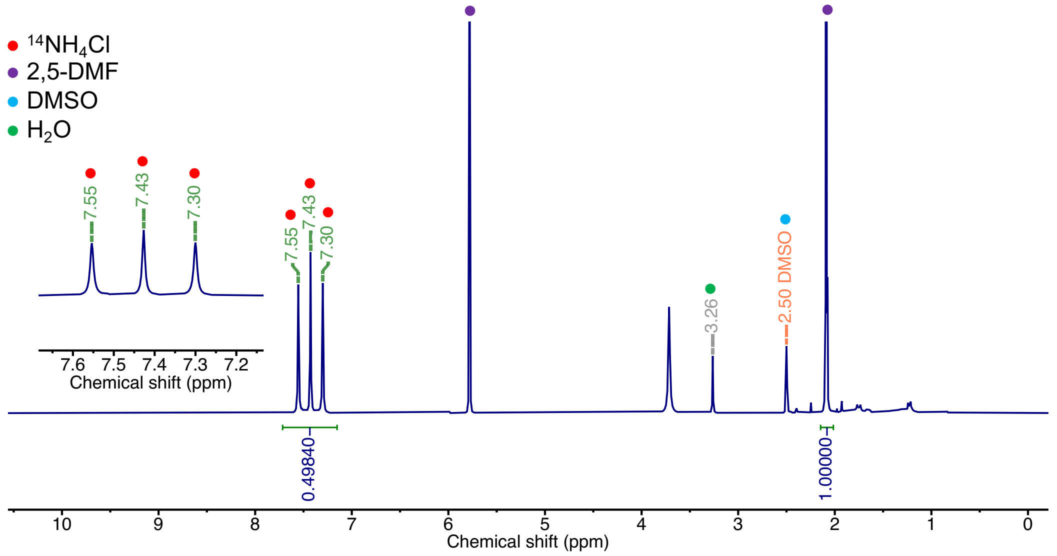

Supplementary Figure 61. Quantitative ^1H NMR (400 MHz, DMSO-d$_6$, 298 K) spectrum of an aliquot taken from the HCl-trapped volatile fraction in a catalytic acidification reaction using 600 equivalents of Cs/[Cy_3PH][I] in diethyl ether (Table 1, Entry 23). The $^{14}NH_4^+$ 1:1:1 triplet was integrated relative to the methyl protons of 2.5-dimethylfuran (2.15 ppm, s). Spectrum was acquired with a 25-s relaxation delay.

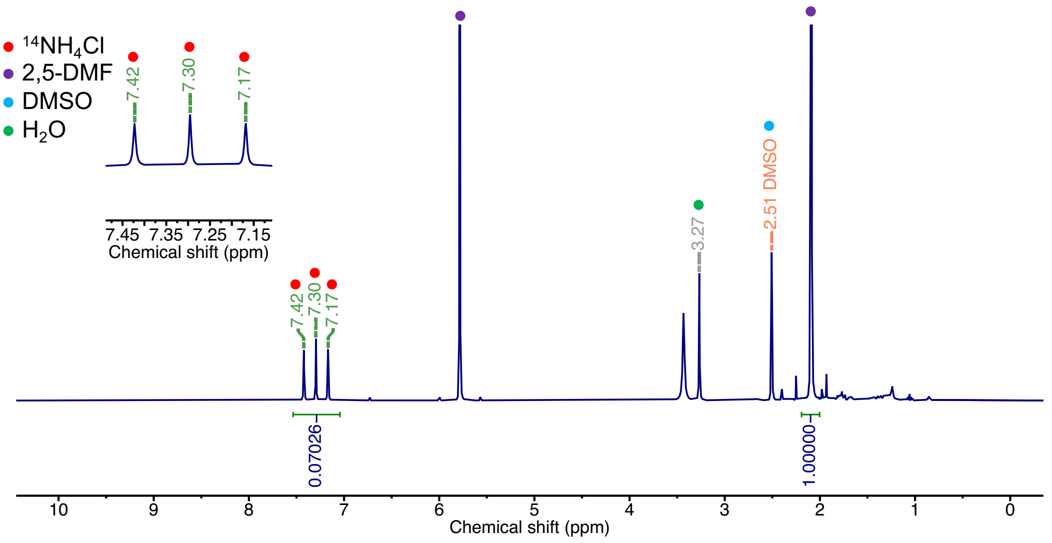

Supplementary Figure 62. Quantitative ^{1}H NMR (400 MHz, DMSO-d$_6$, 298 K) spectrum of an aliquot taken from the HCl-trapped volatile fraction in a catalytic acidification reaction using 600 equivalents of CsC_8/[Cy_3PH][I] in diethyl ether (Table 1, Entry 24). The $^{14}NH_4^+$ 1:1:1 triplet was integrated relative to the methyl protons of 2.5-dimethylfuran (2.15 ppm, s). Spectrum was acquired with a 25-s relaxation delay.

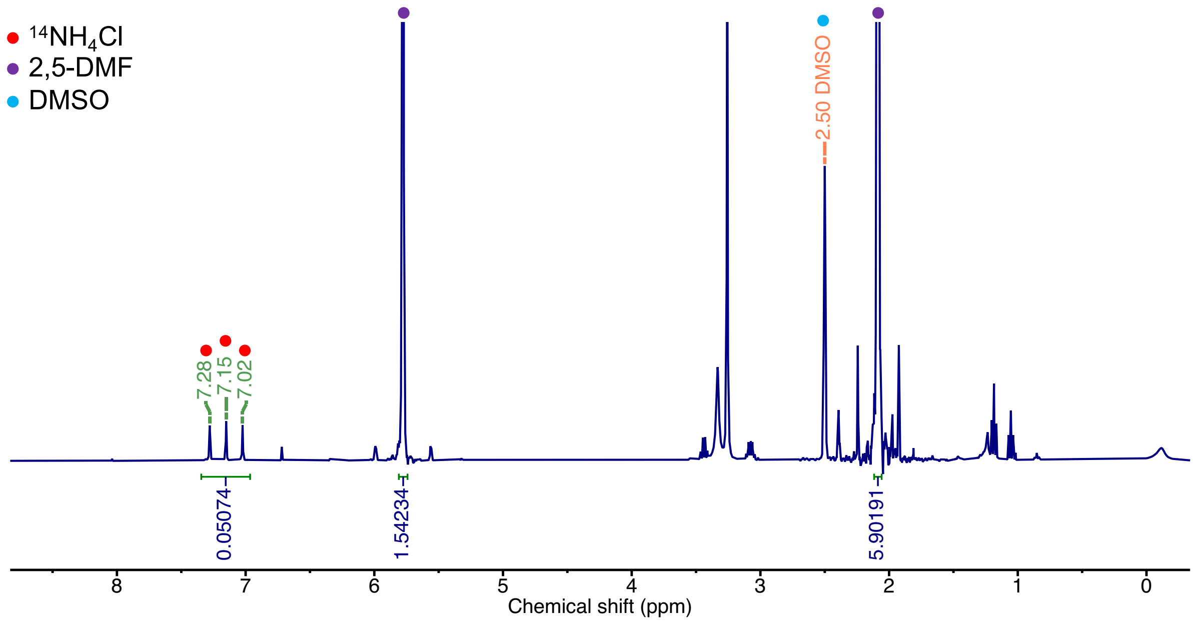

Supplementary Figure 63. Quantitative ^{1}H NMR (400 MHz, DMSO-d$_6$, 298 K) spectrum of an aliquot taken from the HCl-trapped volatile fraction in a catalyst-free acidification reaction using 600 equivalents of KC_8/[Cy_3PH][I] and 1 equivalent of $NaNO_3$ in diethyl ether (Supplementary Table 3, Entry 26). The $^{14}NH_4^+$ 1:1:1 triplet was integrated relative to the methyl protons of 2.5-dimethylfuran (2.15 ppm, s). Spectrum was acquired with a 25-s relaxation delay.

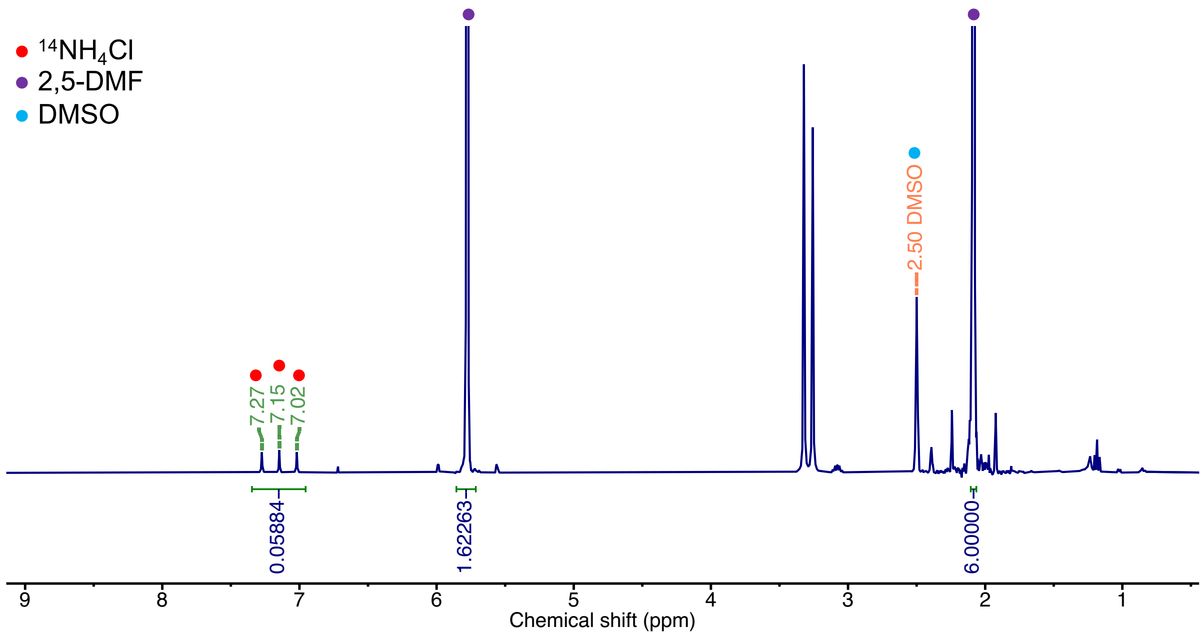

Supplementary Figure 64. Quantitative ^{1}H NMR (400 MHz, DMSO-d$_6$, 298 K) spectrum of an aliquot taken from the HCl-trapped volatile fraction in a catalytic acidification reaction using 600 equivalents of KC_8/[Cy_3PH][I] and 1 equivalent of $NaNO_3$ in diethyl ether (Supplementary Table 3, Entry 27). The $^{14}NH_4^+$ 1:1:1 triplet was integrated relative to the methyl protons of 2.5-dimethylfuran (2.15 ppm, s). Spectrum was acquired with a 25-s relaxation delay

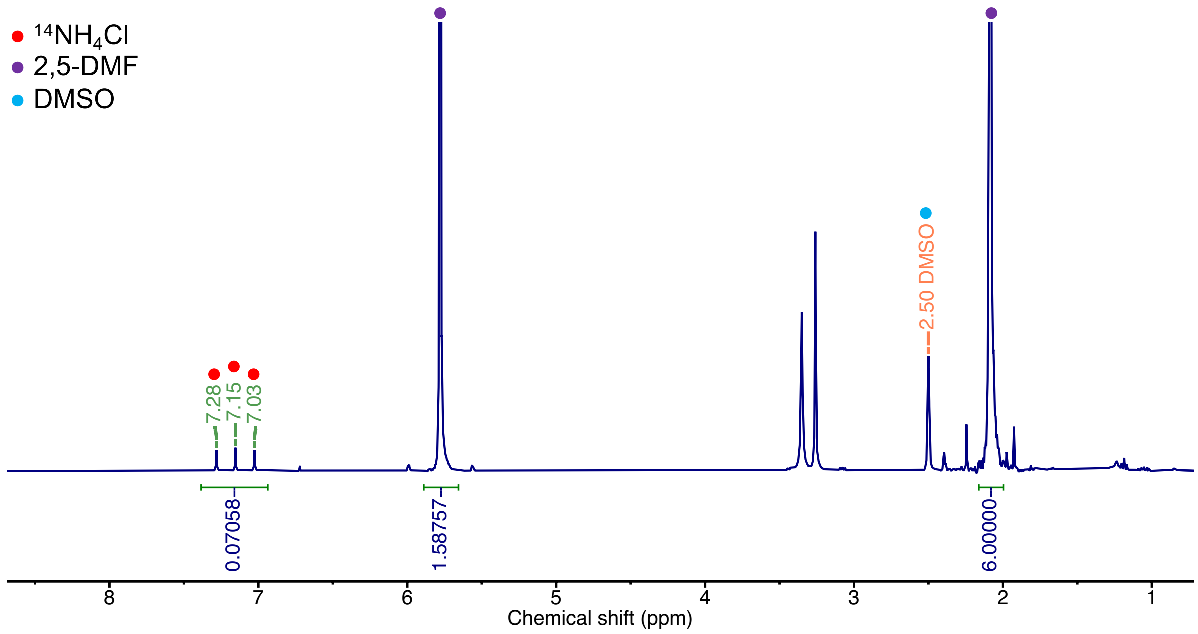

Supplementary Figure 65. Quantitative ^{1}H NMR (400 MHz, DMSO-d$_6$, 298 K) spectrum of an aliquot taken from the HCl-trapped volatile fraction in a catalyst-free acidification reaction using 600 equivalents of KC_8/[Cy_3PH][I] and 1 equivalent of $NaNO_2$ in diethyl ether (Supplementary Table 3, Entry 28). The $^{14}NH_4^+$ 1:1:1 triplet was integrated relative to the methyl protons of 2.5-dimethylfuran (2.15 ppm, s). Spectrum was acquired with a 25-s relaxation delay

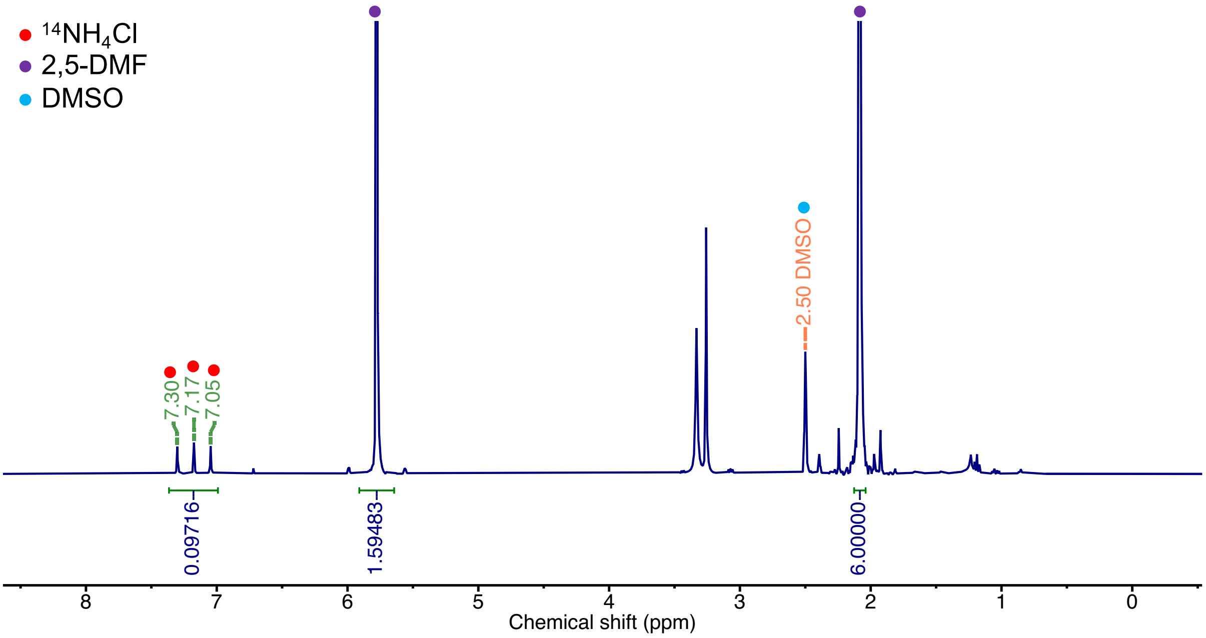

Supplementary Figure 66. Quantitative ^{1}H NMR (400 MHz, DMSO-d$_6$, 298 K) spectrum of an aliquot taken from the HCl-trapped volatile fraction in a catalytic acidification reaction using 600 equivalents of KC_8/[Cy_3PH][I] and 1 equivalent of $NaNO_2$ in diethyl ether (Supplementary Table 3, Entry 29). The $^{14}NH_4^+$ 1:1:1 triplet was integrated relative to the methyl protons of 2.5-dimethylfuran (2.15 ppm, s). Spectrum was acquired with a 25-s relaxation delay

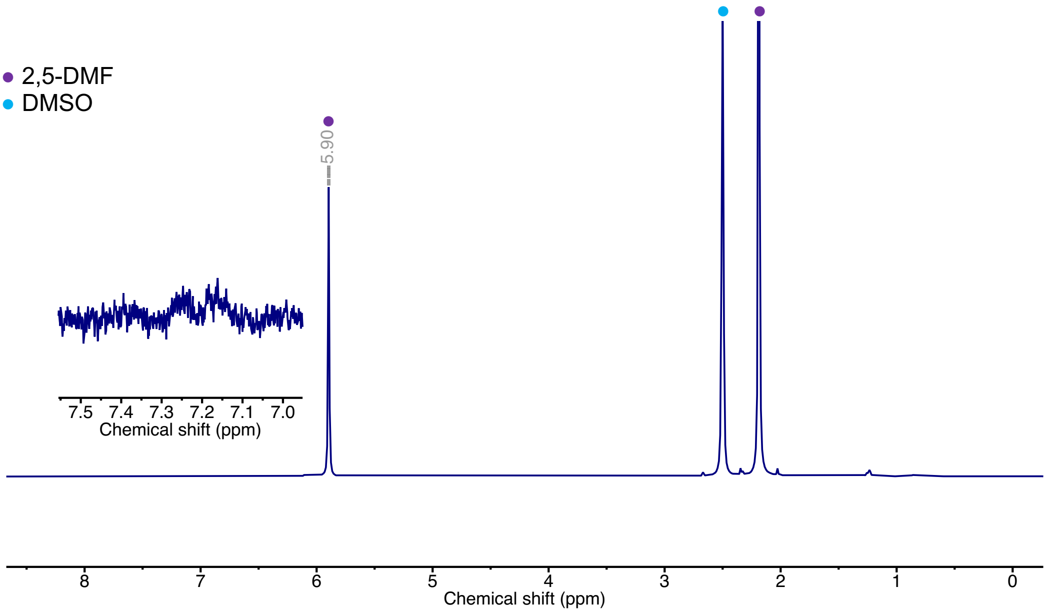

Supplementary Figure 67. Quantitative ^{1}H NMR (400 MHz, DMSO-d$_6$, 298 K) spectrum of an aliquot taken from the HCl-trapped volatile fraction in a blank acidification reaction of **2-crypt**, using 600 equivalents of [Cy_3PH][I] in diethyl ether (Supplementary Table 3, Entry 30). No $^{14}NH_4^+$ peak was detected. Spectrum was acquired with a 25-s relaxation delay.

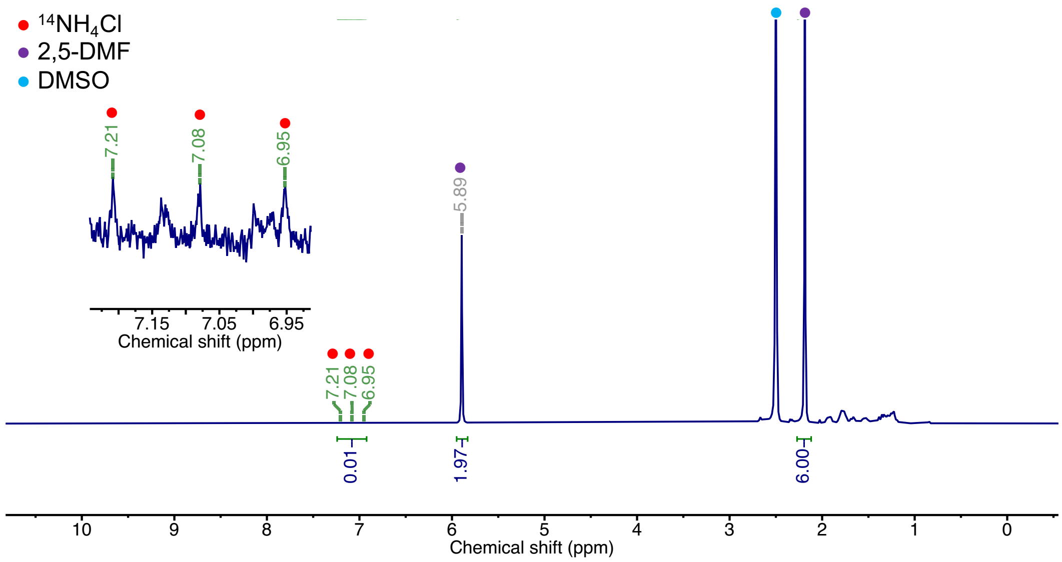

Supplementary Figure 68. Quantitative ^{1}H NMR (400 MHz, DMSO-d_6, 298 K) spectrum of an aliquot taken from the HCl-trapped volatile fraction in a blank acidification reaction of **3**, using 600 equivalents of [Cy_3PH][I] in diethyl ether (Supplementary Table 3, Entry 31). <0.01 eq. of $^{14}NH_4^+$ was detected. Spectrum was acquired with a 25-s relaxation delay.

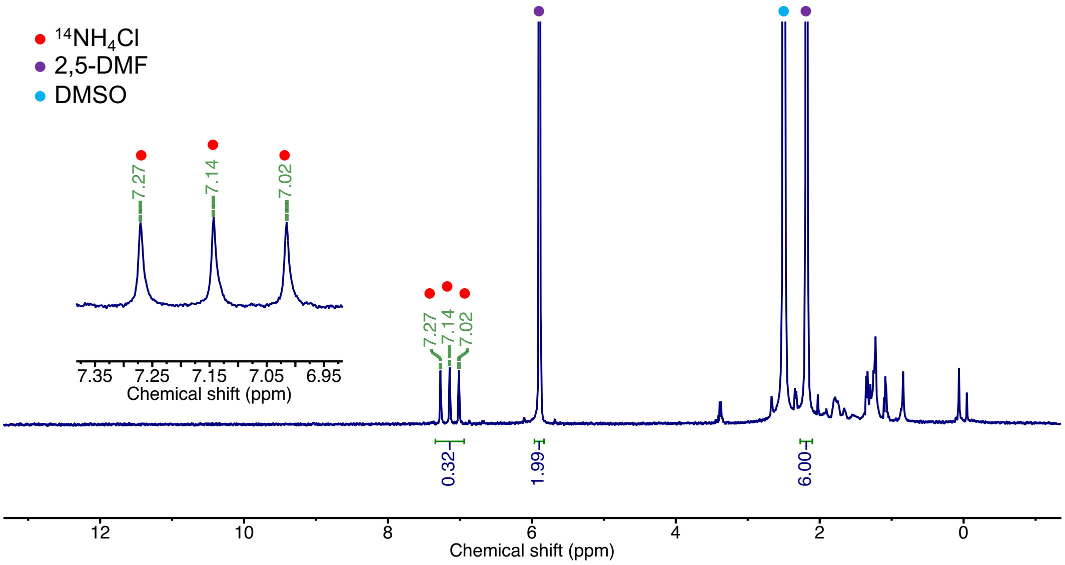

Supplementary Figure 69. Quantitative ^{1}H NMR (400 MHz, DMSO-d_6, 298 K) spectrum of an aliquot taken from the HCl-trapped volatile fraction in a blank acidification reaction of **4**, using 600 equivalents of [Cy_3PH][I] in diethyl ether (Supplementary Table 3, Entry 32). The $^{14}NH_4^+$ 1:1:1 triplet was integrated relative to the methyl protons of 2.5-dimethylfuran (2.15 ppm, s). Spectrum was acquired with a 25-s relaxation delay.

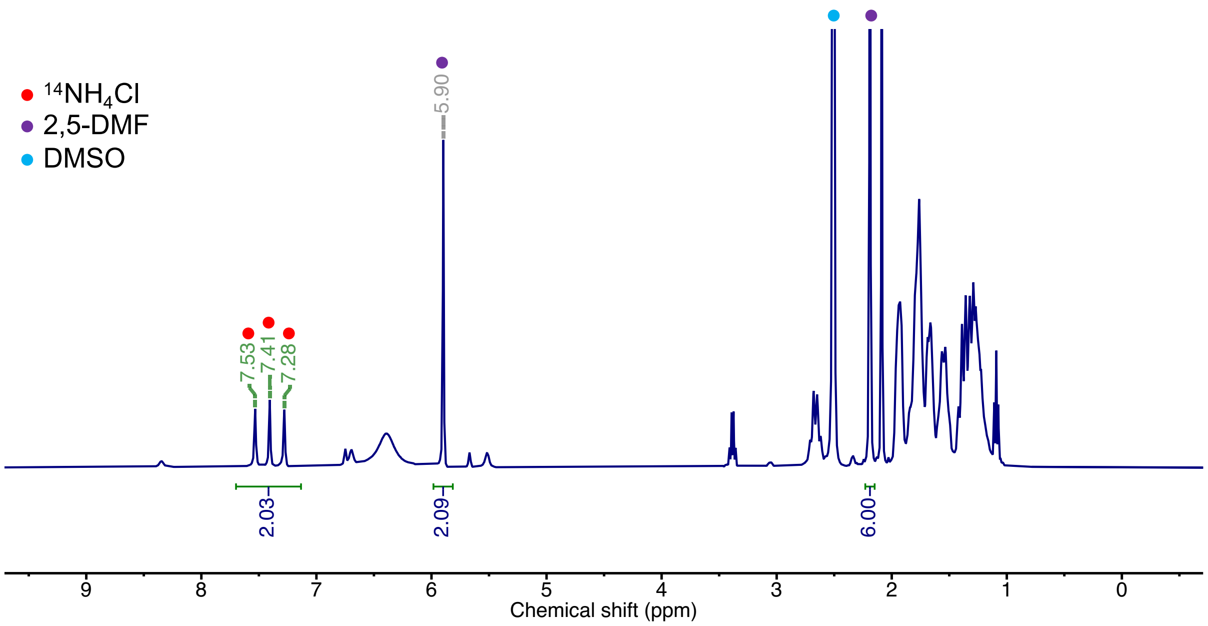

Supplementary Figure 70. Quantitative ^{1}H NMR (400 MHz, DMSO-d$_6$, 298 K) spectrum of an aliquot taken from the HCl-trapped volatile fraction in a blank acidification reaction of **2-crypt**, using 600 equivalents of [Cy$_3$PH][I] and 600 equivalents of RbC$_8$ in diethyl ether under argon (Supplementary Table 3, Entry 33). The $^{14}NH_4^+$ 1:1:1 triplet was integrated relative to the methyl protons of 2.5-dimethylfuran (2.15 ppm, s). Spectrum was acquired with a 25-s relaxation delay.

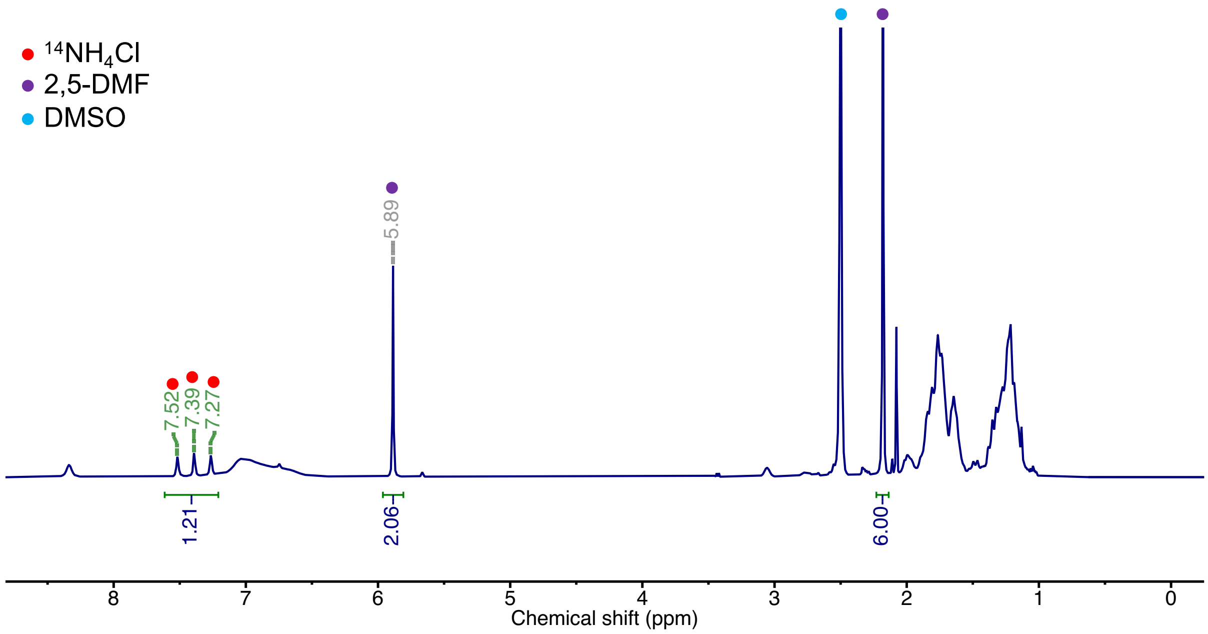

Supplementary Figure 71. Quantitative ^{1}H NMR (400 MHz, DMSO-d$_6$, 298 K) spectrum of an aliquot taken from the HCl-trapped volatile fraction in a blank acidification reaction of **3**, using 600 equivalents of [Cy$_3$PH][I] and 600 equivalents of RbC$_8$ in diethyl ether under argon (Supplementary Table 3, Entry 34). The $^{14}NH_4^+$ 1:1:1 triplet was integrated relative to the methyl protons of 2.5-dimethylfuran (2.15 ppm, s). Spectrum was acquired with a 25-s relaxation delay.

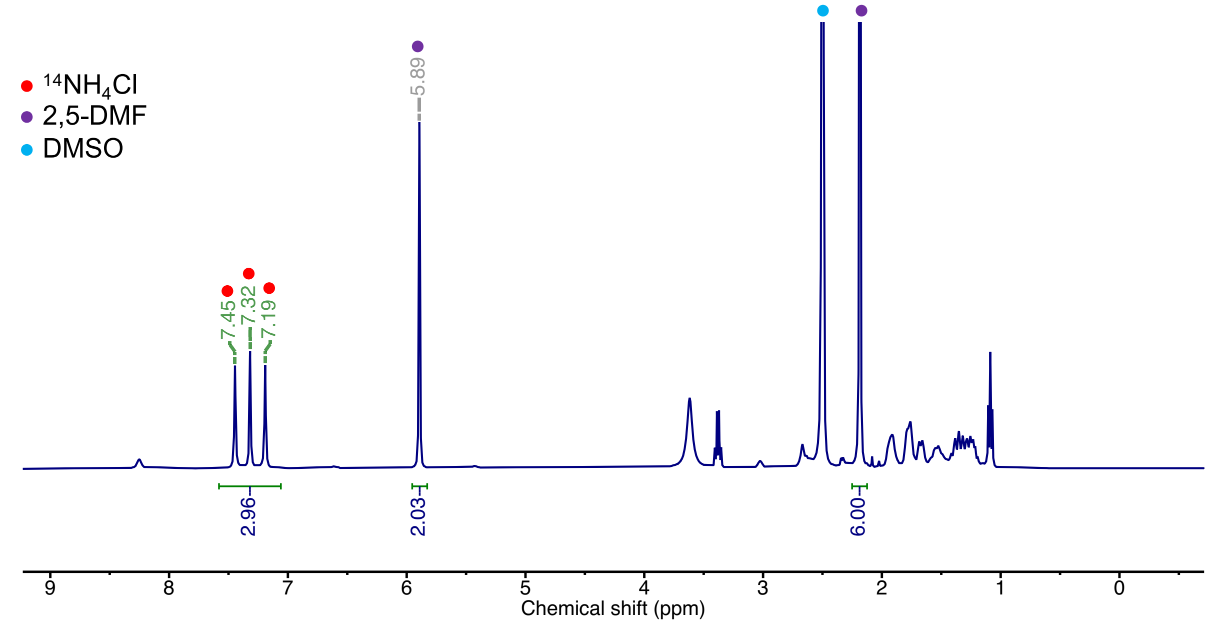

Supplementary Figure 72. Quantitative ^{1}H NMR (400 MHz, DMSO-d_6, 298 K) spectrum of an aliquot taken from the HCl-trapped volatile fraction in a blank acidification reaction of **4**, using 600 equivalents of $[Cy_3PH][I]$ and 600 equivalents of RbC_8 in diethyl ether under argon (Supplementary Table 3, Entry 35). The $^{14}NH_4^+$ 1:1:1 triplet was integrated relative to the methyl protons of 2.5-dimethylfuran (2.15 ppm, s). Spectrum was acquired with a 25-s relaxation delay.

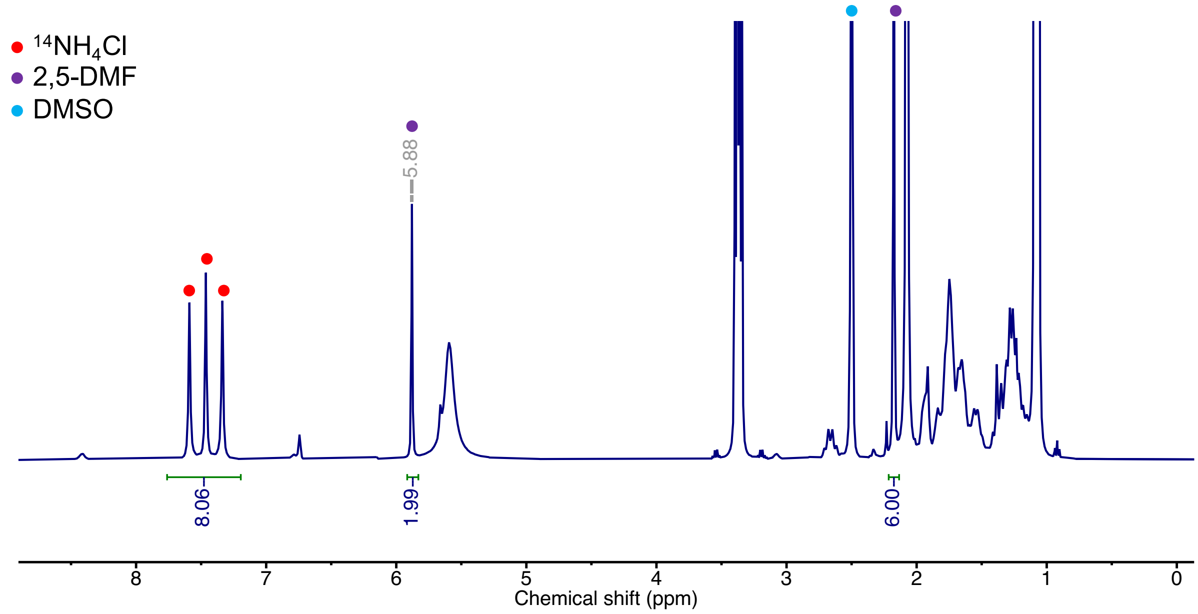

Supplementary Figure 73. Quantitative ^{1}H NMR (400 MHz, DMSO-d_6, 298 K) spectrum of an aliquot taken from the HCl-trapped volatile fraction in a catalytic acidification reaction of **4**, using 600 equivalents of $[Cy_3PH][I]$ and 600 equivalents of RbC_8 in diethyl ether under dinitrogen (Table 1, Entry 25). The $^{14}NH_4^+$ 1:1:1 triplet was integrated relative to the methyl protons of 2.5-dimethylfuran (2.15 ppm, s). Spectrum was acquired with a 25-s relaxation delay.

Miscellaneous

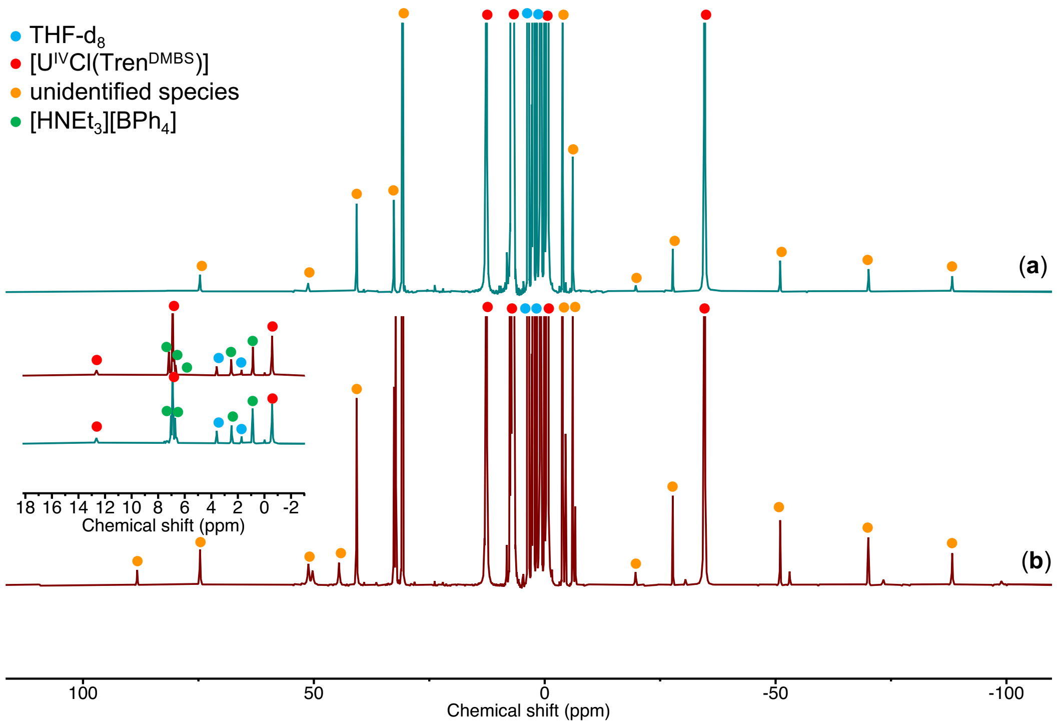

Supplementary Figure 74. ^{1}H NMR (400 MHz, THF-d_8, 298 K) spectrum obtained of after the addition of 1 equivalent of $[HNEt_3][BPh_4]$ to $[U^{IV}Cl(Tren^{DMBS})]$ after 1 (**a**) and 15 hours (**b**) at room temperature, resulting in appearance of unidentified species.

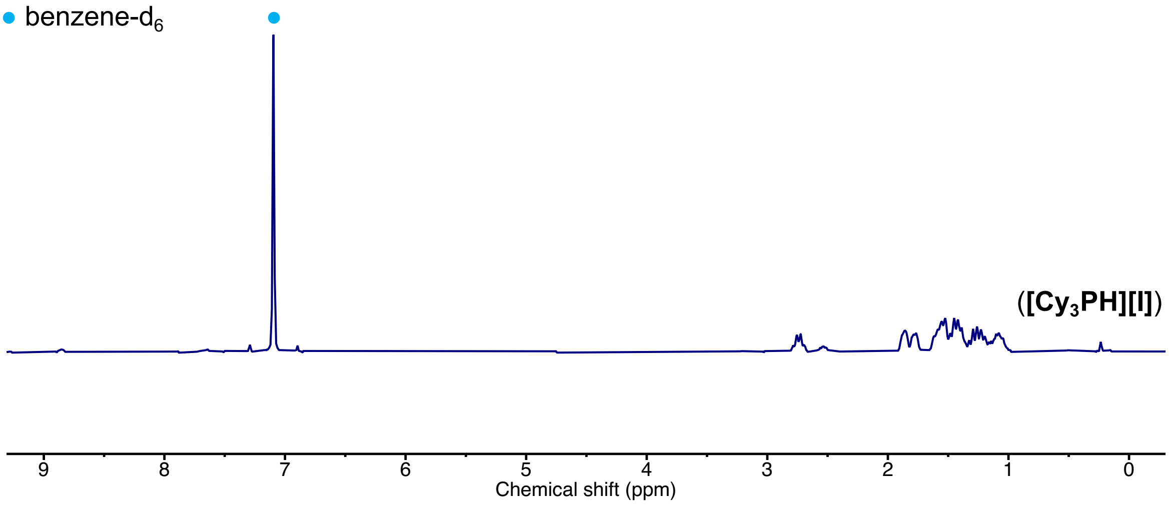

Supplementary Figure 75. ^{1}H NMR (400 MHz, C_6D_6, 298 K) spectrum obtained of $[Cy_3PH][I]$. Due to the poor solubility of this compound in C_6D_6, the ^{1}H NMR can only be tentatively assigned.

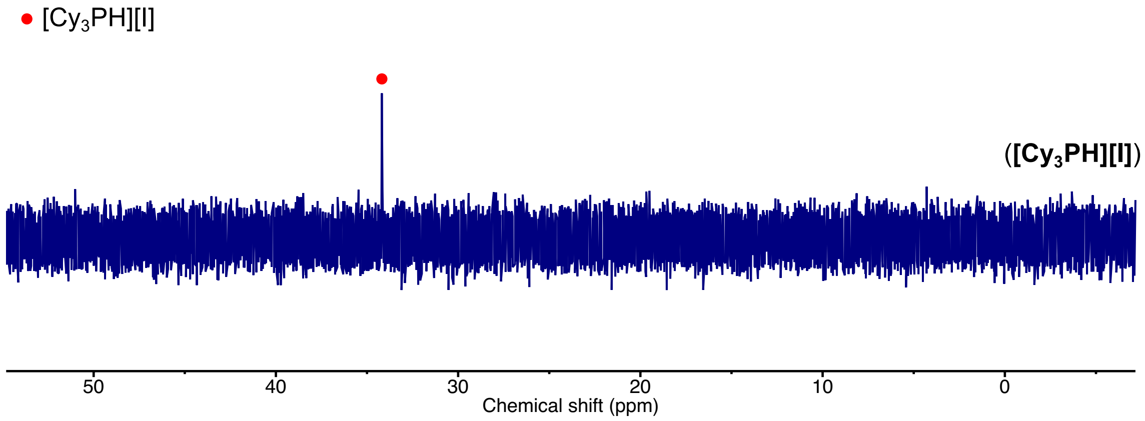

Supplementary Figure 76. ^{31}P NMR (100.61 MHz, C_6D_6, 298 K) spectrum of $[Cy_3PH][I]$.

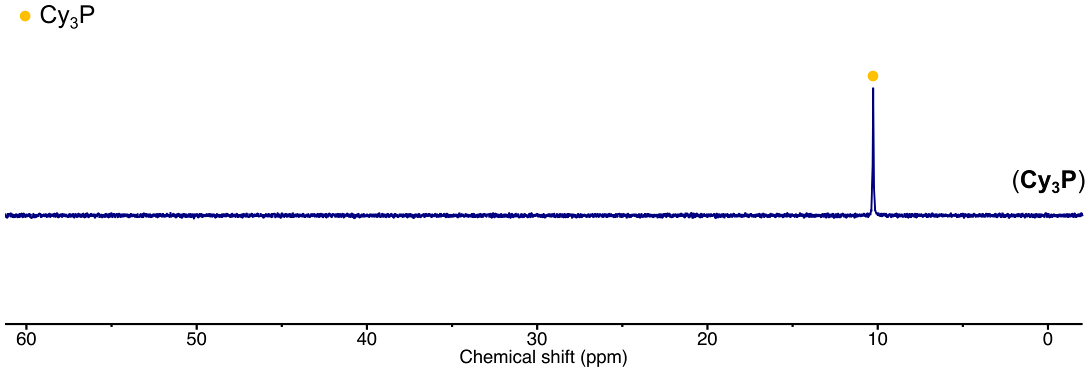

Supplementary Figure 77. ^{31}P NMR (100.61 MHz, C_6D_6, 298 K) spectrum of PCy_3.

UV-Vis-NIR Spectra

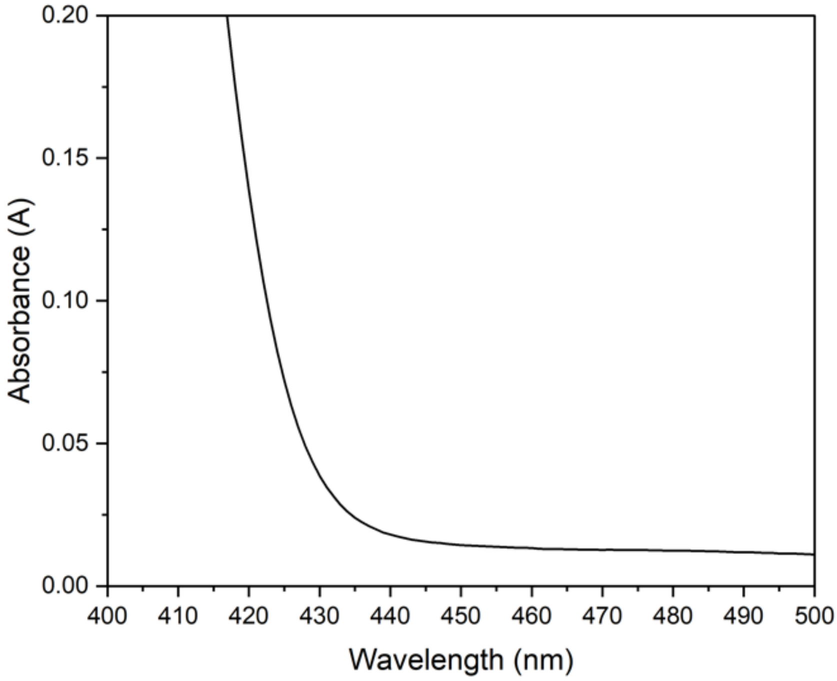

Supplementary Figure 78. Zoom-in of a UV-Vis-NIR spectrum of an aliquot taken from the HCl-trapped volatile fraction in a catalyst-free acidification reaction using 600 equivalents of KC_8/[Cy_3PH][I] in diethyl ether (Table 1, Entry 5). The lack of the characteristic peak at 458 nm indicates that there is no N_2H_4 present.

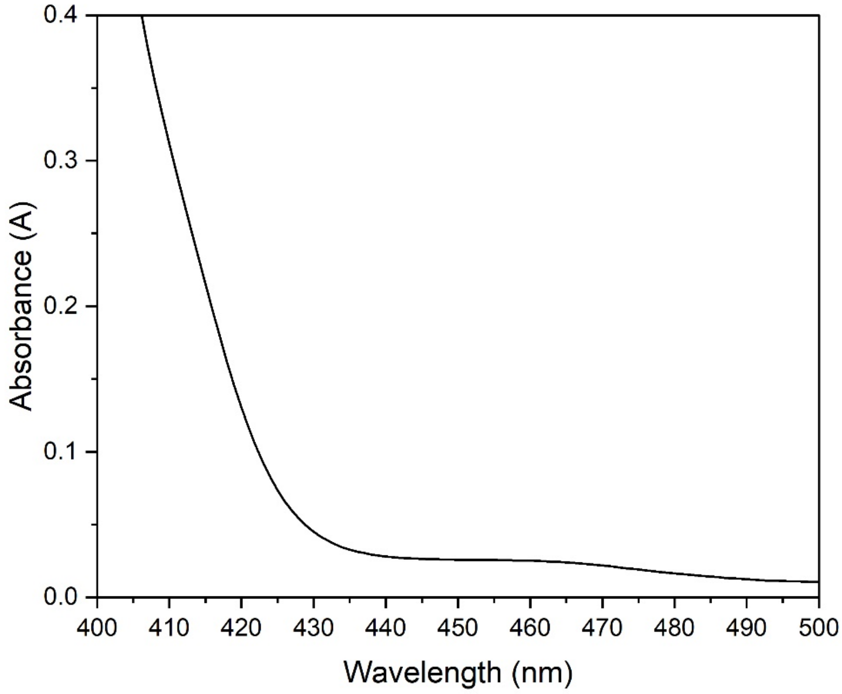

Supplementary Figure 79. Zoom-in of a UV-Vis-NIR spectrum of an aliquot taken from the residues remaining in bulb A in a catalyst-free acidification reaction using 600 equivalents of KC_8/[Cy_3PH][I] in diethyl ether (Table 1, Entry 5). The very small characteristic peak at 458 nm indicates the presence of N_2H_4.

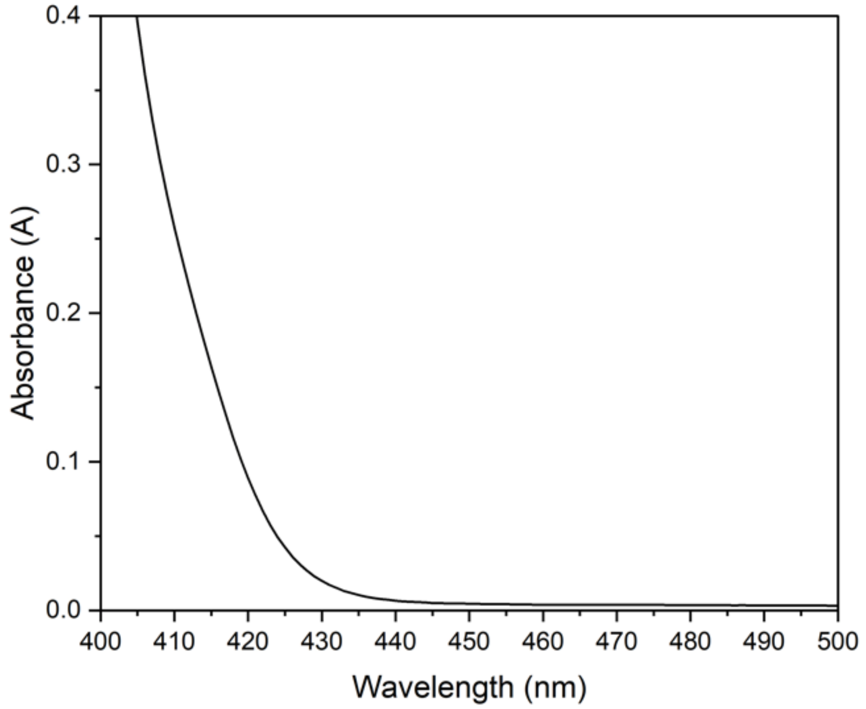

Supplementary Figure 80. Zoom-in of a UV-Vis-NIR spectrum of an aliquot taken from the HCl-trapped volatile fraction in a catalyst-free acidification reaction using 600 equivalents of KC_8/[Cy_3PH][I] in diethyl ether (Table 1, Entry 6). The lack of the characteristic peak at 458 nm indicates that there is no N_2H_4 present.

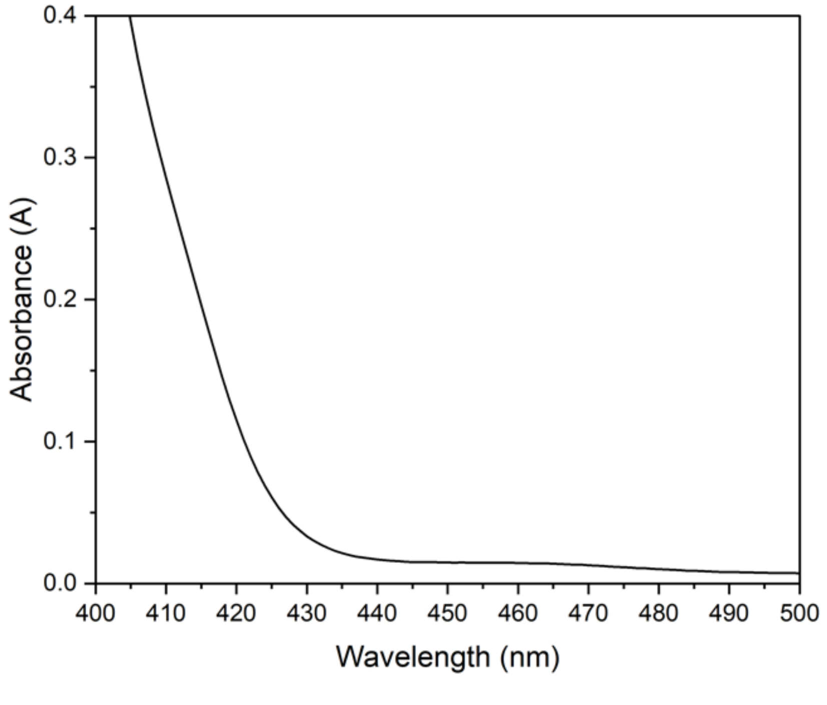

Supplementary Figure 81. Zoom-in of a UV-Vis-NIR spectrum of an aliquot taken from the residues remaining in bulb A in a catalyst-free acidification reaction using 600 equivalents of KC_8/[Cy_3PH][I] in diethyl ether (Table 1, Entry 6). The lack of the characteristic peak at 458 nm indicates that there is no N_2H_4 present.

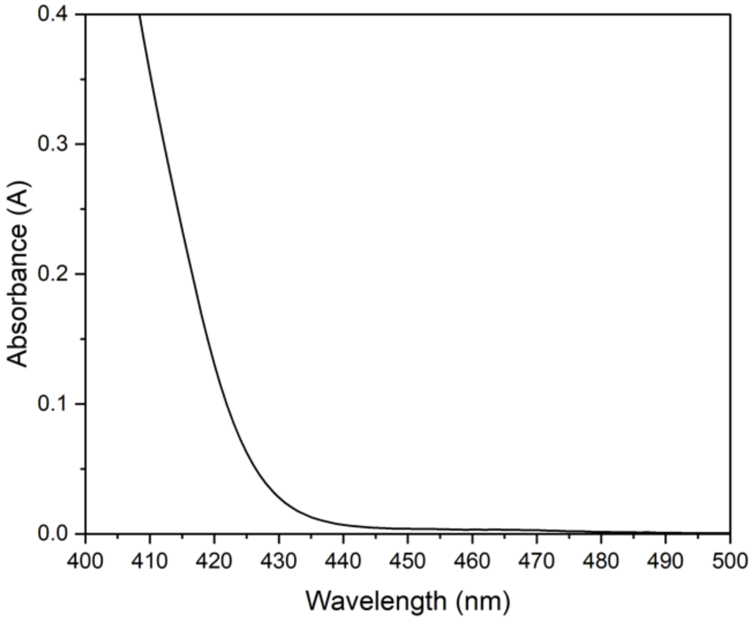

Supplementary Figure 82. Zoom-in of a UV-Vis-NIR spectrum of an aliquot taken from the HCl-trapped volatile fraction in a catalytic acidification reaction using 600 equivalents of KC_8/[Et_3NH][BPh_4] in diethyl ether (Table 1, Entry 10). The lack of the characteristic peak at 458 nm indicates that there is no N_2H_4 present.

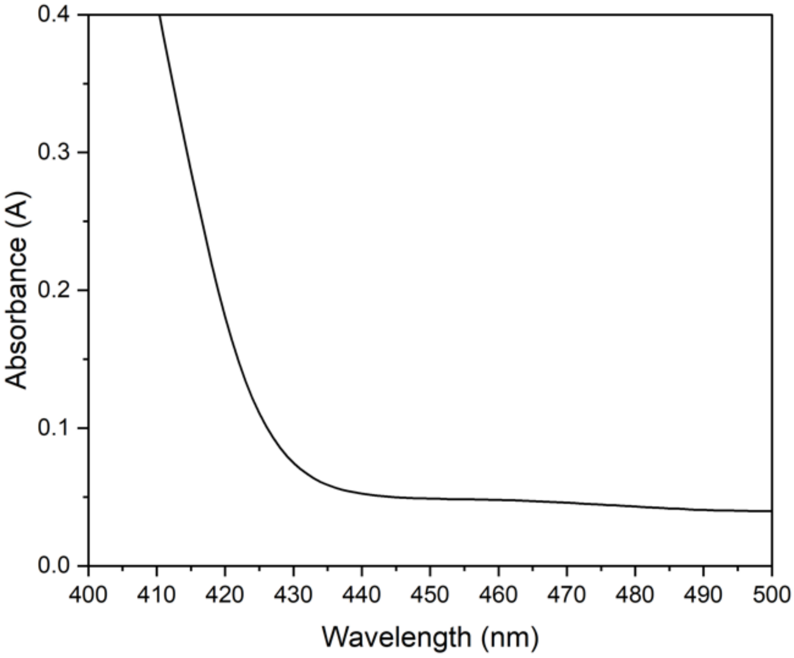

Supplementary Figure 83. Zoom-in of a UV-Vis-NIR spectrum of an aliquot taken from the residues remaining in bulb A in a catalytic acidification reaction using 600 equivalents of KC_8/[Et_3NH][BPh_4] in diethyl ether (Table 1, Entry 10). The lack of the characteristic peak at 458 nm indicates that there is no N_2H_4 present.

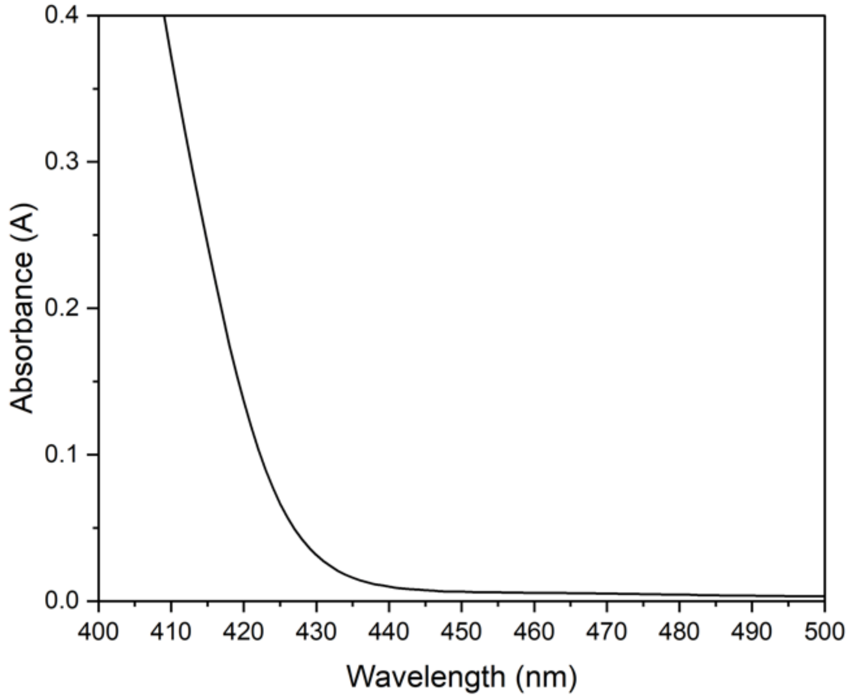

Supplementary Figure 84. Zoom-in of a UV-Vis-NIR spectrum of an aliquot taken from the HCl-trapped volatile fraction in a catalytic acidification reaction using 600 equivalents of KC_8/[Et_3NH][Cl] in diethyl ether (Table 1, Entry 11). The lack of the characteristic peak at 458 nm indicates that there is no N_2H_4 present.

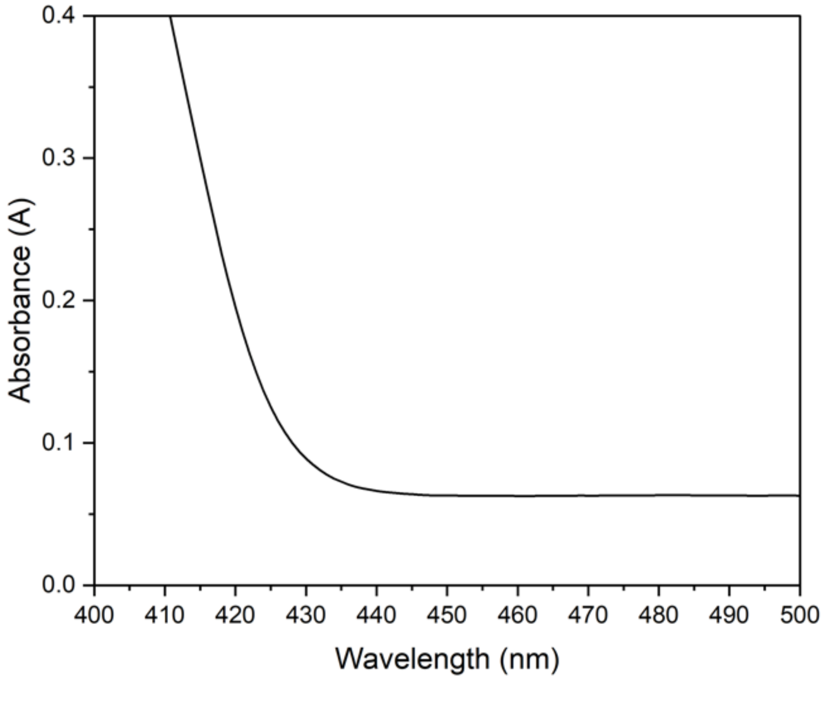

Supplementary Figure 85. Zoom-in of a UV-Vis-NIR spectrum of an aliquot taken from the residues remaining in bulb A in a catalytic acidification reaction using 600 equivalents of KC_8/[Et_3NH][Cl] in diethyl ether (Table 1, Entry 11). The lack of characteristic peak at 458 nm indicates that there is no N_2H_4.

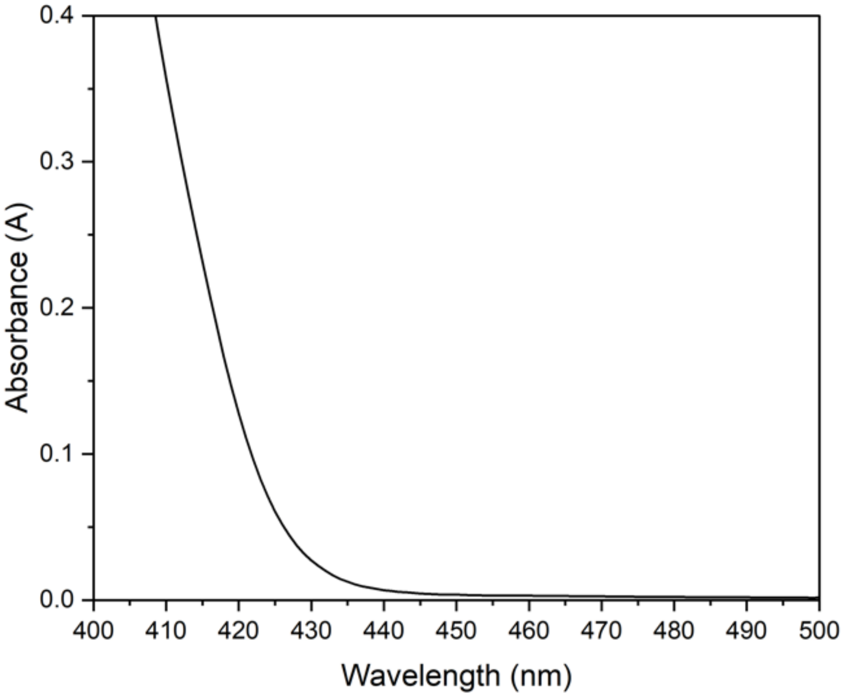

Supplementary Figure 86. Zoom-in of a UV-Vis-NIR spectrum of an aliquot taken from the HCl-trapped volatile fraction in a catalytic acidification reaction using 600 equivalents of KC_8/[Et_3NH][I] in diethyl ether (Table 1, Entry 12). The lack of the characteristic peak at 458 nm indicates that there is no N_2H_4 present.

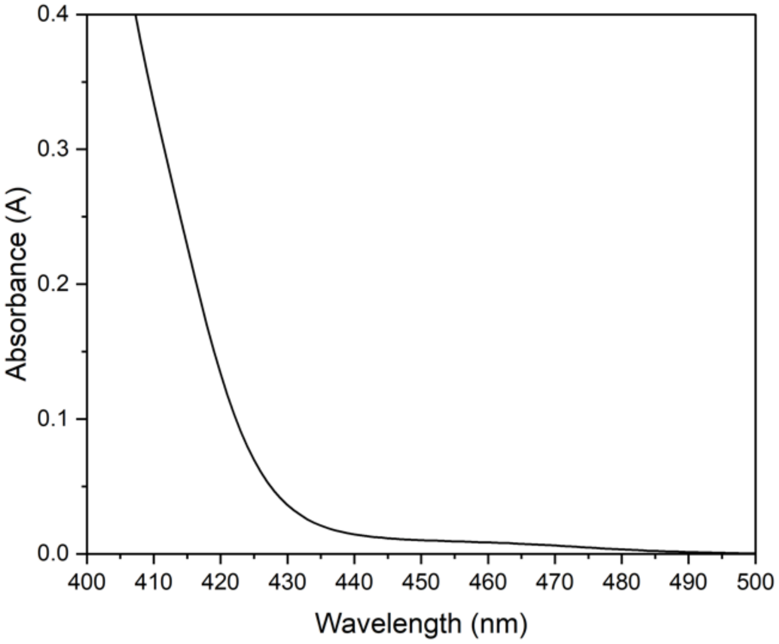

Supplementary Figure 87. Zoom-in of a UV-Vis-NIR spectrum of an aliquot taken from the residues remaining in bulb A in a catalytic acidification reaction using 600 equivalents of KC_8/[Et_3NH][I] in diethyl ether (Table 1, Entry 12). The lack of characteristic peak at 458 nm indicates that there is no N_2H_4 present.

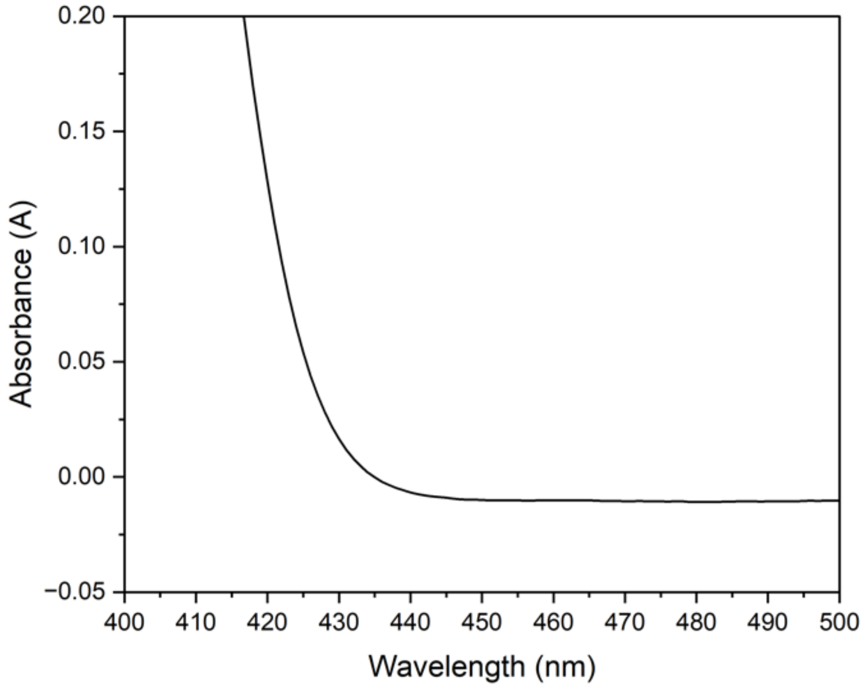

Supplementary Figure 88. Zoom-in of a UV-Vis-NIR spectrum of an aliquot taken from the HCl-trapped volatile fraction in a catalytic acidification reaction using 600 equivalents of Na/[Cy_3PH][I] in diethyl ether (Table 1, Entry 14). The lack of the characteristic peak at 458 nm indicates that there is no N_2H_4 present.

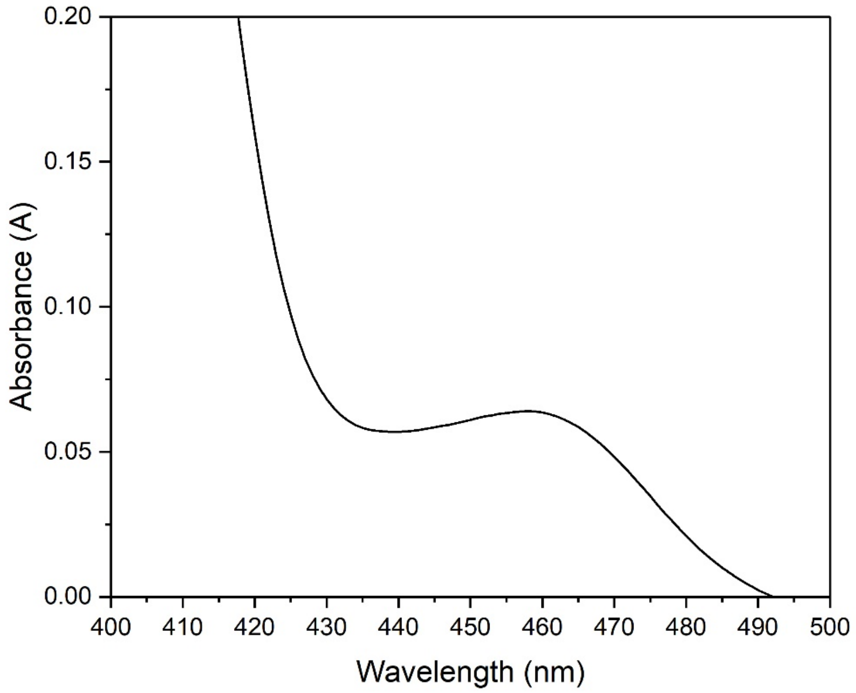

Supplementary Figure 89. Zoom-in of a UV-Vis-NIR spectrum of an aliquot taken from the residues remaining in bulb A in a catalytic acidification reaction using 600 equivalents of Na/[Cy_3PH][I] in diethyl ether (Table 1, Entry 14). The characteristic peak at 458 nm indicates the presence of N_2H_4.

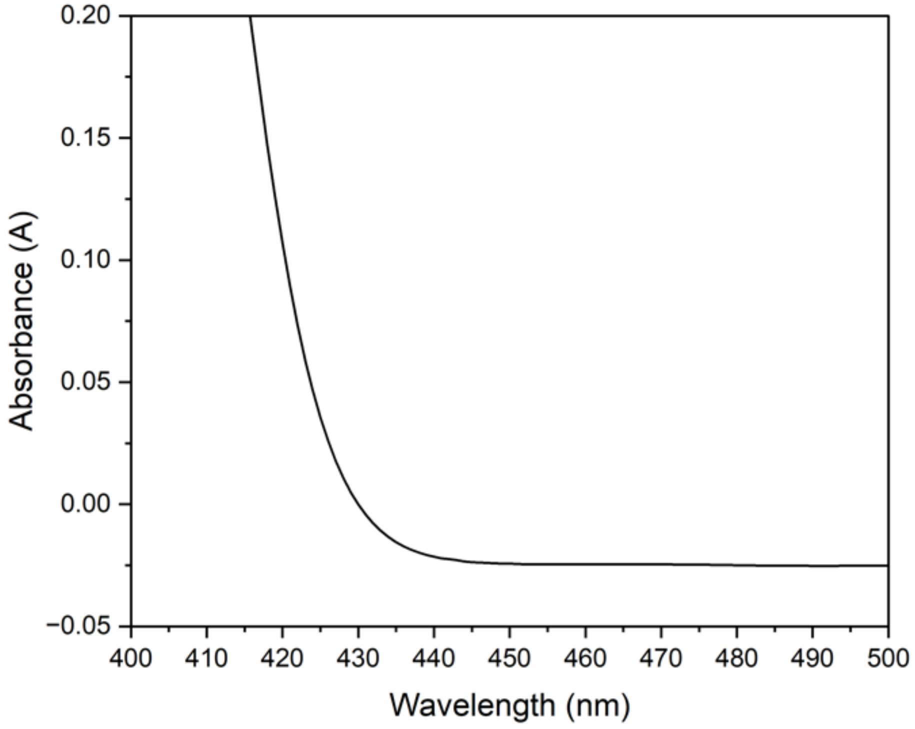

Supplementary Figure 90. Zoom-in of a UV-Vis-NIR spectrum of an aliquot taken from the HCl-trapped volatile fraction in a catalytic acidification reaction using 600 equivalents of K/[Cy_3PH][I] in diethyl ether (Table 1, Entry 15). The lack of the characteristic peak at 458 nm indicates that there is no N_2H_4 present.

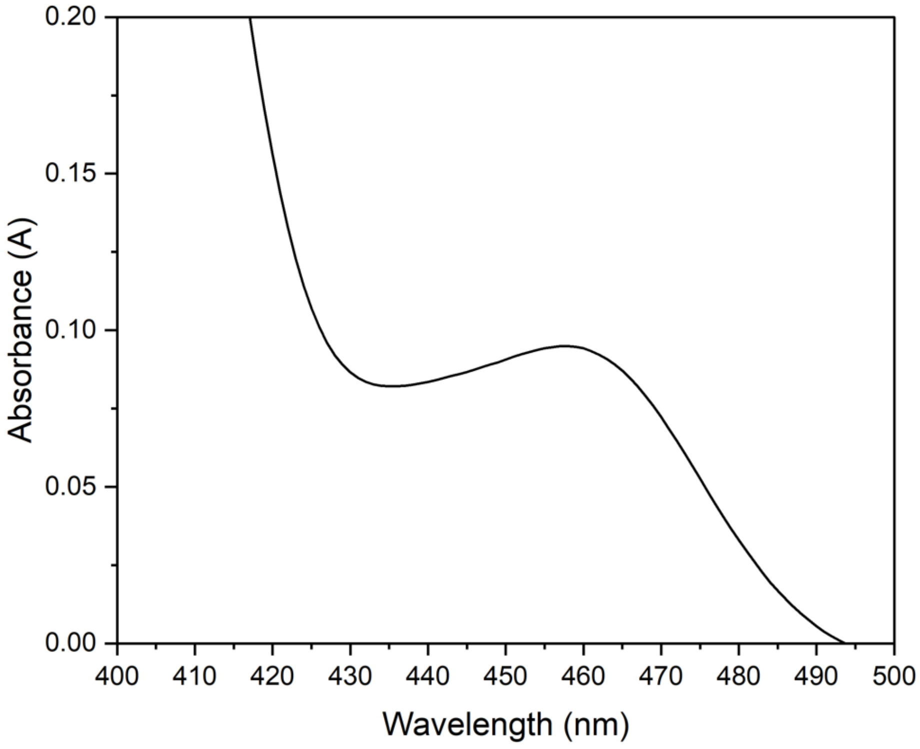

Supplementary Figure 91. Zoom-in of a UV-Vis-NIR spectrum of an aliquot taken from the residues remaining in bulb A in a catalytic acidification reaction using 600 equivalents of K/[Cy_3PH][I] in diethyl ether (Table 1, Entry 15). The characteristic peak at 458 nm indicates the presence of N_2H_4.

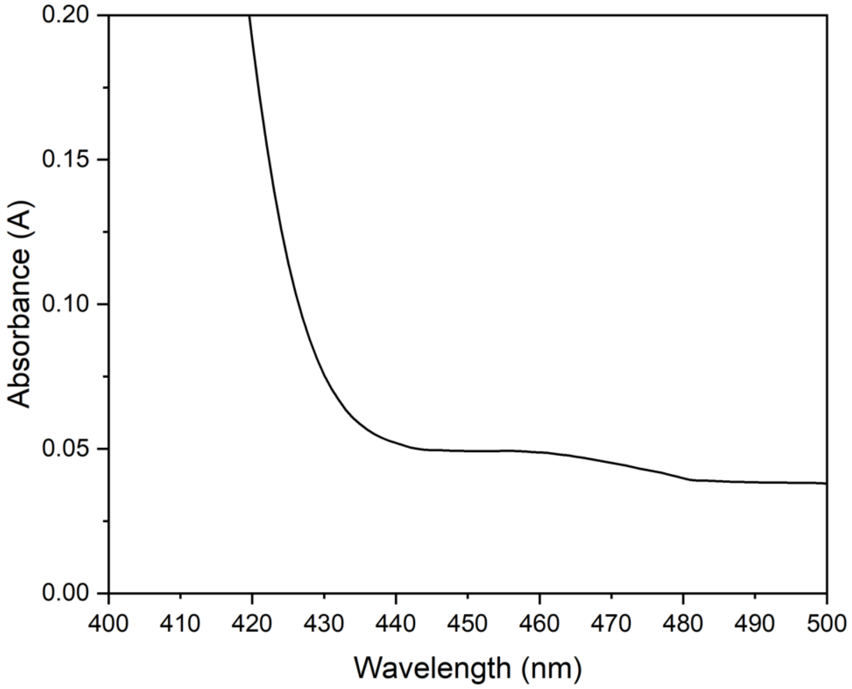

Supplementary Figure 92. Zoom-in of a UV-Vis-NIR spectrum of an aliquot taken from the HCl-trapped volatile fraction in a catalytic acidification reaction using 173 equivalents of KC_8/[Cy_3PH][I] in diethyl ether (Table 1, Entry 16). The characteristic peak at 458 nm indicates the presence of N_2H_4.

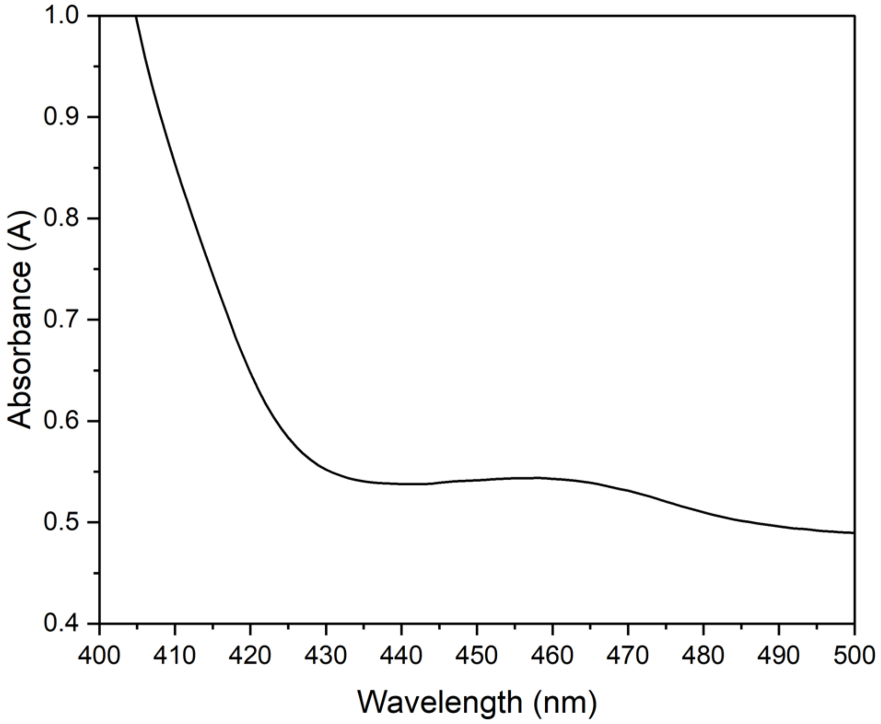

Supplementary Figure 93. Zoom-in of a UV-Vis-NIR spectrum of an aliquot taken from the residues remaining in bulb A in a catalytic acidification reaction using 173 equivalents of KC_8/[Cy_3PH][I] in diethyl ether (Table 1, Entry 16). The characteristic peak at 458 nm indicates the presence of N_2H_4.

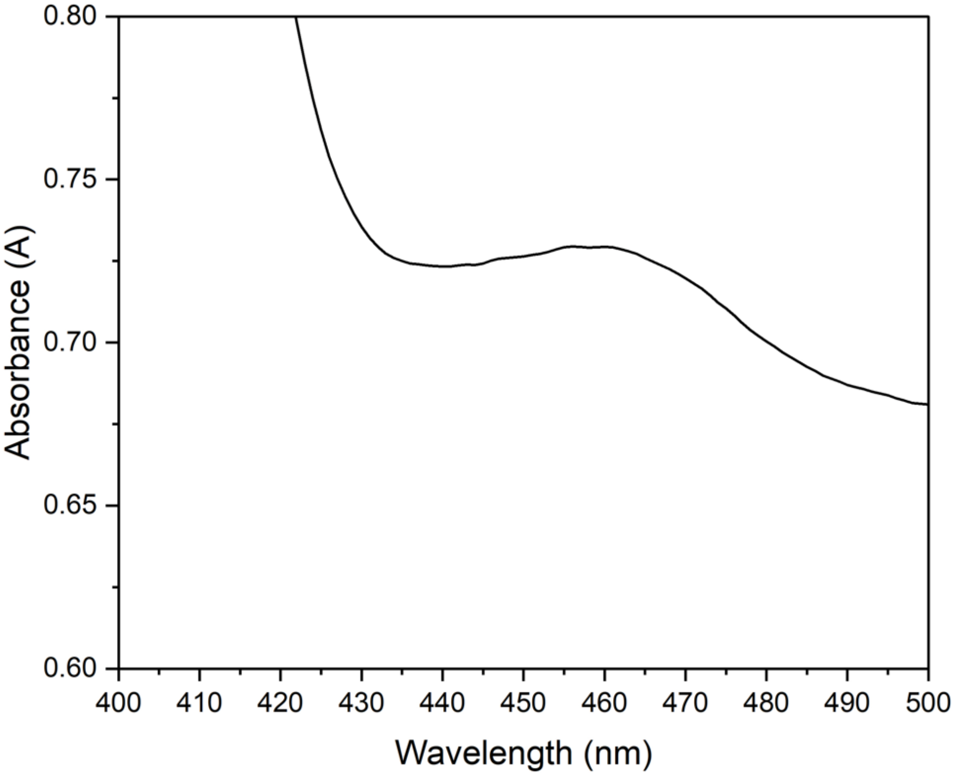

Supplementary Figure 94. Zoom-in of a UV-Vis-NIR spectrum of an aliquot taken from the HCl-trapped volatile fraction in a catalytic acidification reaction using 173 equivalents of KC_8/[Cy_3PH][I] in diethyl ether at a gauge pressure of 1.2 bar (Table 1, Entry 17). The characteristic peak at 458 nm indicates the presence of N_2H_4.

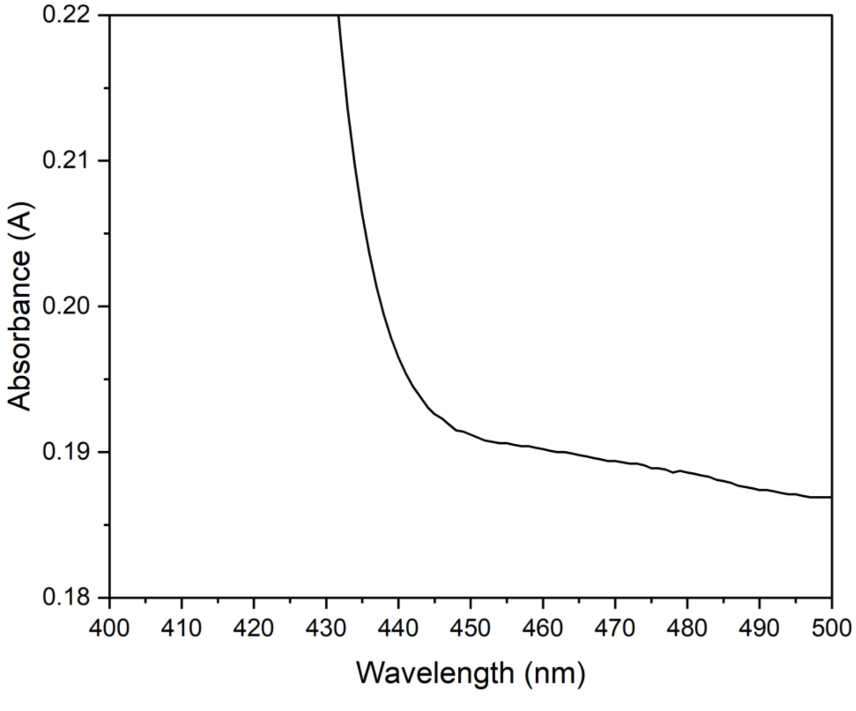

Supplementary Figure 95. Zoom-in of a UV-Vis-NIR spectrum of an aliquot taken from the residues remaining in bulb A in a catalytic acidification reaction using 173 equivalents of KC_8/[Cy_3PH][I] in diethyl ether at a gauge pressure of 1.2 (Table 1, Entry 17). The lack of the characteristic peak at 458 nm indicates that there is no N_2H_4 present.

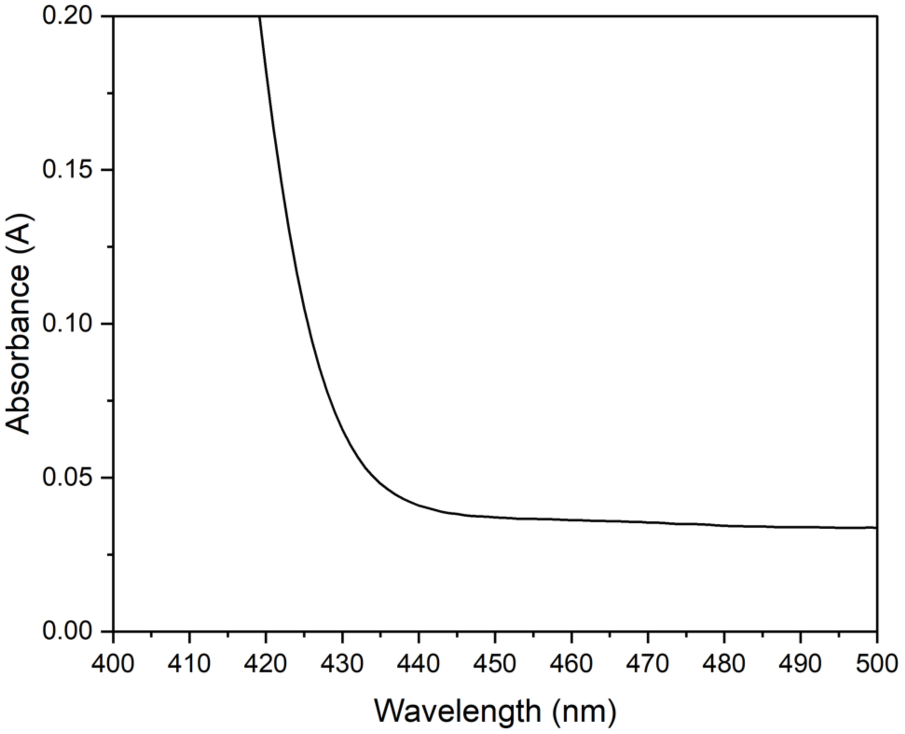

Supplementary Figure 96. Zoom-in of a UV-Vis-NIR spectrum of an aliquot taken from the HCl-trapped volatile fraction in a catalytic acidification reaction using 300 equivalents of KC_8/[Cy_3PH][I] in diethyl ether (Table 1, Entry 18). The lack of the characteristic peak at 458 nm indicates that there is no N_2H_4 present.

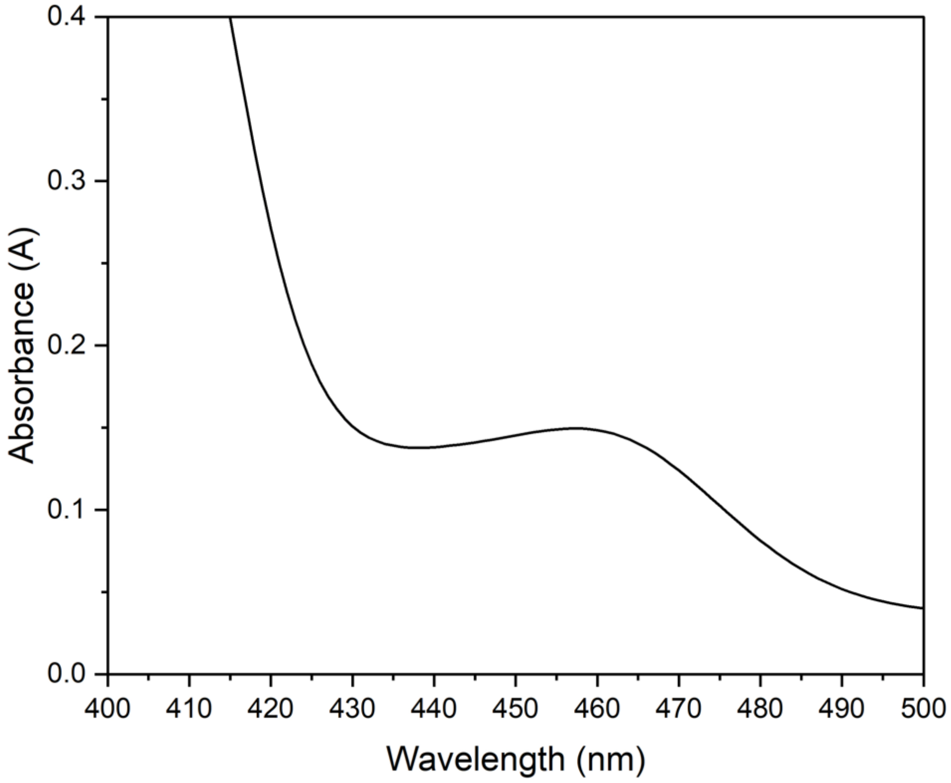

Supplementary Figure 97. Zoom-in of a UV-Vis-NIR spectrum of an aliquot taken from the residues remaining in bulb A in a catalytic acidification reaction using 300 equivalents of KC_8/[Cy_3PH][I] in diethyl ether (Table 1, Entry 18). The characteristic peak at 458 nm indicates the presence of N_2H_4.

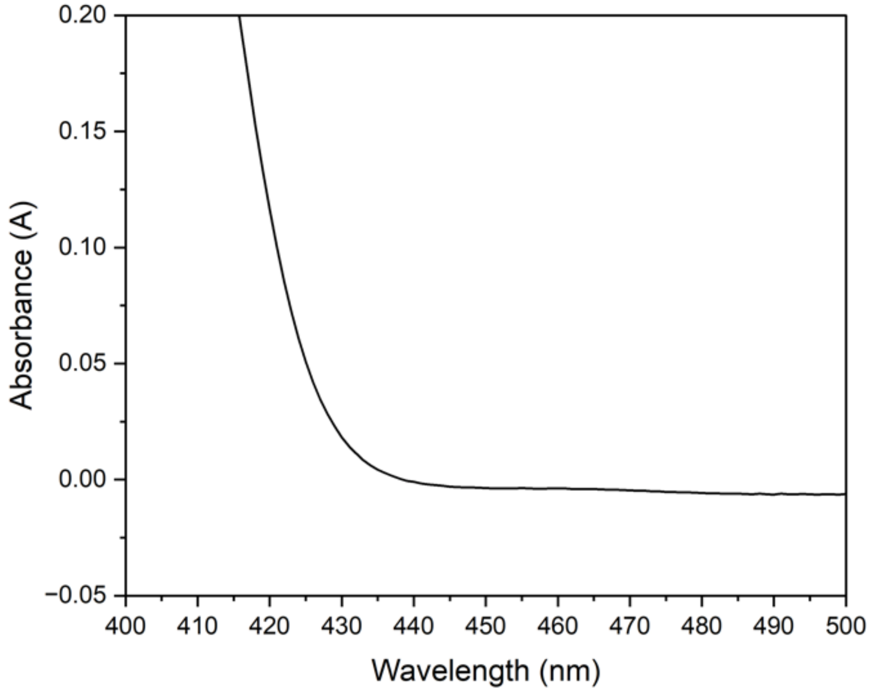

Supplementary Figure 98. Zoom-in of a UV-Vis-NIR spectrum of an aliquot taken from the HCl-trapped volatile fraction in a catalytic acidification reaction using 600 equivalents of KC_8/[Cy_3PH][I] in diethyl ether (Table 1, Entry 19). The lack of the characteristic peak at 458 nm indicates that there is no N_2H_4 present.

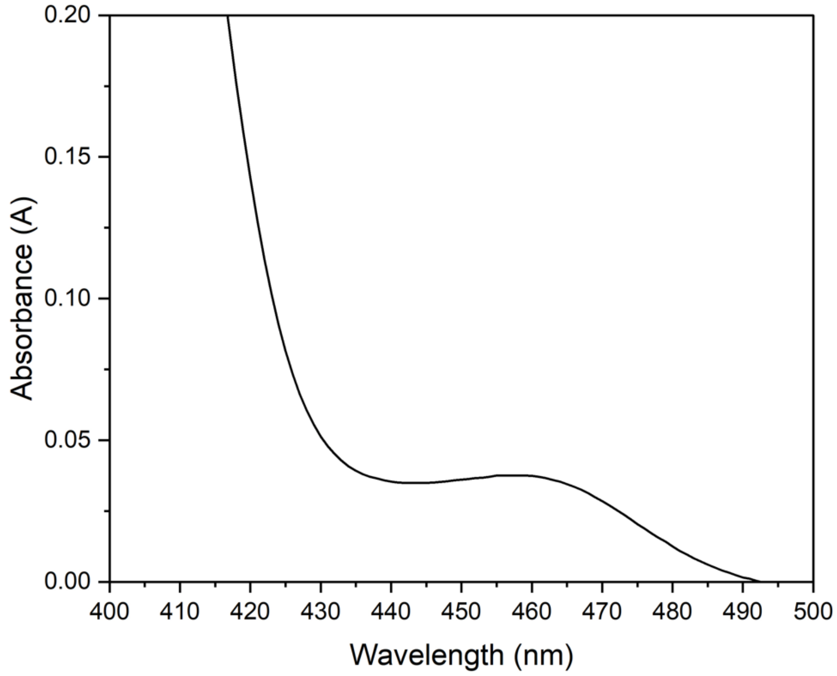

Supplementary Figure 99. Zoom-in of a UV-Vis-NIR spectrum of an aliquot taken from the residues remaining in bulb A in a catalytic acidification reaction using 600 equivalents of KC_8/[Cy_3PH][I] in diethyl ether (Table 1, Entry 19). The characteristic peak at 458 nm indicates the presence of N_2H_4.

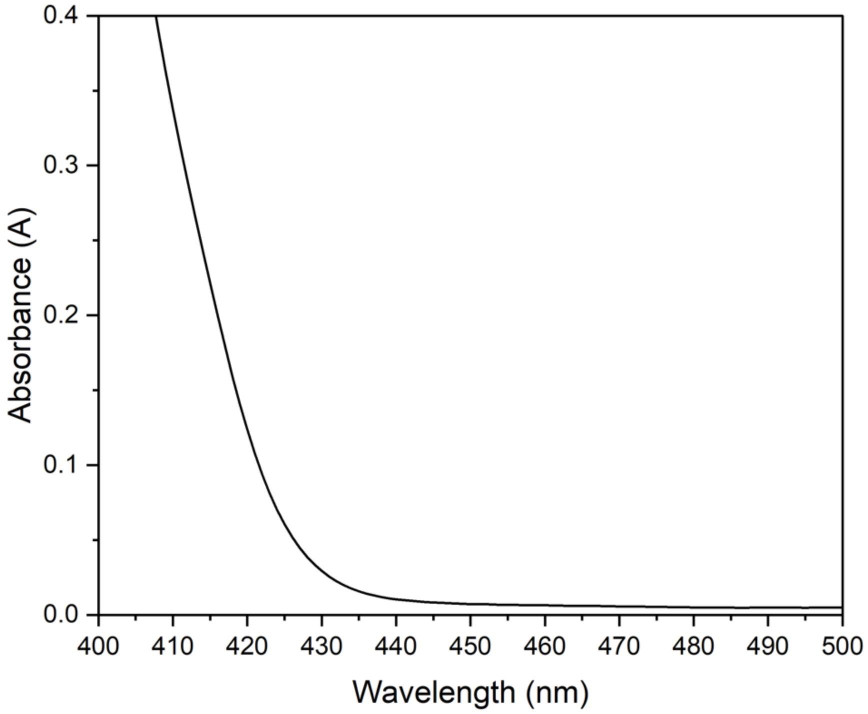

Supplementary Figure 100. Zoom-in of a UV-Vis-NIR spectrum of an aliquot taken from the HCl-trapped volatile fraction in a catalytic acidification reaction using 600 equivalents of Rb/[Cy_3PH][I] in diethyl ether (Table 1, Entry 20). The lack of the characteristic peak at 458 nm indicates that there is no N_2H_4 present.

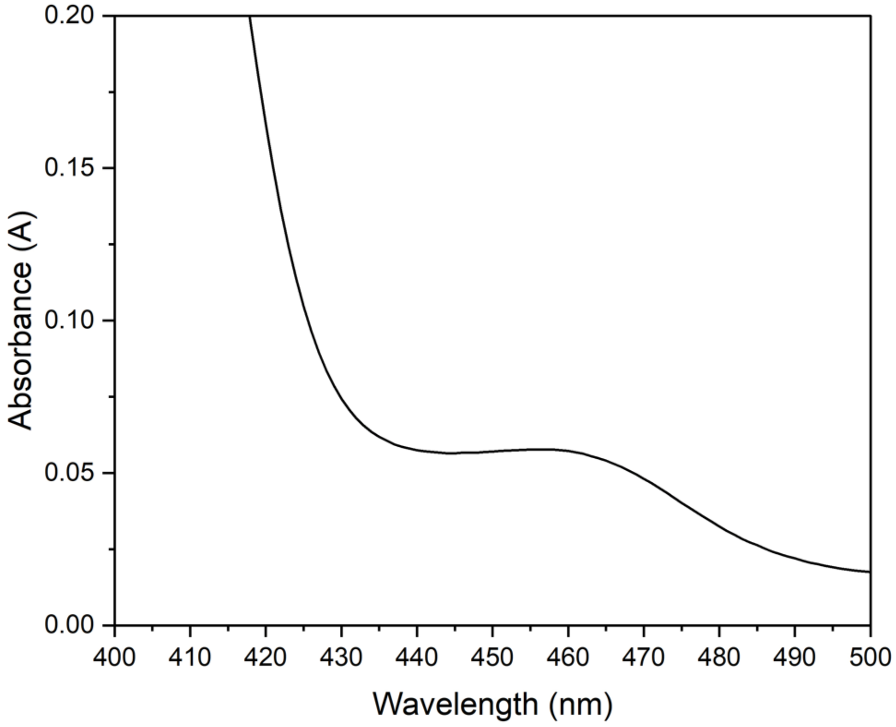

Supplementary Figure 101. Zoom-in of a UV-Vis-NIR spectrum of an aliquot taken from the residues remaining in bulb A in a catalytic acidification reaction using 600 equivalents of Rb/[Cy_3PH][I] in diethyl ether (Table 1, Entry 20). The characteristic peak at 458 nm indicates the presence of N_2H_4.

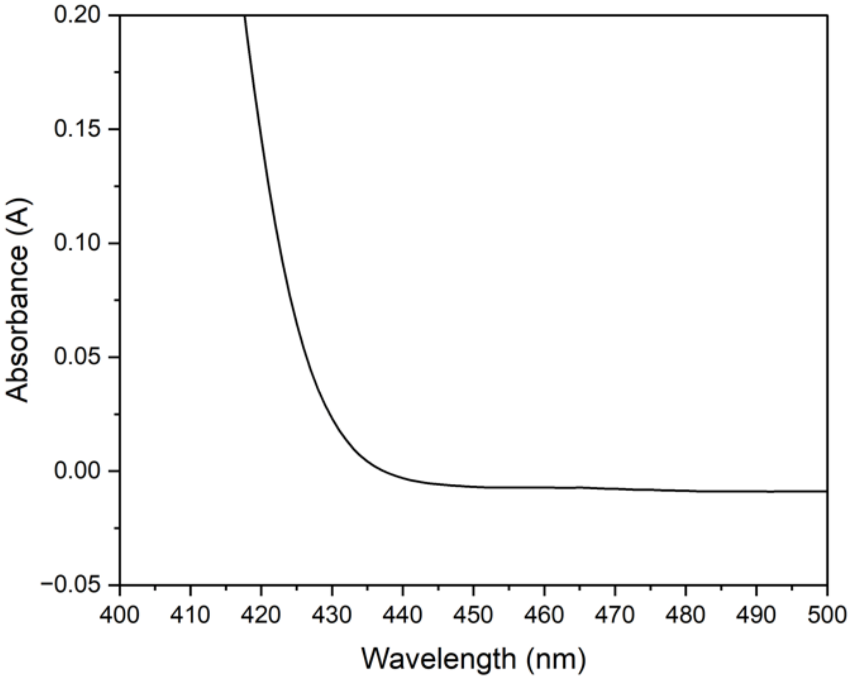

Supplementary Figure 102. Zoom-in of a UV-Vis-NIR spectrum of an aliquot taken from the HCl-trapped volatile fraction in a catalytic acidification reaction using 600 equivalents of RbC_8/[Cy_3PH][I] in diethyl ether (Table 1, Entry 21, $^{14}N_2$). The lack of the characteristic peak at 458 nm indicates that there is no N_2H_4 present.

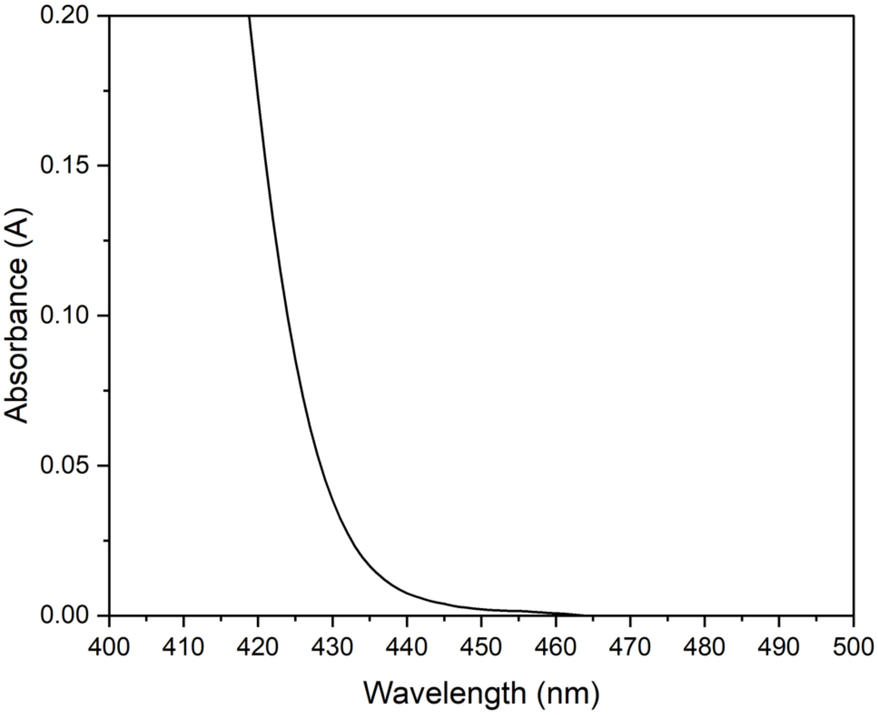

Supplementary Figure 103. Zoom-in of a UV-Vis-NIR spectrum of an aliquot taken from the residues remaining in bulb A in a catalytic acidification reaction using 600 equivalents of RbC_8/[Cy_3PH][I] in diethyl ether (Table 1, Entry 21, $^{14}N_2$). The very small characteristic peak at 458 nm indicates the presence of N_2H_4.

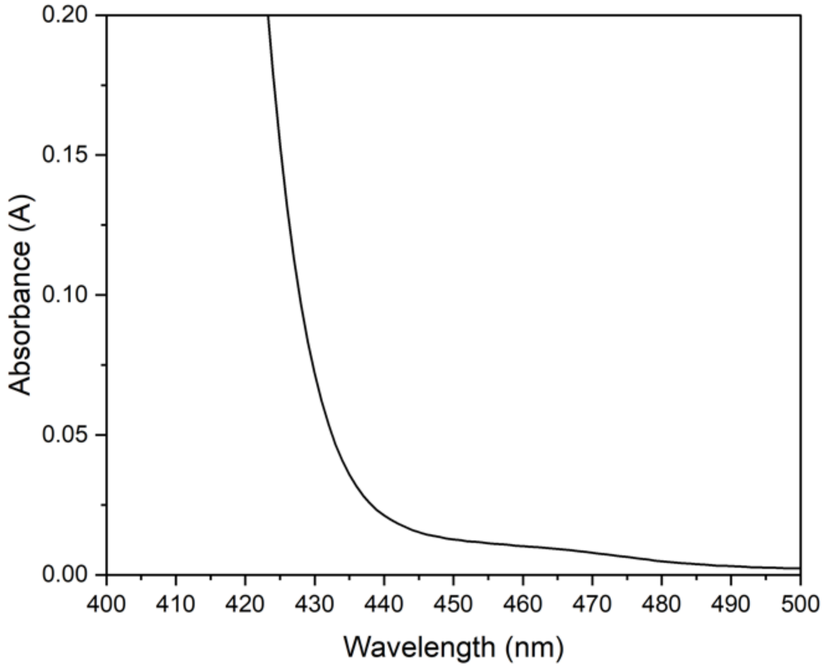

Supplementary Figure 104. Zoom-in of a UV-Vis-NIR spectrum of an aliquot taken from the HCl-trapped volatile fraction in a catalytic acidification reaction using 600 equivalents of RbC_8/[Cy_3PH][I] in diethyl ether (Table 1, Entry 22, $^{14}N_2$). The very small characteristic peak at 458 nm indicates the presence of N_2H_4.

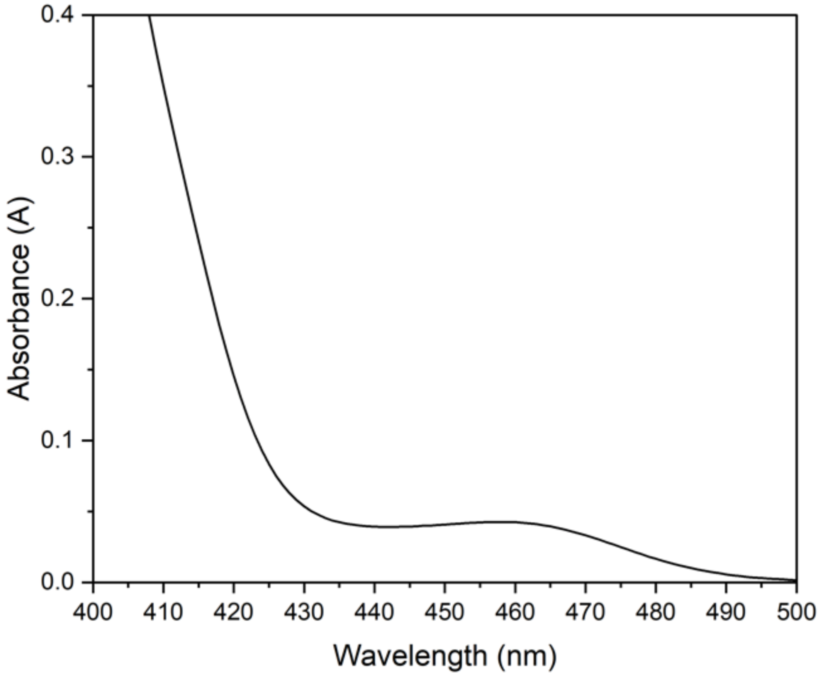

Supplementary Figure 105. Zoom-in of a UV-Vis-NIR spectrum of an aliquot taken from the residues remaining in bulb A in a catalytic acidification reaction using 600 equivalents of RbC_8/[Cy_3PH][I] in diethyl ether (Table 1, Entry 22, $^{14}N_2$). The characteristic peak at 458 nm indicates the presence of N_2H_4.

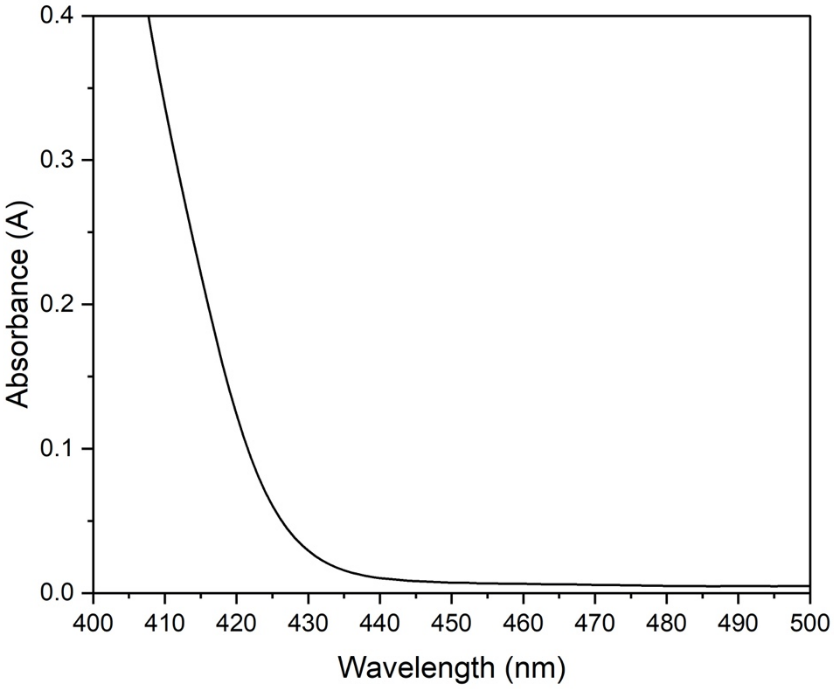

Supplementary Figure 106. Zoom-in of a UV-Vis-NIR spectrum of an aliquot taken from the HCl-trapped volatile fraction in a catalytic acidification reaction using 600 equivalents of RbC_8/[Cy_3PH][I] in diethyl ether under an $^{15}N_2$ atmosphere (Table 1, Entry 22, $^{15}N_2$). The lack of the characteristic peak at 458 nm indicates that there is no N_2H_4 present.

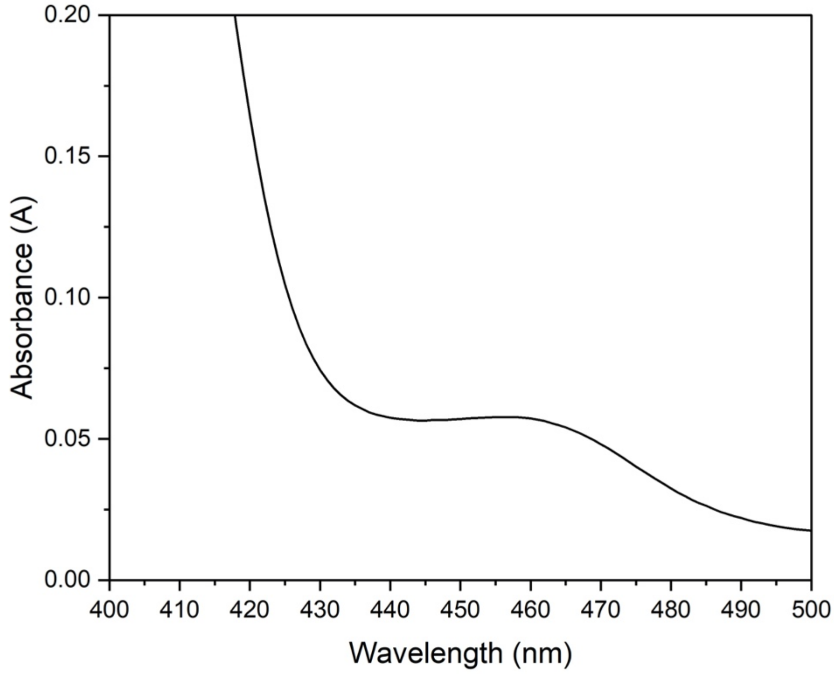

Supplementary Figure 107. Zoom-in of a UV-Vis-NIR spectrum of an aliquot taken from the residues remaining in bulb A in a catalytic acidification reaction using 600 equivalents of RbC_8/[Cy_3PH][I] in diethyl ether under an $^{15}N_2$ atmosphere (Table 1, Entry 22, $^{15}N_2$). The characteristic peak at 458 nm indicates the presence of N_2H_4.

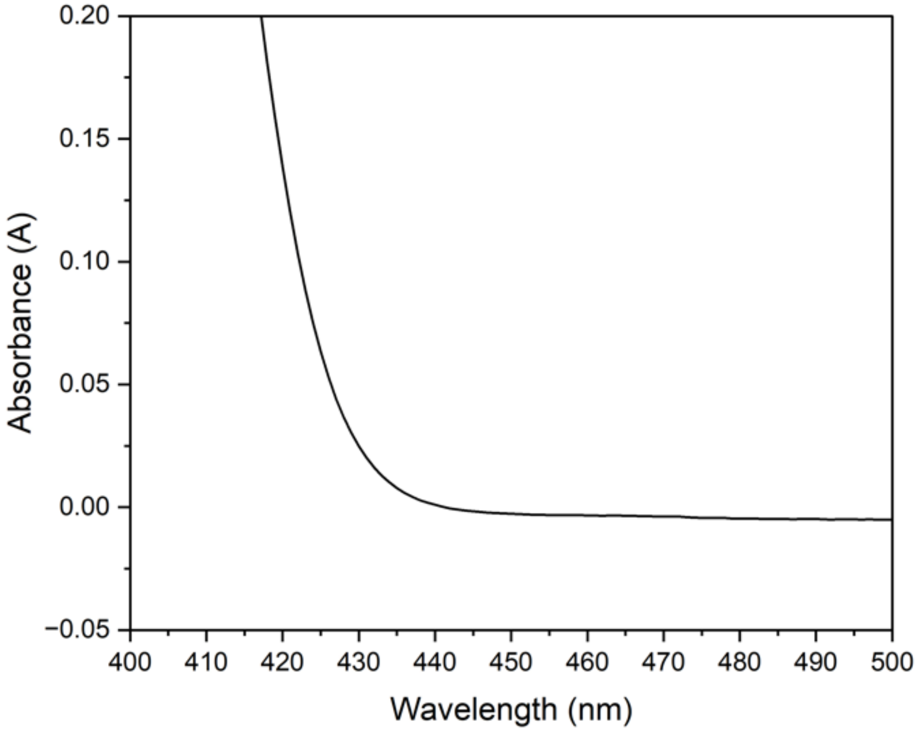

Supplementary Figure 108. Zoom-in of a UV-Vis-NIR spectrum of an aliquot taken from the HCl-trapped volatile fraction in a catalytic acidification reaction using 600 equivalents of Cs/[Cy_3PH][I] in diethyl ether (Table 1, Entry 23). The lack of the characteristic peak at 458 nm indicates that there is no N_2H_4 present.

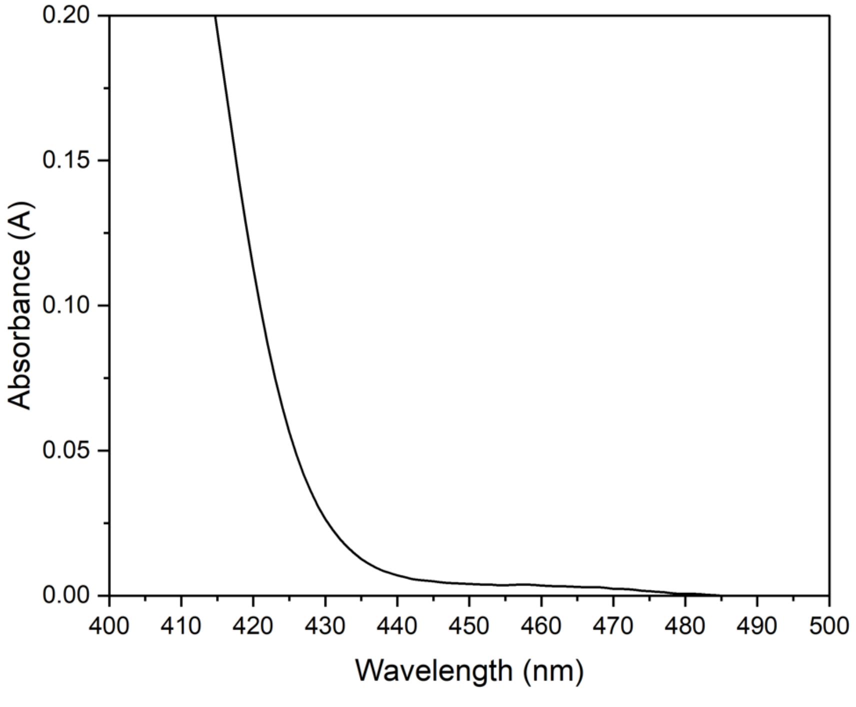

Supplementary Figure 109. Zoom-in of a UV-Vis-NIR spectrum of an aliquot taken from the residues remaining in bulb A in a catalytic acidification reaction using 600 equivalents of Cs/[Cy_3PH][I] in diethyl ether (Table 1, Entry 23). The very small characteristic peak at 458 nm indicates the presence of N_2H_4.

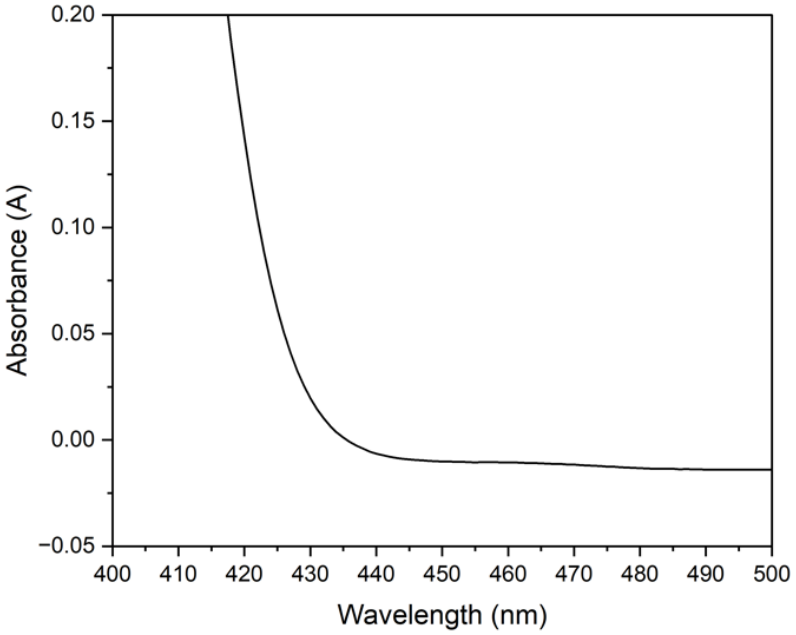

Supplementary Figure 110. Zoom-in of a UV-Vis-NIR spectrum of an aliquot taken from the HCl-trapped volatile fraction in a catalytic acidification reaction using 600 equivalents of CsC_8/[Cy_3PH][I] in diethyl ether (Table 1, Entry 24). The lack of the characteristic peak at 458 nm indicates that there is no N_2H_4 present.

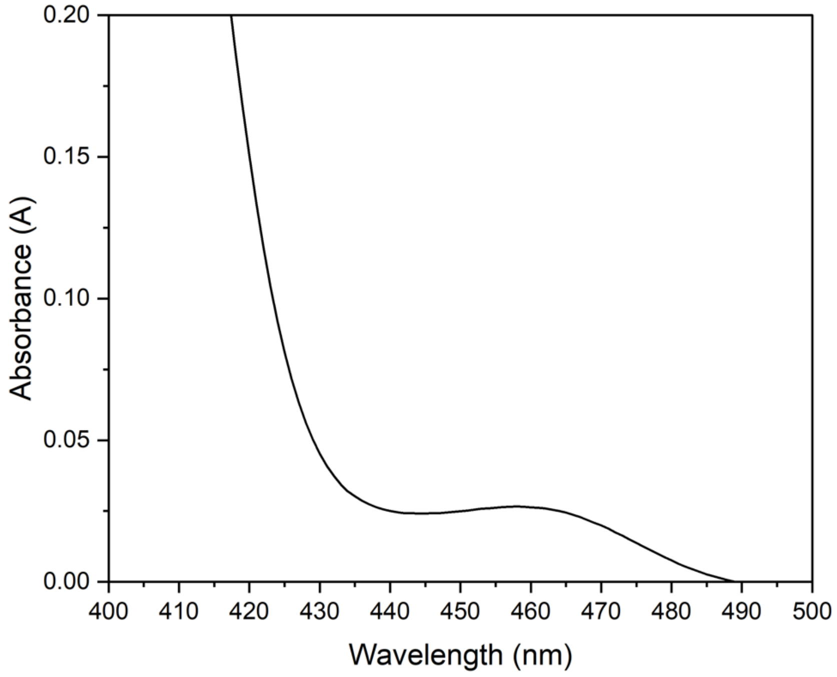

Supplementary Figure 111. Zoom-in of a UV-Vis-NIR spectrum of an aliquot taken from the residues remaining in bulb A in a catalytic acidification reaction using 600 equivalents of CsC_8/[Cy_3PH][I] in diethyl ether (Table 1, Entry 24). The characteristic peak at 458 nm indicates the presence of N_2H_4.

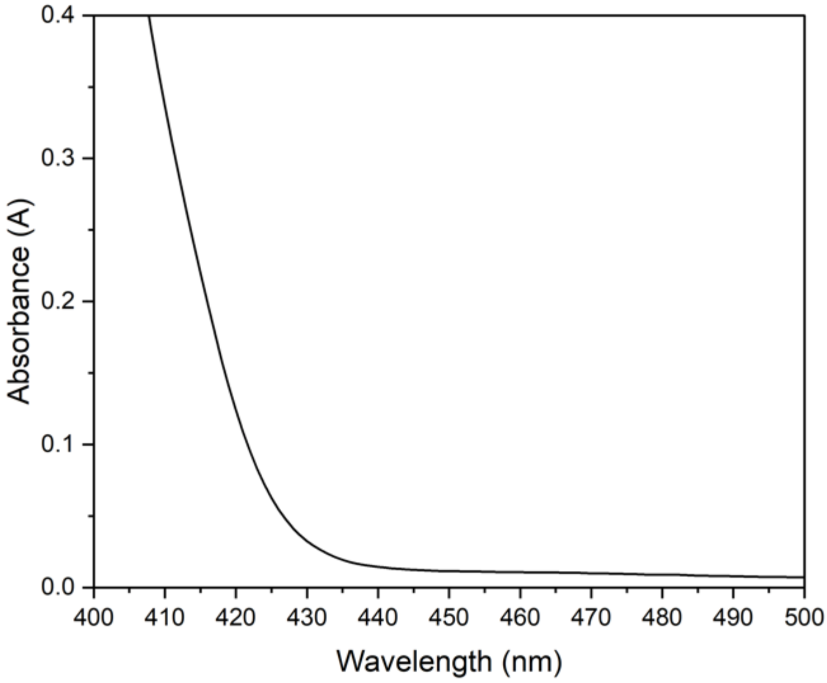

Supplementary Figure 112. Zoom-in of a UV-Vis-NIR spectrum of an aliquot taken from the HCl-trapped volatile fraction in a catalyst-free acidification reaction using 600 equivalents of KC_8/[Cy_3PH][I] along with 1 equivalent of $NaNO_3$ in diethyl ether (Supplementary Table 3, Entry 26). The lack of the characteristic peak at 458 nm indicates that there is no N_2H_4 present.

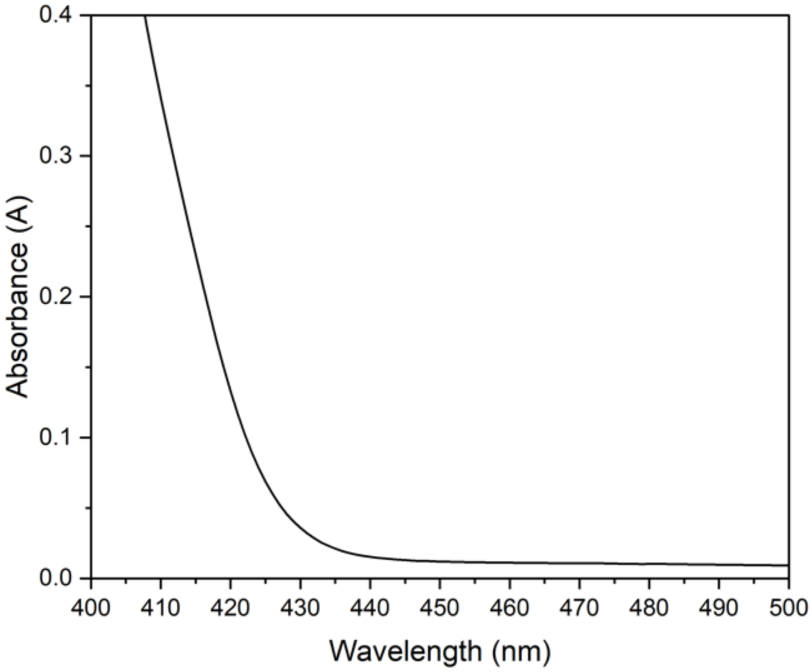

Supplementary Figure 113. Zoom-in of a UV-Vis-NIR spectrum of an aliquot taken from the residues remaining in bulb A in a catalyst-free acidification reaction using 600 equivalents of KC_8/[Cy_3PH][I] along with 1 equivalent of $NaNO_3$ in diethyl ether (Supplementary Table 3, Entry 26). The lack of the characteristic peak at 458 nm indicates that there is no N_2H_4 present.

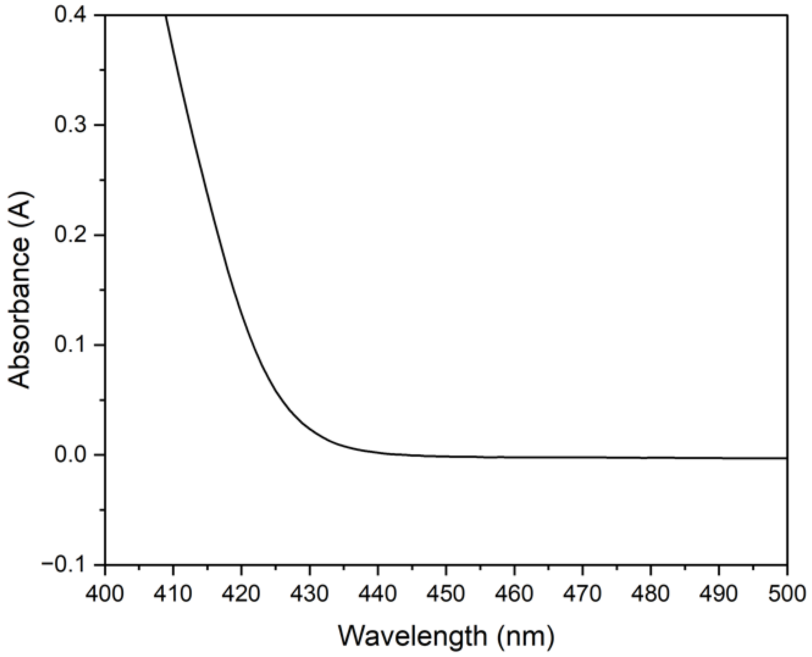

Supplementary Figure 114. Zoom-in of a UV-Vis-NIR spectrum of an aliquot taken from the HCl-trapped volatile fraction in a catalytic acidification reaction using 600 equivalents of KC_8/[Cy_3PH][I] along with 1 equivalent of $NaNO_3$ in diethyl ether (Supplementary Table 3, Entry 27). The lack of the characteristic peak at 458 nm indicates that there is no N_2H_4 present.

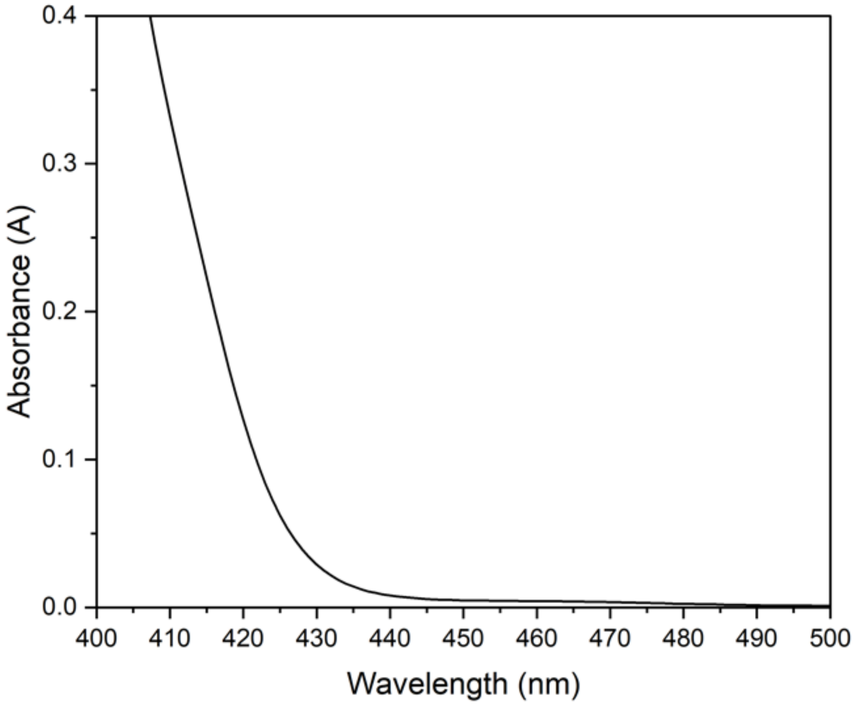

Supplementary Figure 115. Zoom-in of a UV-Vis-NIR spectrum of an aliquot taken from the residues remaining in bulb A in a catalytic acidification reaction using 600 equivalents of KC_8/[Cy_3PH][I] along with 1 equivalent of $NaNO_3$ in diethyl ether (Supplementary Table 3, Entry 27). The lack of the characteristic peak at 458 nm indicates that there is no N_2H_4 present.

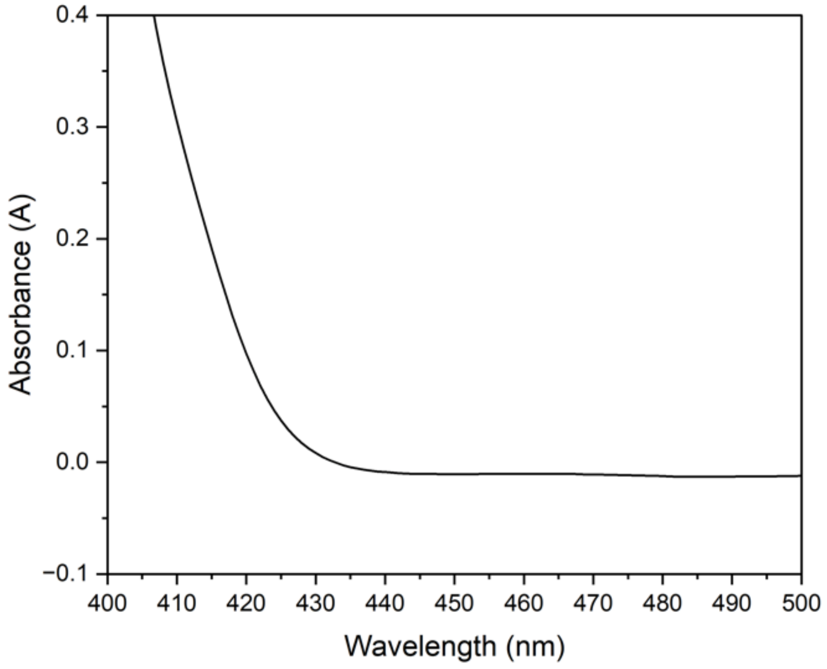

Supplementary Figure 116. Zoom-in of a UV-Vis-NIR spectrum of an aliquot taken from the HCl-trapped volatile fraction in a catalyst-free acidification reaction using 600 equivalents of KC_8/[Cy_3PH][I] along with 1 equivalent of $NaNO_2$ in diethyl ether (Supplementary Table 3, Entry 28). The lack of the characteristic peak at 458 nm indicates that there is no N_2H_4 present.

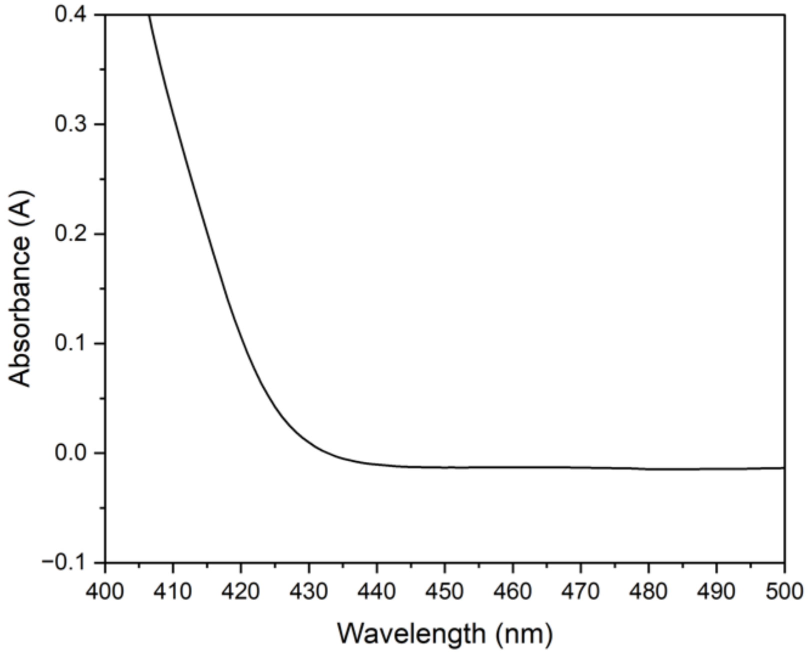

Supplementary Figure 117. Zoom-in of a UV-Vis-NIR spectrum of an aliquot taken from the residues remaining in bulb A in a catalyst-free acidification reaction using 600 equivalents of KC_8/[Cy_3PH][I] along with 1 equivalent of $NaNO_2$ in diethyl ether (Supplementary Table 3, Entry 28). The lack of the characteristic peak at 458 nm indicates that there is no N_2H_4 present.

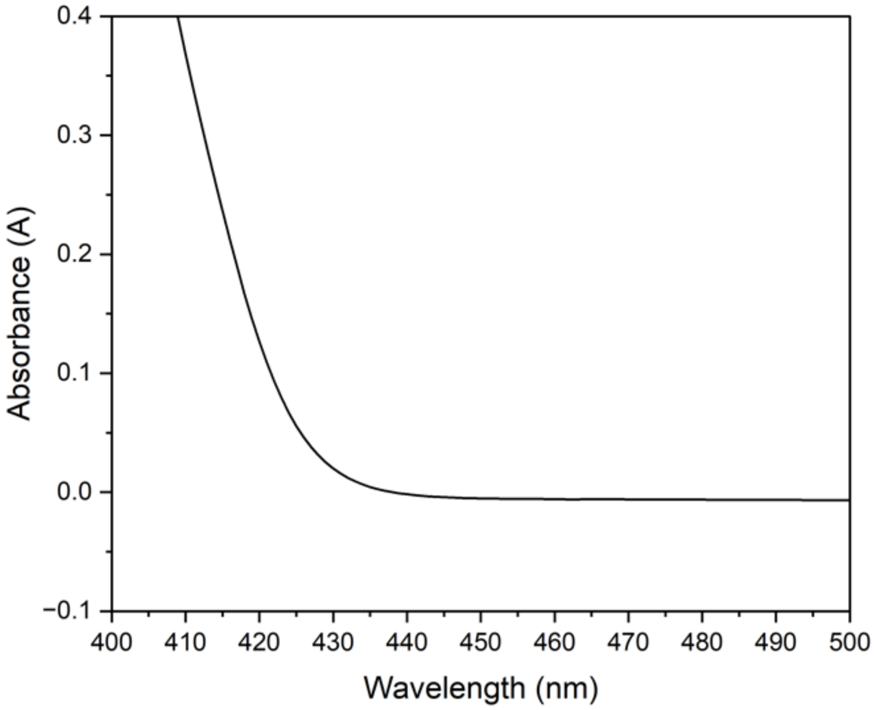

Supplementary Figure 118. Zoom-in of a UV-Vis-NIR spectrum of an aliquot taken from the HCl-trapped volatile fraction in a catalytic acidification reaction using 600 equivalents of KC_8/[Cy_3PH][I] along with 1 equivalent of $NaNO_2$ in diethyl ether (Supplementary Table 3, Entry 29). The lack of the characteristic peak at 458 nm indicates that there is no N_2H_4 present.

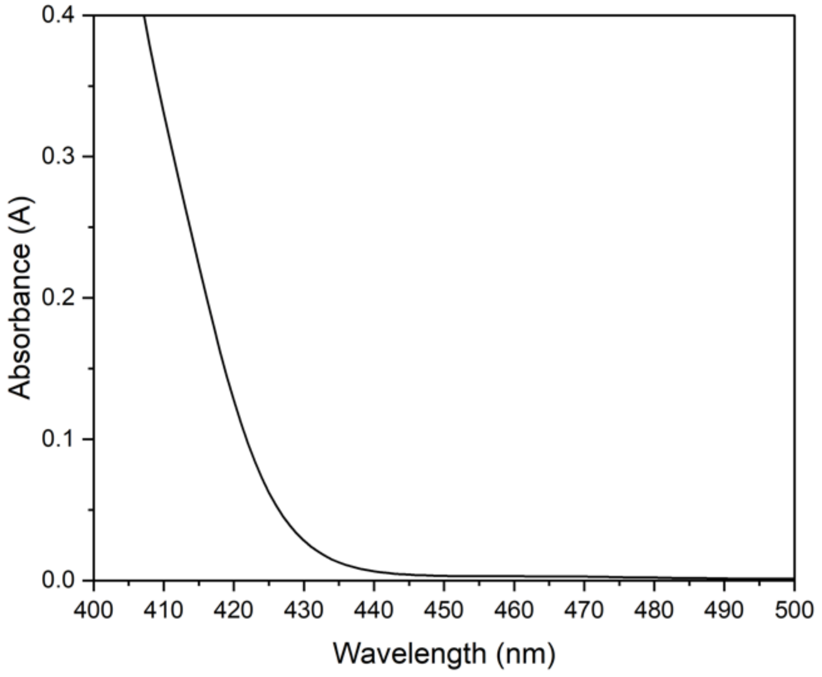

Supplementary Figure 119. Zoom-in of a UV-Vis-NIR spectrum of an aliquot taken from the residues remaining in bulb A in a catalytic acidification reaction using 600 equivalents of KC_8/[Cy_3PH][I] along with 1 equivalent of $NaNO_2$ in diethyl ether (Supplementary Table 3, Entry 29). The lack of the characteristic peak at 458 nm indicates that there is no N_2H_4 present.

IR Spectra

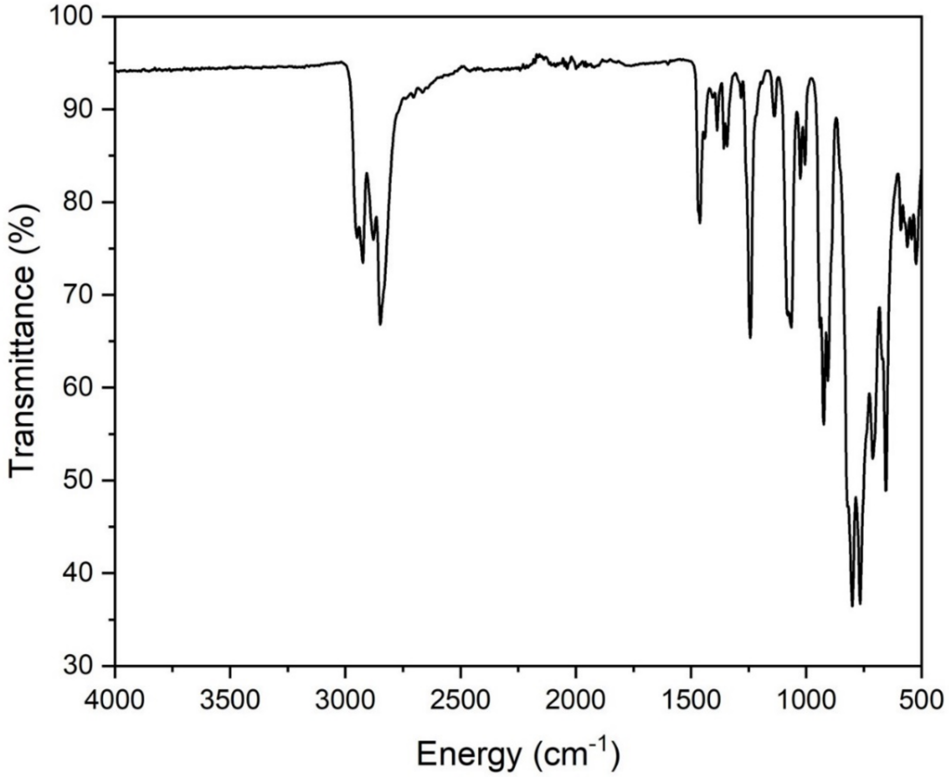

Supplementary Figure 120. ATR-IR spectrum of **1**.

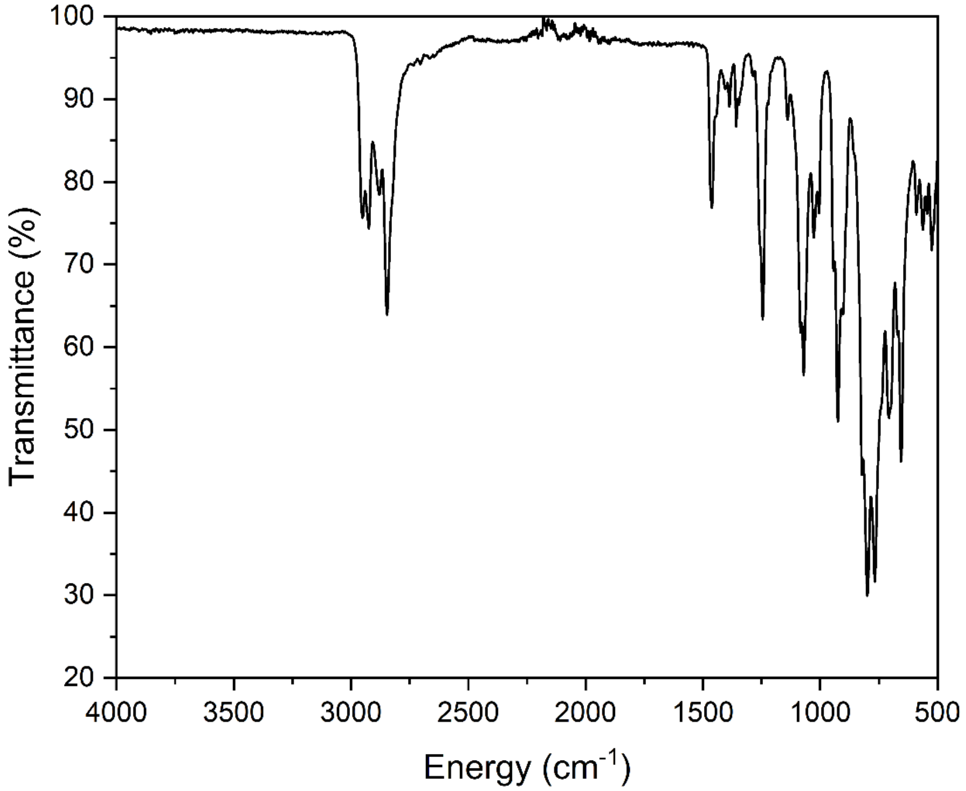

Supplementary Figure 121. ATR-IR spectrum of **1-$^{15}N_2$.**

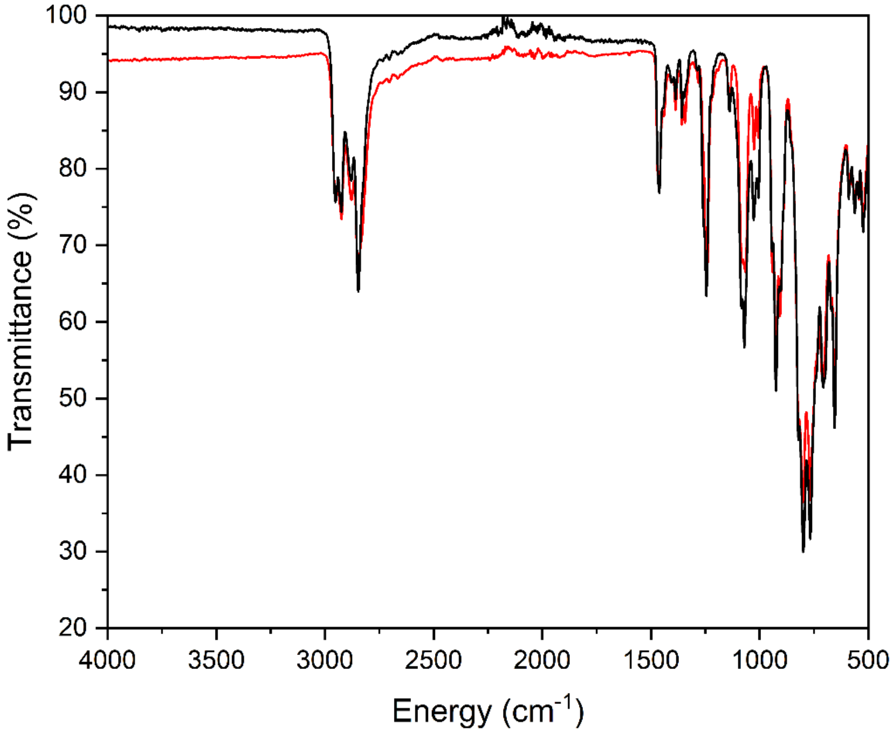

Supplementary Figure 122. Comparative ATR-IR spectra of **1** and **1-$^{15}N_2$**. They are super-imposable.

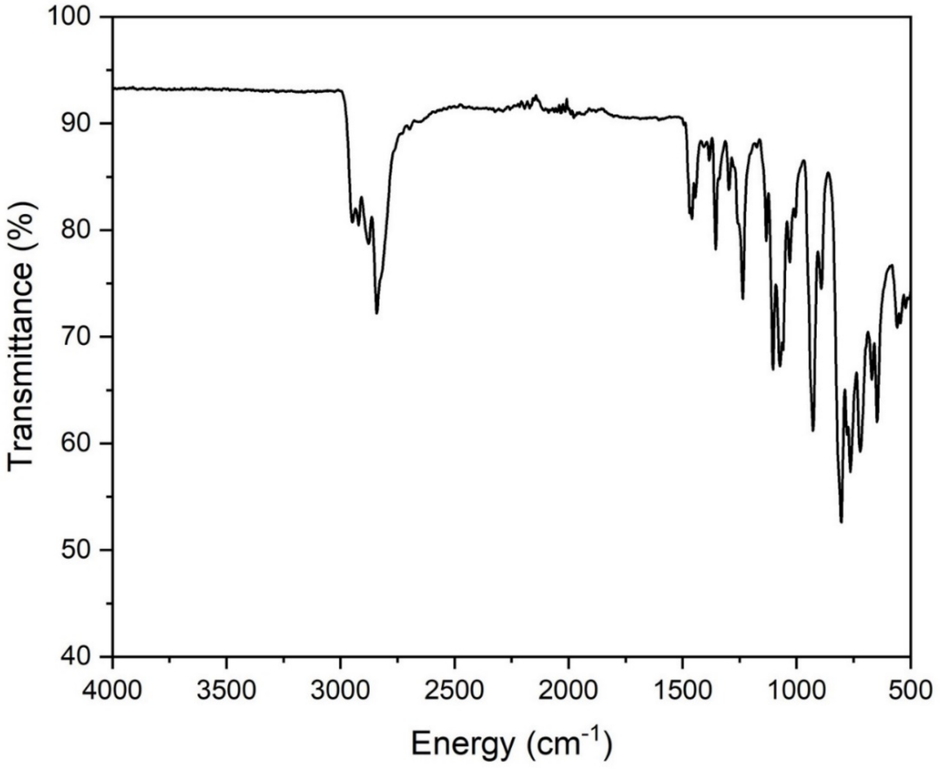

Supplementary Figure 123. ATR-IR spectrum of **2-crypt**.

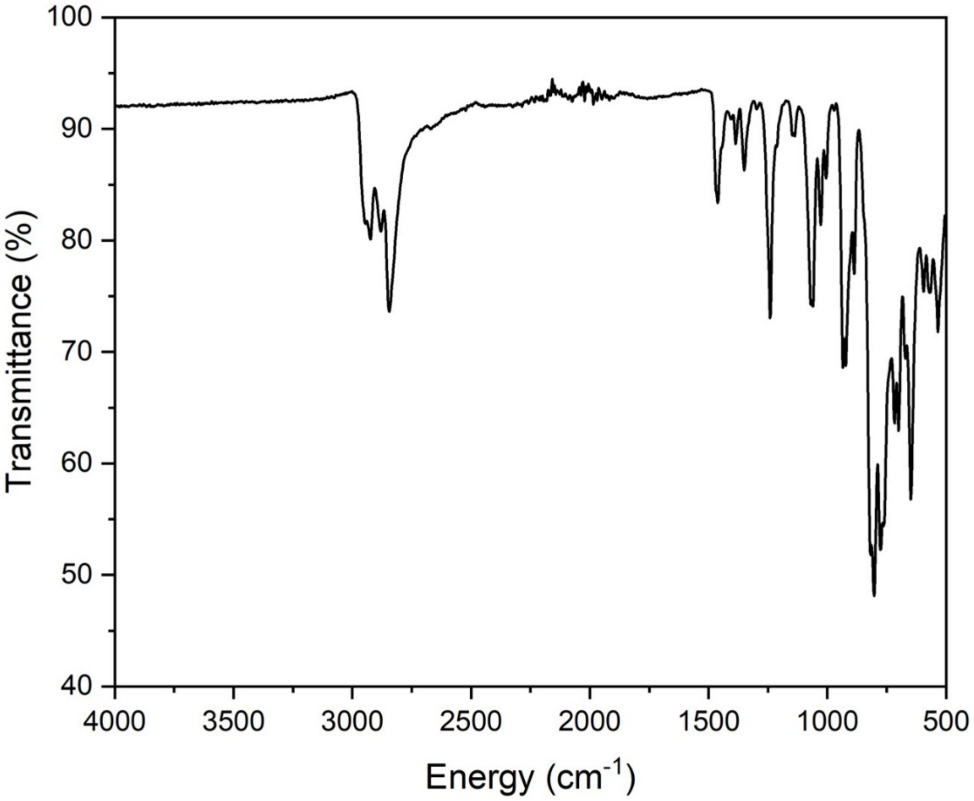

Supplementary Figure 124. ATR-IR spectrum of **2-K**.

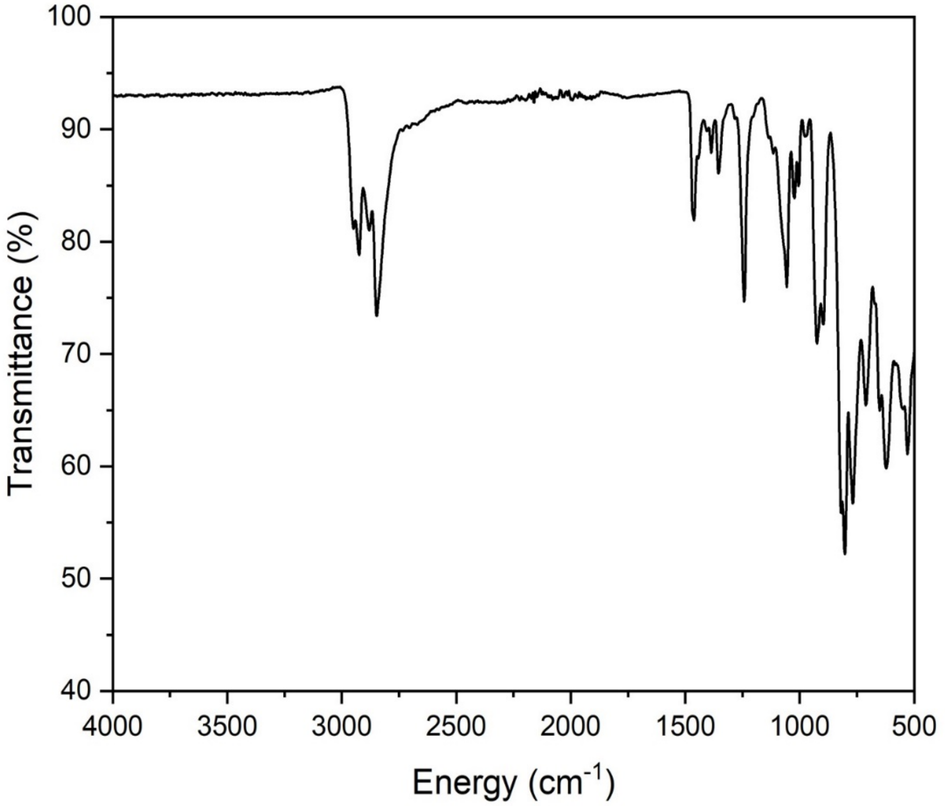

Supplementary Figure 125. ATR-IR spectrum of **3**.

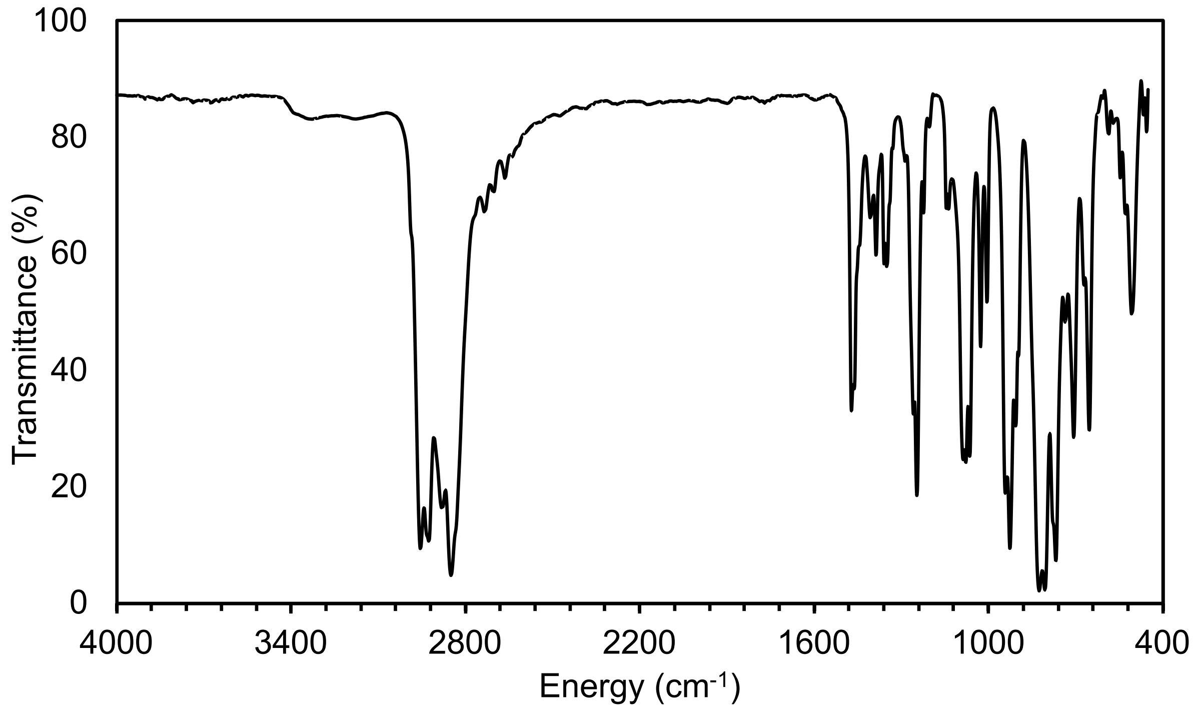

Supplementary Figure 126. IR spectrum of **4,** recorded in a KBr pellet.

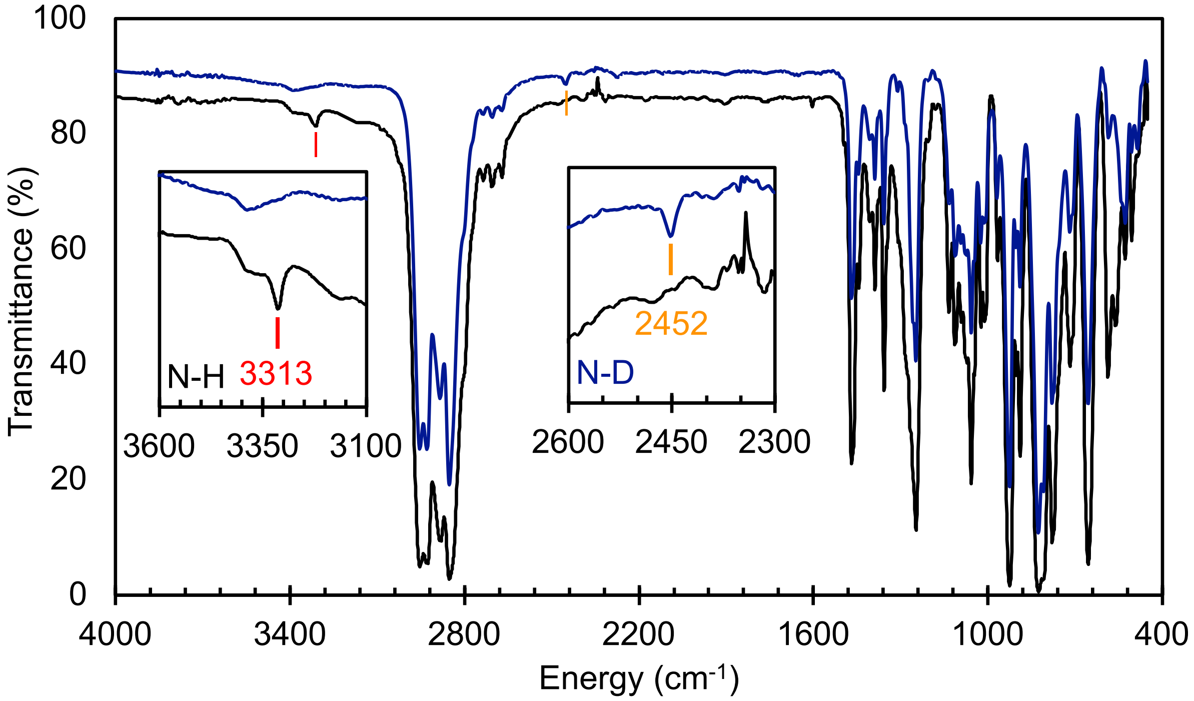

Supplementary Figure 127. Comparative IR spectrum of **5** and **5-D₂**, recorded in a KBr pellet. N-H and N-D stretches at 3313 cm^{-1} and 2452 cm^{-1} are explicitly labelled.

Raman spectra

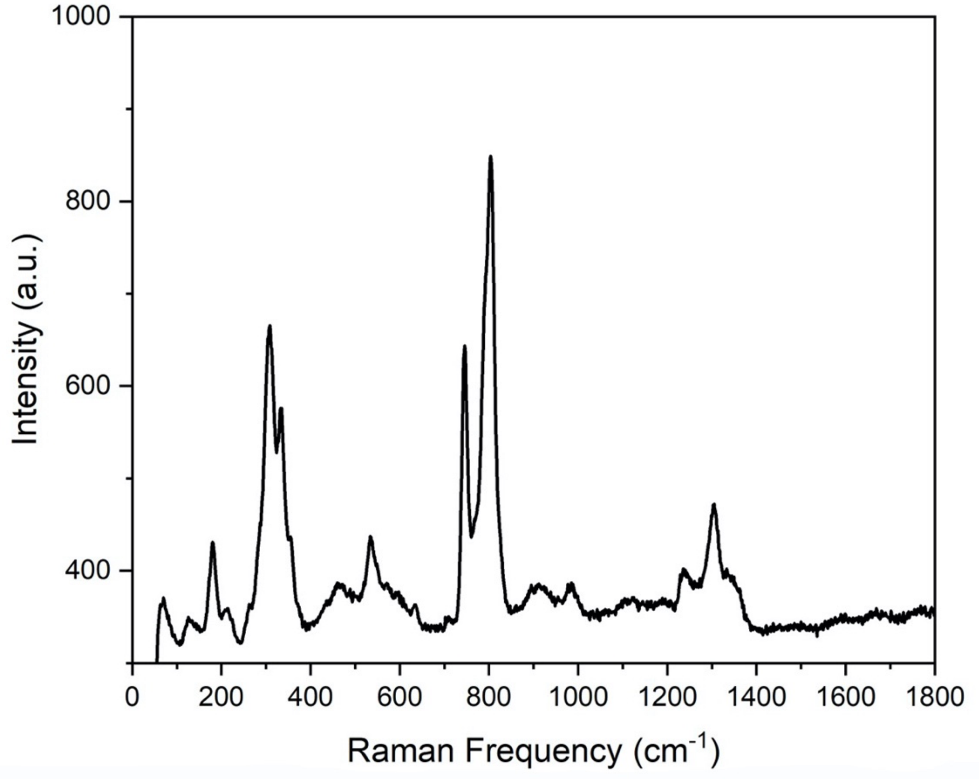

Supplementary Figure 128. Raman spectrum of **1** using a 638 nm over the range 10-1800 cm^{-1}. There were no other distinguishable features in the spectrum past this frequency.

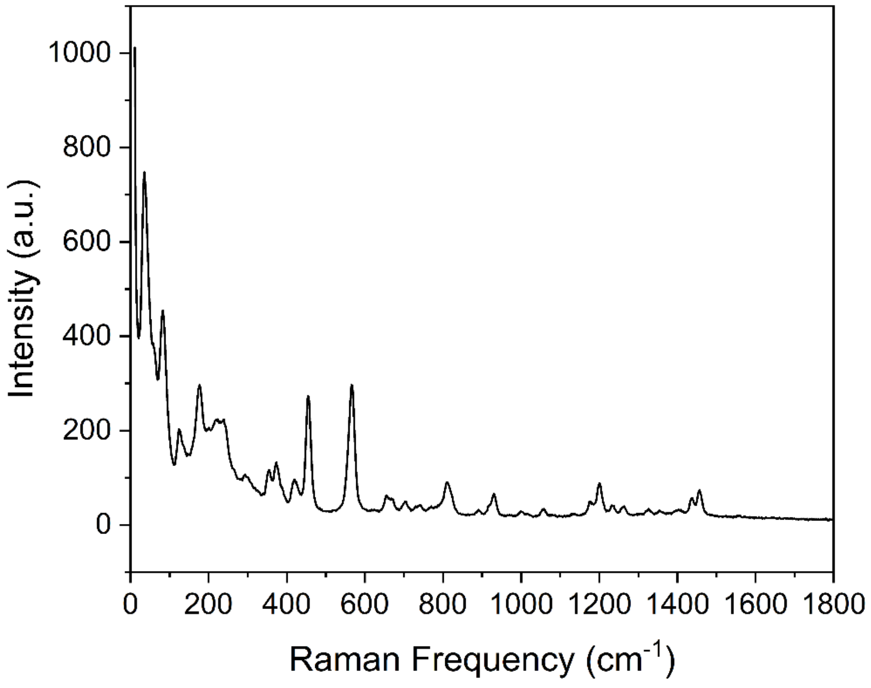

Supplementary Figure 129. Raman spectrum of **1-$^{15}N_2$** using a 638 nm over the range 10-1800 cm^{-1}. There were no other distinguishable features in the spectrum past this frequency.

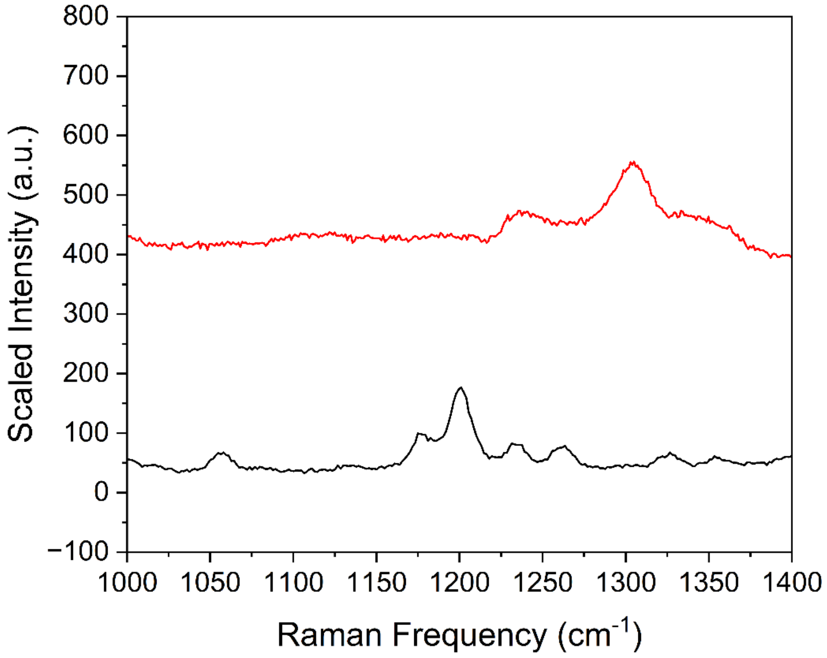

Supplementary Figure 130. Comparative Raman spectra of **1** (red line) and **1-$^{15}N_2$** (black line) using a 638 nm over the range 10-1800 cm^{-1}. Zoomed-in between 1000 and 1400 cm^{-1} to highlight N=N stretching frequency bands. It shifts from the range ~1225-1375 cm^{-1} to ~1125-1275 cm^{-1} for **1-$^{15}N_2$**, which is consistent with reduced mass isotopologue considerations.

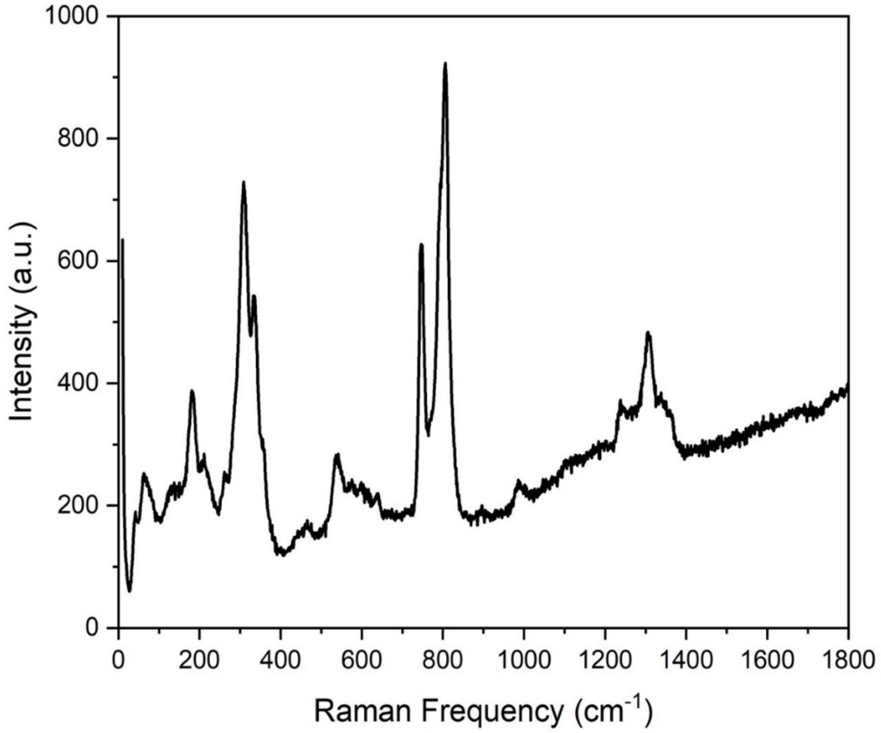

Supplementary Figure 131. Raman spectrum of **2-crypt** using a 638 nm over the range 10-1800 cm^{-1}. There were no other distinguishable features in the spectrum past this frequency.

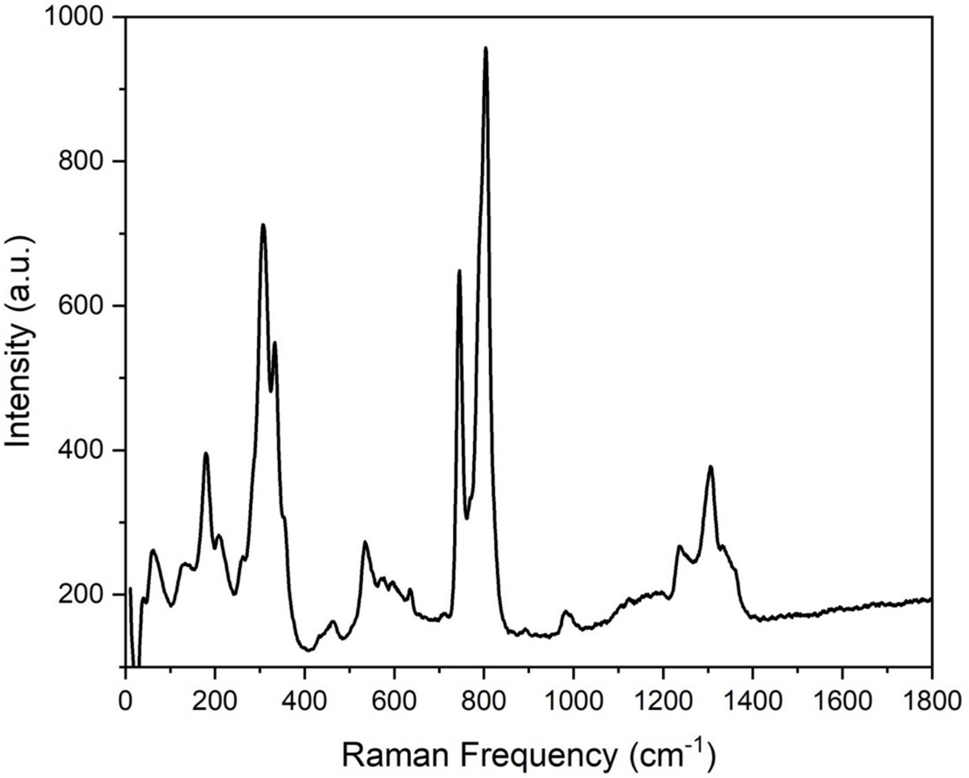

Supplementary Figure 132. Raman spectrum of **2-K** using a 638 nm over the range 10-1800 cm^{-1}. There were no other distinguishable features in the spectrum past this frequency.

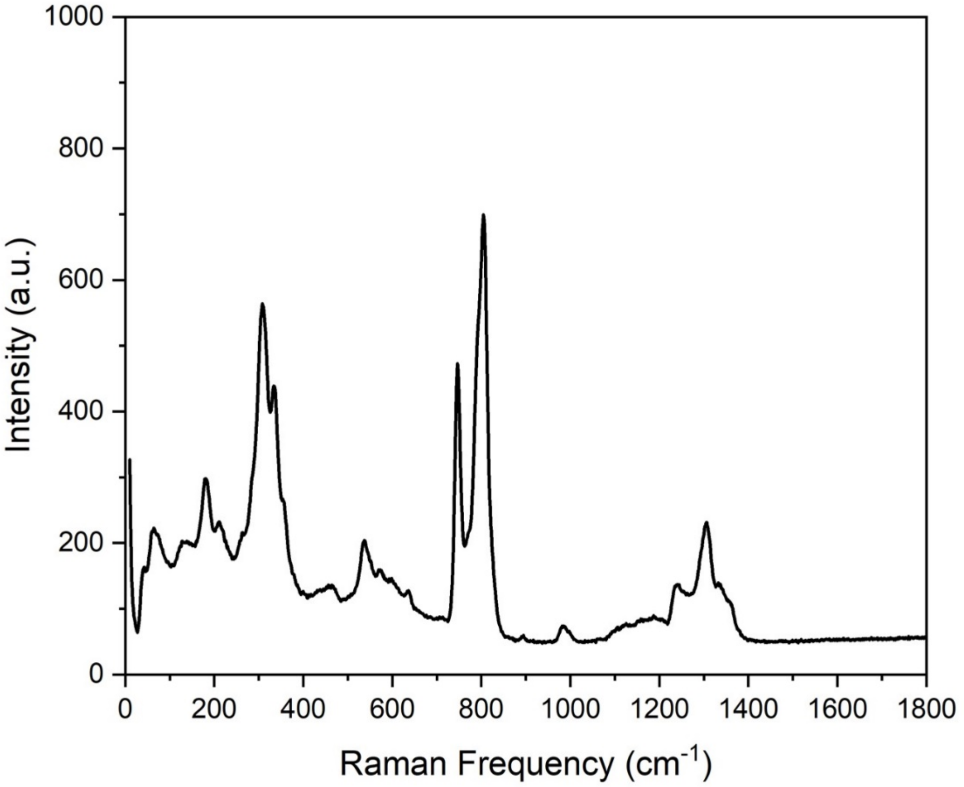

Supplementary Figure 133. Raman spectrum of **3** using a 638 nm over the range 10-1800 cm^{-1}. There were no other distinguishable features in the spectrum past this frequency.

EPR spectra

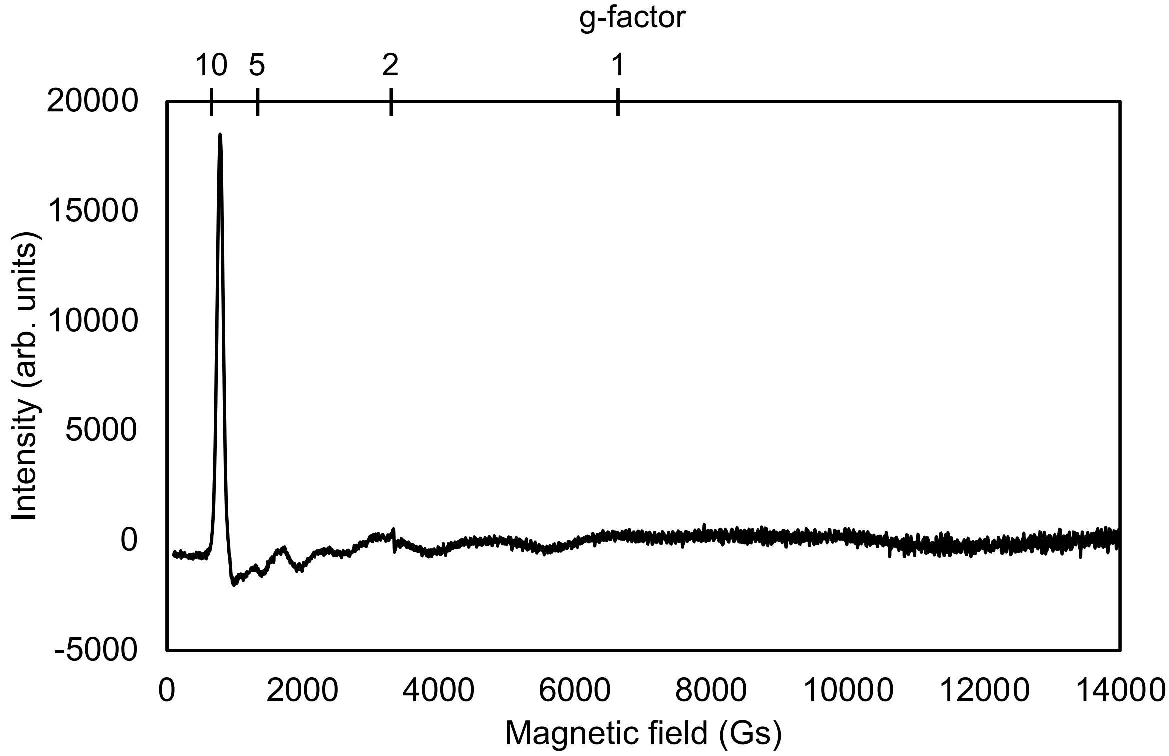

Supplementary Figure 134. Solid-state EPR spectrum of **2-crypt**, recorded at 6 K.

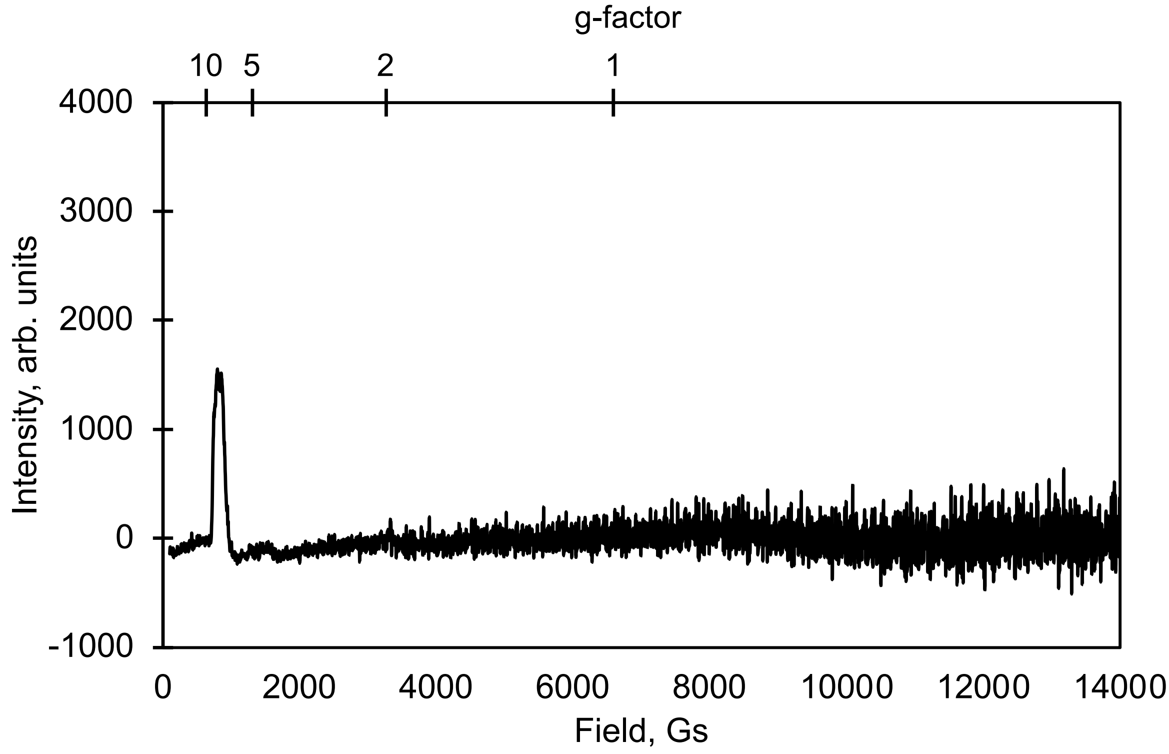

Supplementary Figure 135. Solution-state EPR spectrum of **2-crypt**, recorded at 6 K in 2-MeTHF glass at 10 mM sample concentration.

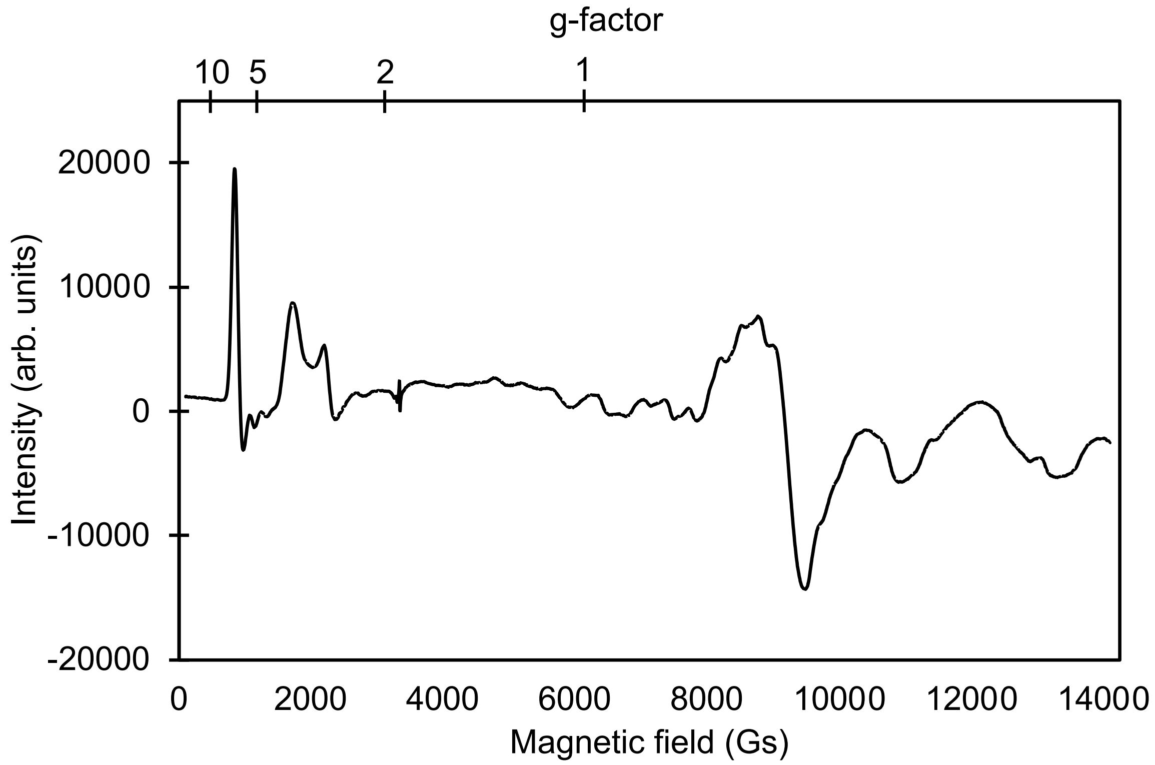

Supplementary Figure 136. Solid-state EPR spectrum of **2-K**, recorded at 6 K.

SQUID magnetometry

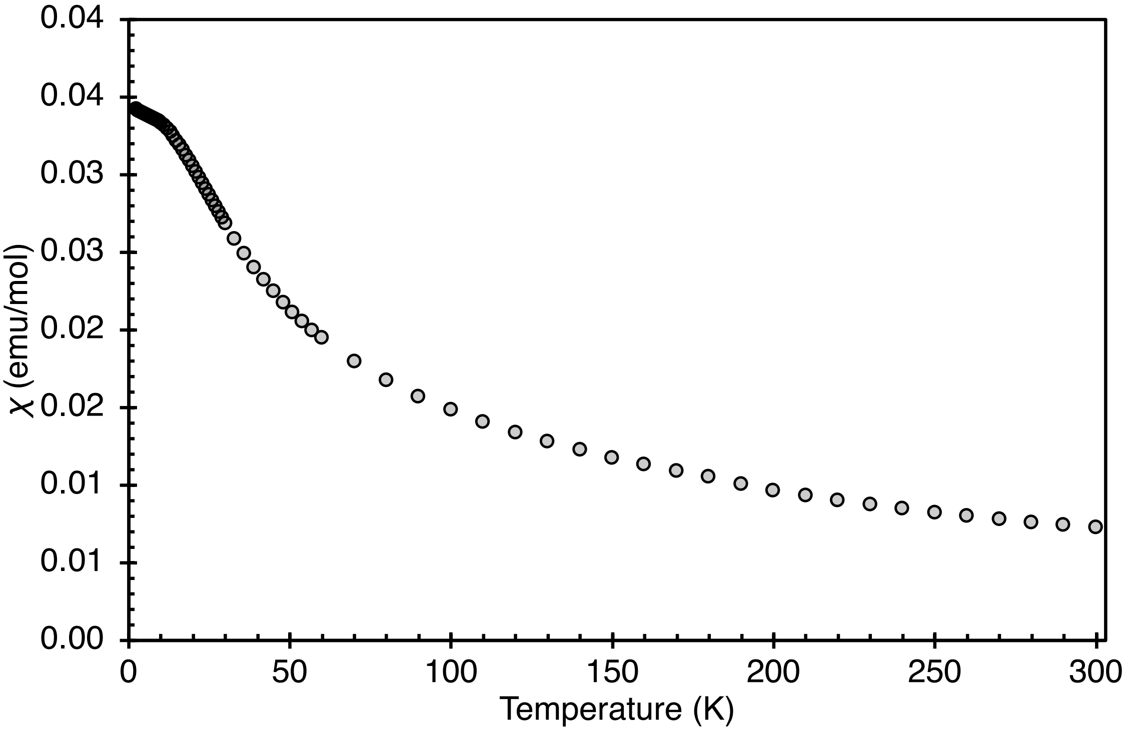

Supplementary Figure 137. Molar magnetic susceptibility *versus* temperature plot for **1** over the temperature range 2-300 K, measured under applied field of 0.1 T.

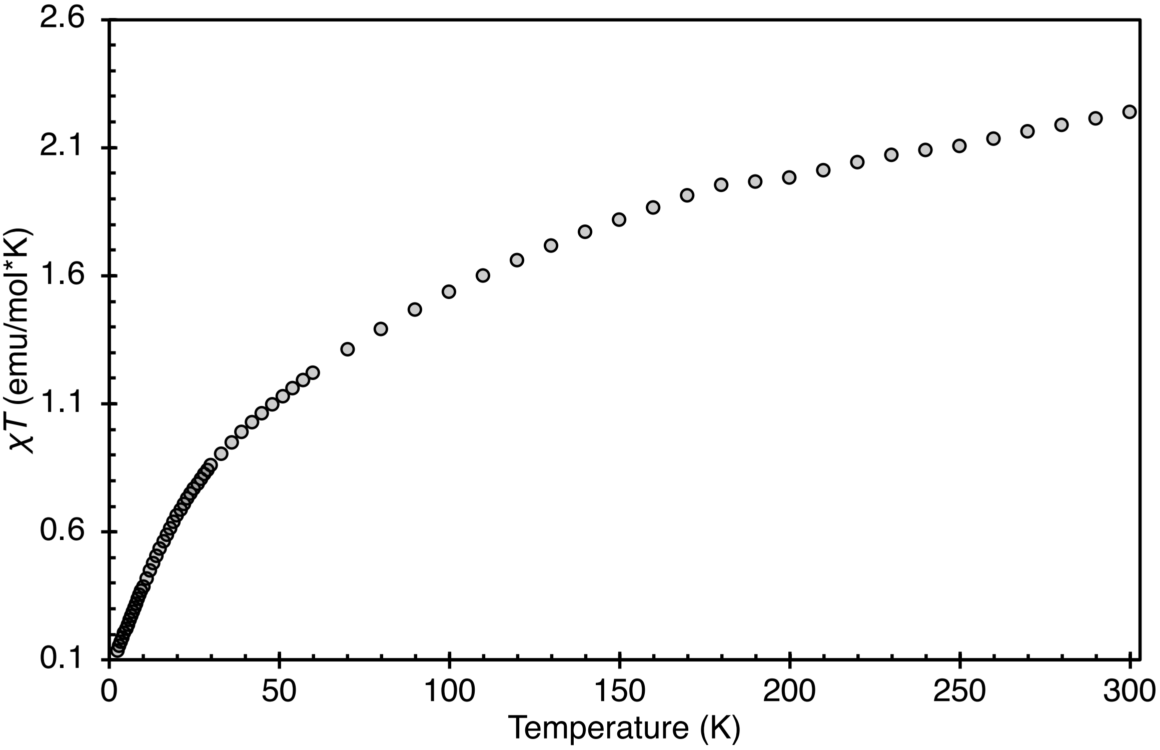

Supplementary Figure 138. Temperature-dependent magnetisation data plot for **1** over the temperature range 2-300 K, measured under applied field of 0.1 T.

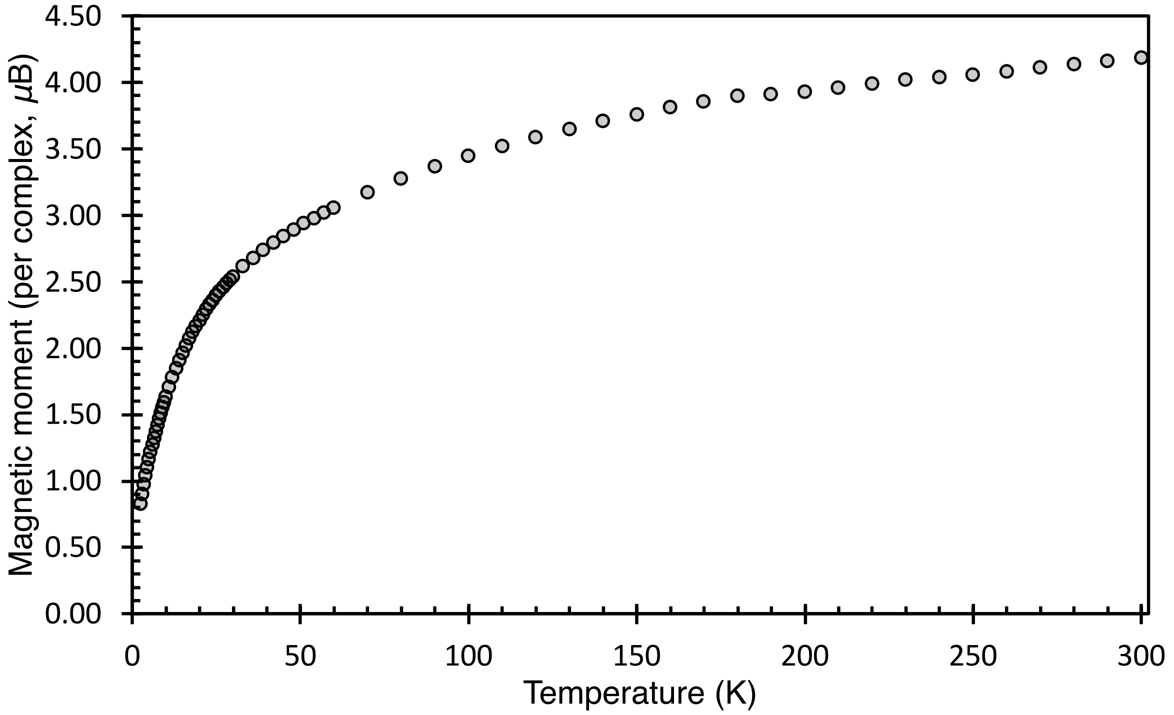

Supplementary Figure 139. Magnetic moment per complex *versus* temperature for **1** over the temperature range 2-300 K, measured under applied field of 0.1 T

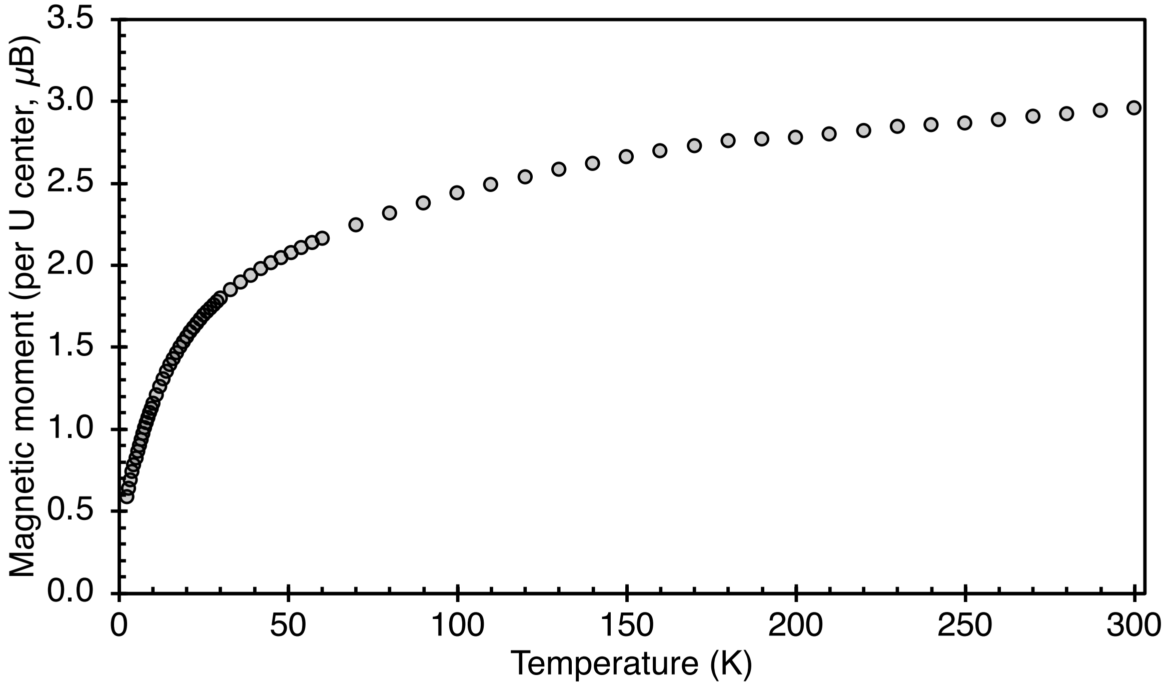

Supplementary Figure 140. Magnetic moment per uranium centre *versus* temperature for **1** over the temperature range 2-300 K, measured under applied field of 0.1 T

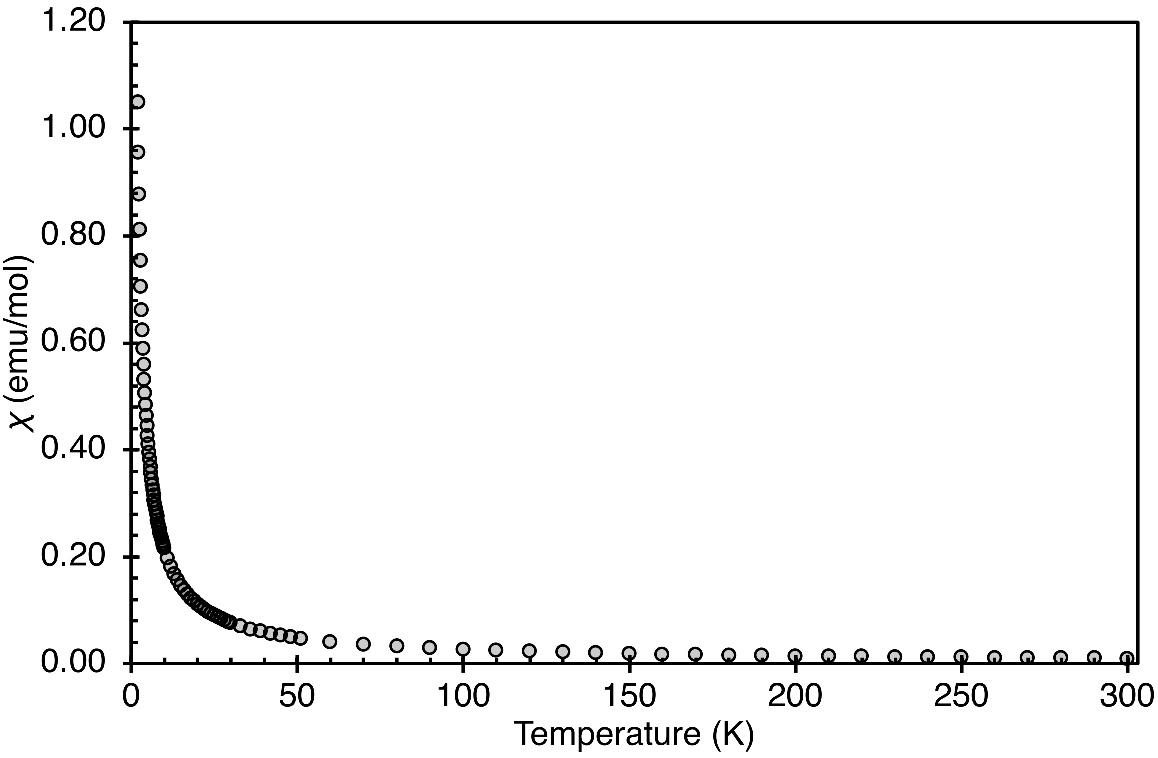

Supplementary Figure 141. Molar magnetic susceptibility *versus* temperature plot for **2-crypt** over the temperature range 2-300 K, measured under applied field of 0.1 T.

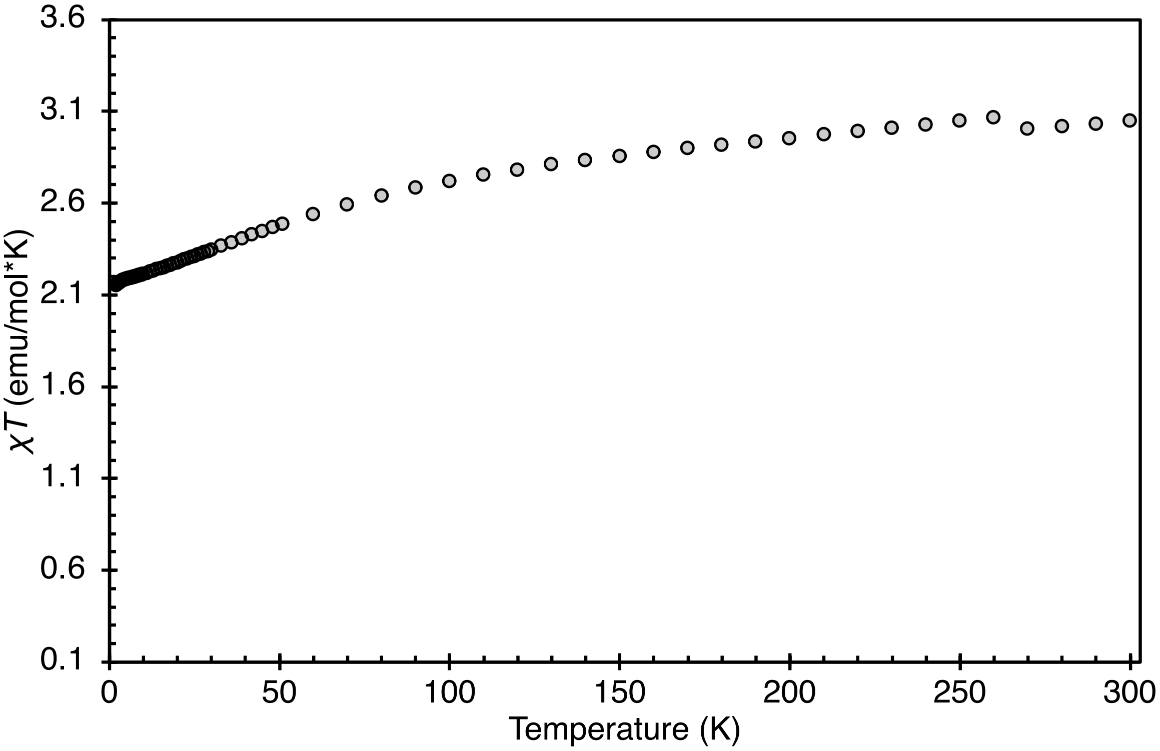

Supplementary Figure 142. Temperature-dependent magnetisation data plot for **2-crypt** over the temperature range 2-300 K, measured under applied field of 0.1 T.

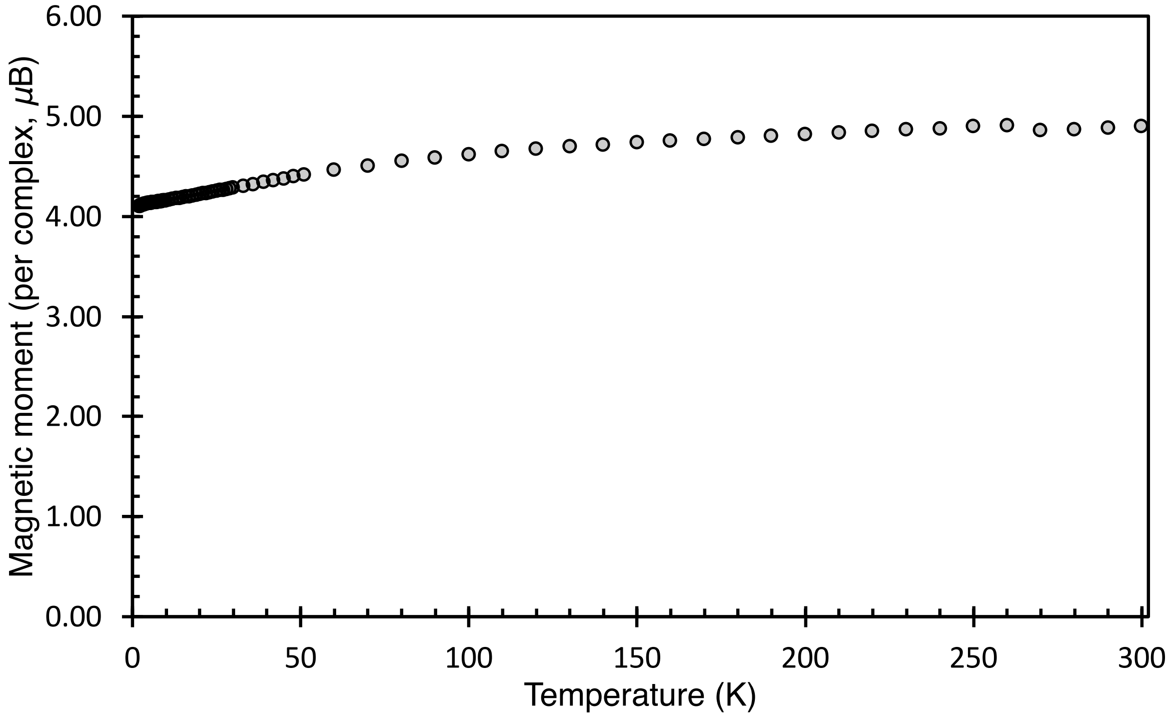

Supplementary Figure 143. Magnetic moment per complex *versus* temperature for **2-crypt** over the temperature range 2-300 K, measured under applied field of 0.1 T.

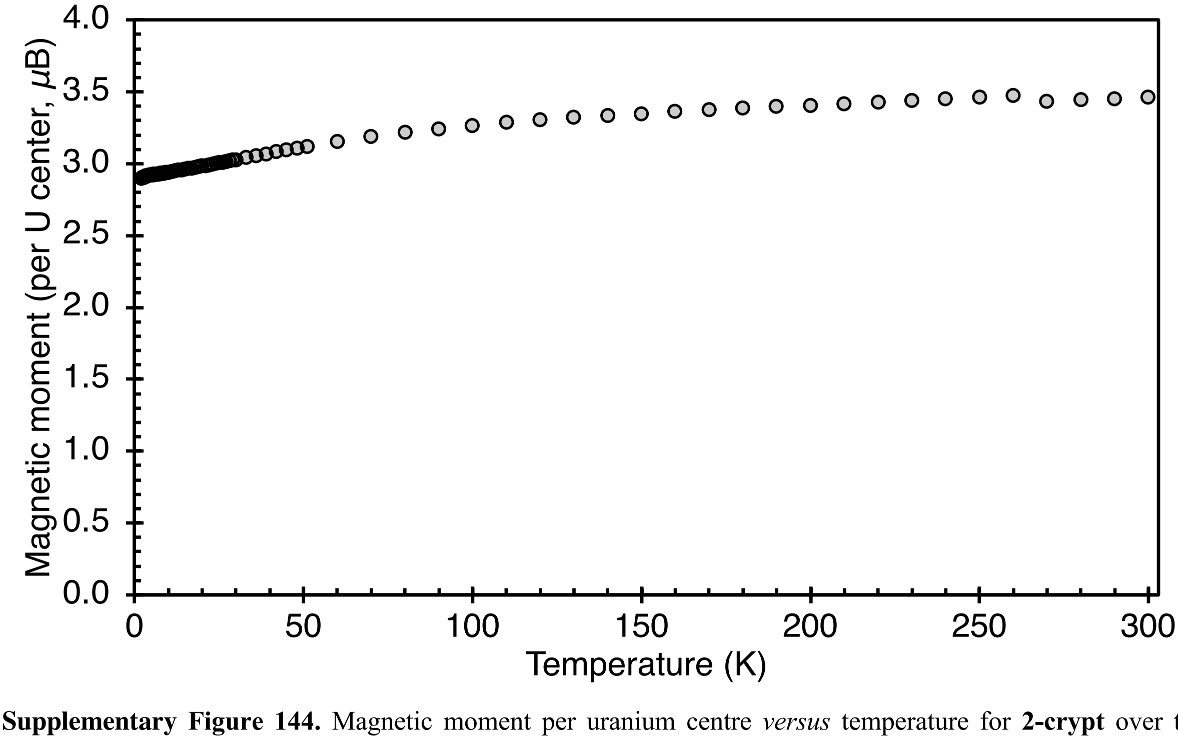

Supplementary Figure 144. Magnetic moment per uranium centre *versus* temperature for **2-crypt** over the temperature range 2-300 K, measured under applied field of 0.1 T

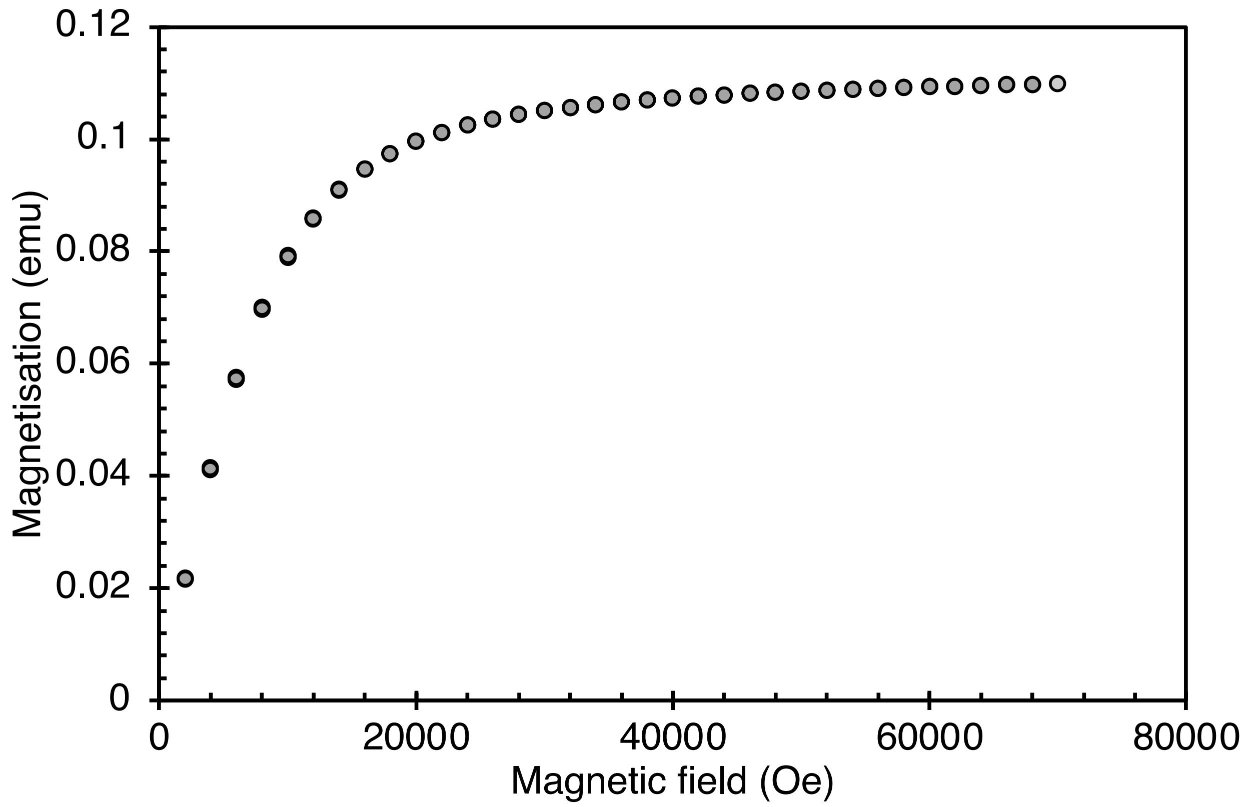

Supplementary Figure 145. Magnetisation *versus* field (M *vs* H) curve for **2-crypt** at 1.8 K.

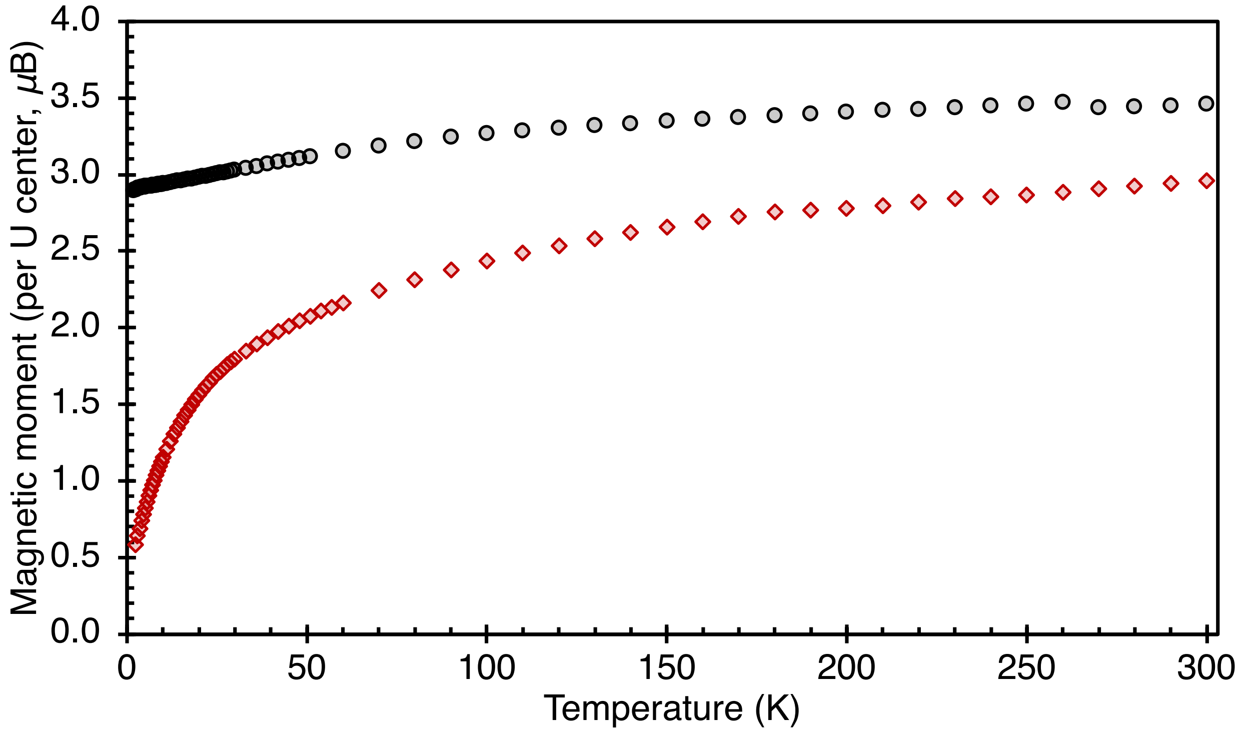

Supplementary Figure 146. Magnetic moments per uranium centre *versus* temperature for **2-crypt** (black shaded circles) and **1** (red shaded diamonds) over the temperature range 2-300 K, measured under applied field of 0.1 T.

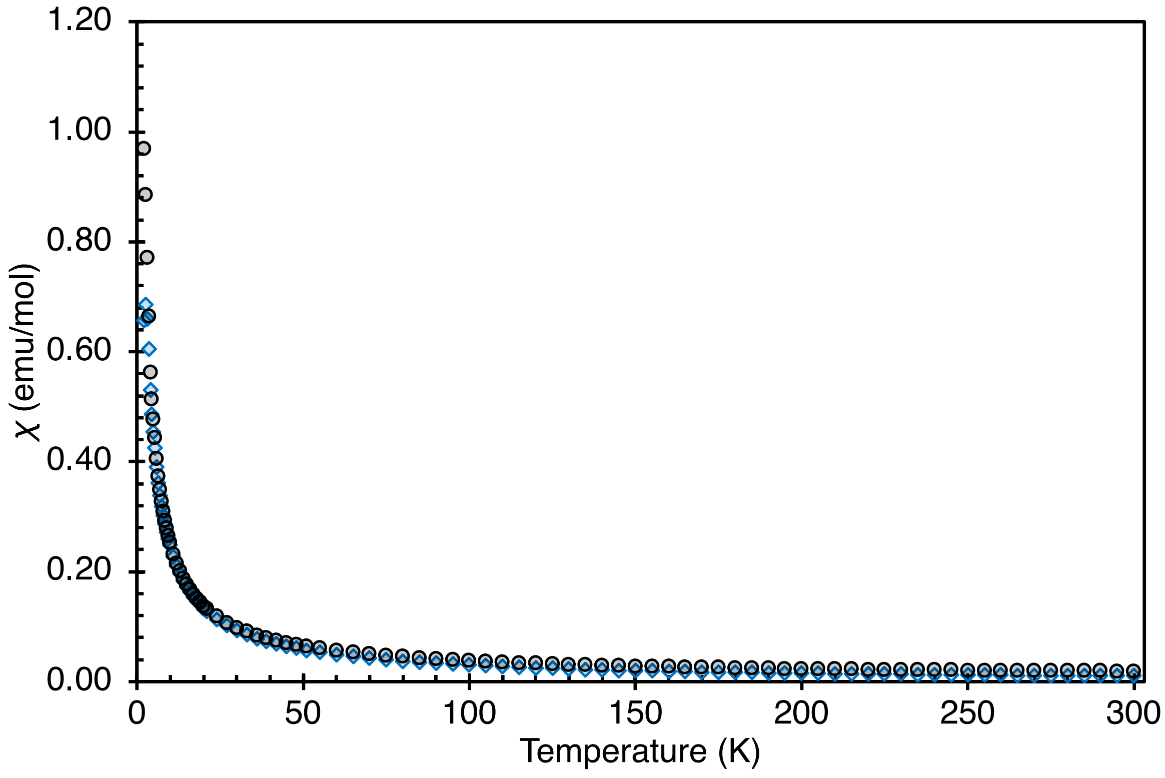

Supplementary Figure 147. Molar magnetic susceptibility *versus* temperature plot for **2-K** over the temperature range 2-300 K, measured under applied field of 0.1 T (black shaded circles) and 1 T (blue shaded diamonds).

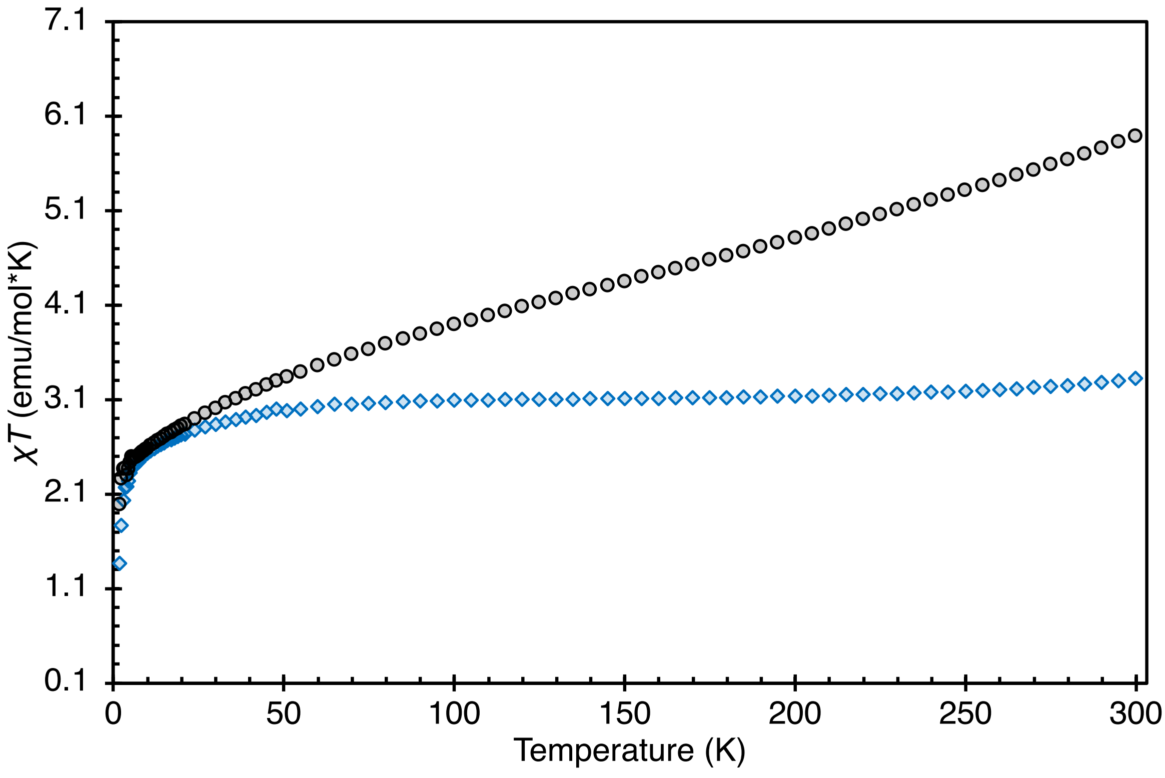

Supplementary Figure 148. Temperature-dependent magnetisation data plot for **2-K** over the temperature range 2-300 K, measured under applied field of 0.1 T (black shaded circles) and 1 T (blue shaded diamonds).

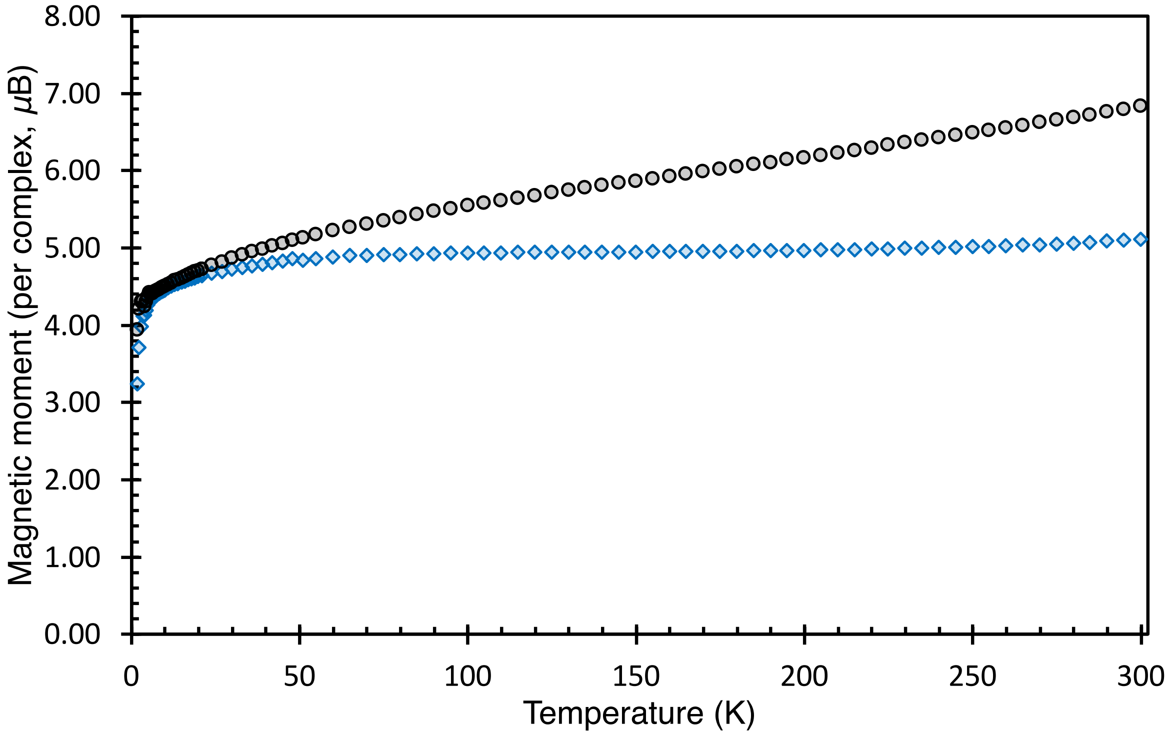

Supplementary Figure 149. Magnetic moment per complex *versus* temperature for **2-K** over the temperature range 2-300 K, measured under applied field of 0.1 T (black shaded circles) and 1 T (blue shaded diamonds).

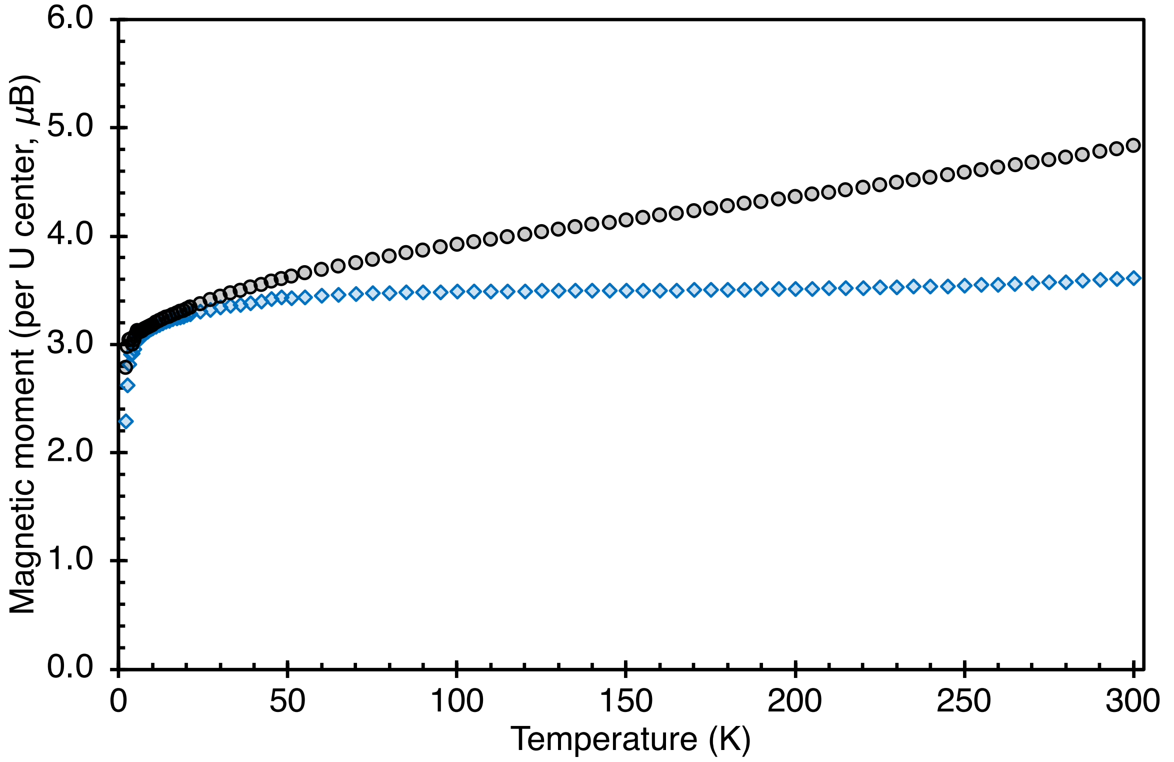

Supplementary Figure 150. Magnetic moment per uranium centre *versus* temperature for **2-K** over the temperature range 2-300 K, measured under applied field of 0.1 T (black shaded circles) and 1 T (blue shaded diamonds).

Density Functional Theory Molecular Orbital (MO) Representations

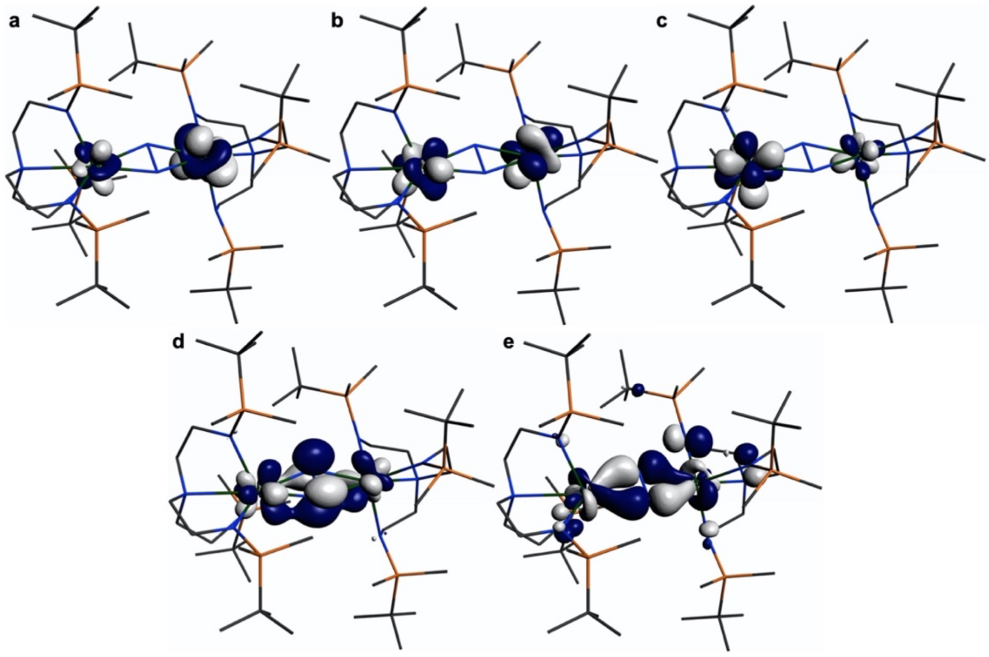

Supplementary Figure 151. Selected α-spin frontier MOs of **1**. a) HOMO (372a, −2.895 eV). b) HOMO−1 (371a, −2.919 eV). c) HOMO−2 (370a, −2.981 eV). d) HOMO−3 (369a, −3.661 eV). e) HOMO−4 (368a, −4.534 eV). The β-spin congener of 368a is very similar. H-atoms are omitted for clarity.

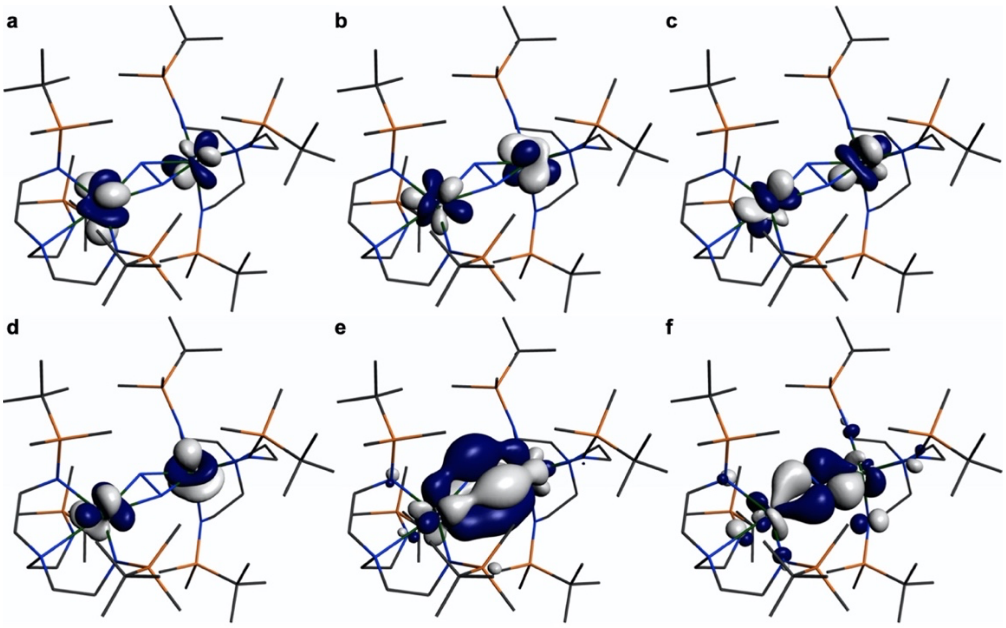

Supplementary Figure 152. Selected α-spin frontier MOs of **2'**. a) HOMO (373a, 0.184 eV). b) HOMO−1 (372a, 0.162 eV). c) HOMO−2 (371a, 0.142 eV). d) HOMO−3 (370a, 0.103 eV). e) HOMO−4 (369a, −0.908 eV). f) HOMO−5 (368a, −1.806 eV) The β-spin congener of 368a is very similar. H-atoms are omitted for clarity.

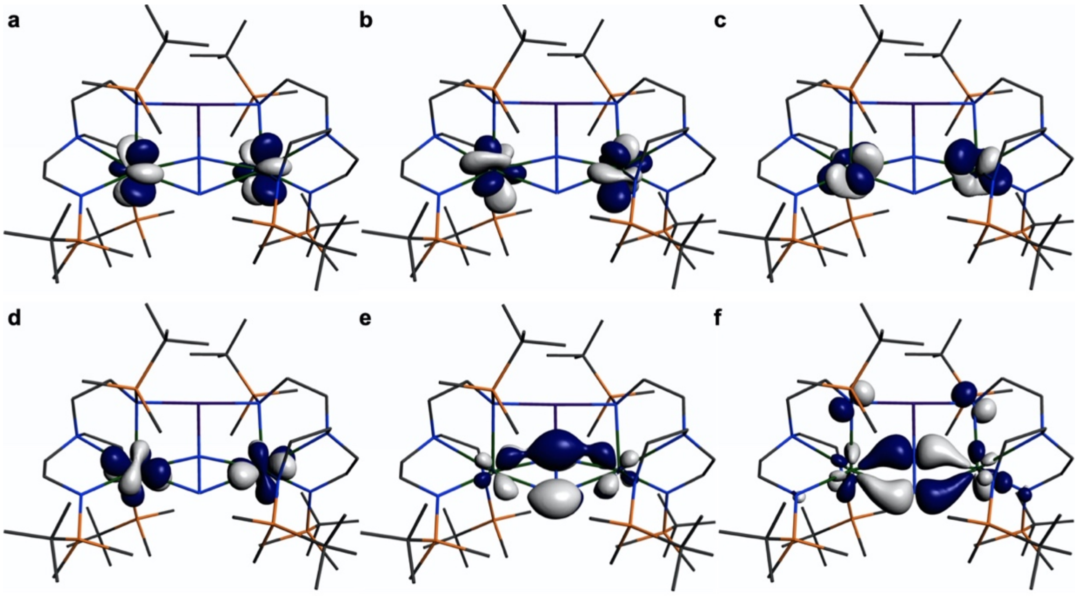

Supplementary Figure 153. Selected α-spin frontier MOs of **2-Li**. a) HOMO (374a, –2.251 eV). b) HOMO–1 (373a, –2.300 eV). c) HOMO–2 (372a, –2.349 eV). d) HOMO–3 (371a, –2.381 eV). e) HOMO–4 (370a, –3.416 eV). f) HOMO–5 (369a, –4.298 eV) The β-spin congener of 369a is very similar. H-atoms are omitted for clarity.

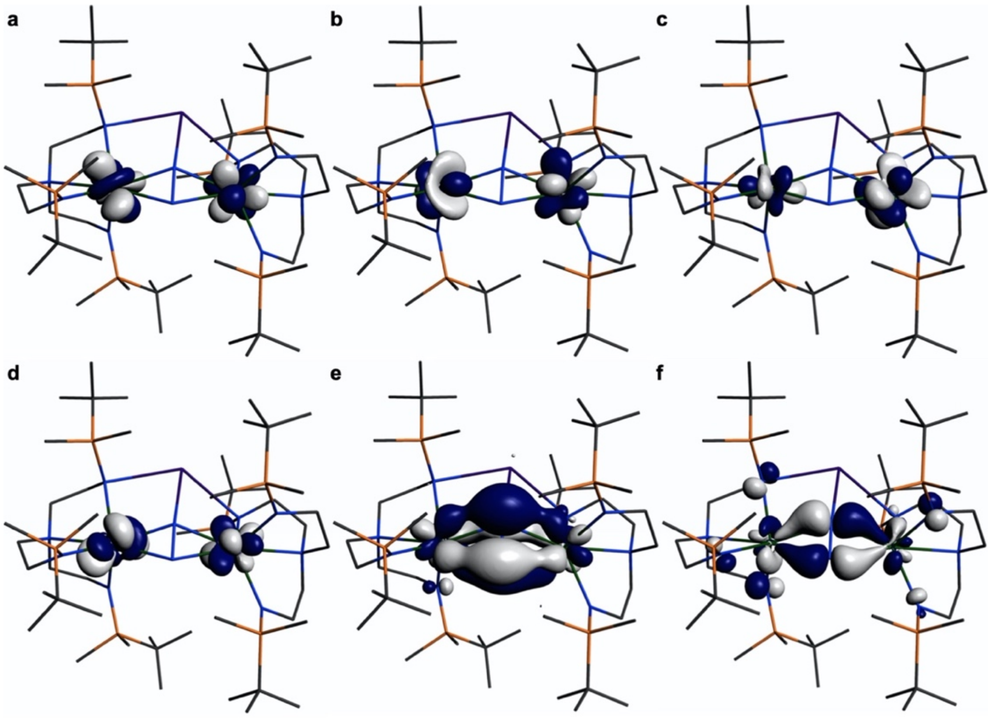

Supplementary Figure 154. Selected α-spin frontier MOs of **2-K**. a) HOMO (382a, –2.363 eV). b) HOMO–1 (381a, –2.390 eV). c) HOMO–2 (380a, –2.426 eV). d) HOMO–3 (379a, –2.438 eV). e) HOMO–4 (378a, –3.462 eV). f) HOMO–5 (377a, –4.268 eV) The β-spin congener of 377a is very similar. H-atoms are omitted for clarity.

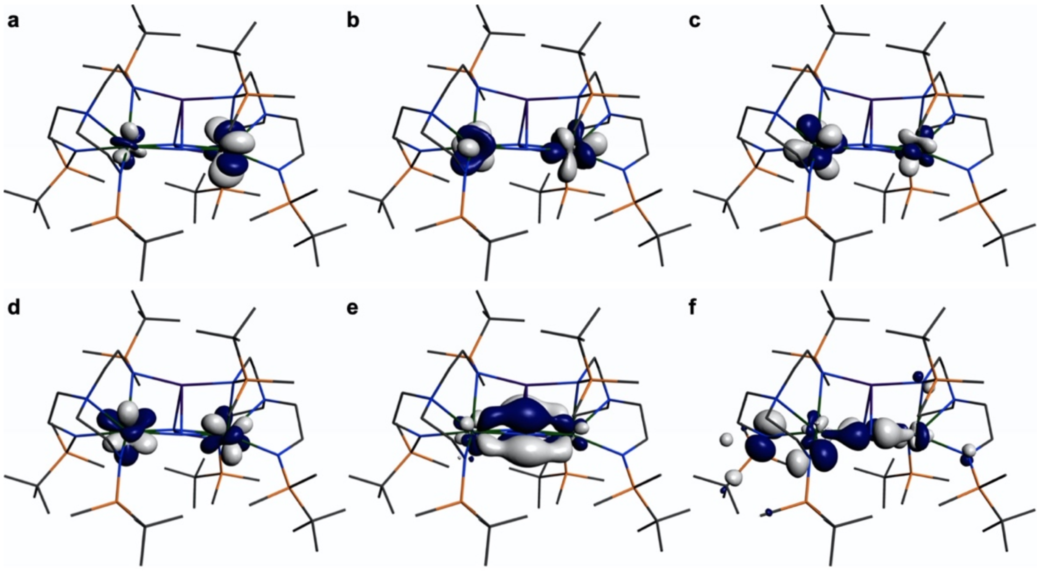

Supplementary Figure 155. Selected α-spin frontier MOs of **3A** (end-on/side-on isomer). a) HOMO (375a, −2.162 eV). b) HOMO−1 (374a, −2.208 eV). c) HOMO−2 (373a, −2.232 eV). d) HOMO−3 (372a, −2.264 eV). e) HOMO−4 (371a, −3.429 eV). f) HOMO−6 (369a, −4.420 eV) The β-spin congeners of 371a and 369a are very similar. H-atoms are omitted for clarity.

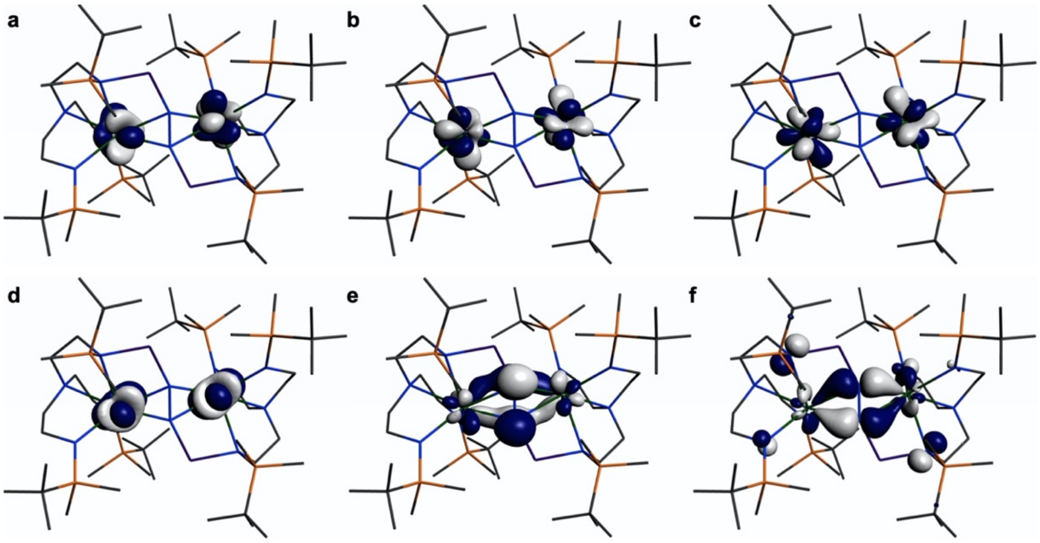

Supplementary Figure 156. Selected α-spin frontier MOs of **3B** (end-on/end-on isomer). a) HOMO (375a, −2.161 eV). b) HOMO−1 (374a, −2.164 eV). c) HOMO−2 (373a, −2.214 eV). d) HOMO−3 (372a, −2.235 eV). e) HOMO−4 (371a, −3.372 eV). f) HOMO−6 (369a, −4.429 eV) The β-spin congeners of 371a and 369a are very similar. H-atoms are omitted for clarity.

SUPPLEMENTARY TABLES

Supplementary Table 1. Selected XRD-derived bond lengths (Å) and angles for the crystallographically characterized complexes **2-crypt**, **2-K**, **2-Li**, **3, 4**, **5.** Previously reported[1b] **1** is also given for reference.

	**1** (from ref.)[1b]	**2-crypt**	**2-K**	**2-Li**	**3**	**4**	**5**
U–$N_{amide, av.}$	2.281	2.327	2.332	2.319	2.402	2.484	2.380
U–$N_{amide, longest}$	2.290(5)	2.342(3)	2.405(11)	2.421(7)	2.516(11)	2.56(1)	2.44(1)
U–$N_{amide, shortest}$	2.264(5)	2.312(4)	2.249(14)	2.250(7)	2.333(10)	2.401(11)	2.27(1)
U–$N_{amine, av.}$	2.411	2.743	2.670	2.653	2.628	2.744	2.659
U–$N_{amine, longest}$	2.601(5)	2.771(3)	2.732(13)	2.654(8)	2.633(10)	2.777(10)	2.71(1)
U–$N_{amine, shortest}$	2.555(5)	2.715(4)	2.608(15)	2.652(8)	2.623(10)	2.71(1)	2.61(1)
U–$N_{diazenido, av.}$	2.419	2.257	2.286	2.289			
U–$N_{diazenido, longest}$	2.444(6)	2.280(3)	2.301(14)	2.325(7)			
U–$N_{diazenido, shortest}$	2.394(6)	2.232(3)	2.267(13)	2.245(7)			
U–$N_{hydrazido, av.}$					2.271		
U–$N_{hydrazido, longest}$					2.379(15)		
U–$N_{hydrazido, shortest}$					2.11(3)		
U–$N_{nitride, av.}$						2.166	2.181
U–$N_{nitride, longest}$						2.248(9)	2.35(1)
U–$N_{nitride, shortest}$						2.211(9)	2.02(1)
U–$N_{imido, av.}$							2.176
U–$N_{imido, longest}$							2.21(1)
U–$N_{imido, shortest}$							2.14(1)
N=$N_{diazenido}$	1.109(7)	1.336(6)	1.364(18)	1.28(1)			
N-$N_{hydrazido}$					1.47(2), 1.483(19)		
N···N						2.70(1)	2.58(2)
(N_{amine}-U-$N_{nitride}$) ∠ °						168.99, 149.25	108.20, 96.26
(U1-N_2-U2)$_{plane}$ ∠ °	177.83	157.43	158.09	179.4			
U···U	4.704	4.228	4.284	4.387	4.124	3.389	3.500

Supplementary Table 2. Selected crystallographic details for complexes **2-crypt**, **2-K**, **2-Li**, **3**, **4**, **5.**

**Compound**	**2-crypt**	**2-K**	**2-Li**	**3**
CCDC number	2377328	2355682	2377329	2377330
Formula	$C_{48}H_{114}N_{10}Si_6U_2$, $C_{18}H_{36}KN_2O_6$, $2(C_7H_8)$	$C_{48}H_{114}KN_{10}Si_6U_2$	$C_{48}H_{114}LiN_{10}Si_6U_2$	$2(C_{48}H_{114}Li_2N_{10}Si_6U_2)$, $C_4H_{10}O$
$D_{calc.}$/ g cm^{-3}	1.359	1.499	1.472	1.473
m/mm^{-1}	10.347	15.335	14.818	14.421
Formula Weight	2075.94	1515.19	1483.03	3054.05
Colour	clear intense brown	clear light green	clear intense brown	clear dark red
Shape	prism	prism	plate	prism
Size/mm^3	0.20×0.07×0.06	0.10×0.02×0.01	0.07×0.06×0.05	0.65×0.10×0.06
T/K	199.99(10)	140.01(10)	200.00(10)	200.00(10)
Crystal System	triclinic	triclinic	monoclinic	monoclinic
Space Group	*P*-1	*P*-1	$P2_1/n$	$P2_1/n$
a/Å	14.4455(4)	11.8521(2)	18.3517(6)	12.8064(4)
b/Å	16.1456(5)	14.2208(3)	17.1041(6)	19.5953(6)
c/Å	21.8982(6)	20.9559(4)	21.8841(8)	27.7043(10)
a/°	94.632(2)	93.2744(16)	90	90
b/°	92.923(2)	96.7694(14)	103.008(3)	97.873(3)
g/°	93.636(2)	106.0202(17)	90	90
V/Å^3	5072.3(3)	3356.31(11)	6692.9(4)	6886.7(4)
Z	2	2	4	2
Z'	1	1	1	0.5
Wavelength/Å	1.54184	1.54184	1.54184	1.54184
Radiation type	CuKα	CuKα	CuKα	CuKα
Q_{min}/°	2.752	2.132	2.845	2.771
Q_{max}/°	72.566	74.775	72.542	73.811
Measured Refl's.	42378	55640	34873	14837
Indep't Refl's	19606	55640	12899	14837
Refl's I≥2 *s*(I)	16408	44625	8844	13672
R_{int}	0.0344	n/a	0.0692	n/a
Parameters	1181	635	634	854
Restraints	631	18	682	1069
Largest Peak	1.931	14.752	1.412	1.971
Deepest Hole	-2.176	-6.318	-2.397	-1.856
GooF	1.006	1.024	1.006	1.066
wR_2 (all data)	0.0689	0.2405	0.0980	0.1876
wR_2	0.0643	0.2305	0.0853	0.1823
R_1 (all data)	0.0381	0.0979	0.0796	0.0635
R_1	0.0289	0.0834	0.0433	0.0597

	**4**	**5**
CCDC number	2377331	2377332
Formula	$C_{56}H_{134}Li_4N_{10}O_2Si_6U_2$	$C_{48}H_{115}Li_3N_{10}Si_6U_2$
$D_{calc.}$/ g cm^{-3}	1.436	1.453
m/mm^{-1}	13.052	14.489
Formula Weight	1652.08	1497.91
Colour	clear dark orange	clear light brown
Shape	plate	plate
Size/mm^3	0.28×0.23×0.11	0.13×0.06×0.05
T/K	140.00(10)	200.00(10)
Crystal System	monoclinic	monoclinic
Space Group	$P2_1/n$	$P2_1/n$
a/Å	23.9982(11)	12.9673(9)
b/Å	13.7738(5)	23.9529(14)
c/Å	24.1880(9)	22.1636(13)
a/°	90	90
b/°	107.143(4)	96.090(6)
g/°	90	90
V/Å^3	7640.0(6)	6845.3(7)
Z	4	4
Z'	1	1
Wavelength/Å	1.54184	1.54184
Radiation type	CuKα	CuKα
Q_{min}/°	3.089	2.725
Q_{max}/°	76.139	66.600
Measured Refl's.	18643	34336
Indep't Refl's	18643	12089
Refl's I≥2 *s*(I)	16502	6905
R_{int}	n/a	0.1341
Parameters	756	652
Restraints	867	795
Largest Peak	3.611	2.619
Deepest Hole	-2.859	-1.469
GooF	1.074	0.977
wR_2 (all data)	0.1837	0.1517
wR_2	0.1793	0.1186
R_1 (all data)	0.0693	0.1294
R_1	0.0626	0.0603

Supplementary Table 3. Stoichiometric acidification experiments for the catalyst-free reactions (entries 26 and 28) or solutions of **1** (entries 27 and 29) at a concentration of 1.3 mM with acids, reductants and $NaNO_2$/$NaNO_3$ under N_2 to produce NH_3 and N_2H_4. Direct acidifications of **2-crypt**, **3** and **4** with the phosphonium acid in an absence of a reducing agent under Ar (entries 30-32); in presence of a reducing agent under Ar (entries 33-35).

Entry[b]	Compound	Solvent	Acid	Reductant	Acid [eq.]	Reductant [eq.]	Additional Reagents[c]	NH_3 [eq.]	N_2H_4 [eq.]	Fixed-N [eq.]
26 (N_2)	-	Et_2O	[Cy_3PH][I]	KC_8	600	600	$NaNO_3$	0.26	0.02	0.30
27 (N_2)	**1**	Et_2O	[Cy_3PH][I]	KC_8	600	600	$NaNO_3$	0.29	0.01	0.31
28 (N_2)	-	Et_2O	[Cy_3PH][I]	KC_8	600	600	$NaNO_2$	0.39	0.00	0.39
29 (N_2)	**1**	Et_2O	[Cy_3PH][I]	KC_8	600	600	$NaNO_2$	0.53	0.00	0.53
30 (Ar)	**2-crypt**	Et_2O	[Cy_3PH][I]	-	600	-	-	0.00	-	0.00
31 (Ar)	**3**	Et_2O	[Cy_3PH][I]	-	600	-	-	<0.01	-	<0.01
32 (Ar)	**4**	Et_2O	[Cy_3PH][I]	-	600	-	-	0.40	-	0.40
33 (Ar)	**2-crypt**	Et_2O	[Cy_3PH][I]	RbC_8	600	600	-	1.32	-	1.32
34 (Ar)	**3**	Et_2O	[Cy_3PH][I]	RbC_8	600	600	-	0.91	-	0.91
35 (Ar)	**4**	Et_2O	[Cy_3PH][I]	RbC_8	600	600	-	1.95	-	1.95

[a] All reactions conducted twice to ensure reproducibility. [b] Experiments performed under 1.31 bar N_2 at −78 °C followed by warming to 25 °C and then stirring for 17 hr. [c] Both $NaNO_2$ and $NaNO_3$ were added in a 1-equivalent ratio to **1** to their respective reactions.

Supplementary Table 4. Computed data for **1**, **2'**, **2-Li**, **2-K**, **3A**, **3B**

Entry	MDC_q[a]		MDC_m[b]		Bond Order[c]		U-N QTAIM[d]				N_2 QTAIM			
	U[e]	N_2	U[e]	N_2	U-N	N-N	ρ	$\nabla^2\rho$	H	ε	ρ	$\nabla^2\rho$	H	ε
**1**	2.42	−1.88	2.09	0.25	0.91	1.66	0.09	0.21	−0.03	0.12	0.40	−0.64	−0.67	0.04
**2'**	2.22	−2.01	2.51	0.34	0.95	1.57	0.09	0.22	−0.04	0.14	0.37	−0.51	−0.59	0.05
**2-Li**	2.13	−2.14	2.53	0.37	0.89	1.64	0.08	0.22	−0.03	0.26	0.38	−0.59	−0.63	0.05
**2-K**	1.97	−2.01	2.52	0.36	0.90	1.61	0.08	0.22	−0.03	0.16	0.37	−0.55	−0.61	0.05
**3A**	1.90	−2.68	2.31	−0.29	0.98	1.27	0.09	0.24	−0.04	0.12	0.28	−0.16	−0.35	0.10
**3B**	2.14	−2.73	2.31	−0.30	0.96	1.32	0.09	0.24	−0.04	0.37	0.29	−0.22	−0.39	0.09

[a] Multipole derived charge. [b] Multipole derived net spin density. [c] Nalewajski-Mrozek bond order. [d] Average QTAIM value for the U-N_2 bonding interactions. [e] Average of both uranium ions. [f] Total for the N_2 unit.

SUPPLEMENTARY REFERENCES

1. a) Roussel, P., Hitchcock, P. B., Tinker, N. & Scott, P., A mixed-valence uranium(III/IV) bimetallic; structure, magnetism and reactivity. *Chem. Commun.* **17**, 2053-2054 (1996), b) Roussel, P. & Scott, P. Complex of dinitrogen with trivalent uranium. *J. Am. Chem. Soc.* **120**, 1070-1071 (1998).
2. Bergbreiter D. E. & Killough J. M., Reactions of potassium-graphite. *J. Am. Chem. Soc.* **100**, 2126-2134 (1978).
3. Maity, S & Hoz, S., Deciphering a 20-Year-Old Conundrum: The Mechanisms of Reduction by the Water/Amine/SmI_2 Mixture. *Chem. Eur. J.* **21**, 18394-18400 (2015).
4. Doyle, L. R., Wooles, A. J., Jenkins, L. C., Tuna, F., McInnes, E. J. L. & Liddle, S. T., Catalytic Dinitrogen Reduction to Ammonia at a Triamidoamine–Titanium Complex. *Angew. Chem. Int. Ed.*, **57**, 6314-6318 (2018).
5. Bain, G. A. & Berry, J. F., Diamagnetic Corrections and Pascal's Constants. *J. Chem. Educ.* **85**, 532-536 (2008);
6. Fonseca Guerra, C., Snijders, J. G., Te Velde, G. & Baerends, E. J. Towards an order-N DFT Method. *Theor. Chem. Acc.* **99**, 391-403 (1998).
7. Te Velde, G., Bickelhaupt, F. M., Baerends, E. J., Fonseca Guerra, C., Van Gisbergen, S. J. A., Snijders, J. G. & Ziegler, T. Chemistry with ADF. *J. Comput. Chem.* **22**, 931-967 (2001).
8. Van Lenthe, E., Baerends, E. J. & Snijders, J. G. Relativistic regular two-component Hamiltonians. *J. Chem. Phys.* **99**, 4597-4610 (1993).
9. Van Lenthe, E., Baerends, E. J. & Snijders, J. G. Relativistic total energy using regular approximations. *J. Chem. Phys.* **101**, 9783-9792 (1994).
10. Van Lenthe, E., Ehlers, A. E. & Baerends, E. J. Geometry optimization in the Zero Order Regular Approximation for relativistic effects. *J. Chem. Phys.* **110**, 8943-8953 (1999).
11. Vosko, S. H., Wilk, L. & Nusair, M. Accurate spin-dependent electron liquid correlation energies for local spin density calculations: a critical analysis. *Can. J. Phys.* **58**, 1200-1211 (1980).
12. Becke, A. D. Density-functional exchange-energy approximation with correct asymptotic behaviour. *Phys. Rev. A.* **38**, 3098-3100 (1988).
13. Perdew, J. P. Density-functional approximation for the correlation energy of the inhomogeneous electron gas. *Phys. Rev. B.* **33**, 8822-8824 (1986).
14. Bader, R. F. W. *Atoms in Molecules: A Quantum Theory*, Oxford University Press, New York, 1990.
15. Bader, R. F. W. A bond path: a universal indicator of bonded interactions. *J. Phys. Chem. A* **102**, 7314-7323 (1998).
16. Motta, L. C. & Autschbach, J. Actinide inverse trans influence versus cooperative pushing from below and multi-center bonding. *Nat. Commun.* **14**, 4307 (2023).
17. *CrysAlis*^{Pro}, Rigaku Oxford Diffraction, release 1.171.41.113a, 2021.
18. G. M. Sheldrick, SHELXT - Integrated space-group and crystal-structure determination. *Acta Cryst. Section A* **71**, 3-8 (2015).
19. G. M. Sheldrick, Crystal structure refinement with SHELXL. *Acta Cryst. Section C* **71**, 3-8 (2015).
20. Camp, C., Pecaut, J. & Mazzanti, M. Tuning Uranium-Nitrogen Multiple Bond Formation with Ancillary Siloxide Ligands. *J. Am. Chem. Soc.* **135**, 12101-12111.
21. Palumbo, C. T. *et al.* Tuning the structure, reactivity and magnetic communication of nitride-bridged uranium complexes with the ancillary ligands. *Chem. Sci.* **10**, 8840-8849 (2019).
22. Barluzzi, L. *et al.* Synthesis, structure, and reactivity of uranium(VI) nitrides. *Chem. Sci.* **12**, 8096-8104 (2021).
23. King, D. M. *et al.* Uranium-nitride chemistry: uranium-uranium electronic communication mediated by nitride bridges. *Dalton Trans.i* **51**, 8855-8864 (2022).
