# Supplementary material for: Catalytic and stoichiometric stepwise conversion of side-on bound dinitrogen to ammonia mediated by a uranium complex
Source: Nat Chem. 2025 Jul 16;17(9):1425–33. doi: 10.1038/s41557-025-01867-z (PMC12411223; doi:10.1038/s41557-025-01867-z)
Supplement: Supplementary file 2 — Geometry optimized coordinates and single point energy of 1. [file 41557_2025_1867_MOESM2_ESM.xyz]

180Complex 1 Energy -977.12374414 eV   1.C         4.203249   -0.314970   -5.741004   2.C        -1.955595   -4.886037   -4.415113   3.C         3.899124   -0.898558   -4.343464   4.C         2.483429   -1.506577   -4.356479   5.C        -4.126022    2.218140   -4.255761   6.C         4.922626   -2.011666   -4.034603   7.C        -4.125888   -2.110673   -3.822415   8.C         5.591663    1.481686   -3.297628   9.C        -1.118107   -1.868378   -3.292112  10.C        -3.918276   -5.355546   -2.935981  11.C         2.524950    1.677645   -3.268112  12.C        -2.544917   -4.648255   -3.004213  13.C        -2.631334    5.192445   -2.869723  14.C        -1.314222    2.131861   -3.111709  15.C        -1.605011   -5.283960   -1.962840  16.C        -3.351526    4.184088   -1.945204  17.C        -4.841920    4.581943   -1.864754  18.C        -5.047661    1.069361   -1.240614  19.C         1.890588   -3.538229   -0.985861  20.C         5.183630   -0.645745   -0.839854  21.C        -5.496304   -0.295162   -0.721043  22.C        -2.729483    4.271126   -0.537776  23.C        -4.126786   -2.919082   -0.436594  24.C         1.322681    3.931346   -0.454139  25.C         4.365622    3.928251   -0.187227  26.C         5.666136    0.126236    0.388778  27.C        -4.694075   -2.135797    0.746722  28.C         3.856011   -4.657123    1.063056  29.C         2.469178    6.384044    1.439903  30.C         0.753729   -5.939532    1.373516  31.C        -0.490799   -3.767054    1.478534  32.C        -5.003830    0.132283    1.669004  33.C         0.844755   -4.459408    1.807541  34.C         2.633572    5.015286    2.147070  35.C        -3.815935    1.058212    1.927759  36.C         4.814182   -1.282314    2.213327  37.C         4.658466    1.165441    2.431250  38.C         3.214425    1.630779    2.622944  39.C         3.444371   -1.830920    2.603120  40.C         3.910748    5.100740    3.015188  41.C         1.405008    4.783284    3.051536  42.C         1.073923   -4.397838    3.332806  43.C        -0.055151   -0.253534    3.479192  44.C        -2.588428   -1.885417    4.106819  45.C        -2.052071    2.516393    4.663643  46.C        -2.394461    1.057148    5.031867  47.C        -3.875838    0.955813    5.466634  48.C        -1.537562    0.663032    6.261916  49.H         4.097749   -1.101574   -6.510267  50.H         3.510480    0.497647   -6.011921  51.H         5.228735    0.078500   -5.812648  52.H        -2.581765   -4.447147   -5.208277  53.H         2.424857   -2.338095   -5.082299  54.H        -3.780018    2.959471   -4.993984  55.H        -3.976467   -2.418142   -4.869646  56.H        -1.891074   -5.971339   -4.615141  57.H         4.872233   -2.802380   -4.805148  58.H        -3.975677    1.221041   -4.697035  59.H        -0.940716   -4.472589   -4.517212  60.H         1.722166   -0.765339   -4.643481  61.H         5.518048    2.046157   -4.240685  62.H        -1.034841   -1.908853   -4.388861  63.H         5.957238   -1.633359   -4.024383  64.H         2.411226    1.922926   -4.335421  65.H        -5.207948    2.368115   -4.127295  66.H        -4.628580   -4.955062   -3.675241  67.H        -3.005719    5.149405   -3.904943  68.H        -4.173539   -1.012005   -3.796242  69.H        -5.108867   -2.497780   -3.512171  70.H         6.482449    0.839604   -3.375820  71.H        -1.177892    1.187155   -3.657752  72.H        -0.984321    2.939530   -3.782291  73.H        -3.794872   -6.432650   -3.151279  74.H         2.203871   -1.910046   -3.372536  75.H        -1.095048   -0.799398   -3.021477  76.H         4.726593   -2.487875   -3.062465  77.H         1.564393    1.257421   -2.933313  78.H        -1.544244    5.025498   -2.895240  79.H        -5.331223    4.552865   -2.850406  80.H        -0.213334   -2.328188   -2.872405  81.H        -2.797399    6.222391   -2.504824  82.H         5.775261    2.210868   -2.493721  83.H         2.682824    2.625987   -2.735258  84.H        -4.384389   -5.275794   -1.943311  85.H        -1.477607   -6.362595   -2.168102  86.H        -0.637767    2.120015   -2.247143  87.H        -5.637651    1.271578   -2.152836  88.H        -0.605341   -4.826420   -1.979749  89.H        -4.937210    5.614514   -1.481975  90.H         5.990637   -0.595807   -1.590231  91.H        -5.323446   -1.040208   -1.504583  92.H         2.731352   -3.114230   -1.555422  93.H         1.709404   -4.551305   -1.374458  94.H        -5.412871    3.930195   -1.186895  95.H        -4.937983   -3.080238   -1.172906  96.H         1.516913    4.797799   -1.104258  97.H        -1.997205   -5.185893   -0.940733  98.H         0.996962   -2.932315   -1.191223  99.H         4.456615    3.191216   -0.998280 100.H         1.159262    3.061009   -1.104514 101.H         4.400071    4.930335   -0.643317 102.H         5.085202   -1.715214   -0.586643 103.H        -6.578391   -0.282251   -0.479974 104.H        -1.670131    3.975065   -0.539296 105.H        -5.356252    1.846042   -0.512622 106.H        -2.781445    5.305636   -0.152652 107.H        -3.869044   -3.927416   -0.062808 108.H         0.380191    4.114651    0.081386 109.H         5.743630    1.186305    0.117193 110.H        -3.253909    3.625156    0.181503 111.H         0.597769   -6.045585    0.288635 112.H         5.257059    3.828047    0.451220 113.H        -0.724849   -3.787337    0.406475 114.H         3.692075   -5.644451    0.603329 115.H         6.667275   -0.224152    0.712600 116.H         1.552843    6.434665    0.833108 117.H         3.323262    6.617204    0.784432 118.H        -5.705147   -2.504607    1.011641 119.H         4.721003   -4.202098    0.555355 120.H        -3.774781    1.792000    1.097099 121.H        -5.940510    0.696014    1.521567 122.H         1.655916   -6.508870    1.644877 123.H        -0.104532   -6.425647    1.873358 124.H        -4.048658   -2.272911    1.625943 125.H         5.280542   -2.005538    1.531843 126.H         2.409854    7.188725    2.195408 127.H        -0.485621   -2.714218    1.787586 128.H        -1.318548   -4.273862    2.007666 129.H         5.245197    1.985478    1.997134 130.H         4.137458   -4.832847    2.112690 131.H         4.810377    5.276686    2.405659 132.H         0.469840    4.760975    2.471755 133.H        -5.151159   -0.539103    2.526038 134.H         0.284855   -0.905171    2.659665 135.H        -3.998014    1.667417    2.824527 136.H         5.481216   -1.165432    3.089703 137.H         3.595595   -2.799132    3.110684 138.H         2.645432    0.811443    3.105272 139.H         5.126740    0.892129    3.394227 140.H         3.182477    2.449576    3.359401 141.H         1.468237    3.837277    3.609119 142.H         2.975661   -1.181525    3.364406 143.H         3.826932    5.945014    3.723517 144.H         4.086480    4.196330    3.615897 145.H        -2.294800   -2.656585    3.379984 146.H         2.004830   -4.903545    3.634848 147.H         1.312958    5.599197    3.791166 148.H         1.114391   -3.359905    3.695561 149.H         0.243799   -4.899838    3.863180 150.H        -3.685964   -1.905931    4.194934 151.H         0.354259    0.753182    3.303860 152.H        -2.651191    2.896460    3.823682 153.H         0.375459   -0.645003    4.412643 154.H        -0.993600    2.626529    4.384484 155.H        -4.583940    1.251844    4.678692 156.H        -2.176493   -2.187537    5.083170 157.H        -2.235702    3.181725    5.526738 158.H        -4.138680   -0.065621    5.781767 159.H        -0.460426    0.786379    6.076301 160.H        -4.060168    1.620520    6.330099 161.H        -1.710701   -0.378510    6.575446 162.H        -1.800516    1.309076    7.119211 163.N        -3.591704    1.123384   -1.496429 164.N         3.900127   -0.100732   -1.350983 165.N        -2.963997   -2.242478   -1.038769 166.N         0.042325   -0.770508   -0.007684 167.N         0.031783    0.417893   -0.538879 168.N        -4.694160   -0.684957    0.461614 169.N         2.596167    1.985238    1.336512 170.N         4.676240    0.010464    1.490636 171.N         2.602640   -1.932410    1.396872 172.N        -2.552761    0.294966    1.990250 173.Si        3.982893    0.500559   -3.007181 174.Si       -2.702584   -2.730460   -2.717090 175.Si       -3.127328    2.386000   -2.645056 176.Si        2.739100    3.647271    0.765613 177.Si        2.290398   -3.583448    0.861534 178.Si       -1.941193   -0.170700    3.581738 179.U        -2.205706   -0.206294   -0.219010 180.U         2.230810    0.002068    0.223162 
