# Supplementary material for: Catalytic and stoichiometric stepwise conversion of side-on bound dinitrogen to ammonia mediated by a uranium complex
Source: Nat Chem. 2025 Jul 16;17(9):1425–33. doi: 10.1038/s41557-025-01867-z (PMC12411223; doi:10.1038/s41557-025-01867-z)
Supplement: Supplementary file 3 — Geometry optimized coordinates and single point energy of 2′. [file 41557_2025_1867_MOESM3_ESM.xyz]

180Complex 2prime.Energy: -978.80895743 eV   1.C         1.114985    3.215002   -5.571250   2.C        -5.327516   -1.935335   -4.552946   3.C         3.770725    1.426050   -4.754627   4.C        -1.895592   -2.088604   -4.357149   5.C        -4.579163   -4.210459   -3.829547   6.C         1.263019    3.115554   -4.037392   7.C         1.223600    0.006271   -3.855202   8.C         1.946527    4.398662   -3.520923   9.C        -4.748118   -2.743437   -3.372016  10.C         3.591071   -4.457203   -3.207839  11.C        -0.145211    3.018902   -3.419347  12.C        -3.484911    0.688756   -2.817051  13.C         1.186636   -4.055195   -2.639490  14.C        -5.753208   -2.716914   -2.205363  15.C        -4.752452    1.259191   -2.170566  16.C         2.523401   -4.598446   -2.102081  17.C         2.357198   -6.097577   -1.770968  18.C         4.019483    2.346741   -1.615084  19.C         4.650321   -1.665716   -1.502653  20.C         5.244847    1.452250   -1.412723  21.C        -2.326187   -3.243478   -1.511870  22.C        -3.053243    3.519365   -0.794330  23.C         5.615902   -0.850241   -0.641747  24.C        -4.418843    2.955211   -0.414774  25.C        -0.670565    5.568187   -0.176271  26.C         4.496971   -4.492629    0.295415  27.C        -5.461100    0.809526    0.167279  28.C         1.564786   -3.676654    0.720524  29.C         0.924641    3.179904    0.782691  30.C         5.473399    1.008106    0.999081  31.C        -4.871119    0.593646    1.560472  32.C         4.291682    1.585078    1.780481  33.C        -5.116718   -2.714027    2.104223  34.C        -2.043053   -2.582376    2.245549  35.C        -2.722770    5.009676    2.580869  36.C        -1.381659    4.248290    2.553398  37.C        -0.318749    5.098104    3.281342  38.C        -1.555266    2.912938    3.302111  39.C         4.612079   -2.097347    3.667034  40.C         1.295782   -0.870927    3.677087  41.C        -3.733626   -1.243739    4.469811  42.C         4.278926   -0.846218    4.503663  43.C         5.564789   -0.014207    4.699076  44.C        -4.884080   -0.283246    4.839227  45.C         2.564978    1.744641    4.643509  46.C        -2.404520   -0.620106    4.939428  47.C        -3.955592   -2.573555    5.225912  48.C         3.791794   -1.297902    5.898444  49.H         2.083210    3.368782   -6.073089  50.H         0.652495    2.311469   -6.000924  51.H         0.465361    4.071335   -5.835933  52.H         3.409274    1.338394   -5.791902  53.H        -4.645844   -1.927415   -5.417855  54.H        -6.284080   -2.378406   -4.891358  55.H        -2.188814   -1.321682   -5.092147  56.H        -1.920070   -3.065461   -4.866069  57.H         4.438808    2.301313   -4.714549  58.H        -5.553879   -4.624968   -4.150278  59.H         0.783618    0.028901   -4.864702  60.H        -3.887938   -4.302299   -4.681251  61.H         4.377765    0.531018   -4.543859  62.H        -5.533440   -0.889032   -4.279294  63.H         3.297922   -5.050357   -4.095073  64.H         2.951679    4.535911   -3.952282  65.H        -0.853865   -1.883813   -4.071589  66.H         1.349253    5.289083   -3.795046  67.H        -3.684158    0.486065   -3.885709  68.H        -0.705614    2.166162   -3.831235  69.H        -0.726844    3.933480   -3.641733  70.H         3.701833   -3.413443   -3.535626  71.H         1.832137   -0.907906   -3.780013  72.H         0.912192   -4.558753   -3.585473  73.H        -4.200088   -4.852535   -3.020031  74.H         4.580313   -4.820546   -2.884831  75.H         0.402494   -0.081133   -3.128966  76.H         2.013276   -6.649562   -2.666577  77.H        -2.724686    1.500072   -2.832804  78.H         1.235528   -2.974320   -2.836270  79.H        -5.073671    2.190683   -2.672336  80.H        -6.731426   -3.125627   -2.523025  81.H         2.045500    4.391304   -2.425669  82.H         4.262947    3.051233   -2.432886  83.H         4.484022   -1.131393   -2.456743  84.H         5.395533    0.889353   -2.343764  85.H        -0.123586    2.899672   -2.326252  86.H        -5.568119    0.528747   -2.269280  87.H        -5.923120   -1.695793   -1.835045  88.H         5.181100   -2.594356   -1.780723  89.H         0.365871   -4.221849   -1.927789  90.H        -2.940897    3.472934   -1.896684  91.H         3.303830   -6.557110   -1.445035  92.H        -2.092715   -4.229080   -1.940300  93.H        -5.407668   -3.321465   -1.353276  94.H         6.603565   -0.748911   -1.141122  95.H         6.160942    2.053014   -1.229048  96.H        -0.396339    5.417499   -1.232032  97.H         1.610321   -6.266756   -0.978981  98.H        -5.235302    3.514052   -0.921164  99.H        -1.397996   -2.837319   -1.082307 100.H         3.879670    2.990114   -0.725716 101.H        -3.086994    4.598825   -0.561884 102.H         5.362465   -4.573429   -0.381673 103.H        -3.043365   -3.395420   -0.692363 104.H        -1.583435    6.182948   -0.153051 105.H        -5.650065   -0.176900   -0.273099 106.H         1.266029    2.912546   -0.233486 107.H         5.764937   -1.394642    0.299766 108.H         0.133274    6.158994    0.292587 109.H        -6.428536    1.354690    0.231031 110.H         4.225456   -5.516323    0.600375 111.H        -4.550075    3.077995    0.668771 112.H         0.749432   -2.989327    0.448842 113.H         6.274219    1.765165    0.872212 114.H         1.137155   -4.689859    0.775784 115.H         4.826052   -3.953769    1.197196 116.H        -5.094641   -2.947339    1.028667 117.H         1.615748    3.944543    1.172733 118.H         3.923144    2.490184    1.257373 119.H         5.890043    0.176014    1.581662 120.H         1.012421    2.292734    1.428404 121.H        -2.094515   -3.151674    1.306515 122.H         1.903307   -3.410159    1.732787 123.H        -3.524926    4.438027    2.089411 124.H        -2.651946    5.990270    2.083733 125.H        -4.722790    1.574730    2.051237 126.H        -5.667579    0.103387    2.150773 127.H        -6.089010   -2.241452    2.317772 128.H        -1.158335   -1.929436    2.191337 129.H        -5.088541   -3.667530    2.655437 130.H        -0.158426    6.070633    2.788701 131.H         4.688178    1.968431    2.739746 132.H         4.915800   -1.831084    2.644237 133.H        -2.303253    2.267882    2.815212 134.H        -1.865626   -3.307272    3.055795 135.H         0.652219    4.583096    3.334151 136.H        -3.042853    5.191665    3.624692 137.H         1.427285   -1.871659    3.241056 138.H        -0.614875    2.344129    3.349737 139.H         0.489313   -0.380131    3.116130 140.H         6.015550    0.291380    3.743372 141.H         3.746130   -2.771045    3.582388 142.H         5.434233   -2.674792    4.130724 143.H        -0.636866    5.304273    4.321105 144.H        -4.739521    0.715125    4.402093 145.H        -1.886903    3.086853    4.342529 146.H        -3.209452   -3.339295    4.957741 147.H        -2.193402    0.325789    4.420415 148.H        -5.866302   -0.660868    4.511321 149.H         1.692555    2.289520    4.251192 150.H         3.418767    2.439955    4.627861 151.H         0.965212   -0.999951    4.718849 152.H         5.379662    0.897392    5.289214 153.H         6.326154   -0.607810    5.241281 154.H        -1.552049   -1.291577    4.762359 155.H        -4.954191   -2.995425    5.028645 156.H        -4.933528   -0.156951    5.937898 157.H         2.356632    1.501395    5.698023 158.H        -3.876336   -2.408018    6.317851 159.H        -2.439924   -0.407494    6.024595 160.H         2.898145   -1.937185    5.837276 161.H         4.582057   -1.881740    6.408740 162.H         3.548734   -0.441813    6.547738 163.N        -2.999176   -0.476412   -2.072018 164.N         2.827823    1.526211   -1.880436 165.N         3.373958   -1.921070   -0.805034 166.N        -4.510652    1.512425   -0.722868 167.N         0.047258   -0.425215   -0.603589 168.N         5.041988    0.476051   -0.314889 169.N        -1.951733    2.811852   -0.112001 170.N         0.067098   -0.003198    0.656847 171.N        -3.615939   -0.182715    1.528036 172.N         3.222870    0.585602    1.947508 173.Si        2.289741    1.533421   -3.541520 174.Si       -3.004475   -2.047139   -2.810948 175.Si        3.007141   -3.596768   -0.502911 176.Si       -0.835833    3.880861    0.717273 177.Si       -3.629256   -1.596695    2.551784 178.Si        2.868202    0.170726    3.602528 179.U         2.264363    0.022116   -0.148140 180.U        -2.055694    0.441649   -0.124196 
