# Supplementary material for: Catalytic and stoichiometric stepwise conversion of side-on bound dinitrogen to ammonia mediated by a uranium complex
Source: Nat Chem. 2025 Jul 16;17(9):1425–33. doi: 10.1038/s41557-025-01867-z (PMC12411223; doi:10.1038/s41557-025-01867-z)
Supplement: Supplementary file 4 — Geometry optimized coordinates and single point energy of 2-Li. [file 41557_2025_1867_MOESM4_ESM.xyz]

181Complex 2-Li. Energy: -979.99017913 eV    1.C        -4.467762   -1.517543   -5.199356   2.C        -3.504071   -3.818526   -4.989625   3.C         4.611568   -3.328609   -4.691050   4.C         4.137522   -0.866120   -4.611774   5.C        -1.015093   -1.505607   -4.545844   6.C         0.872115    4.507812   -4.259058   7.C        -3.912930   -2.554536   -4.198185   8.C         6.368708   -1.742700   -3.868766   9.C         4.849789   -2.027303   -3.890734  10.C        -5.028679   -2.941550   -3.208297  11.C         3.737336    3.061223   -3.087434  12.C         1.229819    1.285520   -3.144220  13.C         0.965142    4.357744   -2.722870  14.C        -3.060400    0.811684   -2.829066  15.C        -4.450156    1.209706   -2.321945  16.C         2.343425   -2.816913   -2.203340  17.C         1.585848    5.647162   -2.141521  18.C        -0.462956    4.208966   -2.168559  19.C        -1.742562   -3.222390   -2.070992  20.C         5.167547   -3.587979   -1.222186  21.C         5.406279   -0.095639   -0.905934  22.C        -5.599739    0.134174   -0.416639  23.C        -4.622149    2.381889   -0.153045  24.C        -3.256939    3.069259   -0.054752  25.C         3.052259    3.245682    0.188613  26.C         4.448801    2.626854    0.294585  27.C         5.536477    0.425126    0.525057  28.C        -5.379962   -0.437821    0.983849  29.C        -4.939536   -3.940467    1.115756  30.C         1.936072   -3.261788    1.838547  31.C         0.160103    4.224058    2.141571  32.C        -2.138739   -3.052163    2.037183  33.C        -1.975449    5.495730    2.442689  34.C         4.270990    1.373401    2.419745  35.C        -1.204616    4.220867    2.851080  36.C         2.920029    0.805567    2.868434  37.C         5.145445   -2.914861    3.066924  38.C        -3.894589    2.769275    3.228984  39.C        -1.293626    1.132425    3.213177  40.C        -6.170865   -2.396711    3.877260  41.C        -4.627834   -2.478866    3.840710  42.C         4.010516   -2.595335    4.059054  43.C        -0.956313    4.264839    4.376479  44.C         1.046011   -1.757044    4.398606  45.C        -4.046683   -1.245483    4.558674  46.C        -4.191258   -3.748276    4.607511  47.C         3.648747   -3.890642    4.822241  48.C         4.516081   -1.556115    5.083161  49.H        -5.315105   -1.947118   -5.765142  50.H         5.012462   -3.219430   -5.715546  51.H        -4.380656   -4.230031   -5.523045  52.H        -3.710260   -1.205701   -5.935248  53.H        -2.733268   -3.603871   -5.745465  54.H         4.542364   -0.739993   -5.632569  55.H         6.756606   -1.680945   -4.902175  56.H         3.541097   -3.568809   -4.782555  57.H        -0.752290   -2.401111   -5.130229  58.H        -1.326821   -0.726139   -5.259192  59.H         3.056126   -1.044988   -4.711288  60.H        -4.842485   -0.612552   -4.697034  61.H         1.860384    4.646013   -4.722075  62.H         0.396639    3.636167   -4.734476  63.H         0.263948    5.394128   -4.516645  64.H        -3.119984   -4.613121   -4.331556  65.H         5.115399   -4.195566   -4.235572  66.H         3.636094    3.267281   -4.164340  67.H         4.270808    0.087627   -4.080895  68.H        -5.908598   -3.333657   -3.750272  69.H         1.143166    1.446548   -4.228803  70.H        -0.101983   -1.146197   -4.048908  71.H        -3.066964    0.813362   -3.933323  72.H         6.606252   -0.788010   -3.376879  73.H         6.932659   -2.537125   -3.356120  74.H         4.366208    2.163885   -2.986822  75.H         1.747447   -2.215437   -2.905856  76.H         1.865050    0.390810   -3.023723  77.H        -5.363531   -2.079916   -2.613027  78.H        -5.203725    0.547516   -2.770156  79.H         4.284350    3.907763   -2.644459  80.H        -4.702611   -3.721821   -2.504692  81.H        -1.105909    5.043154   -2.502615  82.H        -4.700912    2.243388   -2.617126  83.H         2.299274   -3.859391   -2.555142  84.H         0.985063    6.527873   -2.434729  85.H        -1.460527   -4.134543   -2.617744  86.H         0.228321    1.038458   -2.762417  87.H         2.609152    5.815330   -2.510297  88.H        -2.353556    1.624861   -2.554484  89.H        -0.944765    3.276968   -2.503407  90.H         5.231107   -4.504697   -1.829624  91.H         5.565416    0.734510   -1.620758  92.H        -0.855674   -2.878237   -1.518809  93.H         1.821393   -2.803685   -1.231568  94.H        -2.513007   -3.506664   -1.337812  95.H         6.198001   -3.261960   -1.011920  96.H        -5.579947   -0.703535   -1.124263  97.H         1.620082    5.629648   -1.041721  98.H        -0.483505    4.236795   -1.066091  99.H         6.271274   -0.768817   -1.054776 100.H        -2.897333    3.294386   -1.074248 101.H        -5.357842    3.030501   -0.671742 102.H        -6.593116    0.623114   -0.487771 103.H         4.852444    2.501052   -0.717459 104.H         4.707363   -3.857332   -0.259421 105.H         3.178945    4.252975   -0.248840 106.H        -4.480687   -4.157071    0.138994 107.H        -3.428016    4.058393    0.408911 108.H         6.500875    0.956918    0.660611 109.H        -4.994894    2.204321    0.863297 110.H         5.138686    3.295158    0.850050 111.H        -5.988361   -3.661022    0.931225 112.H        -6.206056   -1.156524    1.141456 113.H         2.715977   -3.403372    1.074796 114.H         2.668555    3.427327    1.207743 115.H         0.053926    4.352273    1.051205 116.H         5.525526   -0.440561    1.198130 117.H        -1.672353   -3.014391    1.038536 118.H         1.007186   -2.968542    1.327889 119.H        -2.135038    5.553237    1.355209 120.H        -5.543615    0.353983    1.740058 121.H        -4.946900   -4.877040    1.696042 122.H         1.762550   -4.240479    2.310458 123.H         0.788187    5.065001    2.486196 124.H         0.731046    3.300993    2.336287 125.H         4.857942   -3.701420    2.353483 126.H         5.439678   -2.030763    2.482769 127.H        -1.407544    6.397901    2.736134 128.H        -2.021218   -4.088538    2.389783 129.H        -4.508547    3.566896    2.783700 130.H         2.139190    1.558100    2.614710 131.H         4.390558    2.418940    2.750505 132.H        -1.539367   -2.416330    2.705998 133.H        -2.958785    5.557268    2.932797 134.H         5.085072    0.790181    2.873042 135.H        -0.264530    0.986174    2.855200 136.H        -6.552002   -1.484684    3.394210 137.H        -6.642883   -3.261706    3.386561 138.H        -1.847073    0.195952    3.026458 139.H        -4.461728    1.828795    3.161069 140.H         6.042309   -3.271284    3.605720 141.H         2.899266    0.749128    3.970977 142.H        -4.330169   -0.309172    4.056629 143.H         0.146133   -1.345421    3.918386 144.H         3.287732   -4.682705    4.147836 145.H        -4.570059   -4.670401    4.139093 146.H        -3.783274    3.004858    4.299171 147.H        -1.256760    1.248239    4.306228 148.H        -6.523545   -2.381348    4.924947 149.H        -0.425918    5.196135    4.647259 150.H        -3.095915   -3.830724    4.678869 151.H         4.854105   -0.627053    4.599103 152.H        -0.336329    3.422896    4.719721 153.H        -2.947859   -1.277961    4.607769 154.H         0.774265   -2.720591    4.856996 155.H        -1.894752    4.246904    4.951020 156.H         4.541136   -4.285218    5.342018 157.H         1.323925   -1.071561    5.214880 158.H        -4.418490   -1.193482    5.598459 159.H         3.742700   -1.290926    5.820888 160.H         2.874905   -3.718862    5.585845 161.H        -4.586737   -3.720241    5.639608 162.H         5.378075   -1.960122    5.645519 163.Li       -0.064978    2.296177    0.057598 164.N        -2.638800   -0.466952   -2.245277 165.N         4.116681   -0.766551   -1.140661 166.N        -4.518530    1.067763   -0.833699 167.N         2.108908    2.389068   -0.576055 168.N         0.014905   -0.907864   -0.027552 169.N        -0.018745    0.402283    0.042663 170.N        -2.261393    2.244283    0.677448 171.N         4.394943    1.288386    0.930510 172.N        -4.051896   -1.056438    1.132082 173.N         2.645515   -0.474358    2.207036 174.Si       -2.356600   -1.871642   -3.247723 175.Si        2.028537    2.779888   -2.290490 176.Si        4.146284   -2.237561   -2.091539 177.Si       -3.971794   -2.569526    2.012663 178.Si       -2.186594    2.595574    2.400731 179.Si        2.435426   -1.954599    3.113751 180.U         2.242181   -0.013518    0.007267 181.U        -2.254982   -0.127996   -0.017722
