# Supplementary material for: Catalytic and stoichiometric stepwise conversion of side-on bound dinitrogen to ammonia mediated by a uranium complex
Source: Nat Chem. 2025 Jul 16;17(9):1425–33. doi: 10.1038/s41557-025-01867-z (PMC12411223; doi:10.1038/s41557-025-01867-z)
Supplement: Supplementary file 5 — Geometry optimized coordinates and single point energy of 2-K. [file 41557_2025_1867_MOESM5_ESM.xyz]

181Complex 2K. Energy: -978.74260183 eV   1.C         4.244060   -0.314787   -5.737597   2.C        -2.087182   -4.974977   -4.441829   3.C         3.948453   -0.877214   -4.329737   4.C         2.536857   -1.496036   -4.329145   5.C        -4.131845    2.249457   -4.204566   6.C         4.981928   -1.976779   -4.005276   7.C        -4.217596   -2.154419   -3.808505   8.C         5.627523    1.530389   -3.327762   9.C        -1.195838   -1.974084   -3.398494  10.C        -4.012082   -5.414510   -2.905133  11.C         2.548456    1.690534   -3.271439  12.C        -2.637453   -4.716025   -3.019222  13.C        -2.616333    5.208079   -2.876179  14.C        -1.288872    2.101705   -3.110356  15.C        -1.671014   -5.338601   -1.993324  16.C        -3.284019    4.196146   -1.918007  17.C        -4.771148    4.585561   -1.762880  18.C        -4.992521    1.079662   -1.177246  19.C         1.807485   -3.485854   -0.956478  20.C         5.221433   -0.567944   -0.800747  21.C        -5.442334   -0.289699   -0.667590  22.C        -2.597777    4.297461   -0.540735  23.C        -4.145213   -2.953080   -0.431547  24.C         1.388363    4.093175   -0.457114  25.C         4.409759    3.857789   -0.177111  26.C         5.651351    0.177990    0.464003  27.C        -4.667427   -2.162685    0.769566  28.C         3.824009   -4.593066    1.057380  29.C         2.707835    6.488026    1.358455  30.C         0.720988   -5.931598    1.385785  31.C        -0.525433   -3.759685    1.534932  32.C        -4.944807    0.092710    1.724059  33.C         0.814722   -4.457037    1.836616  34.C         2.753662    5.136492    2.111460  35.C        -3.805979    1.083207    1.984038  36.C         4.720966   -1.224835    2.252839  37.C         4.562439    1.223789    2.453654  38.C         3.116039    1.691397    2.662277  39.C         3.347143   -1.772024    2.635973  40.C         4.013634    5.148358    3.007273  41.C         1.499704    5.042648    3.006971  42.C         1.069646   -4.417181    3.358648  43.C        -0.138893   -0.379950    3.705555  44.C        -2.767088   -1.882378    4.124106  45.C        -2.085792    2.471927    4.833177  46.C        -2.475756    1.013074    5.154273  47.C        -3.970428    0.957242    5.547040  48.C        -1.663561    0.567533    6.394944  49.H         4.137224   -1.113338   -6.494366  50.H         3.547221    0.491473   -6.017307  51.H         5.267915    0.080724   -5.820248  52.H        -2.731850   -4.540595   -5.222991  53.H         2.487681   -2.348652   -5.030838  54.H        -3.802828    2.992105   -4.948969  55.H        -4.100543   -2.467704   -4.858585  56.H        -2.033798   -6.062943   -4.631479  57.H         4.937974   -2.779626   -4.763834  58.H        -4.006350    1.253079   -4.655942  59.H        -1.073518   -4.567504   -4.575560  60.H         1.771148   -0.769457   -4.640887  61.H         5.561184    2.058728   -4.292095  62.H        -1.177803   -2.023893   -4.497913  63.H         6.013449   -1.589271   -4.001246  64.H         2.425975    1.944084   -4.336006  65.H        -5.208516    2.407341   -4.041735  66.H        -4.740548   -5.014555   -3.627596  67.H        -3.053477    5.169214   -3.886116  68.H        -4.242772   -1.054185   -3.787379  69.H        -5.200419   -2.520266   -3.471747  70.H         6.524911    0.893900   -3.370962  71.H        -1.182727    1.160055   -3.668316  72.H        -0.933348    2.907427   -3.769925  73.H        -3.902758   -6.494739   -3.114438  74.H         2.253228   -1.871224   -3.335119  75.H        -1.126811   -0.910504   -3.123937  76.H         4.790410   -2.439863   -3.025751  77.H         1.599536    1.237527   -2.943395  78.H        -1.533365    5.036776   -2.970702  79.H        -5.308323    4.543710   -2.722511  80.H        -0.281927   -2.453900   -3.023773  81.H        -2.756353    6.238363   -2.499856  82.H         5.795037    2.289336   -2.548183  83.H         2.679858    2.635955   -2.725087  84.H        -4.449835   -5.322054   -1.900384  85.H        -1.550767   -6.421001   -2.183430  86.H        -0.610640    2.055149   -2.245759  87.H        -5.600442    1.290500   -2.075844  88.H        -0.671235   -4.882571   -2.045012  89.H        -4.856482    5.621550   -1.385488  90.H         6.059920   -0.495545   -1.514959  91.H        -5.279021   -1.025794   -1.461779  92.H         2.664384   -3.116543   -1.540374  93.H         1.568741   -4.491217   -1.334092  94.H        -5.302507    3.934472   -1.053385  95.H        -4.977995   -3.085523   -1.149322  96.H         1.737990    4.789764   -1.232855  97.H        -2.035366   -5.221044   -0.962418  98.H         0.946861   -2.835185   -1.164298  99.H         4.474496    3.053508   -0.924045 100.H         1.025825    3.187953   -0.965798 101.H         4.471003    4.819063   -0.711214 102.H         5.125572   -1.644828   -0.574225 103.H        -6.523915   -0.270889   -0.420951 104.H        -1.525688    4.055706   -0.617651 105.H        -5.294060    1.847136   -0.435972 106.H        -2.678395    5.324287   -0.137459 107.H        -3.916927   -3.971400   -0.064545 108.H         0.524023    4.583348    0.019345 109.H         5.760527    1.239491    0.210726 110.H        -3.060743    3.612202    0.187403 111.H         0.541833   -6.025392    0.303558 112.H         5.298771    3.782851    0.467458 113.H        -0.785192   -3.787003    0.468035 114.H         3.665677   -5.594440    0.626472 115.H         6.630192   -0.193219    0.829628 116.H         1.811658    6.587282    0.727304 117.H         3.589220    6.627782    0.713891 118.H        -5.681093   -2.512639    1.051360 119.H         4.661150   -4.132526    0.508765 120.H        -3.802987    1.808472    1.147862 121.H        -5.912395    0.613118    1.609924 122.H         1.633536   -6.497263    1.629736 123.H        -0.121530   -6.431384    1.899084 124.H        -4.010363   -2.328976    1.634630 125.H         5.189096   -1.954090    1.579428 126.H         2.696883    7.323996    2.081774 127.H        -0.504820   -2.703718    1.842991 128.H        -1.346326   -4.253544    2.086603 129.H         5.141933    2.048882    2.019002 130.H         4.145241   -4.737382    2.100795 131.H         4.935630    5.217187    2.410744 132.H         0.570236    5.121264    2.416195 133.H        -5.038760   -0.589965    2.579513 134.H         0.270318   -0.804476    2.773954 135.H        -4.061141    1.680992    2.875701 136.H         5.380674   -1.109346    3.136060 137.H         3.505645   -2.730832    3.159694 138.H         2.558012    0.861833    3.138077 139.H         5.033218    0.962715    3.420405 140.H         3.122040    2.488341    3.428397 141.H         1.472624    4.107465    3.589099 142.H         2.873382   -1.116175    3.388464 143.H         3.994146    6.026673    3.678190 144.H         4.095085    4.256309    3.645214 145.H        -2.461427   -2.648629    3.396078 146.H         2.010287   -4.920294    3.634366 147.H         1.473236    5.875144    3.732820 148.H         1.111559   -3.384601    3.736808 149.H         0.254099   -4.934006    3.897748 150.H        -3.867226   -1.872810    4.162087 151.H         0.384330    0.571002    3.899950 152.H        -2.632025    2.873723    3.965426 153.H         0.150302   -1.059148    4.521152 154.H        -1.002763    2.568477    4.644238 155.H        -4.641533    1.314551    4.752542 156.H        -2.408360   -2.214230    5.112168 157.H        -2.308463    3.134308    5.688992 158.H        -4.280651   -0.065587    5.807986 159.H        -0.577611    0.646416    6.235980 160.H        -4.153323    1.589691    6.435201 161.H        -1.885794   -0.471982    6.680958 162.H        -1.918626    1.204969    7.261265 163.K        -0.408642    2.418720    1.758957 164.N        -3.541639    1.140442   -1.460206 165.N         3.958556   -0.020691   -1.348903 166.N        -2.975668   -2.311455   -1.055512 167.N         0.117239   -0.486474   -0.713373 168.N        -0.011494    0.684610   -0.117472 169.N        -4.641267   -0.706702    0.507744 170.N         2.446920    2.092208    1.410265 171.N         4.610220    0.066737    1.521247 172.N         2.508117   -1.899083    1.431397 173.N        -2.484555    0.419264    2.078493 174.Si        4.029226    0.539354   -3.012888 175.Si       -2.769895   -2.794218   -2.743074 176.Si       -3.091977    2.384425   -2.616255 177.Si        2.775855    3.697295    0.785282 178.Si        2.235792   -3.548089    0.884242 179.Si       -2.031098   -0.185456    3.665052 180.U        -2.158679   -0.275871   -0.252352 181.U         2.213293    0.001304    0.159871 
