# Supplementary material for: Catalytic and stoichiometric stepwise conversion of side-on bound dinitrogen to ammonia mediated by a uranium complex
Source: Nat Chem. 2025 Jul 16;17(9):1425–33. doi: 10.1038/s41557-025-01867-z (PMC12411223; doi:10.1038/s41557-025-01867-z)
Supplement: Supplementary file 6 — Geometry optimized coordinates and single point energy of 3A. [file 41557_2025_1867_MOESM6_ESM.xyz]

182Complex 3A. Energy: - 982.55117874 eV   1.C         0.303152   -1.899523   -6.274370   2.C         2.683139   -1.419302   -5.672003   3.C         1.220505   -1.297005   -5.186007   4.C         0.879032    0.193276   -5.019524   5.C        -2.971891    3.150915   -4.783384   6.C         1.194245   -4.073612   -3.852089   7.C        -3.325148    0.880421   -3.789601   8.C        -3.413301    2.390669   -3.511831   9.C        -1.636608   -2.342631   -3.409716  10.C        -4.891583    2.750914   -3.231742  11.C         0.029492    3.129719   -3.147541  12.C         1.500392    3.443808   -2.886411  13.C        -2.830147   -2.715872   -2.538639  14.C         2.490584   -1.735335   -2.371551  15.C        -2.562015    4.745661   -1.740450  16.C        -1.816251   -4.781589   -1.654730  17.C         3.213336    4.073511   -1.285855  18.C         1.024885    5.180271   -1.183882  19.C         3.822171    2.722371   -0.929217  20.C         2.594636   -5.717295   -0.686855  21.C        -3.603909   -3.618628   -0.428596  22.C        -0.990143   -5.342410   -0.491131  23.C        -3.170206    2.051157   -0.453237  24.C        -3.849718   -2.336871    0.365581  25.C         0.869722    5.362708    0.331147  26.C         6.448621    1.047024    0.784645  27.C         2.362588   -3.461116    1.408551  28.C         4.759399   -0.758614    1.203610  29.C         0.780932   -7.649999    1.530073  30.C        -2.330835    5.532122    1.907303  31.C         5.286302    0.599600    1.699267  32.C         1.360323   -6.326450    2.079403  33.C         4.552546    3.538275    2.324510  34.C         2.771664   -6.609891    2.644596  35.C        -3.241309   -4.256219    3.037893  36.C        -1.358596    2.830223    3.005344  37.C         5.832385    0.436779    3.135966  38.C         0.464641   -5.827758    3.228574  39.C         2.458854    1.280838    2.846966  40.C         0.521486    6.888783    3.441003  41.C        -5.397025   -1.665529    3.609640  42.C        -0.943259   -2.224884    3.486814  43.C        -3.645551    0.123440    3.630444  44.C         0.061128    5.472351    3.855165  45.C        -3.910389   -1.367981    3.904858  46.C         1.279505    4.679610    4.363593  47.C        -0.952739    5.617678    5.012904  48.C        -3.645955   -1.653114    5.400871  49.H         0.461552   -1.375265   -7.235019  50.H         2.794613   -0.938691   -6.661292  51.H         0.514756   -2.965420   -6.448099  52.H        -0.763641   -1.805096   -6.025370  53.H         1.040260    0.745570   -5.963463  54.H         2.995669   -2.469385   -5.784860  55.H        -3.657015    2.916723   -5.618603  56.H        -1.958640    2.872310   -5.105741  57.H         3.391320   -0.929821   -4.986635  58.H        -2.993215    4.242429   -4.642602  59.H        -0.175517    0.335568   -4.735563  60.H         2.162430   -4.270688   -4.339616  61.H         1.505873    0.670359   -4.250254  62.H        -3.942785    0.604862   -4.663867  63.H         0.414956   -4.461228   -4.525228  64.H        -2.009899   -1.605655   -4.143606  65.H        -5.515606    2.486031   -4.104757  66.H         0.003423    2.577338   -4.103252  67.H        -1.362942   -3.226741   -4.013784  68.H        -2.293386    0.565202   -4.008810  69.H         1.863320    4.171472   -3.641838  70.H        -5.028532    3.827617   -3.049858  71.H        -0.483569    4.085678   -3.362469  72.H        -3.573571   -3.284860   -3.134558  73.H         2.101852    2.529552   -3.011457  74.H         1.169561   -4.663345   -2.924956  75.H        -3.685502    0.284683   -2.936653  76.H        -2.227642    5.334374   -2.607717  77.H         3.435450   -2.013172   -2.862500  78.H        -1.145488   -4.624379   -2.506068  79.H        -5.297973    2.207985   -2.364383  80.H        -3.327556   -1.797762   -2.194252  81.H         3.697306    4.536961   -2.170373  82.H        -2.592851   -5.510998   -1.962812  83.H         2.519005   -0.666208   -2.124638  84.H        -3.628852    4.974748   -1.592317  85.H         3.851890    2.071831   -1.824325  86.H         0.028542    5.115835   -1.629524  87.H         1.539877    6.058476   -1.623722  88.H         2.072699   -6.493630   -1.268469  89.H         2.467345   -2.275839   -1.412744  90.H         2.911109   -4.935063   -1.392730  91.H        -2.023601    5.105759   -0.853936  92.H        -4.501462   -3.914952   -1.010128  93.H        -0.511001   -6.259715   -0.885519  94.H         4.882205    2.897664   -0.672206  95.H         3.354043    4.746786   -0.430519  96.H         3.505212   -6.177001   -0.271082  97.H        -4.177095    2.465254   -0.300256  98.H        -3.263043    0.965904   -0.618199  99.H        -4.164390   -1.534244   -0.327944 100.H         6.147353    1.091633   -0.272244 101.H        -3.383219   -4.425457    0.280916 102.H        -1.676539   -5.696624    0.303696 103.H        -2.589425    2.246857    0.461892 104.H         0.280623    6.291776    0.459752 105.H         2.274098   -2.553560    0.783583 106.H         4.318392   -0.680337    0.199084 107.H         1.379430   -8.049448    0.696764 108.H         1.861990    5.586507    0.768376 109.H         7.282348    0.324203    0.854264 110.H        -4.738432   -2.518134    0.996728 111.H        -0.254920   -7.535645    1.178003 112.H         5.577417   -1.501093    1.162016 113.H        -2.090447    6.498404    1.436459 114.H         5.328028    3.927995    1.645170 115.H         6.849614    2.032780    1.068169 116.H        -2.959905    4.970305    1.201291 117.H         3.453436   -7.012628    1.879932 118.H         3.443419   -3.642818    1.503657 119.H         1.988957   -3.203128    2.410713 120.H         0.772831   -8.418525    2.324826 121.H         3.986320   -1.160423    1.873682 122.H        -2.552682   -4.938474    2.518012 123.H        -2.941938    5.746916    2.798320 124.H        -5.673735   -1.415135    2.574090 125.H         5.012701    3.441508    3.320508 126.H         3.763907    4.302139    2.392521 127.H        -4.254211   -4.441165    2.647483 128.H         1.314935    6.861868    2.680401 129.H         2.708731   -7.360099    3.454006 130.H        -0.307960    7.494845    3.044629 131.H        -3.960076    0.405118    2.608849 132.H        -0.847646    1.974858    2.522863 133.H        -2.438970    2.704372    2.839047 134.H        -0.242229   -2.852587    2.913353 135.H        -0.552656   -5.596838    2.883292 136.H         1.999566    0.352688    2.468306 137.H         6.636010   -0.322700    3.151830 138.H         3.237386   -5.707595    3.070723 139.H         6.261423    1.373003    3.525991 140.H         0.869860   -4.921152    3.702721 141.H         1.671484    2.043612    2.973667 142.H         2.030226    4.536667    3.574853 143.H        -5.650308   -2.722360    3.781315 144.H        -0.588153   -1.183835    3.433010 145.H        -2.586426    0.392403    3.764714 146.H         5.055321    0.101815    3.840920 147.H         0.382808   -6.598595    4.016545 148.H        -3.246972   -4.541441    4.101796 149.H        -6.045581   -1.062655    4.271647 150.H         2.835893    1.069459    3.858758 151.H         0.927181    7.425660    4.318043 152.H        -1.185072    2.714511    4.085813 153.H        -0.864092   -2.548618    4.535334 154.H        -4.225629    0.767874    4.315308 155.H        -1.813466    6.243882    4.733723 156.H         0.996305    3.683692    4.738086 157.H         1.768785    5.213959    5.198325 158.H        -1.340941    4.646361    5.357797 159.H        -2.619392   -1.392462    5.699497 160.H        -3.810537   -2.712404    5.652267 161.H        -0.465349    6.099950    5.880107 162.H        -4.333450   -1.058423    6.029775 163.Li       -0.458890    0.257799   -2.025468 164.Li       -2.247478    0.018916    1.066651 165.N        -0.485817   -1.829774   -2.627689 166.N        -0.627867    2.379998   -2.042993 167.N         1.752611    3.930656   -1.514237 168.N        -2.436178   -3.470270   -1.329030 169.N         0.681017   -0.142519   -0.313444 170.N        -0.016058   -4.374831    0.038688 171.N         3.044831    2.106366    0.151565 172.N        -0.640609    0.178415    0.170978 173.N         0.247470    4.200255    0.995108 174.N        -2.650230   -1.934972    1.136465 175.Si        1.028159   -2.211307   -3.473961 176.Si       -2.325486    2.866498   -1.960508 177.Si        1.492445   -4.984829    0.680413 178.Si        3.838678    1.889991    1.685531 179.Si       -0.774428    4.534874    2.369891 180.Si       -2.703792   -2.443415    2.814936 181.U        -0.493803   -2.086624   -0.195659 182.U         0.714770    2.082159    0.059692 
