# Supplementary material for: Catalytic and stoichiometric stepwise conversion of side-on bound dinitrogen to ammonia mediated by a uranium complex
Source: Nat Chem. 2025 Jul 16;17(9):1425–33. doi: 10.1038/s41557-025-01867-z (PMC12411223; doi:10.1038/s41557-025-01867-z)
Supplement: Supplementary file 7 — Geometry optimized coordinates and single point energy of 3B. [file 41557_2025_1867_MOESM7_ESM.xyz]

182Complex 3B.Energy: -982.40504459 eV    1.C        -3.119706   -2.355668   -6.683815   2.C        -0.977325   -3.643334   -6.577643   3.C        -0.408652   -0.253872   -6.215424   4.C        -2.008064   -2.856057   -5.733571   5.C        -2.627403   -3.817575   -4.701180   6.C        -0.280437    3.372913   -4.819479   7.C        -3.272482    0.085224   -4.218823   8.C         0.395869   -2.123252   -3.921382   9.C        -3.646567    1.468667   -3.675513  10.C         2.386515    4.990048   -3.202244  11.C         0.157467    5.989193   -2.666404  12.C         1.579126    1.758775   -2.960250  13.C         3.898236   -2.076129   -2.719515  14.C         1.073209    4.768583   -2.416510  15.C         4.910618   -4.133890   -1.706304  16.C         6.226690   -2.005738   -1.797498  17.C        -5.212558   -2.660016   -1.789950  18.C        -2.231263    3.511106   -2.038796  19.C         4.813490   -2.595286   -1.595138  20.C        -4.891774    0.833134   -1.641219  21.C        -3.554119    2.860549   -1.636736  22.C         1.397437    4.684665   -0.914281  23.C        -2.306792   -3.288223   -0.923250  24.C        -4.602664    0.259922   -0.252888  25.C         4.551251    0.544939    0.271551  26.C         2.549281   -3.239507    0.444785  27.C        -4.621264   -4.664609    1.068283  28.C        -4.583661   -3.124766    1.194735  29.C         5.386369   -2.501339    1.433235  30.C        -6.009308   -2.628721    1.522294  31.C         4.818291    0.951561    1.721555  32.C        -2.064324    4.058574    1.626648  33.C         3.325288    2.847710    2.002510  34.C        -3.652738   -2.750101    2.363173  35.C         1.962483    3.329896    2.498036  36.C        -1.497784    4.159272    3.055319  37.C        -0.657171    5.451385    3.171294  38.C        -1.628416    1.088748    3.266504  39.C        -0.128619   -2.756707    3.516976  40.C         3.558963    1.202797    3.829172  41.C        -2.673559    4.261921    4.053936  42.C         2.988248   -4.265418    4.160898  43.C         3.305388   -0.267940    4.179000  44.C         0.110625    2.693577    5.261181  45.C         2.303862   -3.476622    5.294317  46.C         1.344062   -4.432117    6.044458  47.C         3.381726   -3.001904    6.294430  48.C         0.462844   -1.117803    6.063651  49.H        -2.737198   -1.642546   -7.430704  50.H        -3.552497   -3.208429   -7.238885  51.H        -0.513414   -3.020251   -7.357808  52.H        -1.478088   -4.486012   -7.088901  53.H        -1.199348    0.345547   -6.693055  54.H         0.088664   -0.830264   -7.011101  55.H        -3.943573   -1.869195   -6.142072  56.H        -0.170647   -4.068543   -5.960349  57.H         0.331021    0.449873   -5.804316  58.H         0.573041    3.694893   -5.437124  59.H        -3.134867   -4.656086   -5.212440  60.H        -3.233259    0.212451   -5.317534  61.H        -0.669804    2.439946   -5.251260  62.H        -1.060418    4.142691   -4.925710  63.H         2.215016    5.054628   -4.288545  64.H         1.171173   -2.457173   -4.626121  65.H        -3.371517   -3.315184   -4.067739  66.H        -1.865860   -4.254539   -4.037243  67.H        -0.109635    6.096289   -3.729075  68.H        -4.115418   -0.610571   -4.042418  69.H        -4.618479    1.792733   -4.100944  70.H        -2.878917    2.181909   -3.994470  71.H         4.249341   -2.410004   -3.712416  72.H         2.369125    1.954024   -3.700000  73.H         2.858156    5.939959   -2.889967  74.H         0.853211   -1.364713   -3.267680  75.H         3.119883    4.187985   -3.024201  76.H         0.123021   -2.975194   -3.282264  77.H         0.677225    6.917421   -2.365108  78.H         6.640899   -2.331426   -2.769268  79.H         5.377017   -4.419409   -2.666917  80.H         1.141347    0.775557   -3.202939  81.H        -2.366254    3.986389   -3.029918  82.H        -4.856006   -2.344862   -2.781727  83.H        -0.776348    5.938491   -2.087951  84.H         2.864233   -2.445701   -2.618214  85.H         3.889810   -0.971381   -2.755625  86.H        -5.156814    0.005695   -2.309569  87.H        -5.455496   -3.732644   -1.855129  88.H         3.922701   -4.616199   -1.663423  89.H         6.223991   -0.905197   -1.801186  90.H        -4.407927    3.499802   -1.939671  91.H         2.034176    1.729474   -1.956695  92.H        -1.925298   -2.906436   -1.883445  93.H        -6.153043   -2.126335   -1.584606  94.H        -5.748261    1.538056   -1.619664  95.H         6.927564   -2.338532   -1.017348  96.H         5.529739   -4.565528   -0.904346  97.H        -2.074015    4.350025   -1.336715  98.H        -2.497558   -4.362983   -1.059484  99.H         1.880255    5.617101   -0.568121 100.H         2.086656    3.854519   -0.694841 101.H        -3.595368    2.766524   -0.541762 102.H         0.494369    4.521944   -0.309372 103.H         1.758101   -3.123039   -0.311408 104.H         4.304065    1.445817   -0.319138 105.H         5.511267    0.193084   -0.147099 106.H        -1.504044   -3.174011   -0.178985 107.H        -5.256150   -4.993678    0.231312 108.H        -5.534738   -0.217597    0.099685 109.H        -4.431975    1.091595    0.455178 110.H        -3.619292   -5.093440    0.918447 111.H        -6.723063   -2.863788    0.718282 112.H         6.278437   -1.866746    1.319251 113.H         3.356897    2.907093    0.904129 114.H         2.843132   -4.299186    0.470690 115.H         2.106810   -3.006408    1.426011 116.H        -1.276923    3.859099    0.884955 117.H         5.720244   -3.547919    1.351683 118.H         5.615313    1.720208    1.788696 119.H        -5.037987   -5.113123    1.989465 120.H        -6.042102   -1.542395    1.696507 121.H        -2.577854    4.995382    1.343850 122.H        -2.804057    3.246530    1.547435 123.H         1.723941    4.234219    1.911305 124.H        -2.606385   -3.041077    2.174182 125.H         5.159488    0.067230    2.271998 126.H        -6.381394   -3.115315    2.442148 127.H         4.999476   -2.358343    2.452375 128.H         4.131768    3.506581    2.383602 129.H        -3.697673   -1.667856    2.584207 130.H         0.123817    5.510055    2.399891 131.H        -2.139644    1.165305    2.292835 132.H        -3.952814   -3.258900    3.296823 133.H        -1.307761    6.336115    3.043380 134.H         0.211677   -3.642044    2.957941 135.H         2.077542    3.683572    3.540970 136.H        -0.494678   -2.038092    2.762760 137.H         2.258668   -4.688222    3.453295 138.H        -1.094684    0.126412    3.340709 139.H         3.682994   -3.637946    3.585940 140.H         4.203799   -0.859083    3.915550 141.H        -3.306035    5.133725    3.803689 142.H        -3.322441    3.372058    4.029927 143.H         4.508179    1.543754    4.290699 144.H        -0.170113    5.545692    4.154446 145.H        -2.391028    1.096413    4.059443 146.H         2.742550    1.802127    4.245284 147.H        -0.984349   -3.059064    4.139997 148.H         3.566887   -5.110482    4.576362 149.H        -2.326480    4.398358    5.089697 150.H         3.261080   -0.293814    5.284606 151.H         0.739165    3.577030    5.453211 152.H         0.682840    1.802254    5.557230 153.H         0.548228   -4.822895    5.391366 154.H        -0.758481    2.766011    5.933465 155.H         4.163055   -2.398442    5.810360 156.H        -0.300066   -0.398917    5.729122 157.H         1.908423   -5.300813    6.430326 158.H         3.882116   -3.875863    6.751844 159.H        -0.026592   -1.828721    6.747781 160.H         0.864945   -3.946024    6.908118 161.H         1.203077   -0.555431    6.654519 162.H         2.953634   -2.406427    7.116244 163.Li        2.156646   -0.429415   -1.334293 164.Li       -2.136511   -0.738106    1.244990 165.N        -2.010339   -0.464963   -3.670728 166.N        -3.689550    1.497618   -2.192487 167.N        -1.111500    2.554167   -2.033194 168.N        -3.440036   -0.658883   -0.277486 169.N         0.463530   -0.352078   -0.570069 170.N         3.464469   -0.458616    0.183143 171.N        -0.452125   -0.453522    0.510607 172.N         0.925053    2.291407    2.378691 173.N         3.576852    1.437202    2.364029 174.N         2.093539   -0.845193    3.553009 175.Si       -1.089224   -1.387172   -4.843747 176.Si        0.234814    3.115162   -3.002417 177.Si       -3.889042   -2.353415   -0.457111 178.Si        4.047269   -2.119889    0.136110 179.Si       -0.438497    2.575435    3.438888 180.Si        1.256588   -2.000932    4.573642 181.U        -1.449493    0.279460   -1.509099 182.U         1.425370    0.152436    1.531537
